# Integrated analysis of mRNA and miRNA expression in HeLa cells expressing low levels of Nucleolin

**DOI:** 10.1038/s41598-017-09353-4

**Published:** 2017-08-21

**Authors:** Sanjeev Kumar, Elizabhet Cruz Gomez, Mounira Chalabi-Dchar, Cong Rong, Sadhan Das, Iva Ugrinova, Xavier Gaume, Karine Monier, Fabien Mongelard, Philippe Bouvet

**Affiliations:** 1 Université de Lyon, Centre de Recherche en Cancérologie de Lyon, Cancer Cell Plasticity Department, UMR INSERM 1052 CNRS, 5286 Centre Léon Bérard, Lyon France; 2 Université de Lyon, Ecole Normale Supérieure de Lyon, Lyon, France; 3 BioCOS Life Sciences Private Limited, AECS Layout, B-Block, Singasandra Hosur Road SAAMI Building, 851/A, 3rd Floor, Bengaluru, Karnataka India; 4 Institute of Molecular Biology “Acad. Roumen Tsanev” Bulgarian Academy of Sciences “Acad. G Bonchev str. bl. 21, 1113 Sofia, Bulgaria

## Abstract

Nucleolin is an essential protein that plays important roles in the regulation of cell cycle and cell proliferation. Its expression is up regulated in many cancer cells but its molecular functions are not well characterized. Nucleolin is present in the nucleus where it regulates gene expression at the transcriptional and post-transcriptional levels. Using HeLa cells depleted in nucleolin we performed an mRNA and miRNA transcriptomics analysis to identify biological pathways involving nucleolin. Bioinformatic analysis strongly points to a role of nucleolin in lipid metabolism, and in many signaling pathways. Down regulation of nucleolin is associated with lower level of cholesterol while the amount of fatty acids is increased. This could be explained by the decreased and mis-localized expression of the transcription factor SREBP1 and the down-regulation of enzymes involved in the beta-oxidation and degradation of fatty acids. Functional classification of the miRNA-mRNA target genes revealed that deregulated miRNAs target genes involved in apoptosis, proliferation and signaling pathways. Several of these deregulated miRNAs have been shown to control lipid metabolism. This integrated transcriptomic analysis uncovers new unexpected roles for nucleolin in metabolic regulation and signaling pathways paving the way to better understand the global function of nucleolin within the cell.

## Introduction

Nucleolin (NCL) is a highly conserved protein in eukaryotes with multiple functions in the cells^1^. The modular structure of NCL protein allows protein-protein interaction through its N- and C-terminal domains and interaction with nucleic acids with the central 4 RNA binding domains^2, 3^. NCL protein is the target of many post-translational modifications, like phosphorylation, methylation, acetylation, glycosylation that regulate its function^4^.

Initially described as a nucleolar protein involved in several steps or ribosome biogenesis, it is now well demonstrated that NCL is present in many cell compartments where it can play very different functions^5^.

NCL has gained a lot of interest recently as it could be a therapeutic target for some cancers^5, 6^. An altered expression of NCL has been observed in many cancers. For example, in colorectal and in breast cancer cells, NCL expression is increased by two to six-fold^7^. This deregulated expression of NCL is often associated with a broader localization of NCL in different cell compartments. A high cytoplasmic amount of NCL is associated with worse prognosis for patients with gastric or pancreatic cancer^8, 9^ and for elderly patients with acute myeloid lymphoma (AML)^10^, while, in glioblastoma cells, the presence of glycosylated NCL at the cell surface increases with the malignancy of the tumor^11^. The presence of this extracellular form of NCL seems to be a hallmark of proliferative and cancer cells^5^ and is now the target of several molecules that have anti-tumoral activities^12^.

One major question now is to understand the molecular function of NCL in normal and cancer cells. In the nucleoli, NCL participates to the production of ribosomal RNA by RNA polymerase I (RNAPI), and a growing number of studies found NCL involved in the regulation of transcription by RNA polymerase II (RNAPII)^4^. NCL is one component of LR1, a B cell-specific transcription factor^13^; it was also found in mantle cell lymphoma (MCL) to bind sites within the cyclin D1 gene and to activate transcription of this gene^14^. NCL can also activate endogenous *CD34* and *Bcl2* gene expression in human CD34- positive hematopoietic cells^15^. NCL could also be a transcriptional repressor like for the acute-phase response gene alpha-1 acid glycoprotein (AGP)^16^.

NCL interacts with chromatin, and facilitates RNAPII transcription through the nucleosomal structure *in vitro* ^17^. This FAcilitation of Chromatin Transcription (FACT activity) is likely the consequence of the histone chaperone properties of NCL. Indeed, *in vitro*, NCL is able to increase the efficiency of SWI/SNF and ACF, two chromatin remodelers and it participates in nucleosome disruption during transcription^18^. In live cells, NCL also plays a role in chromatin accessibility^19^. FRAP experiments on eGFP-tagged histones (H2B, H4 and macroH2A) showed that nuclear histone dynamics was impacted in NCL-silenced cells^19^.

In addition to its interaction with chromatin, NCL interacts with a large number of mRNAs^20^ and could regulate their processing, stability or translation. The co-localization of a modified acetylated form of NCL with SC35 in nuclear speckles suggests a participation of NCL to pre-mRNA splicing^21^; Indeed, NCL was found in RNP complexes formed on a specific HIV pre-mRNA splicing site suggesting that NCL may be involved in the splicing of this pre-mRNA^22^. In addition, NCL has been found associated with DGCR8 and Drosha, the core components of the microprocessor complex and to be involved in the biogenesis of microRNA 15a/16 (miR-15a/16)^23^, miR-21, miR-221, miR-222, and miR-103, that are involved in breast cancer initiation, progression, and drug resistance^24^. This role of NCL in RNA metabolism is reinforced by the fact that the nuclear fraction of NCL interacts with many factors involved in RNA processing, stability and transport^25^.

The expression of NCL is required for cell viability^26, 27^. Depletion of NCL in cultured cells induces a rapid decrease of transcription by RNA Polymerase I, an abrupt cessation of proliferation and cell division, an increase in apoptosis, and alteration of the centrosome function leading to multipolar spindle structures^27, 28^.

The molecular mechanisms that lead to cell proliferation arrest and cell death are not known. It is possible that the nucleolar depletion of NCL induce a nucleolar stress that activates processes that promote cell death, but it is also possible that the nuclear function of NCL on RNAP II transcription or mRNA accumulation contributes to this phenotype.

In this work, we addressed this question by determining the transcriptome of HeLa cells that have been depleted in NCL by siRNA. Interestingly, we found that many mRNAs coding for proteins involved in metabolic pathways are down regulated in cells expressing low levels of NCL, while mRNA coding for several signalling pathways are up-regulated. We also observed that many miRNAs are either up- or down regulated. We demonstrate that, in NCL depleted cells, the level of cholesterol is down-regulated while fatty acids accumulate in the cells. These observations agree with the transcriptomic analysis that shows the down regulation of mRNAs coding for enzymes implicated in cholesterol biosynthesis and in fatty acids degradation. Interestingly, we found that, the level of SREBP1, a major transcription factor involved in the regulation of many of these enzymes is not only down regulated upon NCL silencing, but also its cellular localization is impaired which could also explain the down-regulation of the downstream targets. The identification of the major cellular pathways that are affected by the low expression of NCL is important to understand the multifunctional properties of NCL in the cells.

This global transcriptomic and bioinformatic study provides a new view about the functions of NCL in the cell and suggests that NCL is a key factor to connect ribosome biogenesis in the nucleoli with cell metabolism and signalling pathways to support cell proliferation.

## Results and Discussion

**Figure 1:**
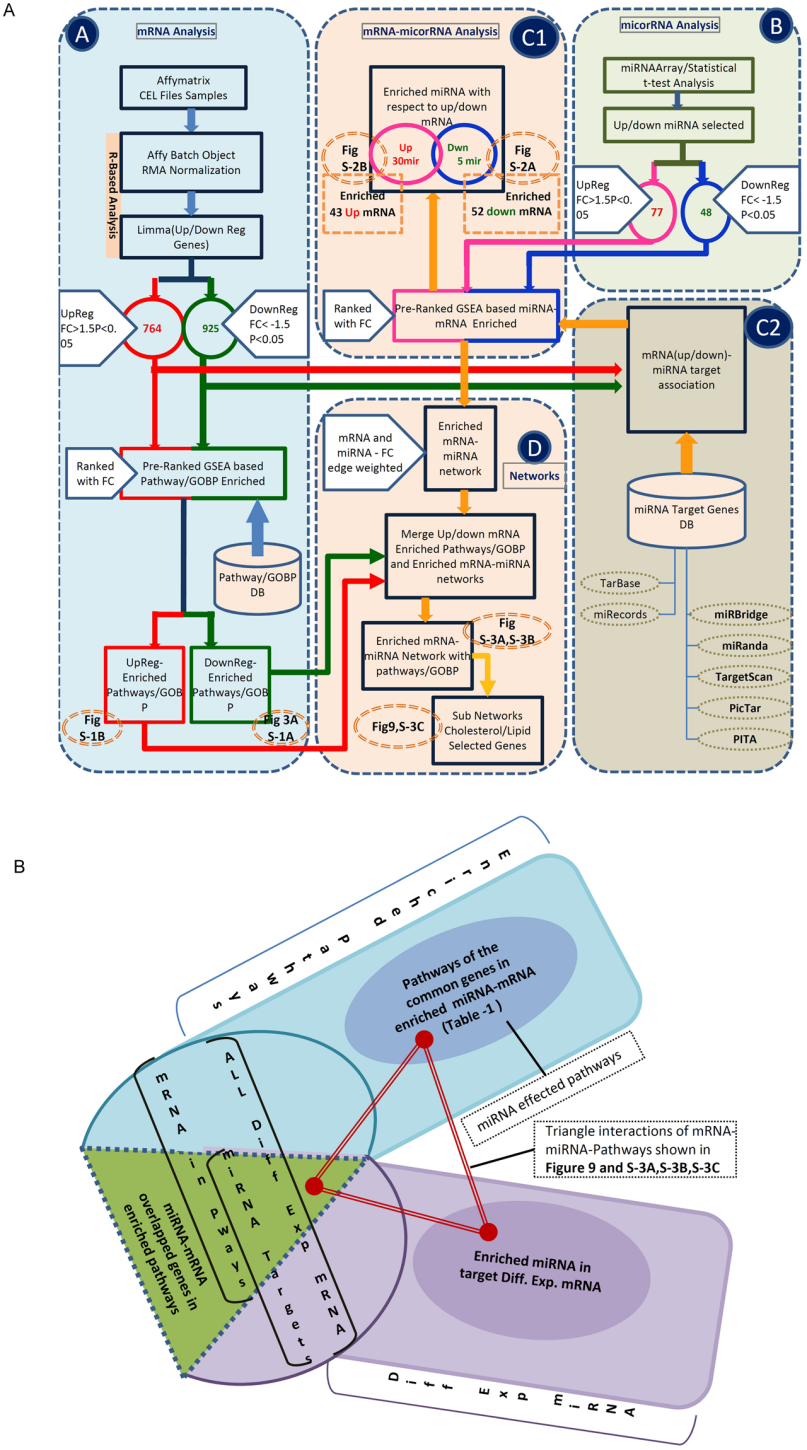
Integrated flow diagram of the mRNA and miRNA analysis. Color code: green and red color lines are for the down and up regulated mRNA analysis flow respectively; blue and pink lines are for the down and up regulated microRNA analysis flow respectively. The orange lines depict the mRNA-miRNA enrichment and network analysis flow. The numbers in the circles show the number of up/down regulated mRNA or miRNA. The figure numbers indicated in the dotted circles with orange color are where the output results of these analysis blocks are shown. Block (**A**) shows the differential gene expression analysis and pre-rank GSEA based transcriptome data processing, pathways and GO biological process (GOBP) enrichments. The flows in red and green show the pathways/GOBP enrichment for the up and down regulated genes analysis respectively. The numbers in the circles show the number of differentially expressed up (red) and down (greed) genes with the selected FC > 1.5 and p-value < 0.05. The bottom part shows the flow of pathway enrichment analysis using the KS statistics using GSEA^50^. Block (**B**) shows the microarray data analysis of miRNA. The t-test is used to obtain the differentially expressed miRNA as shown in the circles up (pink) and down (blue) with the selected FC/p-value parameters. The flow in pink and blue color shows the up and down regulated miRNA analysis and related data. Block (C1) and (C2) show the flow of enriching and finding the target genes for the enriched miRNAs for the differentially regulated mRNAs. In (C2), the target genes are taken from various resources as shown while in (C1) the pre-ranked GSEA tool (KS based statistics) is used for the association of the target genes to the miRNAs. Block (**D**) associates the enriched pathways and GOBP terms to the miRNA-target genes. The networks are also getting built in this block. The yellow color flow shows the network building flow and miRNA-mRNA-Pathway/GOBP association. The figure numbers in the dotted circles indicates the analysis outcome of that part and refer to that particular figure for the result. Figure 1B shows how the functional annotation of the miRNAs (pathway/GOBP association) in the enriched differentially expressed target genes is done.

**Figure 2:**
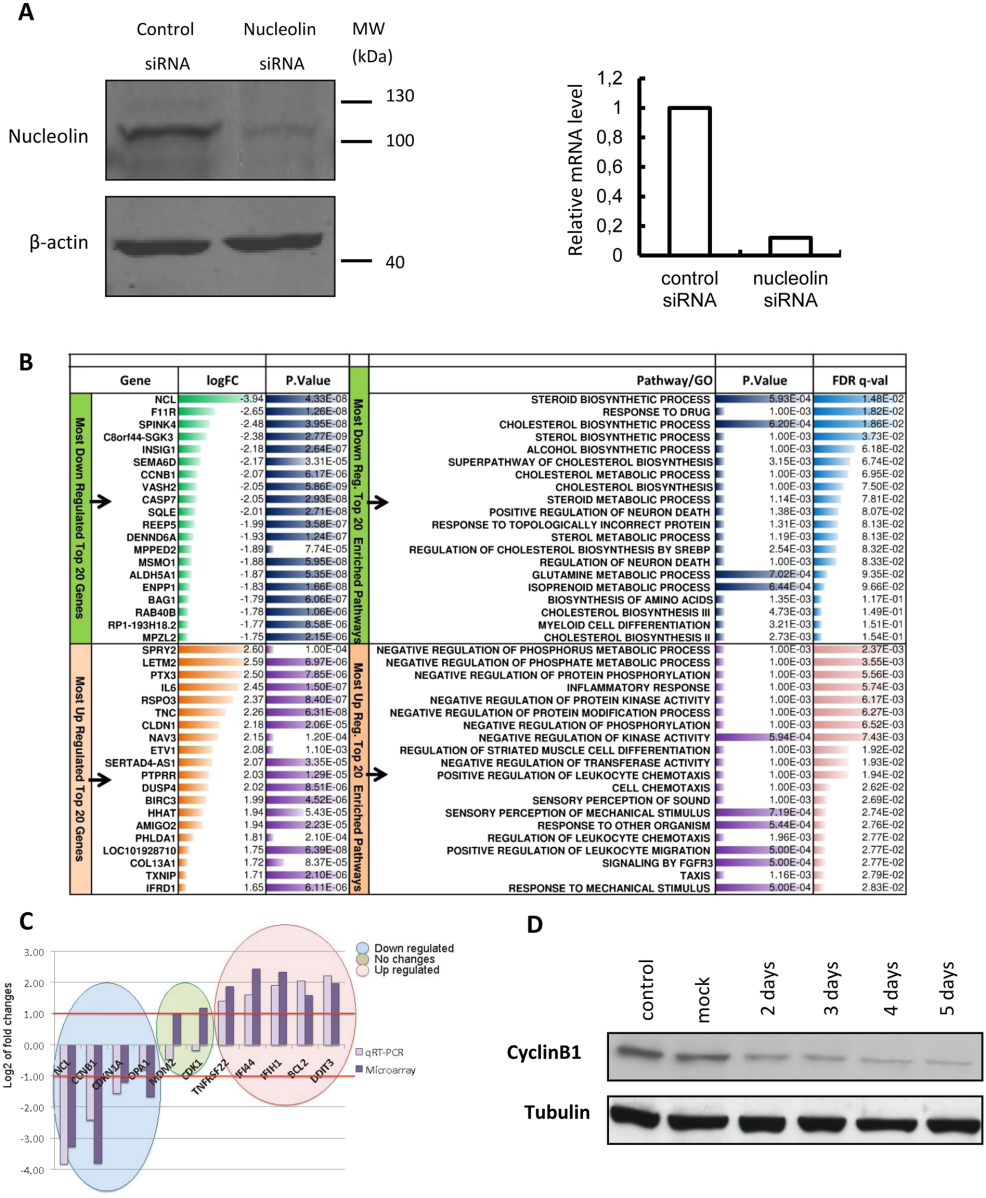
Transcriptomic analysis of nucleolin depleted cells. (**A**) HeLa cells were treated with control or NCL siRNA as previously described^27, 28^ and after 5 days, protein extracts and total RNA were prepared. NCL protein expression was assessed by western blot and normalized by comparison with β-actin (left panel) while NCL mRNA level was measured by RT-qPCR (right panel). (**B**) Lists of the 20 most down or up- regulated genes and their fold change (FC) and p-values with top 20 pathways and GOBP terms enriched with least FDR-q value. (**C**) RT-qPCR validation of genes that were found either up-, down- or unchanged in the microarray analysis. NCL was included in the analysis, together with some genes involved in cell cycle regulation and other randomly chosen genes. (**D**) Western blot analysis with total extract of the expression of CCNB1 protein upon the depletion of NCL. As CCNB1 was strongly down-regulated and may be important for the arrest of HeLa cells in G2/M phase upon NCL depletion, we choose this protein to check for the correspondence between RNA level (Affymetrix microarrays-RT-qPCR) and protein levels.

To gain a more global view on NCL function in the cell, a transcriptomic analysis of HeLa cells that have been depleted in NCL was performed. The bioinformatic analysis flow (Fig. 1) described in the methods section was followed. RNA samples were prepared in triplicates from wild type and NCL-depleted HeLa cells. As previously described, about 80 to 90% of the NCL protein and RNA was depleted upon transfection with specific NCL siRNA (Fig. 2A) and this resulted in an arrest of cell proliferation^27^. Total RNA was analyzed on Affymetrix Human genome U133 plus 2.0 microarrays to detect changes in gene expression (Supplementary Table 1).

Genes having an expression fold change of ≥1.5 and a detection Benjamini Hochberg adjusted p-value less than 0.05 were selected for further analysis. With these criteria, we found 925 and 764 genes that were down- and up-regulated respectively upon NCL depletion (Supplementary Table 2.1). The list of the 20 most down or up-regulated genes with p-values is shown in Fig. 2B. To validate this microarray analysis, we performed RT-qPCR on a selection of 11 genes. As expected, NCL is the strongest down regulated mRNA in the NCL-siRNA treated cells compared to controls both in microarray data and in the RT-qPCR assay (Fig. 2C). The RT-qPCR assay reproduced the microarray data with accuracy for the other randomly chosen genes (Fig. 2C).

### Genes involved in cell cycle regulation are deregulated upon NCL depletion

Our previous work showed that depletion of NCL in HeLa cells has a profound effect on the cell cycle, and cells rapidly accumulates in G2/M phase^27^. In addition, an accumulation of immature centrosomal structures is observed which is accompanied with multipolar centrosomal structures. Recent work also described a function of NCL in microtubule anchorage at centrosome and in microtubules dynamics^28, 29^.

To try to understand how NCL expression can affect cell cycle progression, we searched for genes coding for cell cycle proteins in the list of deregulated genes. One of the strongest repressed gene in cells expressing low amount of NCL is CCNB1 coding for the G2/M specific cyclin B1 (Fig. 2B). This down regulation of CCNB1 mRNA level was also confirmed by RT q-PCR (Fig. 2C) and western blot analysis of protein samples prepared from control and siRNA treated cells also confirm the depletion of CCNB1 protein (Fig. 2D) upon NCL depletion. This down regulation of CCNB1 together with the up-regulation of CCNB1IP1 (Supplementary Table 2.1; FC + 1.60, an E3 Ubiquitin Protein Ligase that modulates cyclin B protein levels is well correlated with the accumulation of NCL depleted cells in G2/M. Other cyclins (CCNE2, CCNG1 CCNG2) are also down regulated in these cells.

Interestingly, many mRNAs coding for proteins that participates in centrosome maturation and organization of mitotic spindle (CEP70, CEP68, CEP120, TUBGCP5, DYNC1I2) are also down regulated after NCL depletion. The down regulation of TUBGPCP5, a member of the Gamma-tubulin complex which is necessary for microtubule nucleation at the centrosome^30^ may explain why in NCL depleted cells the dynamics of microtubules nucleation at centrosome is slowed down and the number of centrosomal microtubules per cell is greatly reduced^28^. In addition, the down-regulation of major factors involved in centrosome maturation could explain why amplified centrioles remains immature in silenced NCL cells^28^. Furthermore, down regulation of the motor DYNC1I2, would suggest that intracellular retrograde mobility of vesicles and organelles along microtubules is reduced in silenced cells.

### Cholesterol and fatty acids metabolic genes expression are strongly deregulated upon NCL depletion

**Figure 3:**
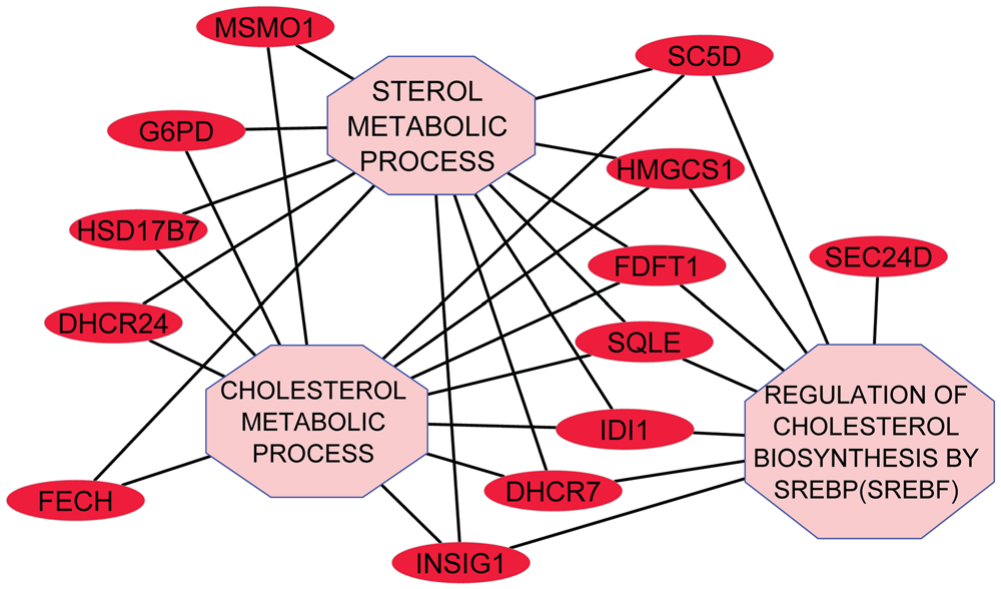
Network of down regulated genes involved in several aspects of cholesterol/sterol metabolism.

Despite these few cell cycle genes that are deregulated upon NCL depletion, Gene Ontology (GO) terms enrichment analysis for down regulated mRNAs do not identify a significant enrichment in biological processes and pathways GO terms involved in cell cycle regulation. This analysis rather highlights several processes implicated in metabolism (Fig. 2B and Supplementary Fig. 1) and in particular in sterol, phospholipids and amino acids biosynthesis (GOBP/Pathway terms with FDR < = 0.25 and p-value < = 0.05, supplementary Table 2.2). The clustering of the down-regulated genes together with the enriched pathways and GO Biological process (BP) terms illustrates the strong enrichment of down regulated genes coding for enzymes involved in the cholesterol biosynthetic process (Fig. 3) and in amino-acids synthesis and apoptotic processes (Supplementary Fig. 1).

**Figure 4:**
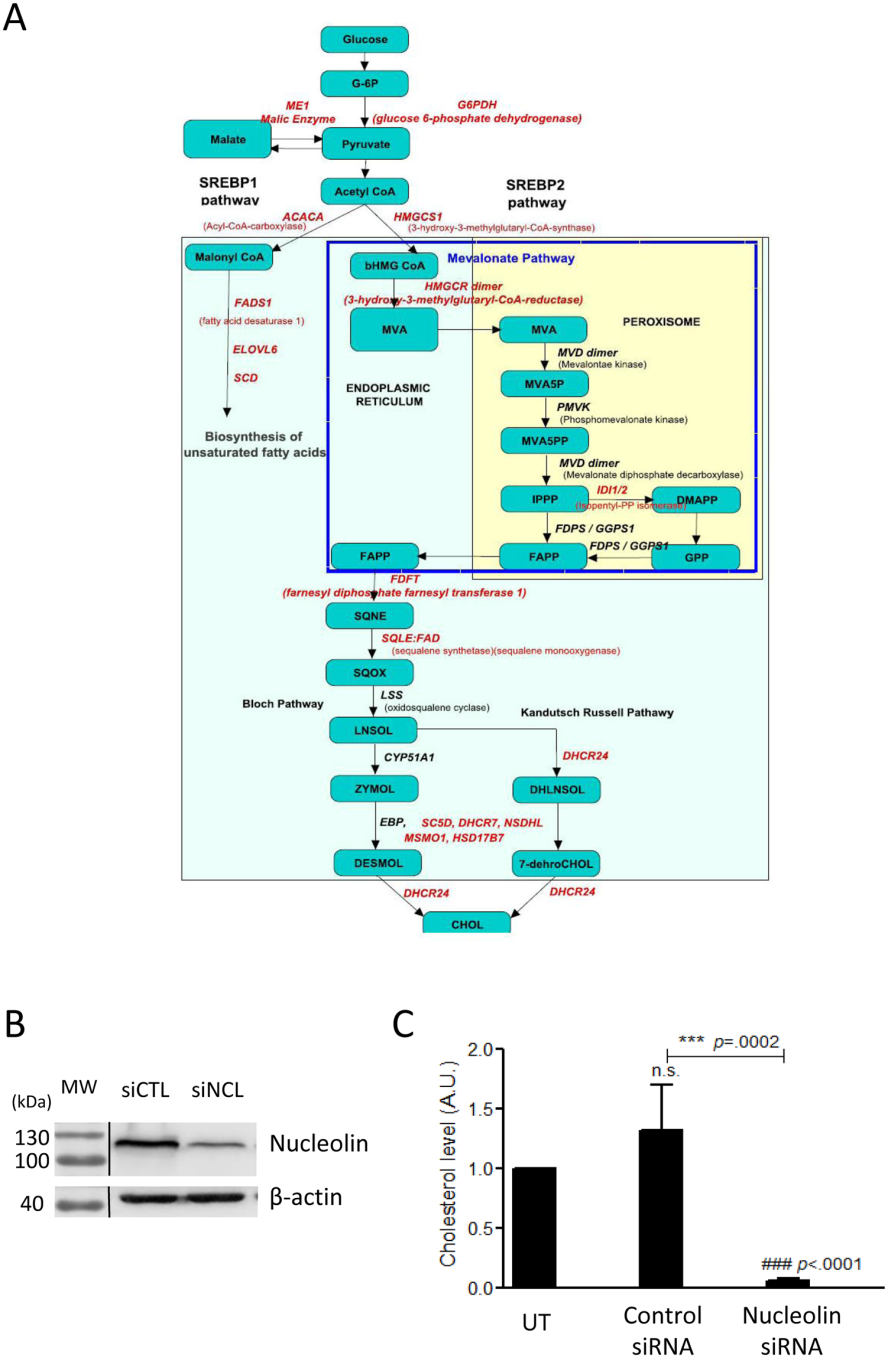
NCL expression affects cholesterol synthesis. (**A**) The different enzymes of the cholesterol biosynthetic pathways that are down regulated in the NCL siRNA treated cells are indicated in red on the scheme of the cholesterol synthesis pathway. (**B**) Western blot to detect nucleolin and β actin in cells transfected either with control siRNA or siRNA targeting nucleolin. Total extracts were used and loaded onto a 10% SDS-PAGE. (**C**) Cholesterol dosage from untransfected (UT), control siRNA or nucleolin siRNA treated cells. Assays were performed in triplicates for each condition, and samples were processed as recommended by the manufacturer. Results were expressed compared to untransfected cells.

As many enzymes involved in the synthesis of cholesterol (HMGCS1; HMGCR; IdI1; FDFT1; SQLE; CYP51A1; SC5D; DHCR; HSD17B7, see Fig. 4A) are down regulated, we experimentally determined if this influenced cholesterol accumulation (Fig. 4C). Triplicates of untransfected cells (UT), cells transfected with control siRNA or NCL siRNA were used for cholesterol level measurements (Fig. 4C). Upon NCL depletion (Fig. 4B), we observed a strong decrease of cholesterol levels compared to untransfected cells or cells transfected with control siRNA (Fig. 4C). Therefore, the expression of NCL that is required for the expression of enzymes involved in the synthesis of cholesterol in HeLa cells results indeed in lower level of total cholesterol in the cells.

**Figure 5:**
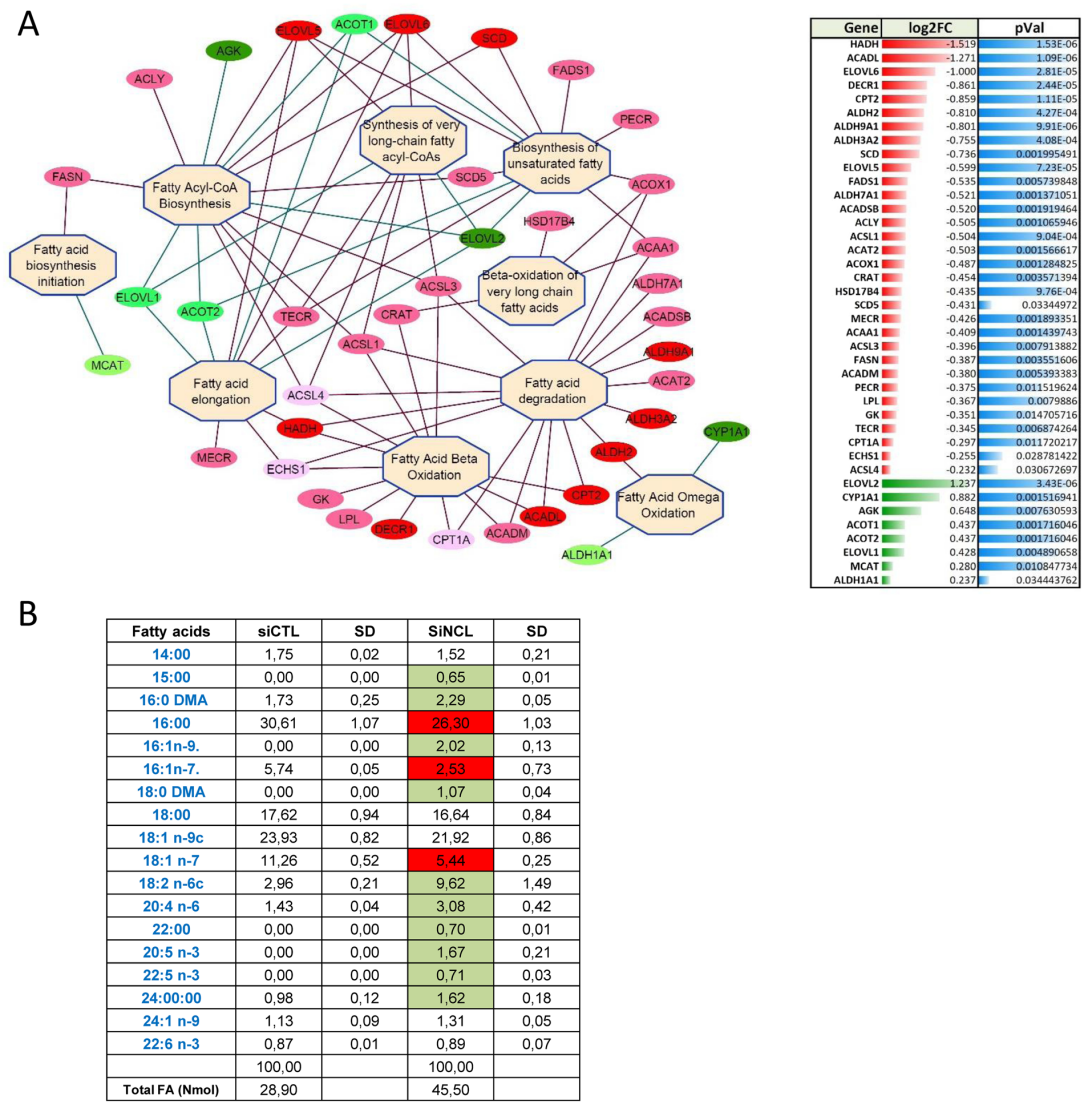
NCL expression affects fatty acids synthesis and accumulation. (**A**) Network of the genes involved in fatty acids biosynthesis, beta-oxidation or degradation that are significantly deregulated in nucleolin depleted cells. In red genes that are down-regulated (FC > 1.5), pink (1.3 < FC > 1.5) and in green genes that are upregulated. (**B**) Fatty acids analysis. Samples from siRNA control (siCTL) and siRNA nucleolin (siNCL) were processed as indicated in the materials and methods. Highlighted in red the fatty acids that are down regulated and in green the fatty acids that accumulate in NCL depleted cells. The data are presented for each fatty acids as a percentage compared to the total fatty acids. The amount of fatty acids in NCL depleted cells is higher (45.5) compared to control cells (28.9).

Several genes involved in fatty acids (FA) metabolism were also down regulated (FC > 1.5 and p-value < 0.05; FADS1, ELOVL6, SCD). To further explore the possibility that NCL expression is required for FA synthesis, we found in our transcriptomic analysis additional genes that were down regulated (with p < 0.05 and 1.5 > FC > 1.3) like for example ALDH7A1, ACADSB, ACLY, ACSL1, ACAT2, ACOX1, CRAT, HSD17B4, MECR, ACAA1, ACSL3, FASN). These genes are directly involved in different steps of FA metabolism (Fig. 5A) and in particular in fatty acyl-CoA biosynthesis, and degradation.

The cytosolic fatty acid synthase (FASN), an important enzyme that catalyzes the formation of C16:0 palmitic acid from acetyl-CoA, malonyl-CoA and NADPH is slightly down regulated, while the elongase enzymes ELOVL5 and 6 which control the first, rate limiting condensation steps of very long chain fatty acid (VLCFA) elongation^31^ are strongly down-regulated. This lead us to determine how the fatty acids synthesis was affected in NCL depleted cells. We performed a lipidomic studies of FA by gas chromatography in cells transfected with control or NCL siRNA (Fig. 5B). Globally a significant increased of FA quantity is observed in NCL depleted cells (45.5 vs 28.9 Nmol). Indeed, compared to control cells, all FA acids are increased except for the unsaturated C16:1n-7 and C18:1n-7. C16:1n-7 is probably formed from the pathway involving the Stearoyl-CoA desaturase (SCD) that is strongly down-regulated in our study while the global increased of saturated FA and unsaturated n-3 and n-6 are likely the consequences of the upregulation of the elongase ELOV1and ELOV2. The strong down-regulation of many of the enzymes involved in FA beta-oxidation or degradation (Fig. 5A) results in a global increased of FA in NCL depleted cells.

**Figure 6:**
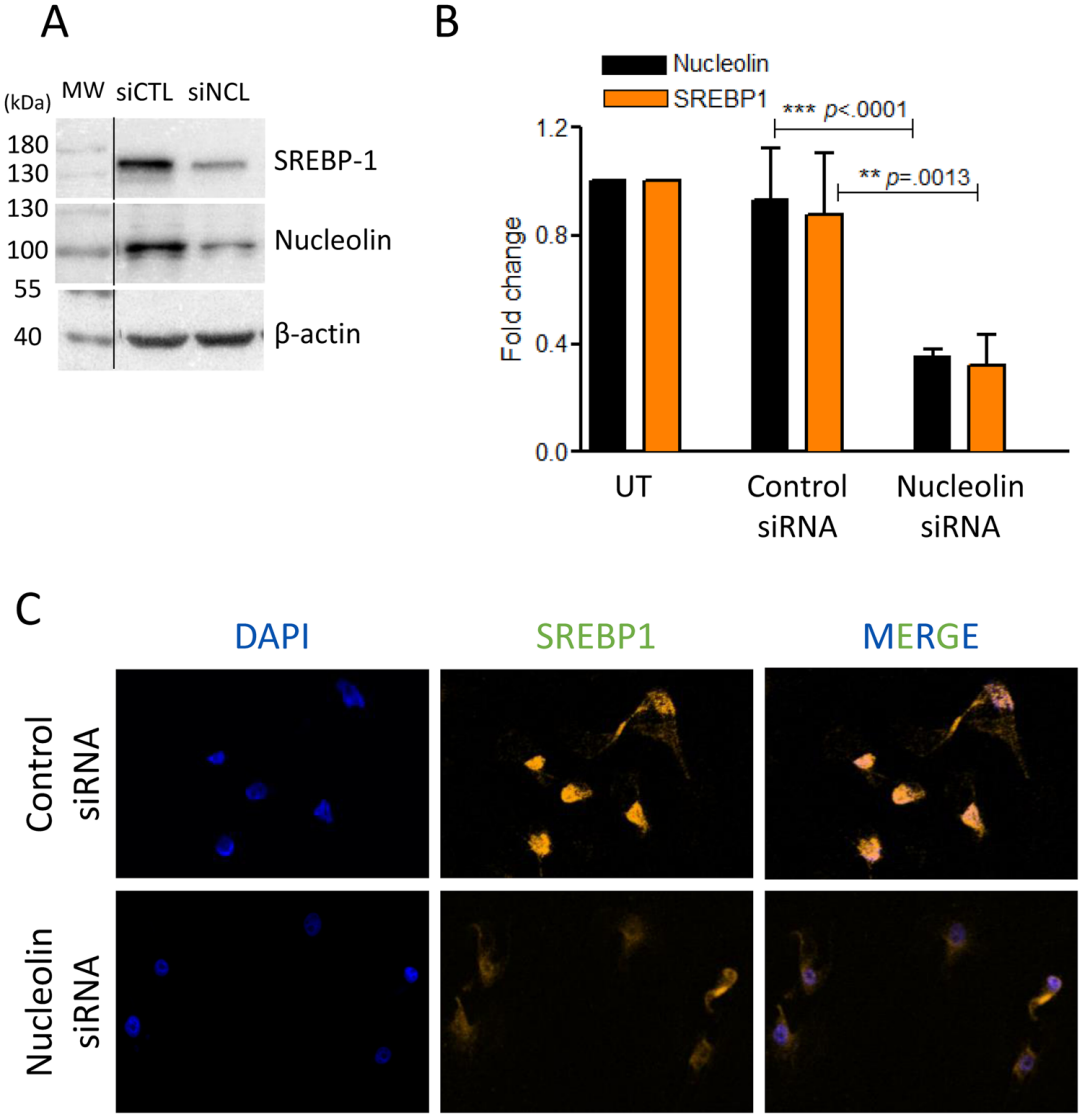
NCL expression affects SREBP1 accumulation and localization. (**A**) The level of SREBP1 protein was determined by western blot in cells transfected with control siRNA or with siRNA for NCL. Total extract were used and loaded onto a 10% SDS-PAGE. (**B**) Quantification of the expression of NCL and SREBP1 in untransfected cells (UT), and in cells transfected with control or NCL siRNA. Quantifications represent an average of 3 independent experiments. (**C**) Immuno-localization of SREBP1 in cells transfected with control or NCL siRNA. 96 h after transfection, cells were process for immunofluorescence with anti-SREBP1 antibody and with DAPI. Cells were then observed with confocal microscope (Zeiss LSM510).

The transcription factor SREBP1 is involved in the regulation of many of these enzymes that are found down-regulated in our study. As the expression of SREBP1 mRNA is also down-regulated (FC -1.6; Supplementary Table 1) we determined if the level of SREBP1 protein was also down-regulated in cells depleted in NCL. Indeed, western blot analysis (Fig. 6A,B) indicated a good correlation between the decreased level of expression observed in the transcriptomic data and the level of accumulation of the SREBP1 protein. The nuclear transcriptional activity of SREB1 is also regulated by its translocation from the membrane of the endoplasmic reticulum to the cell nucleus. Therefore, we determined by immunofluorescence the localization of SREBP1 in controls and NCL depleted cells (Fig. 6C). In control cells, a strong SREBP1 signal is found in the nucleus. However, upon the depletion of NCL, we not only observed a much weaker signal for SREBP1 but also that most of the signal seems to remain in the cytoplasmic compartment. The expression of the mRNA coding for the SREBP Cleavage Activating Protein (SCAP) involved in the release of the nuclear form of SREBP is also only slightly affected in NCL depleted cells (FC: -1.3). Therefore, both the expression and localization of SREBP is affected in NCL depleted, and could explain the effect on lipid metabolism.

Altogether, this analysis indicates that the normal expression of NCL is required for these lipids metabolic pathways. This finding might be key to understand the requirement of NCL for cell proliferation, and to explain the over-expression of NCL in many cancers. Indeed, an increased metabolic activity is observed in cancer cells to sustain the high proliferation rate of these cells. For example, in pancreatic ductal adenocarcinoma (PDAC) tumors, that express high levels of NCL^9^, the oncogenic KRAS mutation is crucial to reprogram cellular metabolism to sustain unrestricted tumor growth^32^. In particular, it was shown that KRAS was able to reprogram glutamine metabolism by the activation of the GOT1 enzyme (cytosolic aspartate aminotransferase 1) producing oxaloacetate used for the production of NADPH which is essential to maintain redox homeostasis and support tumor growth. Interestingly, NCL expression is essential for the expression of ME1 (malic enzyme 1) (Fig. 4), G6PD and PGDH which are key enzyme in the NADPH production pathway. In addition, in PDAC, increased glycolysis is achieved through the transcriptional up-regulation of many enzymes involved in these pathways and through the reprogramming of metabolic intermediates of glycolysis in the pentose phosphate pathways (PPP). Our data show that expression of NCL is also important for the expression of many enzymes involved in glycolysis and in the PPP pathways such as Phosphofrutokinase and the glucose-6-phosphate dehydrogenase (G6PD). It would be interesting to determine if the recent finding^9^ that the targeting of NCL with an aptamer in PDAC which result in tumor growth reduction is linked to the inhibition of expression of the metabolic genes identified in this study. In addition, our data agree and may provide a molecular mechanism with a recent observation that NCL targeting in retinoblastoma result in global lipid reduction^33^.

These data suggest that the over-expression of NCL protein in cancer cells probably participates in the increased metabolism required for the proliferation of tumor cells.

### Many genes involved in signaling pathways are up-regulated upon NCL depletion

Gene ontology and pathways analysis of the 764 up-regulated genes (Supplementary Table 2.3) show an enrichment of the biological process terms involved in chemotaxis, migration and the negative regulation of protein phosphorylation and of intracellular signal transduction (Fig. 2B, Supplementary Table 2.3).

Indeed, cell shape is drastically affected when NCL expression is reduced^27–29^. Many studies have shown that the expression of NCL is required for cell migration or adhesion^34–37^. In most of these studies, NCL silencing using siRNA reduced cell migration and invasion. The molecular mechanism involving NCL is not well defined, therefore the identification in this study of genes that participate in chemotaxis and cell migration, and that are deregulated upon NCL depletion may help to understand the implication of NCL in these processes.

GO and pathways analysis indicate a strong enrichment of genes involved in different signaling pathways: TGF beta, FoxO, NF-kappa B, wnt, TNF, TWEAK,etc (Supplementary Tables 2.1 and 2.3 with FDR <  = 0.25 and p-value <  = 0.05 enrichment using GSEA Pre-ranked). Indeed, amongst the major upregulated genes upon NCL depletion (Fig. 2B) are genes coding for regulators of MAPK like SPRY2, DUSP4 and DUSP6, PTPRR, CLDN1. SPRY2 is a negative regulator of MAPK activation induced by several stimuli including EGF, FGF, vEGF, PDGF, NGF, GDNF. The DUSP4 and DUSP6 proteins are phosphatases that negatively regulate MAPK (ERK, JNK, p38) associated with cell proliferation and differentiation while PTPRR is a protein tyrosine phosphatase that can bind and inactivate MAPK. CLDN1 (Claudin), a membrane bound protein involved in the paracellular transport process within the tight junction is regulated at the expression level by phosphorylation by kinases like PKC, EGK, MAPK. Hhat is a transmembrane enzyme that mediates the palmitoylation of Sonic Hedgehog (Shh) a modification that is critical for signaling activity, TNC (Tenascin) involved in the PI3K-Akt and ERK signaling pathways, RSPO3 (R-spondin 3) a secreted protein that activates Wnt/beta-catenin signaling and PTX3 (Pentraxin) that down regulates FGF2 are also upregulated in NCL depleted cells.

This analysis shows that the lower expression of NCL in siRNA treated cells induces the activation of many genes involved in the down regulation of signaling pathways implicated in cell proliferation and cell migration which contribute to the inhibition of cell proliferation observed in these NCL depleted cells.

### miRNA expression analysis in HeLa cells upon NCL depletion

**Figure 7:**
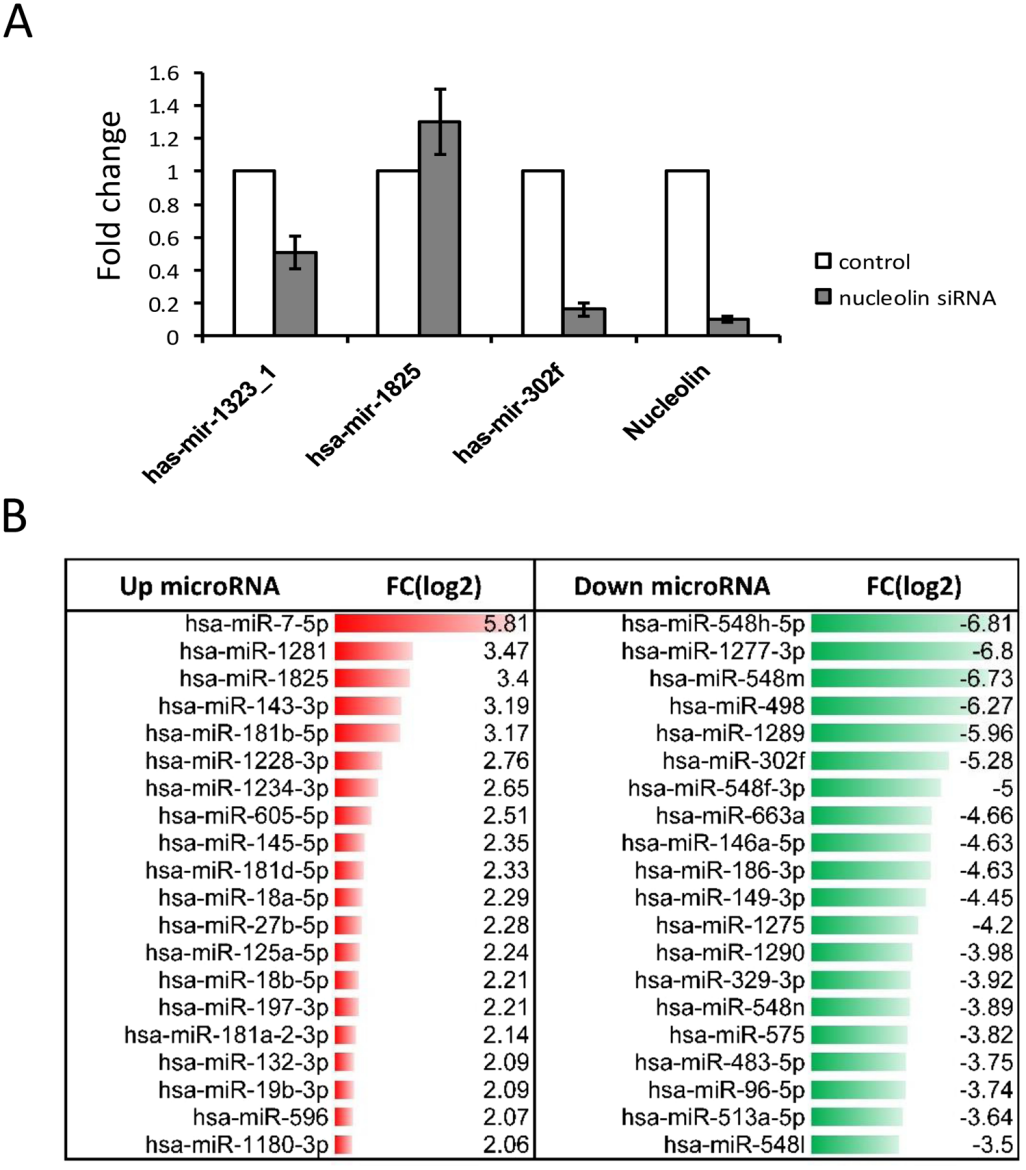
miRNAs expression analysis in NCL depleted HeLa cells. (**A**) Fold change of randomly selected miRNAs (Upregulated Hsa-miR-1825 and down regulated hsa-miR-1323 -hsa-miR-302f and NCL) as determined by RT-qPCR. (**B**) First 20 differentially expressed (Up/Down) microRNAs with FC ≥  ± 1.5 and p-value ≤ 0.05.

As previous studies have pointed out to a function of NCL in miRNA synthesis^23, 24^ we analyzed the effects of NCL depletion in HeLa cells on the steady state level of microRNAs. Triplicates RNA samples extracted from control or NCL-depleted HeLa cells were analyzed on Human microarrays (MRA-1001 LC Sciences). By applying a filter for miRNAs having a fold change expression of ≥1.5 and a detection P-value less than 0.05 we found that 77 miRNAs were up regulated and 48 were down regulated (Fig. 7B and Supplementary Table 4). Up-regulated Hsa-miR-1825 and down regulated hsa-miR-1323 -hsa-miR-302f were randomly selected for RT-qPCR validation of the micro arrays data (Fig. 7A).

Several studies have implicated NCL in miRNA synthesis in cancer cells. For instance, the expression of miR-21, mir-221, miR-222 and miR-103 which are involved in breast cancer initiation and progression is regulated by NCL^24^. In these cancer cells, inhibition of NCL expression leads to a decreased level of these miRNAs which is associated with a reduced breast cancer cell aggressiveness. In another study, the maturation of mir-15a and mir-16 was shown to be dependent of NCL^23^. All these miRNAs are also found deregulated in our study using HeLa cells, indicating that the regulation of these miRNAs by NCL is not restricted to breast cancer cells as described in these studies.

### Correlation of differentially expressed miRNA-mRNA (target genes), enrichment and functional annotations

**Figure 8:**
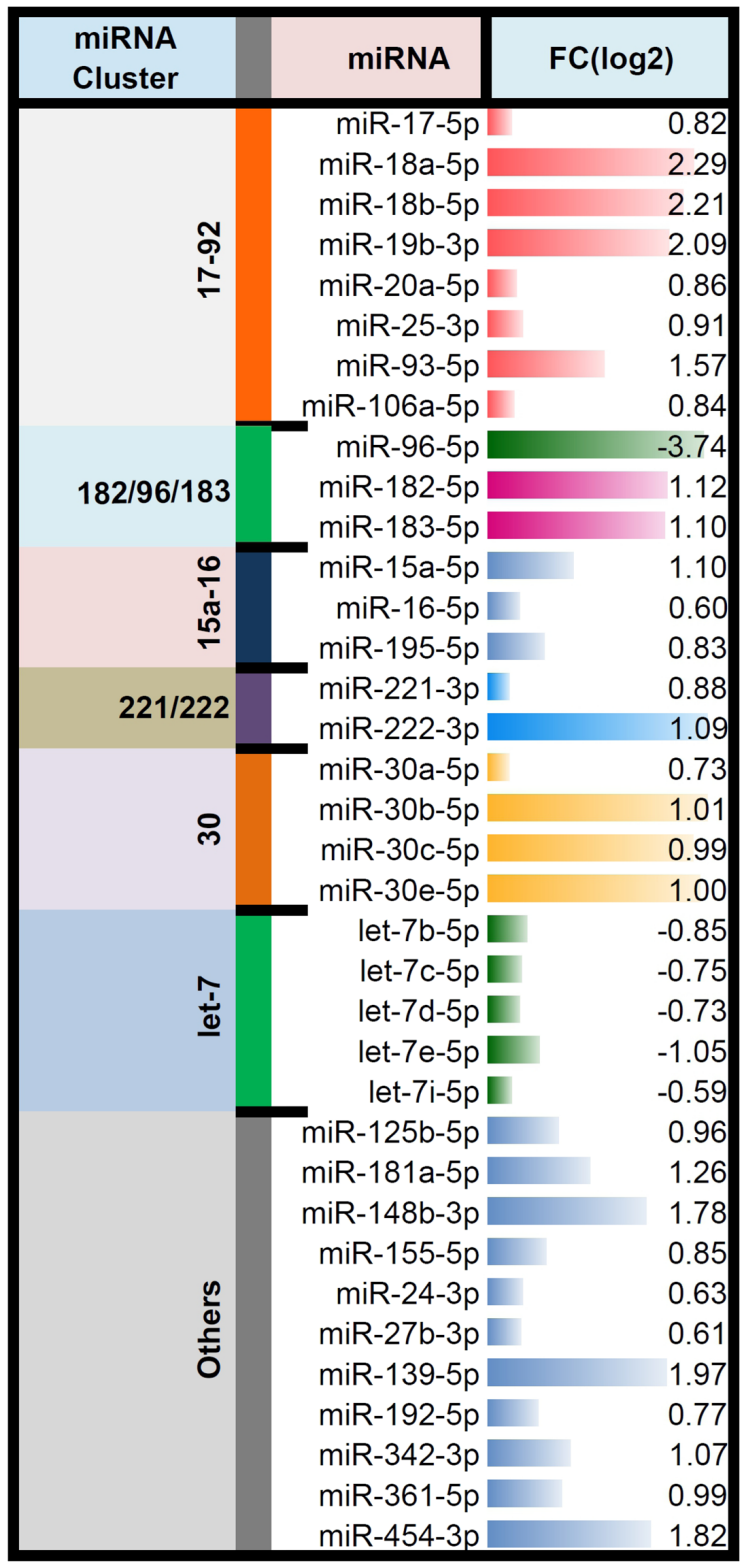
The miRNA clusters with their Fold Change (log2) observed in the enriched miRNAs.

Several differentially regulated miRNAs found in our study have been already reported to be functionally associated with NCL. To understand and annotate the functional aspects (the pathways and the biological processes) which might be affected by these differentially expressed miRNAs (Supplementary Table 4) by targeting the differentially expressed mRNAs (Supplementary Table 2.1), we performed an analysis using GSEA tool^35^ and a statistical enrichment analysis based on weighted Kolmogorov-Smirnov (KS) test. The bioinformatics analysis steps with KS-test from GSEA are shown in Fig. 1 (blocks C1 and C2) and detailed in the methods section. KS-test is used to determine whether two data sets differ significantly in their distribution. In our case, using the miRNA-target genes (Supplementary Table 6) and the differentially expressed miRNA (Supplementary Table 4) this analysis will allow us to find out the enriched miRNAs and the corresponding enriched target genes from the differentially expressed mRNAs by pre-ranking with the fold change of these differentially expressed miRNAs. For mathematical details of the KS statistics which is a distribution independent and non-parametric test, refer to Hollander, M.and Wolfe, DA^38^. The various specific clusters of these enriched miRNAs along with their fold change associated with the differentially expressed genes is reported (Fig. 8).

The 5 down and 30 up regulated miRNAs and the corresponding 52 down and 43 up (Supplementary Table 4) regulated target genes with the statistically significance FDR < 0.25 and p-value < 0.05 from GSEA^35^ got enriched. Many of these enriched miRNAs and associated targets differentially expressed genes are also reported to be regulated by these miRNAs in the literature and in an experimentally validated database (Tarbase)^39^. In our case, 42 out of 52 (80%) of down regulated and 40 out of 43 (93%) of up regulated genes are matching with these already reported literature and Tarbase data (Supplementary Tables 7.1. and 7.2).

The biological network based analysis (Fig. 1B) of these pathways and GO Biological Processes which might be getting affected by these differentially regulated miRNAs points out to their involvement in regulation of the various pathways like apoptosis, proliferation, signaling, chemotaxis, cell motility, cholesterol synthesis, cell migration, and cell differentiation (Supplementary Fig. 2 and Table 1).

**Table 1:** Pathways and their categories of the miRNA target enriched genes and the pathways effected by these miRNAs.

Apoptosis	REGULATION OF NEURON APOPTOTIC PROCESS
	CELL-TYPE SPECIFIC APOPTOTIC PROCESS
	REGULATION OF EXTRINSIC APOPTOTIC SIGNALING PATHWAY IN ABSENCE OF LIGAND
	POSITIVE REGULATION OF NEURON APOPTOTIC PROCESS
Proliferation	REGULATION OF STEM CELL PROLIFERATION
	REGULATION OF LYMPHOCYTE PROLIFERATION
	POSITIVE REGULATION OF EPITHELIAL CELL PROLIFERATION
	POSITIVE REGULATION OF STEM CELL PROLIFERATION
	REGULATION OF LEUKOCYTE PROLIFERATION
	REGULATION OF EPITHELIAL CELL PROLIFERATION
	EPITHELIAL CELL PROLIFERATION
	REGULATION OF MONONUCLEAR CELL PROLIFERATION
Signaling	SECOND-MESSENGER-MEDIATED SIGNALING
	SIGNALING BY PDGF
	NOTCH SIGNALING PATHWAY
	SIGNAL TRANSDUCTION BY PHOSPHORYLATION
	SYNDECAN-4-MEDIATED SIGNALING EVENTS
	SIGNALING BY FGFR3
	ERBB SIGNALING PATHWAY
	INTRACELLULAR RECEPTOR SIGNALING PATHWAY
	TGF-BETA SIGNALING PATHWAY
	PI3K-AKT SIGNALING PATHWAY
	REGULATION OF PROTEIN KINASE B SIGNALING
	IMMUNE RESPONSE-ACTIVATING SIGNAL TRANSDUCTION
	SIGNALING BY WNT
	SIGNALING PATHWAYS IN GLIOBLASTOMA
	POSITIVE REGULATION OF PROTEIN KINASE B SIGNALING
	AGE-RAGE SIGNALING PATHWAY IN DIABETIC COMPLICATIONS
	NEGATIVE REGULATION OF INTRACELLULAR SIGNAL TRANSDUCTION
	REGULATION OF NUCLEAR BETA CATENIN SIGNALING AND TARGET GENE TRANSCRIPTION
Chemotaxis	REGULATION OF GRANULOCYTE CHEMOTAXIS
	POSITIVE REGULATION OF CHEMOTAXIS
	POSITIVE REGULATION OF POSITIVE CHEMOTAXIS
	POSITIVE REGULATION OF LEUKOCYTE CHEMOTAXIS
	REGULATION OF CHEMOTAXIS
	REGULATION OF POSITIVE CHEMOTAXIS
	REGULATION OF LEUKOCYTE CHEMOTAXIS
Cancer	APOPTOSIS-RELATED NETWORK DUE TO ALTERED NOTCH3 IN OVARIAN CANCER
	INTEGRATED BREAST CANCER PATHWAY
	MICRORNAS IN CANCER
	TRANSCRIPTIONAL MISREGULATION IN CANCER
Mot	POSITIVE REGULATION OF CELL MOTILITY
Chol Syn	CHOLESTEROL METABOLIC PROCESS
	CHOLESTEROL BIOSYNTHETIC PROCESS
	REGULATION OF CHOLESTEROL BIOSYNTHESIS BY SREBP
Cell Migr	REGULATION OF EPITHELIAL CELL MIGRATION
	POSITIVE REGULATION OF LEUKOCYTE MIGRATION
	POSITIVE REGULATION OF EPITHELIAL CELL MIGRATION
	REGULATION OF LEUKOCYTE MIGRATION
Cell Diff	NEGATIVE REGULATION OF MUSCLE CELL DIFFERENTIATION
	REGULATION OF STRIATED MUSCLE CELL DIFFERENTIATION
	REGULATION OF MYOTUBE DIFFERENTIATION
	EPITHELIAL CELL DIFFERENTIATION
	NUCLEOTIDE-BINDING DOMAIN LEUCINE RICH REPEAT CONTAINING RECEPTOR

Interestingly, several miRNAs whose expression is modified in NCL depleted cells have been shown experimentally to be associated with the regulation of lipid metabolism^40–42^.

**Figure 9:**
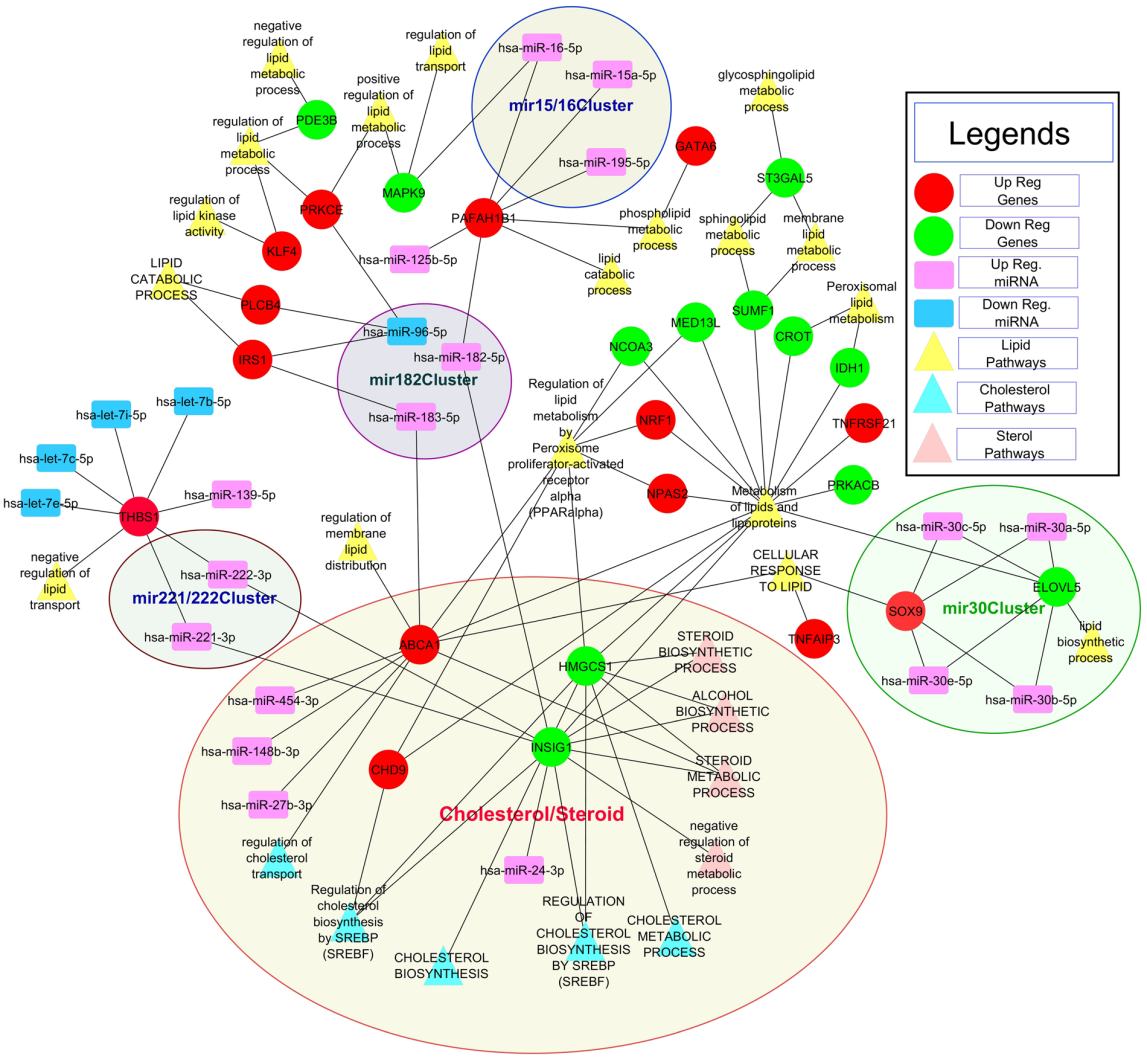
Network of the Differentially expressed and KS statistics based enriched mRNA-miRNA functional interaction and the cholesterol/lipid pathways associations” (Also refer to corresponding Supplementary Figs S1A, S2A and S2B and related network in S3C).

Differentially expressed and KS statistics based enriched mRNA-miRNA were used to build a network (Supplementary Fig. 3) and in particular showing the link between these miRNA-mRNA and cholesterol/lipid pathways/GOBP terms associations (Fig. 9). In particular, miR 17–92, miR 182-183-96 cluster show a clear link with lipid metabolism (red and green edges in Fig. 9 and Supplementary Fig. 3C). miR-182 which is up regulated in NCL depleted cells, has been shown experimentally to negatively regulate the expression of Fbxw7^40^. This protein is known to affect nuclear SREBP accumulation and may contribute to the low level of SREBP that is observed in NCL depleted cells (Fig. 6C). Therefore, the up-regulation of miR-182 could be part of the mechanism that participate to the down regulation of the expression of many genes involved in cholesterol and phospholipid metabolism (Figs 3 and 4), as the expression of these genes is dependent on the transcription factor SREBP.

NCL protein is essential for cell proliferation and division^26, 27^, and its expression level is increased in many cancer types^5^. Interestingly, several strategies have been developed to target NCL in cancer cells to block cancer cells progression. Indeed, nucleotidic and peptidic aptamers that target NCL seem to be efficient molecules to inhibit tumor growth of several types^33, 43–45^. By binding to NCL, these aptamers are believed to inhibit NCL cellular functions which remain to be determined. It would be interesting to know if the targeting of NCL with these aptamers affect the same pathways as the down regulation of NCL expression by siRNA. If this is the case, this may give some indications on the mode of action of these aptamers. In addition, some of the genes that have been identified in this study could be used as biomarkers to follow the efficiency of NCL targeting in cancer cells or as additional targets for combotherapy targeting NCL and other pathways. This transcriptomic study paves the way to analyze more globally the cellular function of NCL.

## Materials and Methods

### Cell culture and transfection

HeLa cells were grown in *α*MEM medium containing Glutamax (PAA), complemented with 10% Fetal Calf Serum (FCS), 1% non essential amino acids, and 1% penicillin/streptomycin. Cells were maintained at 37 °C in a 5% CO_2_-humidified incubator.

A mixture of functional siRNAs (Eurogentec) specific for human nucleolin was used as previously described^27, 28^. siRNAs (siRNA #4 (UUCUUUGACAGGCUCUUCCUU) and siRNA #2 (UCCAAGGUAACUUUAUUUCUU) were reconstituted at a concentration of 100 nM and stored at −20 °C. As a siRNA control, we used stealth high GC siRNA (Invitrogen). Cells were transfected in a 6-well dish using siRNA at 2 nM final concentration. siRNAs were diluted in 200 *μ*l of Opti-MEM and plated in a well. 80 *μ*l of INTERFERin (Polyplus) diluted 1:10 in RNase-free water were added. After 10 min incubation, 2 ml of medium containing 3 × 10^5^ cells were added. RNA extraction, and protein analysis were performed 5 days after the initial transfection. For immunofluorescence staining, SiRNA-transfected HeLa cells were seeded onto cover slips in 24-well plate (BD Falcon) for 48 h. The next day, cells were rinsed with cold PBS and fixed with 4% paraformaldehyde for 10 min at room temperature followed by permeabilization with 0.1% Triton X-100. The cells were subjected to immunofluorescence staining with SREBP1 antibody (1:200) (Covalab) for 2 h at room temperature. Washed with cold PBS three times for 5 min each, cells were incubated with Alexa 555-labeled anti-rabbit secondary antibody (1:500) (BD Bioscience) at room temperature for 1 h and then washed with cold PBS three times for 5 min each. Cell nuclei were counterstained with DAPI and cover slips were rinsed with distilled water and mounted using fluorescent mounting medium (Invitrogen). Cells were visualized under a confocal microscope (Zeiss LSM510).

### RNA extraction and Real Time-PCR

Total RNA was extracted with RNeasy kit (Qiagen, France) and treated with DNase I (Promega). After extraction, the integrity of total RNA was examined on a 1.2% agarose gel containing 1 mg/ml ethidium bromide and quantified. For qPCR analysis and validation of the microarrays data and for the nucleolin depletion, 100 ng of total RNA were reverse-transcribed using random primers and 1^st^ Strand cDNA Synthesis Kit for RT-PCR (Roche Molecular Biochemicals). Real-time PCRs were performed with a Light Cycler 2.0 instrument (Roche Molecular Diagnostics) in Light Cycler capillaries using a commercially available master mix containing Taq DNA polymerase and SYBR-Green I deoxyribonucleoside triphosphates. After the addition of primers (final concentration: 0.5 μM), MgCl_2_ (4 mM) and template DNA to the master mix, 45 cycles of denaturation (95 °C for 1 s), annealing (58 °C for 10 s) and extension (72 °C for 10 s) were performed. Cytoplasmic β-actin and18S rRNA were analyzed in parallel to each PCR, and the resulting actin and 18S rRNA measurements were used as internal standards for quantification of the specific transcripts as indicated.

### Western blot analysis

For all western blot analysis, total cell extracts were prepared and loaded onto a 10% SDS-polyacrylamide gel. Nucleolin antibody (Covalab, pab0971-P), SREBP (Covalab, Mab50754), and beta-actin (Sigma A-5441) were used.

### Lipids extraction and analyses

For fatty acids analysis total lipids were extracted from cells transfected with control or nucleolin siRNA with ethanol/chloroform (1:2, v/v)^46^. Before extraction, internal standard (1,2-diheptadecanoyl-sn-gycero-3-phosphocholine (GPC di-17:0) was added. The organic phases were dried under nitrogen and the phospholipids were separated from neutral lipids by thin-layer chromatography using the solvent mixture hexane-ethyl ether–acetic acid (80:20:1 v/v/v) as eluent. Phospholipid spot on silica gel was scrapped and further transmethylated and the fatty acid methylesters were analyzed by gas chromatography. Briefly, samples were treated with toluene-methanol (1:1, v/v) and boron trifluoride in methanol^46^. Transmethylation was carried out at 100 °C for 90 min. After addition of 1.5 mL K_2_CO_3_ in 10% water, the resulting fatty acid methyl esters were extracted by 2 mL of isooctane and analyzed by gas chromatography with a HP6890 instrument equiped with a fused silica capillary BPX70 SGE column (60 × 0.25 mm). The vector gas was hydrogen. Temperatures of the Ross injector and the flame ionization detector were set at 230 °C and 250 °C, respectively.

The concentration of cholesterol was measured using the Total Cholesterol Assay Kit (Colorimetric) (CELL BIOLABS, INC STA- 384). Extraction of lipids was carried out from 10 × 10^6^ cells (untransfected, transfected with control or nucleolin siRNA). Cells were resuspended in 50 µL of PBS, then 200 μL of the extraction mixture chloroform:isopropanol:NP-40 (7:11:0.1) was added. Samples were incubated in a thermomixer at 1400 rpm for 1 h at room temperature then centrifuged for 10 minutes at 15,000 g. The liquid phase was evaporated and the residual lipid film was dissolved in 200 μL of 1X Assay Diluent. Samples were processed as recommended by the manufacturer and absorbance values were recorded at 540 nm.

### Transcriptome Data Processing and Analysis

To generate the normalized expression data and to find the differentially regulated (up/down) genes, the transcriptome data from Affymetrix Human genome U133 plus 2.0 microarray chip having three controls and three NCL depleted (siRNA) (hereafter called as treated samples) of the HeLa cells were used. The Affy package (library “affy” in R - https://www.r-project.org/) was used to read and analyze the raw (non-normalized) expression data. The Robust Multi-array Average (RMA)^47^ algorithm, part of affy R package, is used to normalize the expression data of both the control and the treated samples. By RMA, the raw expression data (intensity values) is processed in three steps - background corrected, log2 transformed and then quantile normalized. In limma^42^, using the lmFit, eBayes and topTables functions, we computed the differentially expressed up (764) and down (925) regulated genes from the RMA normalized gene expression data with Fold change greater than 1.5 and Benjamini–Hochberg (BH) controlled FDR (False Discovery Rate)^48^ corrected P <  = 0.05 (Fig. 1A- Block (A) and Supplementary Table 1 and Supplementary Table 2.1).

The pathways and biological processes which might be affected by these differentially expressed genes are analyzed using GSEA tool^35^ and the statistical enrichment analysis based on weighted Kolmogorov-Smirnov (KS) test in that tool. The KS-test is used to determine whether two data sets (one data set “Pathway/GOBP - genes”- Supplementary Tables 2.2/2.3 and the other differentially expressed mRNAs (up/down)- Supplementary Table 2.1 in our case) differ significantly in their distribution and accordingly get enriched using the pre-ranking of the differentially expressed mRNA by their fold change. The customized pathway/GOBP database used in the enrichment in GSEA (Supplementary Tables 2.2 and 2.3) was built by taking the data from various human pathway resources such as: Reactome, Kegg, Panther, WikiPathways, HumanCys, NCI Nature, Pathway Commons, and the GO BP terms from the Gene ontology consortium (http://www.geneontology.org) (Supplementary Table 5).

The first top 20 enriched pathways and GOBP terms with statistically significant lowest p-value are shown in Fig. 2B and the heatmap in Supplementary Figs 1A and 1B).

### miRNA data Processing and mRNA-miRNA integrated analysis

Using t-test (Fig. 1A, Block (B)) the differentially expressed miRNA with FC≥ +/−1.5 (Up/down regulated) and with p-value ≤ 0.05 were selected. We got 77 up regulated and 48 down regulated miRNAs (Fig. 7B and full list in Supplementary Table 4).

For these deregulated miRNA in NCL depleted cells, in order to identify potential mRNA targets, we first collected the different mRNA targets for each miRNA by a search in several databases as shown in Fig. 1A Block (C2). To avoid the false positives and having more reliable association of miRNA-mRNA genes, for each miRNA, the target genes considered were either experimentally validated or were present in at least 5 different databases (called as Hit score-5). Several hundred putative targets were identified this way (Supplementary Table 6, “miRNA -target genes” database).

The enrichment analysis and the association of the differentially expressed genes as statistically significant targets of the differentially expressed miRNAs of the Fig. 7B/Supplementary Table 4 is conducted with the Gene Set Enrichment Analysis (GSEA) software-http://software.broadinstitute.org/gsea/index.jsp (Fig. 1 Block C1). As mentioned earlier in transcriptome Data analysis section, GSEA, a computational framework, basically uses weighted Kolmogorov-Smirnov statistics to detect the statistically significant and concordant differences in a priori defined gene sets such as list of pathways or the list of miRNAs (in our case) between two biological states (Phenotypes like up and down regulated genes or miRNAs using pre-ranked analysis).

For each gene-set (Supplementary Table 6), based on the over-representation of members of the gene-set towards the top or bottom of a list of the differentially expressed miRNA (Fig. 7B), pre-ranked by the strength of their fold change (positive or negative) and with the statistical significance, p-value < 0.05 and FDR ≤ 0.25 is calculated by weighted Kolmogorov-Smirnov statistic, adjusted for gene-set size (known as the Normalized Enrichment Score, NES^43^). The statistical significance (p-value and False discovery Rate) of NES score is estimated by a permutation test based on random shuffling of the phenotype or tag (gene) labels. We get 5 down and 30 up miRNAs enriched and associated with in 43 up and 52 down mRNAs (Supplementary Fig. 2) as potential regulator of these mRNAs.

### MiRNA functional and pathway annotations/associations, network construction and analysis

To extend our efforts to understand and elucidate the complex regulation of the miRNA-mRNA and the functional association with the pathways in which these miRNAs may be involved, we constructed the miRNA-mRNA network using the fold changes as edge weights of the network and Cytoscape as visualizer^49^. The enriched miRNA-mRNA as mentioned in Supplementary Fig. 2 and the pathways/GOBP in Supplementary Tables 2.2 and 2.3 were used to construct the network. The data of Supplementary Fig. 2 were parsed appropriately using various in-house written code in Perl/C/HTML to construct and visualize the networks. The flow of the functional annotation of these enriched miRNAs i.e. the association with related pathways/GOBP is shown in the Fig. 1B.

Out of all differentially expressed mRNAs, first the genes which are in the enriched pathways Supplementary Tables 2.2 and 2.3 were taken and mentioned as “mRNAs in pathways” in Fig. 1B. Then the overlapped genes from the enriched target genes (Supplementary Fig. 2) mentioned as “miRNA targets” in Fig. 1B are taken. Basically, these overlap genes indicate the functional and pathway associations of the differentially expressed mRNAs and miRNAs which are shown by shaded/hashed overlapped area as “miRNA-mRNA overlapped genes in enriched pathways” in Fig. 1B. The enriched pathways and GOBP associated with these overlapped genes are basically the pathways and GOBP which might be regulated by the enriched miRNAs and having these overlapped genes as their targets (Table 1 and network in Fig. 9 and Supplementary Fig. 3). The pathways and the biological process show that most of the genes interacting with the enriched miRNA are involved in Cell proliferation, migration, Differentiation or Apoptotic process (Table 1). Figure 8, network in Fig. 9 and Supplementary Fig. 3C also show the functional involvement of various miRNA clusters in cholesterol/sterol and lipid pathways.

### Data availability

All data is provided in supplementary data materials, including the normalized expression data of mRNA and miRNA.

## Electronic supplementary material

Supplementary data

Supplementary Table 1
