# Supplementary material for: Integrated analysis of mRNA and miRNA expression in HeLa cells expressing low levels of Nucleolin
Source: Sci Rep. 2017 Aug 21;7:9017. doi: 10.1038/s41598-017-09353-4 (PMC5567140; doi:10.1038/s41598-017-09353-4)
Supplement: Supplementary file 1 — Supplementary data [file 41598_2017_9353_MOESM1_ESM.pdf]

Integrated analysis of mRNA and miRNA expression in HeLa cells expressing low levels of Nucleolin

Sanjeev Kumar[3,5], Elizabhet Cruz Gomez[1], Mounira Chalabi-Dchar[1], Cong Rong[2], Sadhan Das[2], Iva Ugrinova[4], Xavier Gaume[2], Karine Monier[1], Fabien Mongelard[1,2], and Philippe Bouvet[1,2,5]

[1]Université de Lyon, Centre de Recherche en Cancérologie de Lyon, Cancer Cell Plasticity Department, UMR INSERM 1052 CNRS 5286, Centre Léon Bérard, Lyon, France.
[2] Université de Lyon, Ecole Normale Supérieure de Lyon, Lyon, France.
[3] BioCOS Life Sciences Private Limited,
AECS Layout, B-Block, Singasandra Hosur Road SAAMI Building, 851/A, 3rd Floor, Bengaluru, Karnataka India
[4]Institute of Molecular Biology "Acad. Roumen Tsanev" Bulgarian Academy of Sciences "Acad. G Bonchev str. bl. 21, 1113 Sofia, Bulgaria

[5]Corresponding authors
Philippe.bouvet@ens-lyon.fr
sanjeev@biocosls.in

Supplementary Figure 1:
KS statistics based enriched mRNA-pathways/GOBP.
S1A: For down regulated genes and S1B- for up regulated genes. The green (in S1A) or Red (S1B) shows the gene enriched in the corresponding pathway. The top panel shows the FC for the corresponding genes and the left panel shows the p-values of the enriched pathways/GOBP. p.4

Supplementary Figure 2:
KS statistics based enriched mRNA-miRNA. S2A: For down regulated genes and S2B- for up regulated genes. The green (in S2A) or Red (S2B) shows the gene enriched in the corresponding miRNA as target. The top panel shows the FC for the corresponding miRNA and the left panel shows the FC of the enriched target genes which is also differentially expressed. (U) refers to up regulated miRNA and (D) to down regulated miRNA. p.5

Supplementary Figure 3A**:**
Detailed Networks of the Differentially expressed and KS statistics based enriched mRNA-miRNA functional interaction p.6

Supplementary Figure 3B:
Sub network from S3A p.7

Supplementary Figure 3C:
Detailed Network of the Differentially expressed and KS statistics based enriched mRNA-miRNA functional interaction and the cholesterol/lipid pathways associations including the mir 17 Cluster. p.8

Supplementary Table 1:
The raw data: probeID- gene names- expression file see separate excel file

Supplementary Table 2.1:
List of the 925 and 764 genes that were down- and up-regulated respectively upon NCL depletion. p. 9

Supplementary Table 2.2:
Enriched pathways and GO BP terms for down regulated genes p. 29

Supplementary Table 2.3:
Enriched pathways and GO BP terms for Up regulated genes p. 31

Supplementary Table 3:
Raw data of miRNA expression. p. 45

Supplementary Table 4:
Up and down regulated miRNAs p. 65

Supplementary Table 5:
Pathways and GOBP databases used in Enrichment in Fig.1A p. 68

Supplementary Table 6:
Resources used to compile miRNA targets data p. 69

Supplementary Table 7.1:
Literature evidence of the target genes for the miRNA in supplementary Figs S2A and S2B p. 70

Supplementary Table 7.2:
Comparison with Tarbase miRNA/Target genes of supplementary Figs S2A and S2B. p. 87

S1A

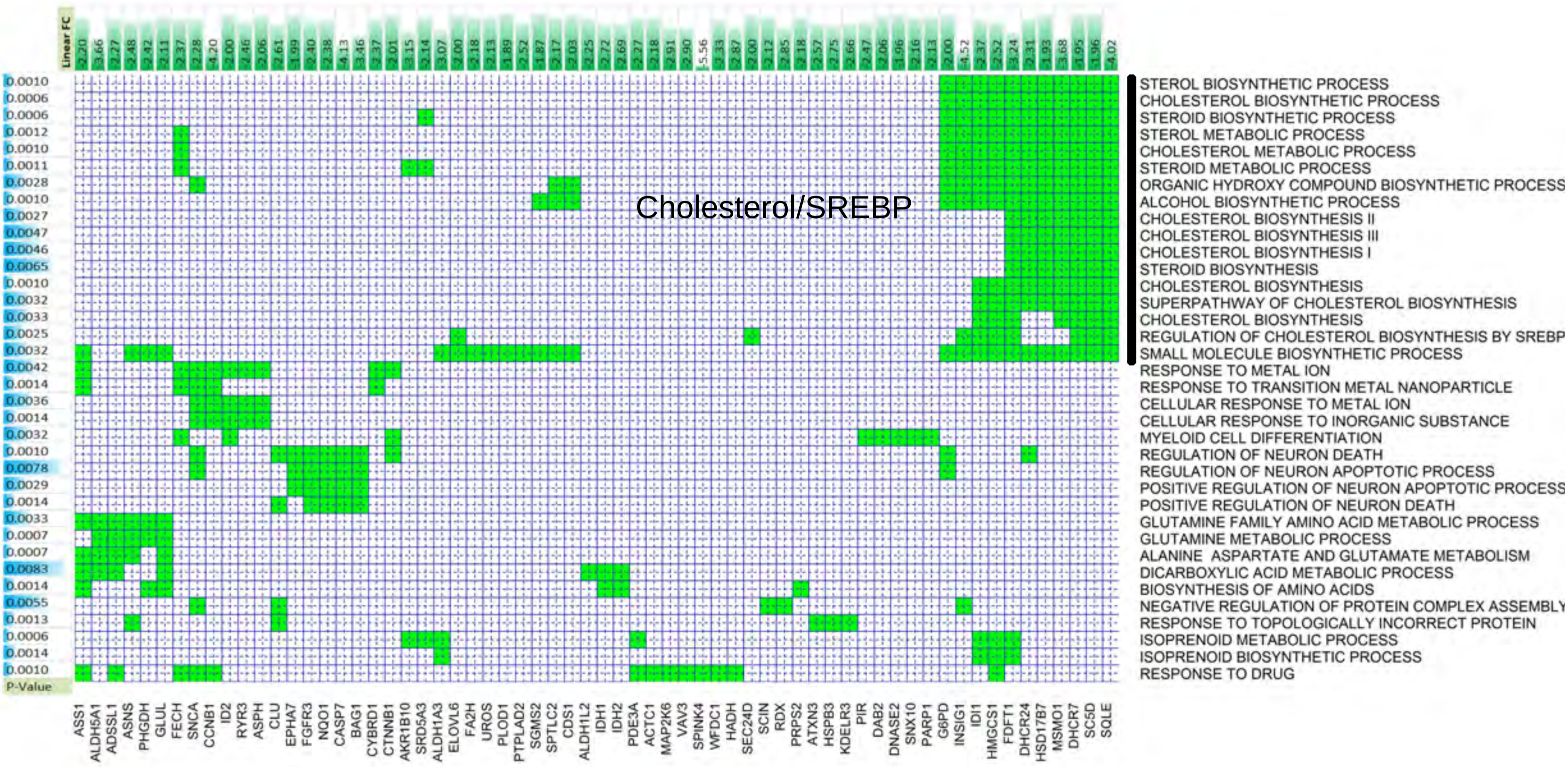

Linear FC
Cholesterol/SREBP
P-Value
STEROL BIOSYNTHETIC PROCESS
CHOLESTEROL BIOSYNTHETIC PROCESS
STEROID BIOSYNTHETIC PROCESS
STEROL METABOLIC PROCESS
CHOLESTEROL METABOLIC PROCESS
STEROID METABOLIC PROCESS
ORGANIC HYDROXY COMPOUND BIOSYNTHETIC PROCESS
ALCOHOL BIOSYNTHETIC PROCESS
CHOLESTEROL BIOSYNTHESIS II
CHOLESTEROL BIOSYNTHESIS III
CHOLESTEROL BIOSYNTHESIS I
STEROID BIOSYNTHESIS
CHOLESTEROL BIOSYNTHESIS
SUPERPATHWAY OF CHOLESTEROL BIOSYNTHESIS
CHOLESTEROL BIOSYNTHESIS
REGULATION OF CHOLESTEROL BIOSYNTHESIS BY SREBP
SMALL MOLECULE BIOSYNTHETIC PROCESS
RESPONSE TO METAL ION
RESPONSE TO TRANSITION METAL NANOPARTICLE
CELLULAR RESPONSE TO METAL ION
CELLULAR RESPONSE TO INORGANIC SUBSTANCE
MYELOID CELL DIFFERENTIATION
REGULATION OF NEURON DEATH
REGULATION OF NEURON APOPTOTIC PROCESS
POSITIVE REGULATION OF NEURON APOPTOTIC PROCESS
POSITIVE REGULATION OF NEURON DEATH
GLUTAMINE FAMILY AMINO ACID METABOLIC PROCESS
GLUTAMINE METABOLIC PROCESS
ALANINE ASPARTATE AND GLUTAMATE METABOLISM
DICARBOXYLIC ACID METABOLIC PROCESS
BIOSYNTHESIS OF AMINO ACIDS
NEGATIVE REGULATION OF PROTEIN COMPLEX ASSEMBLY
RESPONSE TO TOPOLOGICALLY INCORRECT PROTEIN
ISOPRENOID METABOLIC PROCESS
ISOPRENOID BIOSYNTHETIC PROCESS
RESPONSE TO DRUG
ASS1 ALDH5A1 ADSSL1 ASNS PHGDH GLUL FECH SNCA CCNB1 ID2 RYR3 ASPH CLU EPHA7 FGFR3 NQO1 CASP7 BAG1 CYBRD1 CTNNB1 AKR1B10 SRD5A3 ALDH1A3 ELOVL6 FA2H UROS PLOD1 PTPLAD2 SGMS2 SPTLC2 CDS1 ALDH1L2 IDH1 IDH2 PDE3A ACTC1 MAP2K6 VAV3 SPINK4 WFDC1 HADH SEC24D SCIN RDX PRPS2 ATXN3 HSPB3 KDELR3 PIR DAB2 DNASE2 SNX10 PARP1 G6PD INSIG1 IDI1 HMGCS1 FDFT1 DHCR24 HSD17B7 MSMO1 DHCR7 SC5D SQLE

S1B

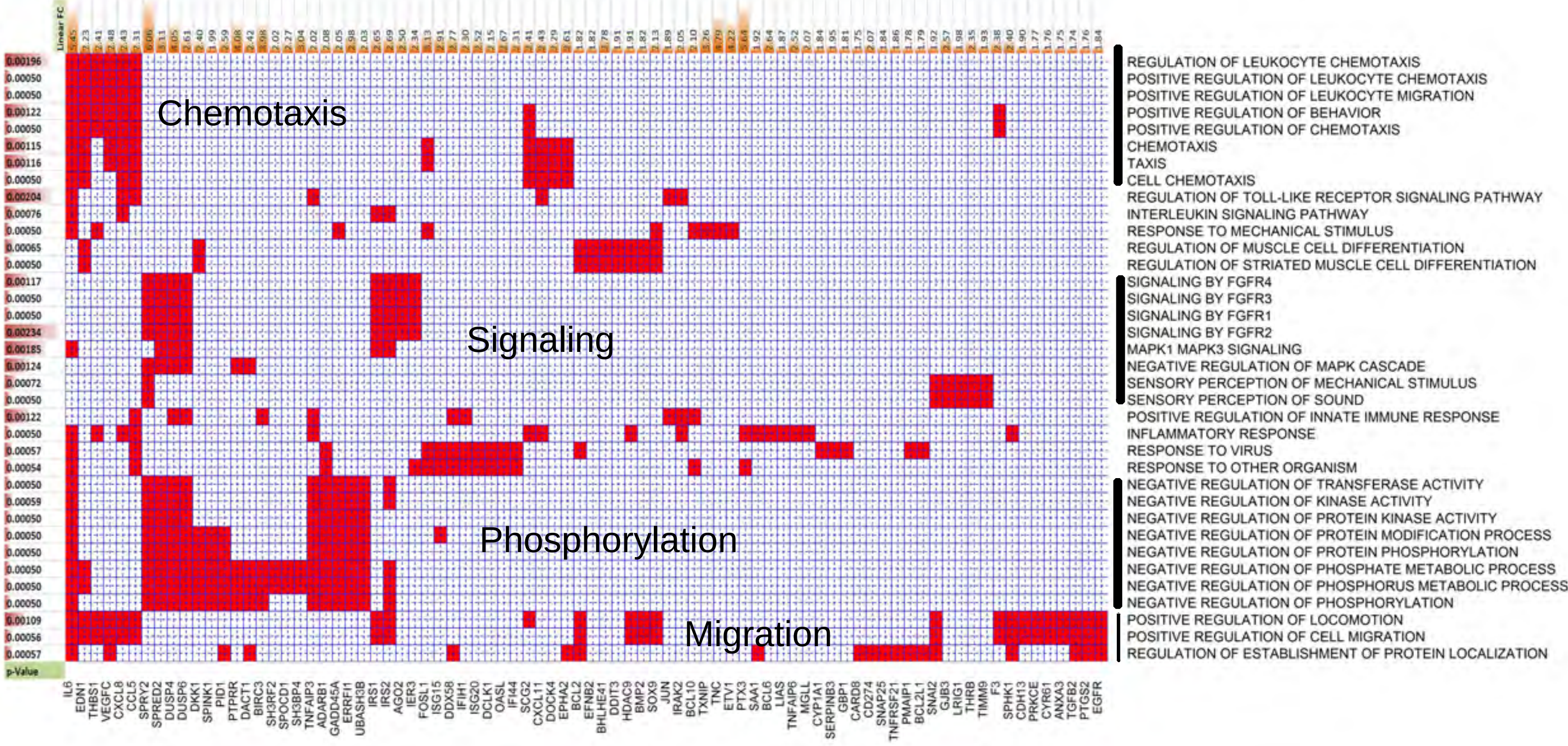

Linear FC
Chemotaxis
Signaling
Phosphorylation
Migration
p-Value
REGULATION OF LEUKOCYTE CHEMOTAXIS
POSITIVE REGULATION OF LEUKOCYTE CHEMOTAXIS
POSITIVE REGULATION OF LEUKOCYTE MIGRATION
POSITIVE REGULATION OF BEHAVIOR
POSITIVE REGULATION OF CHEMOTAXIS
CHEMOTAXIS
TAXIS
CELL CHEMOTAXIS
REGULATION OF TOLL-LIKE RECEPTOR SIGNALING PATHWAY
INTERLEUKIN SIGNALING PATHWAY
RESPONSE TO MECHANICAL STIMULUS
REGULATION OF MUSCLE CELL DIFFERENTIATION
REGULATION OF STRIATED MUSCLE CELL DIFFERENTIATION
SIGNALING BY FGFR4
SIGNALING BY FGFR3
SIGNALING BY FGFR1
SIGNALING BY FGFR2
MAPK1 MAPK3 SIGNALING
NEGATIVE REGULATION OF MAPK CASCADE
SENSORY PERCEPTION OF MECHANICAL STIMULUS
SENSORY PERCEPTION OF SOUND
POSITIVE REGULATION OF INNATE IMMUNE RESPONSE
INFLAMMATORY RESPONSE
RESPONSE TO VIRUS
RESPONSE TO OTHER ORGANISM
NEGATIVE REGULATION OF TRANSFERASE ACTIVITY
NEGATIVE REGULATION OF KINASE ACTIVITY
NEGATIVE REGULATION OF PROTEIN KINASE ACTIVITY
NEGATIVE REGULATION OF PROTEIN MODIFICATION PROCESS
NEGATIVE REGULATION OF PROTEIN PHOSPHORYLATION
NEGATIVE REGULATION OF PHOSPHATE METABOLIC PROCESS
NEGATIVE REGULATION OF PHOSPHORUS METABOLIC PROCESS
NEGATIVE REGULATION OF PHOSPHORYLATION
POSITIVE REGULATION OF LOCOMOTION
POSITIVE REGULATION OF CELL MIGRATION
REGULATION OF ESTABLISHMENT OF PROTEIN LOCALIZATION
IL6 EDN1 THBS1 VEGFC CXCL8 CCL5 SPRY2 SPRED2 DUSP4 DUSP6 DKK1 SPINK1 PID1 PTPRR DACT1 BIRC3 SH3RF2 SPOCD1 SH3BP4 TNFAIP3 ADARB1 GADD45A ERRFI1 UBASH3B IRS1 IRS2 AGO2 IER3 FOSL1 ISG15 DDX58 IFIH1 ISG20 DCLK1 OASL IFI44 SCG2 CXCL11 DOCK4 EPHA2 BCL2 EFNB2 BHLHE41 DDIT3 HDAC9 BMP2 SOX9 JUN IRAK2 BCL10 TXNIP TNC ETV1 PTX3 SAA1 BCL6 LIAS TNFAIP6 MGLL CYP1A1 SERPINB3 GBP1 CARD8 CD274 SNAP25 TNFRSF21 PMAIP1 BCL2L1 SNAI2 GJB3 LRIG1 THRB TIMM9 F3 SPHK1 CDH13 PRKCE CYR61 ANXA3 TGFB2 PTGS2 EGFR

S2A

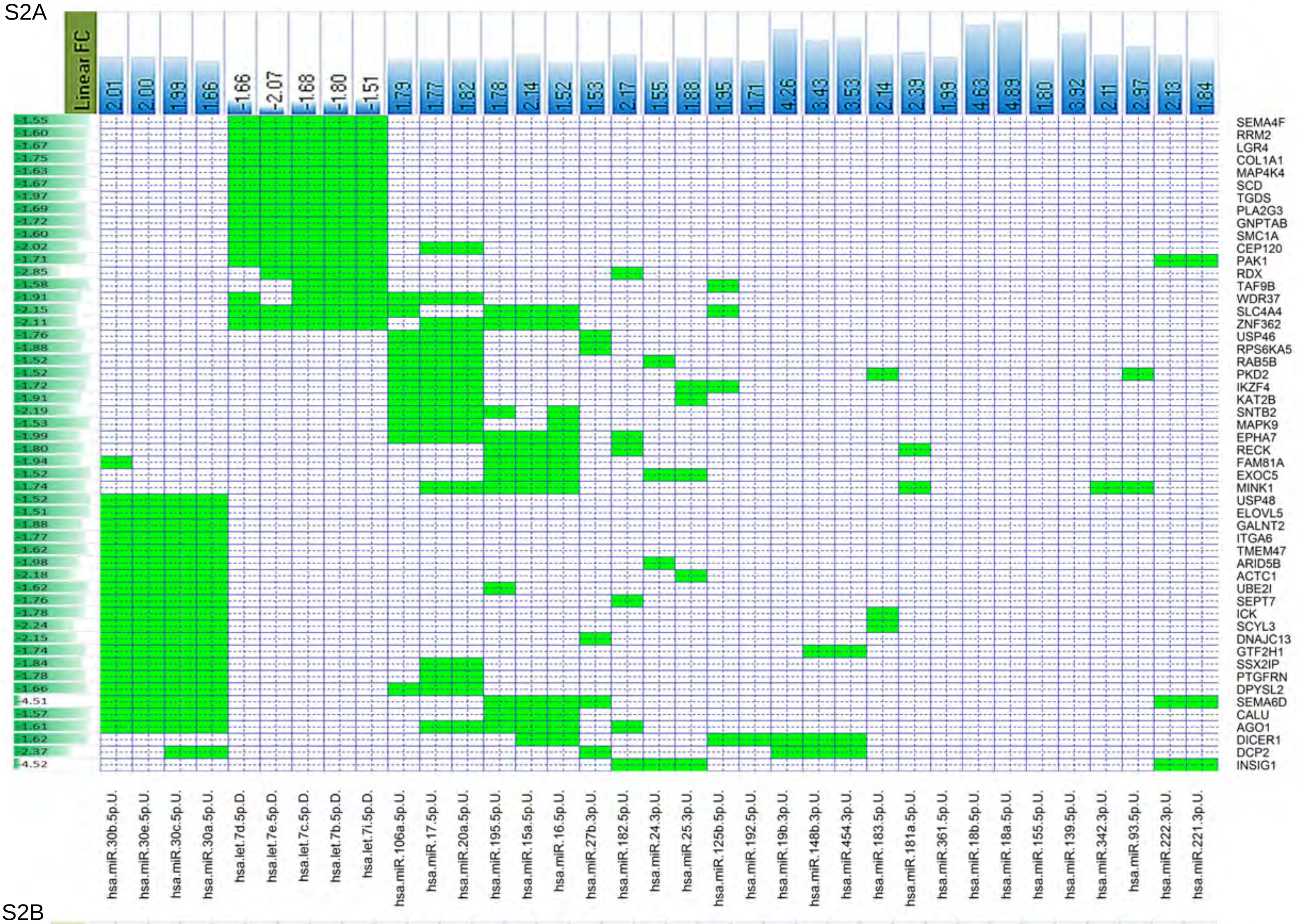
Linear FC
2.01 2.00 1.99 1.66 -1.66 -2.07 -1.68 -1.80 -1.51 1.79 1.77 1.82 1.78 2.14 1.52 1.53 2.17 1.55 1.88 1.95 1.71 4.26 3.43 3.53 2.14 2.39 1.99 4.63 4.89 1.80 3.92 2.11 2.97 2.13 1.84
SEMA4F
RRM2
LGR4
COL1A1
MAP4K4
SCD
TGDS
PLA2G3
GNPTAB
SMC1A
CEP120
PAK1
RDX
TAF9B
WDR37
SLC4A4
ZNF362
USP46
RPS6KA5
RAB5B
PKD2
IKZF4
KAT2B
SNTB2
MAPK9
EPHA7
RECK
FAM81A
EXOC5
MINK1
USP48
ELOVL5
GALNT2
ITGA6
TMEM47
ARID5B
ACTC1
UBE2I
SEPT7
ICK
SCYL3
DNAJC13
GTF2H1
SSX2IP
PTGFRN
DPYSL2
SEMA6D
CALU
AGO1
DICER1
DCP2
INSIG1
hsa.miR.30b.5p.U.
hsa.miR.30e.5p.U.
hsa.miR.30c.5p.U.
hsa.miR.30a.5p.U.
hsa.let.7d.5p.D.
hsa.let.7e.5p.D.
hsa.let.7c.5p.D.
hsa.let.7b.5p.D.
hsa.let.7i.5p.D.
hsa.miR.106a.5p.U.
hsa.miR.17.5p.U.
hsa.miR.20a.5p.U.
hsa.miR.195.5p.U.
hsa.miR.15a.5p.U.
hsa.miR.16.5p.U.
hsa.miR.27b.3p.U.
hsa.miR.182.5p.U.
hsa.miR.24.3p.U.
hsa.miR.25.3p.U.
hsa.miR.125b.5p.U.
hsa.miR.192.5p.U.
hsa.miR.19b.3p.U.
hsa.miR.148b.3p.U.
hsa.miR.454.3p.U.
hsa.miR.183.5p.U.
hsa.miR.181a.5p.U.
hsa.miR.361.5p.U.
hsa.miR.18b.5p.U.
hsa.miR.18a.5p.U.
hsa.miR.155.5p.U.
hsa.miR.139.5p.U.
hsa.miR.342.3p.U.
hsa.miR.93.5p.U.
hsa.miR.222.3p.U.
hsa.miR.221.3p.U.

S2B

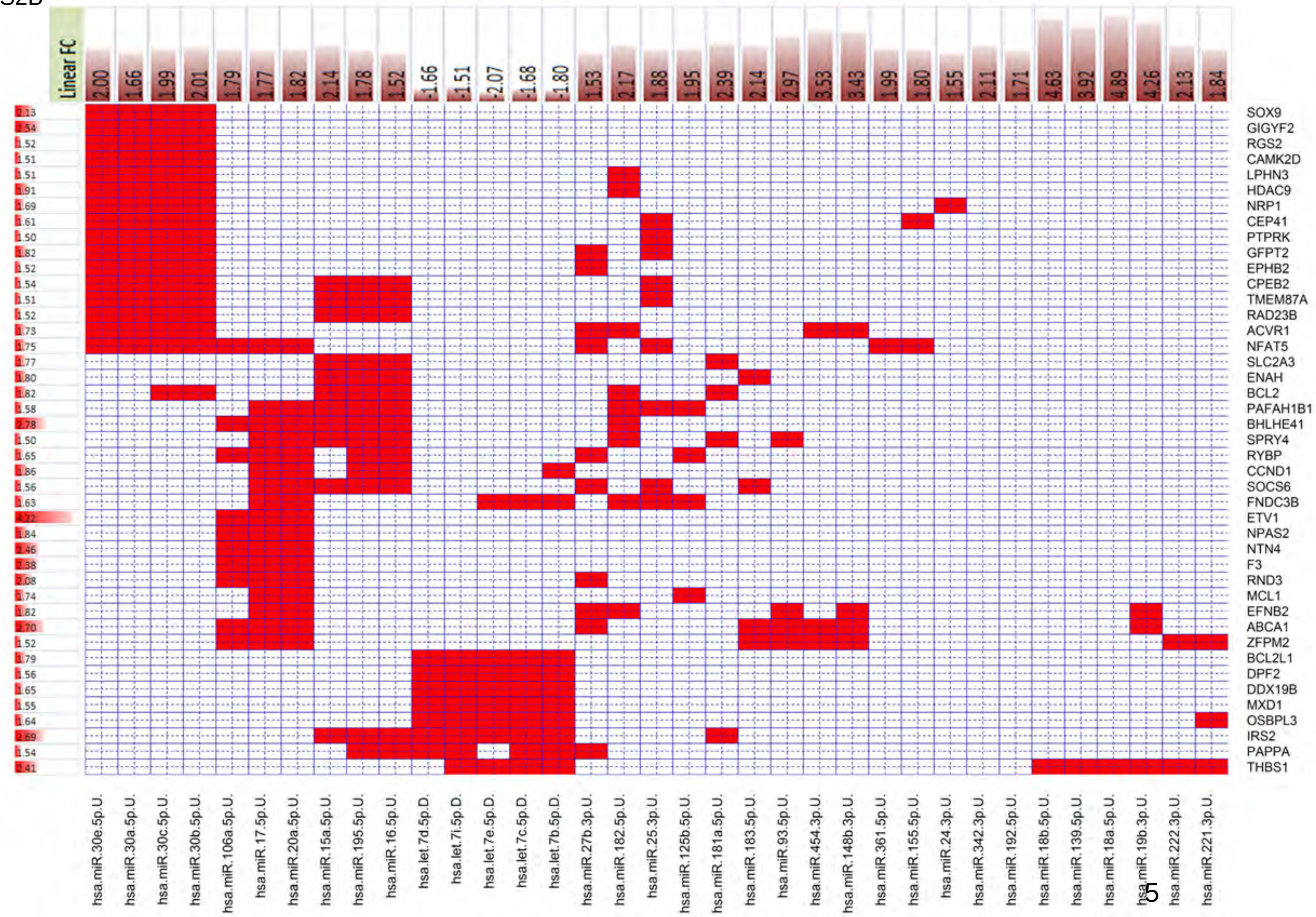
Linear FC
2.00 1.66 1.99 2.01 1.79 1.77 1.82 2.14 1.78 1.52 -1.66 -1.51 -2.07 -1.68 -1.80 1.53 2.17 1.88 1.95 2.39 2.14 2.97 3.53 3.43 1.99 1.80 1.55 2.11 1.71 4.63 3.92 4.89 4.26 2.13 1.84
SOX9
GIGYF2
RGS2
CAMK2D
LPHN3
HDAC9
NRP1
CEP41
PTPRK
GFPT2
EPHB2
CPEB2
TMEM87A
RAD23B
ACVR1
NFAT5
SLC2A3
ENAH
BCL2
PAFAH1B1
BHLHE41
SPRY4
RYBP
CCND1
SOCS6
FNDC3B
ETV1
NPAS2
NTN4
F3
RND3
MCL1
EFNB2
ABCA1
ZFPM2
BCL2L1
DPF2
DDX19B
MXD1
OSBPL3
IRS2
PAPPA
THBS1
hsa.miR.30e.5p.U.
hsa.miR.30a.5p.U.
hsa.miR.30c.5p.U.
hsa.miR.30b.5p.U.
hsa.miR.106a.5p.U.
hsa.miR.17.5p.U.
hsa.miR.20a.5p.U.
hsa.miR.15a.5p.U.
hsa.miR.195.5p.U.
hsa.miR.16.5p.U.
hsa.let.7d.5p.D.
hsa.let.7i.5p.D.
hsa.let.7e.5p.D.
hsa.let.7c.5p.D.
hsa.let.7b.5p.D.
hsa.miR.27b.3p.U.
hsa.miR.182.5p.U.
hsa.miR.25.3p.U.
hsa.miR.125b.5p.U.
hsa.miR.181a.5p.U.
hsa.miR.183.5p.U.
hsa.miR.93.5p.U.
hsa.miR.454.3p.U.
hsa.miR.148b.3p.U.
hsa.miR.361.5p.U.
hsa.miR.155.5p.U.
hsa.miR.24.3p.U.
hsa.miR.342.3p.U.
hsa.miR.192.5p.U.
hsa.miR.18b.5p.U.
hsa.miR.139.5p.U.
hsa.miR.18a.5p.U.
hsa.miR.19b.3p.U.
hsa.miR.222.3p.U.
hsa.miR.221.3p.U.

S3A

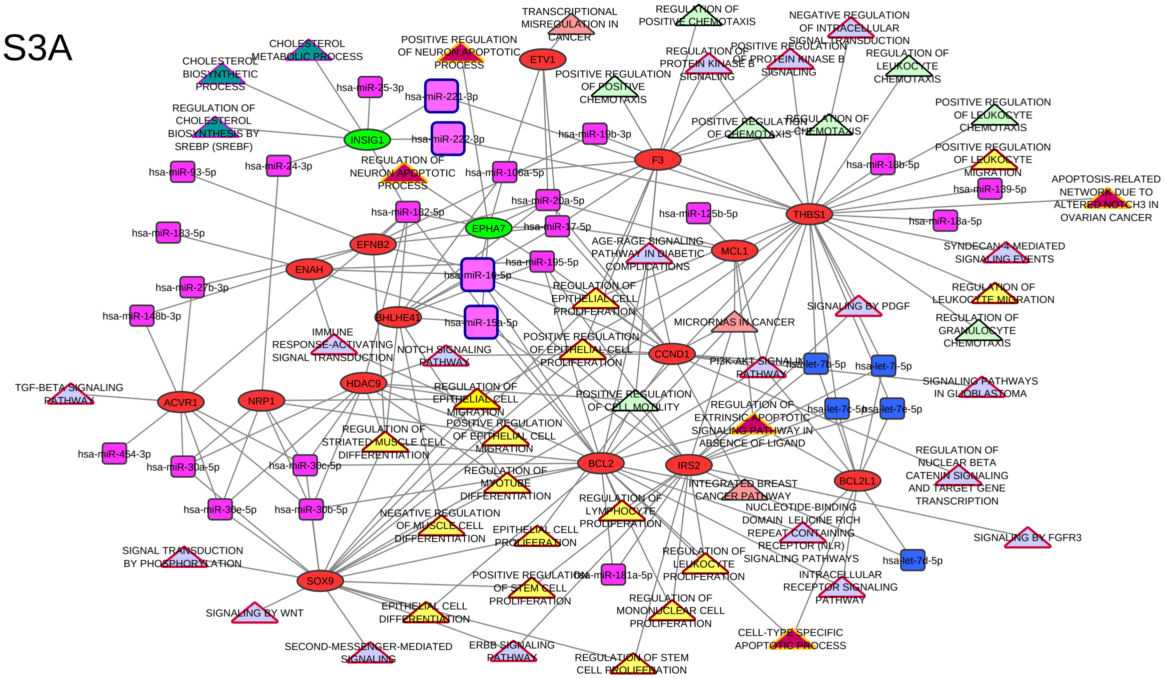

TRANSCRIPTIONAL MISREGULATION IN CANCER
REGULATION OF POSITIVE CHEMOTAXIS
NEGATIVE REGULATION OF INTRACELLULAR SIGNAL TRANSDUCTION
CHOLESTEROL METABOLIC PROCESS
POSITIVE REGULATION OF NEURON APOPTOTIC PROCESS
CHOLESTEROL BIOSYNTHETIC PROCESS
ETV1
REGULATION OF PROTEIN KINASE B SIGNALING
POSITIVE REGULATION OF PROTEIN KINASE B SIGNALING
REGULATION OF LEUKOCYTE CHEMOTAXIS
hsa-miR-25-3p
hsa-miR-221-3p
POSITIVE REGULATION OF POSITIVE CHEMOTAXIS
REGULATION OF CHOLESTEROL BIOSYNTHESIS BY SREBP (SREBF)
INSIG1
hsa-miR-222-3p
hsa-miR-19b-3p
POSITIVE REGULATION OF CHEMOTAXIS
REGULATION OF CHEMOTAXIS
POSITIVE REGULATION OF LEUKOCYTE CHEMOTAXIS
hsa-miR-93-5p
hsa-miR-24-3p
REGULATION OF NEURON APOPTOTIC PROCESS
hsa-miR-106a-5p
F3
hsa-miR-18b-5p
POSITIVE REGULATION OF LEUKOCYTE MIGRATION
hsa-miR-20a-5p
hsa-miR-139-5p
APOPTOSIS-RELATED NETWORK DUE TO ALTERED NOTCH3 IN OVARIAN CANCER
hsa-miR-182-5p
hsa-miR-125b-5p
THBS1
hsa-miR-18a-5p
hsa-miR-133-5p
EPHA7
hsa-miR-17-5p
EFNB2
AGE-RAGE SIGNALING PATHWAY IN DIABETIC COMPLICATIONS
MCL1
SYNDECAN-4-MEDIATED SIGNALING EVENTS
ENAH
hsa-miR-195-5p
hsa-miR-16-5p
hsa-miR-27b-3p
REGULATION OF EPITHELIAL CELL PROLIFERATION
REGULATION OF LEUKOCYTE MIGRATION
SIGNALING BY PDGF
hsa-miR-148b-3p
BHLHE41
hsa-miR-15a-5p
MICRORNAS IN CANCER
REGULATION OF GRANULOCYTE CHEMOTAXIS
IMMUNE RESPONSE-ACTIVATING SIGNAL TRANSDUCTION
POSITIVE REGULATION OF EPITHELIAL CELL PROLIFERATION
NOTCH SIGNALING PATHWAY
CCND1
PI3K-AKT SIGNALING PATHWAY
hsa-let-7b-5p
hsa-let-7i-5p
SIGNALING PATHWAYS IN GLIOBLASTOMA
TGF-BETA SIGNALING PATHWAY
HDAC9
ACVR1
NRP1
REGULATION OF EPITHELIAL CELL MIGRATION
POSITIVE REGULATION OF CELL MOTILITY
REGULATION OF EXTRINSIC APOPTOTIC SIGNALING PATHWAY IN ABSENCE OF LIGAND
hsa-let-7c-5p
hsa-let-7e-5p
POSITIVE REGULATION OF EPITHELIAL CELL MIGRATION
REGULATION OF STRIATED MUSCLE CELL DIFFERENTIATION
REGULATION OF NUCLEAR BETA CATENIN SIGNALING AND TARGET GENE TRANSCRIPTION
hsa-miR-454-3p
hsa-miR-30a-5p
hsa-miR-30c-5p
BCL2
IRS2
REGULATION OF MYOTUBE DIFFERENTIATION
BCL2L1
INTEGRATED BREAST CANCER PATHWAY
hsa-miR-30e-5p
hsa-miR-30b-5p
REGULATION OF LYMPHOCYTE PROLIFERATION
NUCLEOTIDE-BINDING DOMAIN, LEUCINE RICH REPEAT CONTAINING RECEPTOR (NLR) SIGNALING PATHWAYS
NEGATIVE REGULATION OF MUSCLE CELL DIFFERENTIATION
EPITHELIAL CELL PROLIFERATION
SIGNALING BY FGFR3
SIGNAL TRANSDUCTION BY PHOSPHORYLATION
REGULATION OF LEUKOCYTE PROLIFERATION
hsa-let-7d-5p
SOX9
POSITIVE REGULATION OF STEM CELL PROLIFERATION
hsa-miR-181a-5p
INTRACELLULAR RECEPTOR SIGNALING PATHWAY
SIGNALING BY WNT
EPITHELIAL CELL DIFFERENTIATION
REGULATION OF MONONUCLEAR CELL PROLIFERATION
CELL-TYPE SPECIFIC APOPTOTIC PROCESS
SECOND-MESSENGER-MEDIATED SIGNALING
ERBB SIGNALING PATHWAY
REGULATION OF STEM CELL PROLIFERATION

S3B

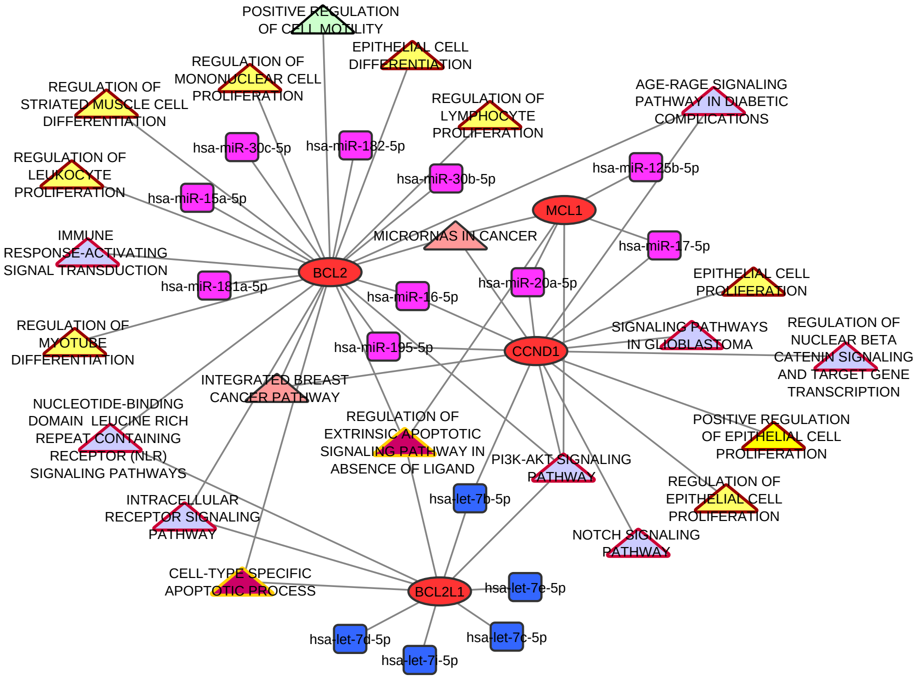
POSITIVE REGULATION OF CELL MOTILITY
EPITHELIAL CELL DIFFERENTIATION
REGULATION OF MONONUCLEAR CELL PROLIFERATION
REGULATION OF STRIATED MUSCLE CELL DIFFERENTIATION
REGULATION OF LYMPHOCYTE PROLIFERATION
AGE-RAGE SIGNALING PATHWAY IN DIABETIC COMPLICATIONS
REGULATION OF LEUKOCYTE PROLIFERATION
hsa-miR-30c-5p
hsa-miR-182-5p
hsa-miR-30b-5p
hsa-miR-125b-5p
hsa-miR-15a-5p
MCL1
IMMUNE RESPONSE-ACTIVATING SIGNAL TRANSDUCTION
MICRORNAS IN CANCER
hsa-miR-17-5p
BCL2
hsa-miR-181a-5p
hsa-miR-20a-5p
EPITHELIAL CELL PROLIFERATION
hsa-miR-16-5p
REGULATION OF MYOTUBE DIFFERENTIATION
SIGNALING PATHWAYS IN GLIOBLASTOMA
REGULATION OF NUCLEAR BETA CATENIN SIGNALING AND TARGET GENE TRANSCRIPTION
hsa-miR-195-5p
CCND1
INTEGRATED BREAST CANCER PATHWAY
NUCLEOTIDE-BINDING DOMAIN LEUCINE RICH REPEAT CONTAINING RECEPTOR (NLR) SIGNALING PATHWAYS
REGULATION OF EXTRINSIC APOPTOTIC SIGNALING PATHWAY IN ABSENCE OF LIGAND
POSITIVE REGULATION OF EPITHELIAL CELL PROLIFERATION
PI3K-AKT SIGNALING PATHWAY
REGULATION OF EPITHELIAL CELL PROLIFERATION
hsa-let-7b-5p
INTRACELLULAR RECEPTOR SIGNALING PATHWAY
NOTCH SIGNALING PATHWAY
CELL-TYPE SPECIFIC APOPTOTIC PROCESS
BCL2L1
hsa-let-7e-5p
hsa-let-7d-5p
hsa-let-7c-5p
hsa-let-7i-5p

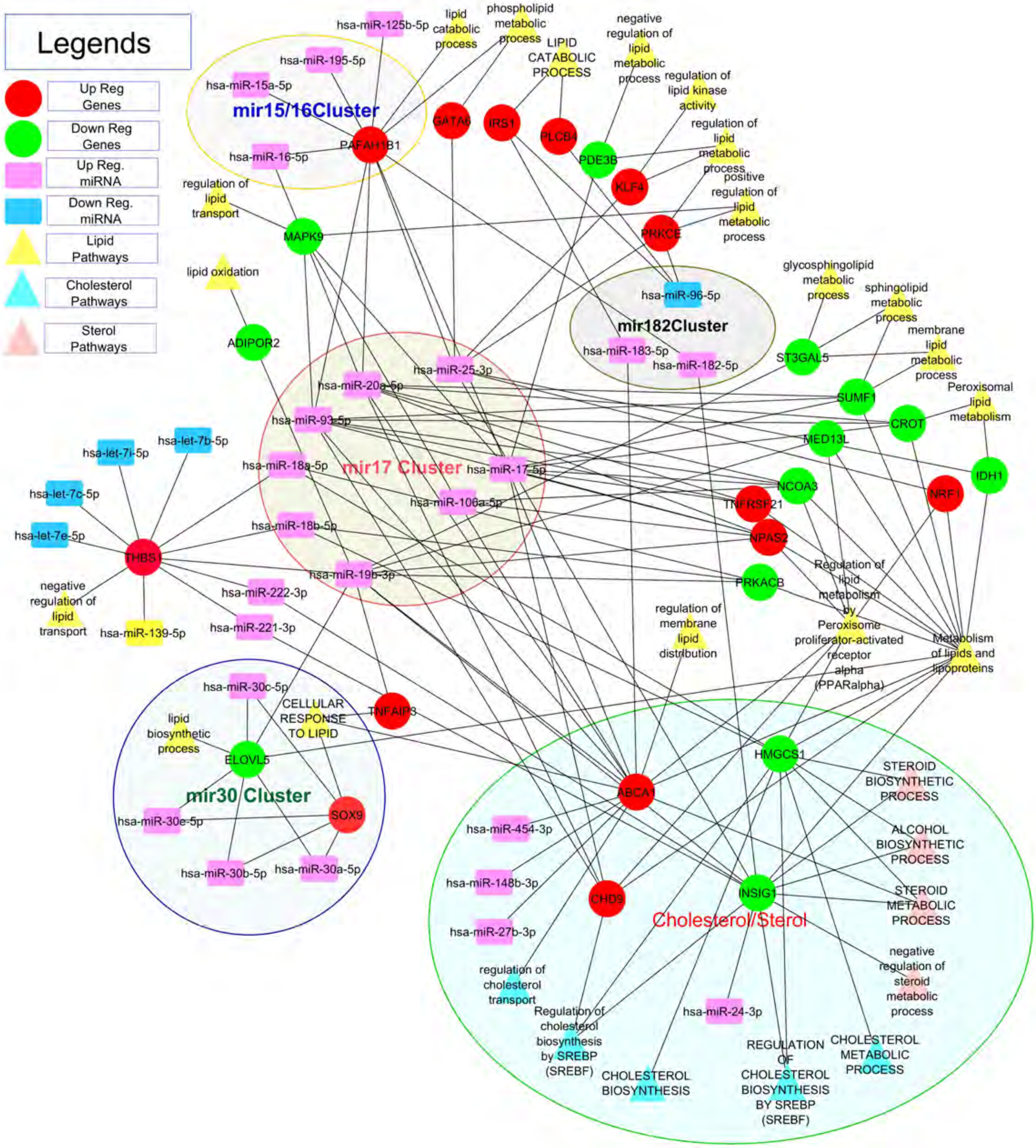

Legends
Up Reg Genes
Down Reg Genes
Up Reg. miRNA
Down Reg. miRNA
Lipid Pathways
Cholesterol Pathways
Sterol Pathways
mir15/16Cluster
mir182Cluster
mir17 Cluster
mir30 Cluster
Cholesterol/Sterol
hsa-miR-125b-5p
hsa-miR-195-5p
hsa-miR-15a-5p
hsa-miR-16-5p
PAFAH1B1
GATA6
IRS1
PLCB4
PDE3B
KLF4
PRKCE
MAPK9
ADIPOR2
hsa-miR-96-5p
hsa-miR-183-5p
hsa-miR-182-5p
ST3GAL5
SUMF1
CROT
MED13L
IDH1
NCOA3
NRF1
TNFRSF21
NPAS2
PRKACB
hsa-miR-25-3p
hsa-miR-20a-5p
hsa-miR-93-5p
hsa-miR-18a-5p
hsa-miR-17-5p
hsa-miR-106a-5p
hsa-miR-18b-5p
hsa-miR-19b-3p
hsa-let-7i-5p
hsa-let-7b-5p
hsa-let-7c-5p
hsa-let-7e-5p
THBS1
hsa-miR-222-3p
hsa-miR-221-3p
hsa-miR-139-5p
hsa-miR-30c-5p
hsa-miR-30e-5p
hsa-miR-30b-5p
hsa-miR-30a-5p
ELOVL5
SOX9
TNFAIP3
ABCA1
HMGCS1
INSIG1
CHD9
hsa-miR-454-3p
hsa-miR-148b-3p
hsa-miR-27b-3p
hsa-miR-24-3p
lipid catabolic process
phospholipid metabolic process
LIPID CATABOLIC PROCESS
negative regulation of lipid metabolic process
regulation of lipid kinase activity
regulation of lipid metabolic process
positive regulation of lipid metabolic process
regulation of lipid transport
lipid oxidation
glycosphingolipid metabolic process
sphingolipid metabolic process
membrane lipid metabolic process
Peroxisomal lipid metabolism
Regulation of lipid metabolism by Peroxisome proliferator-activated receptor alpha (PPARalpha)
Metabolism of lipids and lipoproteins
regulation of membrane lipid distribution
negative regulation of lipid transport
CELLULAR RESPONSE TO LIPID
lipid biosynthetic process
STEROID BIOSYNTHETIC PROCESS
ALCOHOL BIOSYNTHETIC PROCESS
STEROID METABOLIC PROCESS
negative regulation of steroid metabolic process
regulation of cholesterol transport
Regulation of cholesterol biosynthesis by SREBP (SREBF)
CHOLESTEROL BIOSYNTHESIS
REGULATION OF CHOLESTEROL BIOSYNTHESIS BY SREBP (SREBF)
CHOLESTEROL METABOLIC PROCESS

Supplementary Table 2.1:

List of the 925 and 764 genes that were down- and up-regulated respectively upon NCL depletion.

Up Regulated Genes				Down Regulated Regulated Genes			
Gene Symbol	LinearFC	P Value	BH adj P Val	Gene Symbo	LinearFC	P Value	BH adj P Val
SPRY2	6.0629187	0.0001002	0.0059938	NCL	-15.354377	4.33E-08	0.000215109
LETM2	6.0207559	6.97E-06	0.0018509	F11R	-6.2731983	1.26E-08	0.000177989
PTX3	5.6442257	7.85E-06	0.001969	SPINK4	-5.5606765	3.95E-08	0.000215109
IL6	5.4548391	1.50E-07	0.0003914	C8orf44-SGK3	-5.1937282	2.77E-09	0.00015151
RSPO3	5.1661101	8.40E-07	0.0007766	INSIG1	-4.5237666	2.64E-07	0.00055611
TNC	4.7917418	6.31E-08	0.0002184	SEMA6D	-4.5139722	3.31E-05	0.003501911
CLDN1	4.5193496	2.06E-05	0.0028074	CCNB1	-4.2001852	6.17E-06	0.001730288
NAV3	4.4469573	0.00012145	0.0066006	VASH2	-4.1367888	5.86E-09	0.000160088
ETV1	4.2160047	0.00110793	0.022561	CASP7	-4.1277305	2.93E-08	0.000177989
SERTAD4-AS1	4.2045118	3.35E-05	0.0035264	SQLE	-4.024184	2.71E-08	0.000177989
PTPRR	4.078714	1.29E-05	0.0023649	REEP5	-3.9605307	3.58E-07	0.00064127
DUSP4	4.0483118	8.51E-06	0.0020227	DENND6A	-3.8145581	1.24E-07	0.000377644
BIRC3	3.9750451	4.52E-06	0.0016344	MPPED2	-3.6971511	7.74E-05	0.005250669
HHAT	3.8451755	5.43E-05	0.0044926	MSMO1	-3.6825694	5.95E-08	0.000218393
AMIGO2	3.8274542	2.23E-05	0.0028846	ALDH5A1	-3.6585048	5.35E-08	0.000218393
PHLDA1	3.5022799	0.00021702	0.0089416	ENPP1	-3.5561954	1.66E-08	0.000177989
LOC101928710	3.3608222	6.39E-08	0.0002184	BAG1	-3.4635987	6.06E-07	0.000743678
COL13A1	3.3024279	8.37E-05	0.0054479	RAB40B	-3.4230034	1.06E-06	0.000837073
TXNIP	3.2644751	2.10E-06	0.001273	RP1-193H18.	-3.4096597	8.58E-06	0.002022734
IFRD1	3.1488918	6.11E-06	0.0017296	MPZL2	-3.3687561	2.15E-06	0.001272984
FOSL1	3.1308351	0.00053781	0.0148507	FDFT1	-3.2423082	2.11E-08	0.000177989
SPRED2	3.1063569	0.00012082	0.006583	METTL7A	-3.198265	0.0007354	0.017611846
SH3BP4	3.0417676	6.75E-07	0.0007437	AKR1B10	-3.1469658	3.63E-05	0.003644738
RP1-78O14.1	3.0298039	9.76E-06	0.0021599	MMP13	-3.1352084	3.53E-06	0.001463899
ERRFI1	2.9764168	1.80E-05	0.0026469	SLC12A3	-3.1200346	3.21E-06	0.001463805
LINC01094	2.9621682	3.65E-05	0.0036621	RP11-355B11	-3.093468	5.52E-07	0.000743678
MYEOV	2.9463021	1.15E-05	0.0022619	ALDH1A3	-3.0653259	0.0004826	0.013770474
ISG15	2.9089167	3.04E-06	0.0014593	H19 /// MIR6	-2.9769566	2.88E-06	0.001459329
SCG5	2.8256679	0.00010718	0.0062151	FAM115A ///	-2.9176457	6.90E-07	0.000743678
CASC10	2.7893634	9.51E-06	0.0021311	MAP2K6	-2.9094594	0.000155	0.007438976
BHLHE41	2.7819336	9.21E-06	0.0020903	VAV3	-2.9023887	2.27E-05	0.002884643
DDX58	2.7673908	5.30E-05	0.0044565	LINC00470	-2.8737137	5.20E-07	0.000743678
IL7R	2.7490237	5.89E-06	0.0017039	HADH	-2.8653611	1.53E-06	0.001048033
CCDC71L	2.7172982	0.00032687	0.0111644	NXPE3	-2.8514833	7.04E-07	0.000743678
ABCA1	2.6980833	0.00293597	0.0406604	RDX	-2.8508886	3.09E-06	0.001463805
IRS2	2.6948426	2.40E-05	0.0029896	OLFML2A	-2.8400036	0.0001943	0.008471931
OASL	2.6687947	0.00013327	0.0068483	HOTS	-2.8263223	1.32E-06	0.000946775
IRS1	2.6499502	5.09E-05	0.0043473	SPTSSA	-2.8238196	3.35E-06	0.001463805
BCL6	2.6357597	8.95E-07	0.0007766	SMIM14	-2.7565739	0.0034903	0.045053996
RGS17	2.6295981	0.0003093	0.0108194	HSPB3	-2.7524832	5.60E-06	0.001673101
SAT1	2.6165433	4.72E-06	0.0016344	NETO2	-2.7485204	1.96E-07	0.000464994
TCEA2	2.611868	9.96E-05	0.0059694	ETNPPL	-2.7317414	0.001191	0.023531362
EPHA2	2.6086862	2.44E-05	0.0030094	IDH1	-2.720034	1.04E-05	0.002187732
DUSP6	2.6057013	9.49E-05	0.0058128	RHOBTB3	-2.7118064	4.65E-05	0.004115692
CPA3	2.5952139	5.76E-07	0.0007437	KRCC1	-2.7092506	0.0002016	0.008565061
PID1	2.5866212	2.54E-06	0.0013618	IDH2	-2.6909643	7.30E-06	0.001909802
GJB3	2.5724963	8.37E-07	0.0007766	KDELR3	-2.6612589	1.09E-05	0.002195492
GIGYF2	2.5440871	3.00E-06	0.0014593	SLC41A2	-2.6543429	1.96E-05	0.002756725

FOXD1	2.5174955	5.53E-05	0.004509	MEGF9	-2.6516325	3.39E-06	0.001463899
ISG20	2.516952	6.50E-06	0.0017586	SPOCK3	-2.6447183	0.0004744	0.013630278
TNFAIP6	2.5150537	0.00034349	0.0114165	HEATR5A	-2.6174191	1.53E-06	0.001048033
LINC01106 ///	2.511548	2.15E-07	0.000489	CLU	-2.6136447	1.13E-05	0.00223222
LOC100996843	2.5074195	2.27E-05	0.0028846	TMTC4	-2.5949999	3.77E-07	0.00064127
AGO2 /// CASC	2.4989187	0.00037985	0.0120258	RPL31 /// TB(	-2.5735147	7.41E-08	0.000238404
NALCN	2.4951667	3.49E-05	0.0035739	ATXN3	-2.5735128	1.49E-07	0.000391415
VEGFC	2.4818917	0.00011464	0.0064091	YPEL2	-2.5674583	1.41E-05	0.00239658
NTN4	2.461525	0.00132036	0.0249536	ATP8A1	-2.5616244	7.66E-06	0.001941767
FST	2.4353893	8.19E-05	0.0053813	DPY19L4	-2.5480969	5.74E-06	0.001687058
CXCL11	2.433385	0.00100647	0.0212403	PHTF1	-2.539172	3.97E-06	0.001572583
CXCL8	2.4321691	0.00071627	0.0174342	HMGCS1	-2.5186694	2.42E-06	0.001348487
PPP2R2D	2.4253837	1.98E-05	0.0027799	PTPLAD2	-2.5158217	3.23E-05	0.00347197
DACT1	2.4228129	1.27E-05	0.0023649	GPR180	-2.5132898	0.0002548	0.009772891
WDR20	2.4218504	7.07E-07	0.0007437	ND6	-2.4843285	0.0001321	0.006825365
SCG2	2.4110135	5.27E-06	0.0016406	ASNS	-2.4842551	7.54E-06	0.001941767
PDP1	2.4070953	0.0001253	0.0066657	CCNE2	-2.4679836	8.20E-06	0.002001743
THBS1	2.4052274	0.00282023	0.0396615	PIR	-2.4658274	1.12E-06	0.00085374
SPHK1	2.4020559	4.38E-06	0.0016283	FXR1	-2.4605478	4.12E-05	0.003870577
DKK1	2.4009208	0.00078882	0.0184233	RYR3	-2.4604286	4.66E-05	0.004115692
LOC541472	2.3893763	1.06E-05	0.0021926	PSIP1	-2.4545225	1.31E-05	0.002364945
DAZAP2 /// LO(	2.3867239	8.09E-07	0.0007766	KANK4	-2.4481144	0.0037529	0.047191309
F3	2.3836208	0.00080298	0.0186795	HPDL	-2.4307254	4.72E-06	0.001634419
NEXN	2.3731397	5.53E-06	0.0016721	SH3YL1	-2.4261488	0.0006396	0.016395634
SNAPC1	2.3658966	1.04E-05	0.0021877	CPE	-2.4259819	6.49E-05	0.004850755
ELOVL2	2.3563939	3.43E-06	0.0014639	PHGDH	-2.423155	5.54E-06	0.001672145
THRB	2.3505794	0.00033349	0.0112572	SIPA1L1	-2.4216772	5.19E-06	0.001639622
IER3	2.3405485	7.59E-06	0.0019418	ACADL	-2.4132863	1.09E-06	0.00084284
ENC1	2.3335326	0.00011447	0.0064058	ANKRD9	-2.4054971	3.71E-06	0.001515186
IFI44	2.3134812	0.00028529	0.0102551	FGFR3	-2.3997901	4.19E-06	0.00162371
CCL5	2.3095981	0.00042087	0.012692	FAM210B	-2.3985661	1.22E-05	0.002364945
IFIH1	2.2960155	2.01E-05	0.0028074	RHOU	-2.3983864	0.0001656	0.007700393
RAD51B	2.2872866	1.40E-05	0.0023885	NQO1	-2.3840395	3.87E-07	0.00064127
DOCK4	2.2863068	0.00021778	0.0089661	CYBRD1	-2.3681558	7.55E-06	0.001941767
ARMCX4	2.2792242	0.00014165	0.0070773	FECH	-2.366915	3.22E-07	0.000628672
NFYA	2.275542	1.12E-05	0.0022222	DCP2	-2.3657401	2.69E-05	0.003146845
SFT2D3 /// WD	2.2750977	0.00038944	0.0121672	IDI1	-2.3652274	6.14E-07	0.000743678
AKIP1 /// NUAK	2.2727428	5.71E-06	0.0016871	LPPR4	-2.3570286	6.25E-06	0.001742432
SPOCD1	2.2684223	2.26E-06	0.001273	SMAD9	-2.3339286	1.64E-05	0.00251793
DHRS2	2.2568687	1.91E-06	0.0011876	WFDC1	-2.3304108	0.0004782	0.013696964
TMEM99	2.2276755	3.50E-06	0.0014639	GCA	-2.3280113	0.0001031	0.006093718
EDN1	2.2263708	1.09E-05	0.0022009	COL9A3	-2.3257069	0.0001325	0.006825365
OXR1	2.2054802	1.65E-06	0.0010867	HTATIP2	-2.3147377	6.61E-06	0.001781273
RSL1D1	2.2020374	5.14E-06	0.0016347	ATP6V0E1	-2.3118746	1.72E-05	0.002583394
TMEM79	2.1942424	0.00019468	0.0084719	DHCR24	-2.3071339	8.23E-07	0.000776563
TMEM156	2.1817507	0.00066586	0.0166533	IPP	-2.2970499	4.15E-05	0.003877503
PTPRG	2.1804874	0.00171118	0.0292817	GOLIM4	-2.2897344	2.93E-06	0.001459329
LINC00623 ///	2.1722299	1.63E-06	0.0010867	SNCA	-2.2764001	1.04E-05	0.002187732
ROR1	2.1712782	0.00028672	0.0102865	COL5A1	-2.2710431	9.25E-07	0.000783881
ANO2	2.1699229	6.13E-05	0.0046915	ADSSL1	-2.2698008	0.000988	0.020961226

LINC00909	2.1652447	4.22E-06	0.0016237	CENPU	-2.2694424	0.0001952	0.008471931
ANTXR2	2.1575119	0.0009089	0.0198697	PDE3A	-2.2662825	2.48E-05	0.003024597
DCLK1	2.1528266	0.0010866	0.02229	SERF1A /// SE	-2.263986	6.97E-07	0.000743678
LOC101928820	2.1443943	0.00282772	0.0397157	SCARA5	-2.2566187	8.36E-05	0.005447873
SOX9	2.1324953	0.00326117	0.0432359	ALDH1L2	-2.2515246	8.73E-06	0.00203882
INF2	2.1324907	4.78E-06	0.0016344	SCYL3	-2.2395207	3.35E-06	0.001463805
RNF114	2.1280874	9.52E-07	0.0007839	NLN	-2.236649	8.80E-05	0.005589333
FAM167A	2.1273225	5.73E-05	0.0045511	TSPAN13	-2.217856	4.31E-06	0.001628344
GNGT1	2.1248075	0.00056989	0.0153718	SYTL5	-2.2167982	9.85E-05	0.005924131
RP11-504A18.1	2.110238	0.00063178	0.016286	GPR126	-2.2154922	8.03E-07	0.000776563
BCL10	2.0965302	0.00061197	0.0160092	RBM4	-2.2021759	3.19E-05	0.003461684
SLC22A23	2.0943803	4.72E-06	0.0016344	ASS1	-2.1961525	0.0002025	0.008577793
ADARB1	2.0827201	4.37E-06	0.0016283	EP300-AS1	-2.1958892	0.0002321	0.009244294
RGS7	2.0786262	0.00376529	0.0472823	SNTB2	-2.1857837	2.75E-06	0.001416803
RND3	2.0777207	5.38E-06	0.0016632	ANKRD32	-2.1834996	5.04E-05	0.004316979
ITFG2 /// LOC1	2.0735266	8.72E-07	0.0007766	FA2H	-2.1812306	0.0001758	0.007936543
INPP4B	2.0731824	0.00367287	0.0465278	PPP2R2B	-2.1791405	4.35E-06	0.001628344
CD274	2.0715278	0.00014682	0.007206	ACTC1	-2.1787055	0.0003282	0.011178768
MGLL	2.0678432	2.85E-05	0.0032612	RPS15A	-2.1777321	4.46E-05	0.00401486
SNHG7 /// SNO	2.0644309	1.00E-05	0.0021801	PRPS2	-2.1772542	3.40E-06	0.001463899
TPBG	2.0545452	2.56E-05	0.0030863	SPTLC2	-2.1693876	3.25E-06	0.001463805
LINC00511 ///	2.0523386	8.58E-06	0.0020227	CLGN	-2.1658733	1.53E-05	0.00245844
GADD45A	2.0462843	0.00011531	0.0064265	TNFRSF19	-2.1628984	0.0001269	0.006716488
IRAK2	2.0462045	0.00016932	0.007783	AGPAT3	-2.1627926	2.98E-06	0.001459329
HYI	2.0413393	3.81E-06	0.0015324	RP5-1092A3.4	-2.1621312	0.0003447	0.011444401
LOC100505812	2.0397736	4.25E-05	0.0039343	ESRRG	-2.1608497	6.49E-06	0.001758564
SERTAD4	2.0378274	1.07E-05	0.0021926	LOC10192968	-2.1603998	1.21E-05	0.002346199
ACOX2	2.0339001	0.00020107	0.0085619	SNX10	-2.1570102	5.28E-06	0.001640552
UBASH3B	2.0319461	0.00020185	0.0085685	DNAJC13	-2.1474501	4.58E-06	0.001634419
WFDC21P	2.0318694	2.16E-06	0.001273	SLC4A4	-2.1465842	0.0001754	0.00793088
ARMCX5	2.0280655	1.59E-06	0.0010721	SRD5A3	-2.1384974	4.46E-05	0.00401486
SH3RF2	2.0204646	2.35E-05	0.0029601	C1GALT1C1	-2.1367096	6.76E-07	0.000743678
ZFAND2A	2.0204426	2.09E-06	0.001273	FAM13C	-2.1349736	0.0003995	0.012295927
RYR2	2.0184601	0.00088538	0.0195825	PARP1	-2.1348768	8.97E-05	0.0056077
TNFAIP3	2.0151625	3.49E-05	0.0035739	ST6GALNAC1	-2.1337652	0.001189	0.023531362
FLJ32255	2.0114202	0.00025168	0.0097109	EFHD1	-2.1300553	2.93E-05	0.003330611
PLCB4	2.0025524	0.00089559	0.0197011	UROS	-2.1258945	2.20E-06	0.001272984
MBNL1-AS1	2.001887	0.0041854	0.0503713	METTL21A	-2.1230839	1.41E-05	0.00239658
CYTH1	2.0006364	4.21E-06	0.0016237	PCDH7	-2.1224575	0.0003234	0.011091747
SPINK1	1.9864264	1.53E-05	0.0024584	CCBE1	-2.1217293	0.0004211	0.01269201
PCDH9	1.9833333	2.65E-05	0.0031191	SCIN	-2.1190489	0.0001122	0.006345664
LINGO2	1.9802011	0.00150656	0.0271209	ZNF362	-2.1130007	8.15E-06	0.00199842
LRIG1	1.9792987	0.00013801	0.0069897	LOC254057	-2.1128894	9.16E-06	0.002090333
NEDD9	1.9774481	0.00032423	0.0111073	GLUL	-2.1128259	2.52E-06	0.001361825
LOC101928054	1.9735336	0.00026872	0.010029	APOL6	-2.1122207	5.91E-05	0.004582711
C6orf48	1.9710923	8.33E-06	0.0020188	PDE8B	-2.111891	3.80E-05	0.00368328
ZNF134	1.9709241	2.19E-05	0.0028761	RPARP-AS1	-2.1101714	5.59E-05	0.004519668
HORMAD1	1.9602026	2.69E-06	0.001404	NAGA	-2.1098593	1.34E-05	0.002364945
SMIM7	1.9521593	0.0002563	0.0098063	RAB11FIP2	-2.1084118	1.92E-05	0.002726849
CEP83	1.9476389	0.00173794	0.0295281	PCMTD1	-2.1033345	1.61E-05	0.002500365

SERPINB3	1.9461028	0.00349767	0.0450816	AMN1	-2.0875821	0.0001626	0.007605442
MAML3	1.9459239	0.00175506	0.0297008	NUDT12	-2.0834747	1.51E-05	0.002450781
ZSCAN12	1.9445461	2.26E-05	0.0028846	SREK1	-2.0825407	0.0002374	0.009343174
PARD3B	1.9442118	0.00013671	0.0069636	STRBP	-2.0784694	1.33E-05	0.002364945
MYO5B	1.9413875	3.67E-05	0.0036621	PCOLCE2	-2.0776274	4.90E-06	0.001634419
TIMM9	1.933651	3.04E-06	0.0014593	STARD4	-2.073173	5.66E-05	0.004543098
MAFB	1.9322655	0.00023602	0.0093346	RABL6	-2.0648536	0.000101	0.006019467
TAF1A	1.929314	0.0002195	0.0090158	PQLC3	-2.0619081	0.0002348	0.009309605
SAMD4A	1.9261592	0.00068956	0.0169826	DAB2 /// LOC	-2.0616477	0.0007119	0.01737736
SAA1 /// SAA2	1.9192316	0.00068911	0.0169826	ALDH3A1	-2.0609478	0.0035439	0.045473946
SNAI2	1.9176437	0.0001219	0.0066084	MIR4680 ///	-2.0597346	8.64E-06	0.002026699
RGS20	1.914161	0.00012858	0.0067467	HFE	-2.0596319	3.96E-05	0.003790056
MIR100HG	1.9123092	3.03E-05	0.0033787	ASPH	-2.0588152	7.46E-06	0.001940972
CDC42EP2	1.9122304	4.44E-05	0.0040103	ARHGAP5	-2.0585365	1.63E-05	0.00251402
TLE1	1.9117612	4.01E-05	0.0038042	FZD6	-2.0583506	2.10E-05	0.002840753
HDAC9	1.9108322	0.00092875	0.0201359	EIF4EBP2	-2.0564297	4.32E-06	0.001628344
DDIT3	1.9086179	4.93E-06	0.0016344	BRI3BP	-2.0544328	5.05E-06	0.001634419
SUPT3H	1.904583	1.44E-05	0.0024103	C1orf43	-2.0510602	2.26E-06	0.001272984
CDH13	1.9041548	7.67E-06	0.0019418	TMX4	-2.0438165	0.0003167	0.010946101
TAGLN	1.8973804	0.00243459	0.036448	TBC1D9	-2.0426784	1.29E-05	0.002364945
STX4	1.8947193	5.81E-06	0.0016903	LOC728819	-2.0396537	1.45E-05	0.002413015
HINT3	1.8899765	0.00092004	0.0200252	S100P	-2.0395925	9.45E-05	0.005806079
JUN	1.8874298	0.00014639	0.0071976	GLCE	-2.0391314	0.0001073	0.006215054
RASSF9	1.8873434	0.00049645	0.0140636	ZDHHC13	-2.0389318	8.99E-06	0.002064851
KCNJ12 /// KCN	1.8828649	0.00059072	0.0157075	TCFL5	-2.0362405	1.16E-06	0.000869352
LAMB3	1.878066	5.50E-05	0.0045083	RHOQ	-2.0350344	1.26E-05	0.002364945
MAP3K7CL	1.8775528	0.00105222	0.0218658	CDS1	-2.0336025	3.16E-05	0.003451002
STC2	1.876473	0.0003514	0.0115599	MAN2A1	-2.0329723	6.83E-05	0.004953055
LIAS	1.8722378	5.49E-06	0.0016721	Sep-06	-2.0322924	6.38E-06	0.001758564
GPATCH4	1.8703306	8.87E-06	0.0020553	CAST	-2.0260172	0.0006836	0.016904072
NAT8L	1.8667124	5.93E-05	0.0045827	PDE1A	-2.02467	1.45E-05	0.002413015
TNFRSF21	1.8644807	0.00039804	0.0122959	AGO4	-2.0218847	1.65E-05	0.00252015
RAD18	1.8637996	0.0001359	0.0069444	CEP120	-2.0185903	9.59E-05	0.005827684
TMEM161B-AS	1.8636865	3.25E-06	0.0014638	APAF1	-2.0182092	7.82E-05	0.005273797
HSBP1L1	1.8633918	4.83E-06	0.0016344	ABRACL	-2.0126349	4.10E-05	0.003869146
CCND1	1.8627837	0.00397225	0.0488582	CTNNB1	-2.0094988	1.59E-05	0.002493351
TXNRD2	1.860262	3.27E-06	0.0014638	SLC7A11	-2.0082378	7.03E-05	0.00500775
DTX4	1.8518124	0.0003493	0.0115326	PSCA	-2.0072005	3.21E-05	0.003466607
RBM14	1.8516701	5.01E-06	0.0016344	ID2	-2.0045526	0.0022292	0.034430278
NDUFA7	1.850151	6.24E-05	0.0047312	SEC24D	-2.0028907	0.0012621	0.024327598
HSF2BP	1.844118	8.00E-06	0.0019879	ELOVL6	-2.0001849	2.81E-05	0.00325287
LMO7	1.8435168	0.00048038	0.0137224	CRIPT	-1.9983002	2.06E-05	0.002807439
CYP1A1	1.8434946	0.00151694	0.0272466	HN1L	-1.9957601	2.61E-05	0.003092269
EGFR	1.8433839	0.00026116	0.0099007	G6PD	-1.9954264	2.20E-05	0.002876666
SRR	1.8422776	1.65E-05	0.0025257	HOXA13	-1.9938236	1.59E-05	0.002493351
PDAP1	1.8418156	1.10E-05	0.0022097	KREMEN1	-1.992307	0.0002942	0.010476973
YRDC	1.8411836	0.00049889	0.0140893	EPHA7	-1.990051	0.0004266	0.012823513
SLC35F3	1.8399303	0.00064838	0.0165345	MIS12	-1.9889548	0.0001173	0.006468963
FJX1	1.8387447	0.00020688	0.0087276	PLEKHA7	-1.9865365	0.000142	0.007077297
LOX	1.837318	0.00021575	0.0089154	SESTD1	-1.9858413	0.0001932	0.008471931

NPAS2	1.8365229	0.0002969	0.0105393	OLFM1	-1.9853904	0.00012	0.006580369
SNAP25	1.8358888	0.00012533	0.0066657	ELF3	-1.984159	0.001008	0.021240336
DYX1C1	1.8352212	0.00014815	0.0072452	ATG7	-1.9832522	2.04E-05	0.002807439
MRPL1	1.8348336	2.58E-05	0.0030923	ARID5B	-1.9822918	1.50E-05	0.002442656
PIGL	1.8347584	0.00017492	0.007928	CDKN2C	-1.9818085	0.0001453	0.007178896
C12orf60	1.8334985	0.00026961	0.0100345	FBXO9	-1.9792667	1.90E-05	0.002722592
ASAH2B	1.8315539	8.75E-05	0.0055879	DAB2	-1.9759088	0.0001859	0.008243515
SH3RF3	1.8306075	1.05E-05	0.0021926	HIBADH	-1.9753401	1.28E-05	0.002364945
TGM2	1.827883	2.57E-05	0.0030917	OBSL1	-1.9745486	0.002553	0.037419645
EFNB2	1.824086	0.00010572	0.0062085	TGDS	-1.9728817	0.0017423	0.02955667
MSANTD3-TME	1.8233763	0.00224653	0.0346583	LOC1019282	-1.9681336	3.31E-06	0.001463805
BMP2	1.8232646	0.00107276	0.0220666	DUSP2	-1.9656074	0.0001174	0.006468963
LOC101929787	1.8231917	0.00039961	0.0122959	TDRD7	-1.9648403	7.52E-05	0.00517509
ERICH1	1.8231158	0.0001025	0.0060852	GPRC5A	-1.9640881	5.45E-06	0.001672145
GKAP1	1.820689	0.00022638	0.0091519	DNASE2	-1.9636681	0.0001103	0.006302715
GART	1.8201481	0.00090643	0.019847	SC5D	-1.963023	1.70E-05	0.002572067
GFPT2	1.8185272	3.84E-05	0.0037108	ABCC6 /// AB	-1.9590998	9.99E-05	0.005983356
BCL2	1.8184176	0.00066608	0.0166533	VEZT	-1.9576643	0.0002529	0.00973635
THAP9-AS1	1.8183214	0.00049424	0.014023	TMEM50B	-1.9563884	4.30E-05	0.003965634
LOC100996760	1.8173845	0.00024025	0.0094094	PLAC1	-1.9524028	1.67E-06	0.001087373
RAB27B	1.813419	0.00427185	0.0509968	DNAJC6	-1.9508951	0.0001339	0.006874783
GBP1	1.8103654	0.00065759	0.0166065	RHOBTB1	-1.950174	6.14E-05	0.004692297
LOC100507507	1.8086048	6.81E-05	0.0049531	RNASEL	-1.9498365	0.0003007	0.010600566
ISM1	1.8066544	0.00172701	0.0294432	GALNT12	-1.9480828	0.0018725	0.030901273
NUFIP1	1.8061801	3.76E-05	0.0036833	XXYLT1	-1.9479654	1.40E-05	0.002388507
JAG1	1.8038927	0.00055789	0.0151529	DHCR7	-1.9464548	2.15E-05	0.002876143
MAFF	1.8033069	0.00053757	0.0148507	NT5DC1	-1.9437288	1.15E-05	0.002261862
SNHG1 /// SNC	1.802178	6.24E-05	0.0047312	FAM81A	-1.9429453	5.07E-06	0.001634419
ENAH	1.8019572	8.66E-05	0.0055674	ERGIC2	-1.9426864	1.57E-05	0.002478152
SYNCRIP	1.7989661	3.03E-05	0.0033787	NUCKS1	-1.9419012	5.65E-06	0.001677803
RAB38	1.7967799	0.00085802	0.0192917	ZNF652	-1.940188	5.77E-05	0.004555177
KLF2	1.7964259	5.48E-05	0.0045083	GPR107	-1.9396762	5.90E-05	0.004582711
TNFRSF12A	1.7948761	4.13E-05	0.0038706	HMGN5	-1.9392509	2.95E-05	0.003330611
RPP14	1.7942611	0.00060068	0.0158581	CTB-119C2.1	-1.9370934	3.21E-05	0.003466607
ADAT3 /// SCAI	1.7938269	0.0001027	0.0060903	SUMF1	-1.93643	2.80E-05	0.00325287
INO80B /// INC	1.7910707	8.04E-06	0.0019879	HSD17B7	-1.9305031	0.0001664	0.00772502
GPR158	1.7906764	1.80E-05	0.0026469	CCDC34	-1.9296452	0.0025275	0.037227988
BCL2L1	1.7904795	4.79E-05	0.0041841	PSMD11	-1.9283023	1.75E-05	0.002613747
LOC101928955	1.7896616	0.00219838	0.0342148	PPA2	-1.9247053	0.0001141	0.00639562
TPM1	1.7865821	0.00033212	0.0112438	CTSB	-1.9223651	7.19E-05	0.005073395
GNG4	1.7854217	4.52E-05	0.0040314	LHX2	-1.920923	0.0002753	0.010127117
ZBTB26	1.7853666	6.31E-06	0.0017515	PCYOX1L	-1.9202464	3.20E-05	0.003461684
PMAIP1	1.7848972	3.95E-05	0.0037901	NPHP3	-1.9200829	3.14E-05	0.003438259
GAS5 /// SNOR	1.7843872	1.01E-05	0.0021823	UBE4B	-1.9174241	8.96E-05	0.0056077
GATA6	1.7843857	0.00083743	0.0190647	KAT2B	-1.9138291	1.91E-05	0.002726849
GJB5	1.7841373	0.00065133	0.0165502	ATF7IP2	-1.9096398	4.41E-06	0.001628344
PRRT3-AS1	1.7841196	6.00E-06	0.0017253	SLC35G1	-1.908687	0.000317	0.01094937
HNMT	1.7841023	5.89E-05	0.0045827	WDR37	-1.9073414	0.0012464	0.024166422
GPR37 /// SEL1	1.780785	0.00042769	0.0128342	PDK3	-1.9060504	4.49E-05	0.004027564
LOC645166 ///	1.7806128	8.49E-05	0.0054912	SNN	-1.9030158	0.0009125	0.019925295

LOC101930150	1.7805705	0.00228598	0.035069	SNHG4	-1.9025403	6.12E-05	0.004691516
FLJ31306	1.7801615	7.43E-05	0.005139	FTL	-1.9024762	6.97E-05	0.004987622
ZNHIT6	1.7780693	0.00025978	0.009885	FAM213A	-1.9011714	2.81E-05	0.00325352
PPP1R15A	1.7768377	5.42E-05	0.0044917	COX7B	-1.8999597	0.0001261	0.00669642
RUNX2	1.7767638	0.0023373	0.0355373	ANKRD20A12	-1.899413	0.0009109	0.019897546
FAIM3	1.7764549	0.00327349	0.0433077	CHD6	-1.8991253	2.92E-05	0.003330611
PRKCE	1.7746417	0.00073393	0.0175999	ZMYM6 /// Z	-1.8989731	3.41E-05	0.003541674
NT5E	1.7741454	0.00027278	0.0101044	ENDOD1	-1.893027	0.0001348	0.006912288
SPATA17	1.7712849	0.0004636	0.0134476	PCK2	-1.8918544	0.0001068	0.006215054
MAD1L1	1.7707106	0.00057678	0.0154966	PLOD1	-1.8902647	3.69E-05	0.003662068
PCSK1	1.7680671	0.00408948	0.0497204	C14orf1	-1.8893785	1.30E-05	0.002364945
SLC2A3	1.767462	0.00143872	0.0264056	PRKACA	-1.8888849	8.52E-06	0.002022734
ZNF689	1.7664938	2.43E-05	0.0030094	TUBGCP5	-1.8878956	3.78E-05	0.00368328
LIF	1.7655014	1.04E-05	0.0021877	ANXA9	-1.8872745	0.0014598	0.026639979
ARL6	1.765138	0.00047291	0.0136157	MBNL3	-1.8864643	1.24E-05	0.002364945
MTHFD2L	1.7649626	0.00054135	0.0148909	SNX5	-1.8854441	1.38E-05	0.002388507
PTGS2	1.7645508	0.00346171	0.0448398	GALNT2	-1.8812642	3.77E-05	0.00368328
STAMBPL1	1.7638494	0.00096019	0.0206038	RPS6KA5	-1.8805323	0.0004061	0.012438224
C1orf109	1.7638175	0.00087519	0.019443	IFT80	-1.8789	3.76E-05	0.00368328
Mar-04	1.7625353	7.06E-05	0.0050209	SELO	-1.8783419	4.93E-06	0.001634419
PATL1	1.7618657	1.70E-05	0.0025721	TMEM117	-1.8738517	5.60E-06	0.001673101
CYR61	1.7618092	0.00338565	0.0441896	SGMS2	-1.8736833	0.0004199	0.012669963
PDGFC	1.7606998	0.00057433	0.0154501	CADPS2	-1.8709783	0.0002661	0.00997704
MRPL52	1.7589765	3.08E-05	0.0034191	PRKX	-1.8686536	2.06E-05	0.002807439
CAMSAP2	1.7581084	7.64E-05	0.0052116	LOC1019298!	-1.8685423	0.0012599	0.02431023
ANXA3	1.7545807	7.52E-05	0.0051751	OMA1	-1.8669217	5.75E-05	0.004555177
SLC16A9	1.7525432	0.0005266	0.0146898	LINC01003	-1.8668989	0.000128	0.006721761
NFAT5	1.7516119	0.00061127	0.0159986	TRIQK	-1.8640198	2.60E-05	0.003092269
BTG3	1.7504861	5.45E-05	0.0045018	SGPP1	-1.8634999	5.65E-05	0.004543098
SLC16A14	1.7478579	0.0002214	0.0090462	SESN3	-1.8616218	0.0030337	0.041342935
SLCO1B3	1.7472433	0.00041973	0.01267	SDF2L1	-1.8616206	1.34E-05	0.002364945
OTUD1	1.7468732	5.40E-05	0.0044917	PI4K2B /// SE	-1.8614477	9.21E-06	0.002090333
CARD8	1.7467096	0.00023847	0.0093598	NUTF2 /// NU	-1.85927	3.97E-05	0.003797455
CCDC132	1.7454899	0.00165446	0.0287456	C6orf147 ///	-1.8588377	0.0001995	0.008531215
BACH2	1.7434383	0.0015116	0.0271865	LINC00674	-1.8585324	1.87E-05	0.002703229
IFIT2	1.7426818	6.14E-05	0.0046915	TRIM37	-1.8562896	5.37E-05	0.004491723
SNHG8 /// SNO	1.7420473	2.23E-05	0.0028846	ADD3	-1.8560932	6.41E-06	0.001758564
RELB	1.7398705	0.00040815	0.0124528	EML1	-1.8552613	0.0004283	0.01284545
MCL1	1.7392307	0.00015614	0.0074559	KIAA0101	-1.8537595	2.44E-05	0.003009441
DCLRE1A	1.7387427	9.78E-05	0.0058904	FBXO22	-1.8532009	0.0005325	0.014779292
PPP1R14C	1.7382394	0.00040839	0.0124534	TUSC3	-1.8433462	8.34E-06	0.002018774
TGFB2	1.7361422	9.41E-06	0.002127	RPS27L	-1.8427095	1.56E-05	0.002475249
CUL4A	1.7359231	4.31E-05	0.0039666	FGFBP1	-1.8422804	0.001107	0.022555047
CHIC2	1.7355774	0.00034166	0.0113957	CCNG2	-1.8417152	0.0030384	0.041354324
CC2D2A	1.7345214	0.00069097	0.0170099	RIMKLB	-1.8402277	3.43E-05	0.003547985
OSMR	1.7328171	0.00045186	0.0132397	LOC1019281	-1.8393603	0.0008736	0.019424668
ACVR1	1.732413	0.00023701	0.0093432	SSX2IP	-1.8389153	6.07E-06	0.001728221
C12orf29	1.7320925	0.00026201	0.0099007	HEY1	-1.8375077	0.0004634	0.013447616
DTWD1	1.7307129	2.41E-05	0.0029981	JPH1	-1.8355026	1.81E-05	0.002647264
SH2D5	1.7303238	0.00016935	0.007783	LOC439911	-1.8353938	8.54E-06	0.002022734

CPED1	1.7279564	0.00215287	0.0337659	TMEM37	-1.8352566	2.19E-05	0.002876143
AUP1	1.7260412	9.95E-06	0.0021766	KIAA0232	-1.8341296	3.92E-05	0.003778627
DIRAS3	1.7253251	0.00335919	0.043959	AIF1L	-1.8315878	0.0003674	0.011823763
TJP2	1.7239971	0.00423663	0.0507537	DBT	-1.8299693	0.0019059	0.031261308
SCNM1 /// TNF	1.7223758	1.19E-05	0.0023163	LMO1	-1.8285515	0.0019764	0.031990033
CASP4	1.7211686	0.00017767	0.0079951	LOC1019289(	-1.8280491	4.12E-05	0.003870577
KIFC3	1.7200568	3.24E-05	0.0034746	GTF2IRD2	-1.8271434	2.00E-05	0.002807439
PSTPIP2	1.7198858	0.00022171	0.0090462	UNKL	-1.8271137	0.0004612	0.013406219
HKR1	1.7189872	0.00015287	0.0073903	NADSYN1	-1.8264907	1.35E-05	0.002364945
BICD1	1.7175647	0.00013531	0.00693	NIPAL2	-1.8259451	8.89E-05	0.005601897
HOXA3	1.716753	6.13E-05	0.0046915	MBTD1	-1.82574	0.0015718	0.027808675
RP11-350F4.2	1.7160229	5.03E-05	0.004317	HOOK1	-1.8244689	1.66E-05	0.002526824
ZNF26	1.7155735	0.00012244	0.0066087	ZNF333	-1.823572	1.69E-05	0.002572067
C2orf49	1.7138329	5.72E-05	0.0045511	TMEM183A /	-1.8210584	9.50E-06	0.002131095
ZSCAN21	1.7135017	2.17E-05	0.0028761	SLC2A13	-1.8196309	2.18E-05	0.002876143
SARS	1.7132102	0.00044152	0.013031	MIR7110 ///	-1.8192354	1.38E-05	0.002388507
GABPB1-AS1	1.7113088	0.00013835	0.0069975	ENOX2	-1.8190665	0.0001863	0.008254451
C19orf48 /// SN	1.7097535	1.52E-05	0.0024584	PRKD3	-1.8162205	6.16E-05	0.004695351
TNIP1	1.7092204	8.80E-05	0.0055893	DLEU2	-1.8161258	0.0019894	0.032124125
FLCN	1.7078267	0.00038228	0.0120469	LOC1019281(	-1.8160724	0.0023801	0.03588915
FHL2	1.7074179	8.87E-05	0.0056019	DECR1	-1.8156878	2.44E-05	0.003009441
USE1	1.7069904	8.15E-06	0.0019984	DZIP1	-1.8150571	1.92E-05	0.002726849
MYL9	1.7066429	0.00084904	0.0191982	CPT2	-1.8136849	1.11E-05	0.002215913
BCAR3	1.705529	0.00062995	0.0162616	ATP7A	-1.8124816	2.61E-05	0.003093727
RRAD	1.7034161	0.0018027	0.0301784	SETMAR	-1.8121806	1.01E-05	0.002182283
ZNF655	1.7032445	0.00012453	0.0066622	DHFR	-1.8111372	7.81E-06	0.001966762
NDUFAF4	1.7019582	0.00105459	0.0218847	PAPSS2	-1.8110751	0.0002653	0.009976992
TWIST2	1.701947	0.00041716	0.012615	PCYOX1	-1.8107066	0.0003418	0.011395702
ZNF383	1.6988338	0.00020047	0.0085562	CCM2	-1.8086933	4.34E-05	0.003973143
LOC101928076	1.6977914	4.09E-05	0.0038661	C3orf80	-1.8076728	0.0001077	0.006231104
C11orf1	1.6976819	5.96E-05	0.0045974	SLC26A2	-1.8073172	0.0011874	0.023531362
ALDH8A1	1.6955341	0.00015199	0.0073607	HR	-1.8073145	0.0023104	0.035314905
TESC	1.6947734	0.00280642	0.0396121	RECK	-1.8041294	6.05E-05	0.004650023
LOC100130938	1.6923484	0.00398173	0.0489326	BACE1	-1.8022648	0.0002347	0.009309605
SLC39A9	1.6921842	0.00012088	0.006583	ATP9A	-1.7999497	5.68E-05	0.004543098
ZNF311	1.6919549	0.00048702	0.0138614	PRTG	-1.7994975	0.0001378	0.006989707
FN1	1.691436	7.18E-05	0.0050734	MTFMT	-1.7982451	2.18E-05	0.002876143
RALGAPA1	1.6911101	0.00158882	0.0279661	PEBP1	-1.7969561	1.53E-05	0.00245844
RP11-247L20.4	1.6881041	0.00043896	0.0129887	AFF1	-1.796524	1.26E-05	0.002364945
H1F0	1.6874719	0.00026918	0.0100324	TMEM144	-1.7959334	0.0006895	0.016982622
UST	1.6870226	0.00131166	0.0248673	PBLD	-1.7958798	9.34E-05	0.005768736
NRP1	1.6867769	0.00085334	0.0192552	LOC1005068:	-1.7955148	0.0011391	0.022837946
DNM3	1.6865469	0.00384573	0.0479619	ADAM1A ///	-1.7943528	0.0003667	0.011808174
FGF2	1.6856567	6.77E-05	0.0049531	SLC39A8	-1.7941094	4.23E-05	0.003929839
THAP1	1.6856233	8.95E-05	0.0056077	GBP2	-1.7937734	0.0016855	0.029025774
GLIPR1	1.6852583	0.00073602	0.0176118	VAMP1	-1.7929176	0.0001809	0.008067732
LRRC7	1.6844976	0.00050107	0.0141434	LMF1	-1.7921076	0.0015836	0.027929629
CXorf40A /// C	1.6834825	7.12E-05	0.0050469	CES1 /// LOC	-1.7911774	6.98E-05	0.004987622
IL32	1.6833964	0.00014228	0.0070773	MRE11A	-1.7907979	0.0006012	0.015863952
HRH1	1.6832591	0.00044213	0.0130386	IQGAP2	-1.7896331	0.0006458	0.016492809

AJUBA	1.682602	0.00046667	0.0135216	UGT8	-1.7883703	0.0021175	0.03346214
CEBPD	1.6820394	0.00013187	0.0068254	GPX8	-1.7878444	2.68E-05	0.003132594
PRKAB1	1.6818464	0.00023669	0.0093432	BBS2	-1.7863677	2.19E-05	0.002876143
NFIL3	1.6800919	0.00023675	0.0093432	ZFYVE16	-1.7858685	0.0010634	0.021948866
SMG5	1.679861	3.10E-05	0.0034191	BTN3A3	-1.7844171	4.29E-05	0.003965634
ZCCHC10	1.6795653	0.00037888	0.0120069	MTCH2	-1.7837241	7.16E-06	0.001882948
THSD4	1.6790931	0.00326324	0.0432527	TDRKH	-1.7833881	4.14E-05	0.003877503
SLIT2	1.6779664	0.00038939	0.0121672	ICK	-1.7830666	0.000245	0.009540573
FAM60A	1.6779345	3.98E-05	0.0037975	CD59	-1.7827109	2.65E-05	0.003119135
PLGLB1 /// PLG	1.6758088	0.0002505	0.0096791	CNTNAP3 ///	-1.7817252	6.74E-05	0.004949574
CENPBD1	1.6754257	3.04E-05	0.0033844	PDIA3	-1.7812748	0.0003162	0.010941365
LOC101929147	1.6718078	8.46E-05	0.0054912	FBXO3	-1.7812213	3.11E-05	0.003424078
LOC102724257	1.6714116	0.00027728	0.0101405	PTPRJ	-1.7795741	2.27E-05	0.002884643
LINC01138	1.6711757	0.00012745	0.0067169	CEP70	-1.7794426	1.60E-05	0.002493351
AKIRIN2	1.6704863	0.0014025	0.0259236	GAS6-AS1	-1.7776325	0.0012175	0.023838794
DCAF4L1	1.6693145	0.00138324	0.0257065	PTGFRN	-1.7770951	2.58E-05	0.003092269
DLEU1	1.6676274	3.41E-05	0.003542	COL18A1	-1.777075	3.40E-05	0.003541674
AOX1	1.6671157	0.00040705	0.0124382	HELLS	-1.7767088	0.00059	0.015707504
SIPA1L2	1.6671155	0.00103357	0.0215937	BBIP1	-1.7759884	8.12E-05	0.005381287
RPS19BP1	1.6661003	9.12E-05	0.0056541	MPHOSPH9	-1.7743331	4.61E-05	0.004085711
PDGFB	1.6659187	1.54E-05	0.0024661	SPINK5	-1.7739826	0.0017155	0.029301769
ARHGEF18	1.6638123	5.03E-05	0.004317	EVL	-1.7739088	9.66E-06	0.002147789
CRBN	1.6637922	1.26E-05	0.0023649	EMB	-1.7739049	0.0001454	0.007178896
CCDC68	1.6634822	0.00065742	0.0166065	MAPK1	-1.7732729	8.78E-05	0.005589333
MBP	1.6623475	0.0007483	0.0178272	NIPSNAP3A	-1.7717303	0.0002598	0.00988499
CSNK1E	1.6618642	3.00E-05	0.0033581	IPO8	-1.7708858	0.0001456	0.007178896
RGS5	1.6616144	9.09E-05	0.0056489	SERPINB9P1	-1.7701873	0.0005817	0.015565822
ZNF329	1.6595071	0.00134693	0.0252983	TMEM56	-1.7692643	4.03E-05	0.003815031
TROVE2	1.6581544	0.00045885	0.0133767	L1CAM	-1.7688683	0.0001252	0.006665686
BAG5	1.6567946	2.25E-05	0.0028846	ITGA6	-1.7682665	0.0027293	0.038931458
WDR72	1.6563018	0.00048609	0.0138423	SPG20	-1.7672448	0.0012166	0.023838202
NGRN	1.6560989	1.77E-05	0.002618	NEK7	-1.7662814	0.0001029	0.006090806
AC002310.12 /	1.6556655	0.00023095	0.0092102	HGSNAT	-1.7661957	0.000469	0.013573293
FIGN	1.6548923	0.00123018	0.0239786	KLRC4-KLRK1	-1.7661934	0.0023139	0.035341431
PQBP1	1.6548631	2.09E-05	0.0028293	BTF3L4	-1.7659688	0.000353	0.011574583
HEIH	1.654342	1.56E-05	0.0024752	ITPR1	-1.7652936	0.002639	0.038211293
STARD13	1.6535971	0.00032218	0.0110744	SH3BP5	-1.7649316	6.45E-05	0.004838351
DNAH14	1.6534751	0.00016999	0.0077841	TMEM106B	-1.7648452	0.000459	0.013376681
EPS15L1	1.6518002	7.95E-05	0.0053153	BD495725	-1.7645127	0.0011026	0.022485237
ITGA2	1.6503494	0.00300854	0.0411791	DPY19L1	-1.7644656	5.05E-05	0.004316979
MAML2	1.6499058	0.00187023	0.0308833	ENOSF1	-1.7635955	2.20E-05	0.002876143
GEM	1.6493412	0.00222916	0.0344303	USP46	-1.7629339	3.70E-05	0.003662068
RYBP	1.6489647	3.60E-05	0.0036339	OAT	-1.7626251	8.82E-05	0.005589584
MIR3682	1.6482038	0.00401074	0.0491565	CBX3	-1.7622805	0.0007208	0.017460363
ARMCX6	1.6478566	4.00E-05	0.0038029	LOC1009967[illegible]	-1.7601421	0.001251	0.024219509
FAM92A1	1.6460453	0.0001222	0.0066084	USP10	-1.7586583	0.0002983	0.010543002
LOC102723721	1.6459957	0.00014721	0.0072186	GNE	-1.757397	1.64E-05	0.00251793
LOC284023	1.645991	0.00028363	0.0102166	Sep-07	-1.7561806	0.0005269	0.014689997
LATS2	1.6458184	0.00042331	0.0127517	VPS13C	-1.755917	0.0010526	0.021865751
DDX19B	1.6457691	0.00012087	0.006583	BOLA3	-1.7551029	0.0008662	0.019360038

BOLA2 /// LOC1	1.6455171	0.00011194	0.0063422	TMX1	-1.7545428	0.000822	0.018922407
ST7L	1.6443142	0.00011126	0.0063272	CTTNBP2	-1.7541971	0.0002124	0.008839765
CFH	1.6443053	0.00026514	0.009977	LRP4	-1.7540791	0.0014456	0.026478842
IFIT1	1.6438287	0.0015197	0.027251	DDHD2	-1.7532796	0.0002407	0.009419935
ADORA2B	1.6424229	0.00031507	0.0109373	ALDH2	-1.7527184	0.0004266	0.012823513
SEC14L1	1.64167	0.00233209	0.0354875	DGKQ	-1.752617	1.37E-05	0.002388507
LOC374443	1.6408868	0.00087153	0.0194019	NBPF20	-1.752367	9.76E-05	0.005890001
TRIB1	1.6407971	2.19E-05	0.0028761	RPL35A	-1.7517182	6.39E-05	0.004817108
MRPS31	1.6403151	4.56E-05	0.0040474	LOC1005061(	-1.7513873	0.0001721	0.007841615
FBXO28	1.6396011	0.00019148	0.0084225	LUM	-1.7506852	0.0008422	0.019090198
FAM120C	1.639598	8.13E-05	0.0053813	LITAF	-1.750135	0.0003263	0.011158301
MSL2	1.6390746	2.80E-05	0.0032529	FKBP5	-1.749615	1.24E-05	0.002364945
LOC100506990	1.638195	0.00026918	0.0100324	TMED4	-1.7492675	6.49E-05	0.004850755
MSRB3	1.6381325	0.00191255	0.0313199	MARCKSL1	-1.7471293	2.02E-05	0.002807439
CSF2RA	1.637709	0.00160435	0.0281265	SESN1	-1.7469264	0.0002061	0.008702401
OSBPL3	1.6374892	0.0006777	0.0168558	RABGAP1L	-1.7467027	0.0002099	0.008760619
ARL4C	1.6360885	0.00366454	0.0464439	COL1A1	-1.7456182	0.0013332	0.02511848
SLC16A6	1.6350304	0.00289014	0.0403211	TLR3	-1.7449123	0.0013797	0.025657504
ZFAS1	1.6350243	1.76E-05	0.002618	NUDT5	-1.7447411	1.26E-05	0.002364945
STEAP1	1.6344027	0.00104179	0.0217074	TXNDC16	-1.744195	0.0001172	0.006468963
FNDC3B /// LO(	1.6338159	0.0003585	0.0116742	CDON	-1.7441173	3.48E-05	0.00357392
BCDIN3D	1.6336647	2.95E-05	0.0033306	RP11-445H22	-1.7439973	4.56E-05	0.004047384
CLCN4	1.6315084	0.00139399	0.025836	RBBP4	-1.7435804	4.33E-05	0.003972406
RP11-499E18.1	1.6308454	0.00012104	0.0065849	TMEM187	-1.7425289	0.0005777	0.015512469
SNHG5 /// SNO	1.6304406	7.46E-05	0.0051461	PREX1	-1.7421446	0.0002838	0.010216631
PLAUR	1.6299049	0.00018041	0.0080589	ALDH9A1	-1.7421383	9.91E-06	0.002176613
SLC16A1-AS1	1.6285899	9.00E-05	0.0056149	GCH1	-1.7420101	4.51E-05	0.004031448
SGK223	1.6285013	0.00026205	0.0099007	ZNF627	-1.7419738	0.0006152	0.016041275
DNAJB6	1.6280831	0.00086235	0.0192917	SEL1L	-1.7403013	0.0001517	0.007355031
CYSRT1	1.6264107	0.0023646	0.0357719	ANKRD46	-1.7397815	7.84E-05	0.005273797
MED10	1.6257944	0.00012777	0.0067169	FGFR2	-1.7393609	0.0007535	0.017907631
BC062763	1.625161	0.00019077	0.0084097	PCBP2	-1.7392104	3.77E-05	0.00368328
FAM126A	1.6249836	4.88E-05	0.0042382	RPL13A /// SN	-1.7391992	3.35E-05	0.003526393
LHX4	1.6249283	0.00040721	0.0124382	GTF2H1	-1.7368495	0.0004678	0.013548008
IFIT3	1.6236987	0.0004333	0.012903	SLC30A9	-1.736431	0.0015637	0.027732951
KIN	1.6224804	0.0003599	0.011692	CSTF3	-1.7364061	0.0001245	0.006662219
DGAT2	1.6220883	0.00038109	0.0120313	MED13L	-1.7360433	6.57E-05	0.004889286
EMP3	1.6209199	0.00015077	0.007329	MINK1	-1.7356276	1.06E-05	0.002192581
NCR3LG1 /// RI	1.6206879	5.55E-05	0.004509	DQ597730 //	-1.7347893	0.000794	0.01852135
DPCD	1.619143	0.000164	0.007657	TPGS2	-1.7330751	0.0002142	0.008887581
STAG3L2 /// ST	1.6188877	0.00095641	0.0205676	SOAT1	-1.7328673	0.0003367	0.011338601
VPS37A	1.6182431	0.00092625	0.0201167	CAND1	-1.7321235	0.0003615	0.011695514
SIX2	1.617398	0.00012346	0.0066308	EMP2	-1.7320126	0.0012139	0.023806163
CHST11	1.6173306	0.00097463	0.0208156	GXYLT1	-1.731148	0.0028257	0.03970534
TUFT1	1.6171129	0.00073043	0.0175699	TMEM14A	-1.730631	7.27E-05	0.005090632
NOG	1.6168593	0.00341053	0.0444084	SAMD11	-1.7292524	0.0015592	0.027688158
MRPS22	1.6166494	1.95E-05	0.0027567	SULF2	-1.7275495	4.42E-05	0.003998859
FAM216A	1.6159588	4.81E-05	0.0041851	SUSD2	-1.7257853	3.46E-05	0.00357392
IRF2BP2	1.6154582	5.28E-05	0.0044445	AF520793	-1.7214192	0.0014744	0.026772861
HERC5	1.6146282	0.00037282	0.0119066	G3BP2	-1.7198813	2.39E-05	0.002987084

TLDC1	1.6145846	4.38E-05	0.0039882	IKZF4	-1.7198232	2.30E-05	0.002906845
GPATCH2	1.6139729	0.00220309	0.0342589	DNAJC10	-1.7192388	1.58E-05	0.002483128
GPC5	1.6131462	0.0014962	0.0270072	LAMP2	-1.7189169	4.50E-05	0.004027564
TRIM8	1.6112259	0.00010849	0.0062562	CXXC5	-1.7175048	2.26E-05	0.002884643
TMOD3	1.6111738	0.00249125	0.036943	GNPTAB	-1.7172265	0.0002196	0.009015824
ZNF300	1.6103965	0.00279082	0.0394591	LPIN1	-1.7171492	0.0004699	0.013573293
ANKRD28	1.6101772	0.00034988	0.0115374	MANEA	-1.7170198	8.15E-05	0.005381287
RALGAPA1 /// I	1.6098456	0.00036059	0.0116941	RP11-649A18	-1.7164289	7.37E-05	0.005125028
SETD7	1.6089086	0.00083828	0.0190652	ZNF385B	-1.7159994	0.0002646	0.009968763
DUS3L	1.6083635	0.00011383	0.0063956	UNC5D	-1.7155903	0.0003073	0.010768791
XRCC4	1.6075229	0.00122568	0.0239105	MED31	-1.7153431	0.0020557	0.032767703
CEP41	1.6066031	5.34E-05	0.0044857	SNRNP70	-1.7142163	2.49E-05	0.003037316
EIF1	1.6065199	3.25E-05	0.0034746	PGAP1	-1.7141692	0.0016045	0.028126499
CDC42EP3	1.6065049	0.00202435	0.0324285	SLC16A1	-1.7134139	3.68E-05	0.003662068
BATF3	1.6046128	6.41E-05	0.0048264	TRAPPC10	-1.7123362	0.000201	0.008561898
LOC100506844	1.6043816	8.15E-05	0.0053813	CHMP3 /// RI	-1.7122512	1.47E-05	0.002418661
CYSLTR1	1.6036598	0.00081849	0.0188742	NR2F1	-1.7116461	0.0001574	0.007478699
CCNB1IP1	1.6035638	5.85E-05	0.0045827	FAM122B	-1.7110296	0.0002759	0.010127117
CSNK1A1	1.6032924	4.20E-05	0.0039029	CCNG1	-1.7091621	3.05E-05	0.003384387
BRD2	1.6032433	3.54E-05	0.0036017	BCLAF1	-1.7083972	6.90E-05	0.00496591
RAD1	1.6025528	3.40E-05	0.0035417	RBM3	-1.7082396	0.0003543	0.011587224
DNAJC17	1.601362	6.74E-05	0.0049496	CRABP2	-1.7066678	0.0034996	0.045082181
SGK494	1.6011761	0.00328054	0.0433559	PAK1	-1.7060412	0.000382	0.012046946
EVA1A	1.6011433	0.00088029	0.0195175	HSPBAP1	-1.7045896	0.0002162	0.008915418
MRPS30	1.6004995	4.70E-05	0.004141	SCNN1A	-1.7043867	0.0036154	0.046066705
LOC100630923	1.5991414	0.00041484	0.0125727	NEURL1B	-1.7042742	0.0006573	0.016606486
SMCHD1	1.5980424	0.002145	0.0336963	BPHL	-1.7035235	0.0001709	0.00781726
HERC3 /// LOC1	1.5979776	0.00416962	0.0502592	CLIC3	-1.7029069	0.0007116	0.017375903
BAD	1.5979393	0.00022973	0.0091783	GALNT6	-1.7014637	5.71E-05	0.004551083
MLH3	1.5977786	3.25E-05	0.0034746	JDP2	-1.700482	0.0003276	0.011165662
LINC01128	1.5976109	0.00333986	0.0438052	SLC35E2 /// S	-1.7004242	0.000114	0.00639562
IDS	1.5973176	5.89E-05	0.0045827	NELL2	-1.7003565	0.0006305	0.016261635
NUDT6	1.5966953	0.00040988	0.0124778	FGD6	-1.6994084	0.000561	0.015207124
HES1	1.5955724	0.00019338	0.0084719	SLFN5	-1.6993355	0.0007234	0.017489048
DYNLL1-AS1	1.5951082	0.00158483	0.0279405	ME1	-1.6988883	0.0001954	0.008471931
SOCS5	1.5949288	0.0020268	0.0324305	CD99L2	-1.6954199	0.0002075	0.008739345
ZCCHC7	1.5938969	0.00014143	0.0070773	ARHGEF26	-1.695103	0.0002138	0.008875765
TCEB3-AS1	1.5937758	0.00029479	0.010477	DLX4	-1.6946013	0.0003385	0.011360387
MCM3AP-AS1	1.5933817	0.00107251	0.0220666	HPS3	-1.6943925	1.89E-05	0.002714957
CCDC58	1.5929231	7.27E-05	0.0050906	ORAOV1	-1.6940546	0.0001816	0.008091554
LIFR	1.5919166	0.00382119	0.0477322	PRKACB	-1.6940518	4.78E-05	0.004184109
ZNF451	1.5909267	0.00034276	0.01141	HPD	-1.6926159	0.0001201	0.006580369
HOTAIRM1	1.5896801	9.02E-05	0.0056149	DAB1 /// OM	-1.6922661	1.78E-05	0.002631582
LEMD2	1.5886697	0.00022578	0.0091373	STXBP4	-1.6915349	6.84E-05	0.004953055
ZNF420	1.5882969	0.00019709	0.0085105	SMPDL3A	-1.6910369	0.0001117	0.006337688
MRPL42	1.5881963	3.60E-05	0.0036339	DEGS1	-1.690865	0.0002433	0.009498956
TSSC4	1.5881623	0.00013221	0.0068254	MYD88	-1.6898357	2.19E-05	0.002876143
PDGFA	1.588099	0.00389601	0.0483464	PTN	-1.6893264	0.0004061	0.012438224
AKNAD1	1.5868516	0.00394488	0.0486328	HSPA13	-1.6892398	0.0002764	0.010127117
DHPS	1.5866013	0.00022852	0.0091733	KBTBD7	-1.6890621	0.0038887	0.048310761

CCDC91	1.5863068	0.00019535	0.0084719	CEP68	-1.6882174	4.98E-05	0.00430162
BAIAP2	1.5862492	5.41E-05	0.0044917	ALDH3A2	-1.6881433	0.000408	0.012452846
CD44	1.5862178	0.00147766	0.0268062	FAM135A	-1.6873785	8.89E-05	0.005601897
DAAM1	1.5854829	0.00024981	0.0096754	PLA2G3	-1.6873307	0.0003125	0.010883372
XPA	1.5852046	3.52E-05	0.0035942	HTRA3	-1.6841657	0.0001445	0.007164996
PAWR	1.5851247	0.00018538	0.008227	PRPF40A	-1.6833193	0.0003309	0.011222563
ZNF322	1.5843377	0.00158802	0.027963	BRD3	-1.6801657	0.0003109	0.010849207
PPP1R10	1.5835763	0.00034011	0.0113884	SERPINB5	-1.6801335	0.0001423	0.007077318
TCN1	1.583313	0.00015798	0.0074858	SEPP1	-1.679629	0.0020132	0.032365102
TNFRSF10A	1.5832955	0.00028325	0.0102166	LINC00165	-1.6788951	0.0001978	0.008517107
LYPD1	1.5832235	0.00209998	0.0332511	PRKX /// PRK	-1.6777586	7.70E-05	0.005239499
PAFAH1B1	1.5830099	3.19E-05	0.0034617	OIP5-AS1	-1.6772169	0.000258	0.009829718
NRF1	1.5824834	0.00055947	0.0151806	CLDN7	-1.6771899	0.0015852	0.027940456
HIVEP1	1.5821561	0.00034097	0.0113884	LINC00478	-1.6770288	0.0002975	0.010539318
CHD9	1.5816969	0.00104176	0.0217074	RAVER2	-1.6769342	0.0033351	0.043791212
AFAP1L1	1.5810609	0.0003903	0.0121803	LAPTM4A	-1.6765284	8.08E-05	0.005381287
ZNF33A	1.5805435	0.00026844	0.010029	F2RL2	-1.676382	0.0011357	0.022812343
SNHG11 /// SN	1.5805068	0.00070121	0.0172153	SECISBP2	-1.6751029	0.0001248	0.00666347
PDXK	1.5803134	0.00028701	0.01029	LGR4	-1.6745485	0.0010619	0.021934004
AP2B1	1.5802561	0.00017898	0.0080278	LOC1027243	-1.6741782	0.0013428	0.025244729
PATZ1	1.5801865	0.00010922	0.0062726	AGPAT5	-1.6734392	5.55E-05	0.004509022
FAM200B	1.5795573	0.00383438	0.047857	DYNC1I2	-1.6721084	0.0004741	0.013628732
CDK8	1.5793227	0.00027461	0.0101271	ABCB10	-1.6712836	0.0005029	0.014181763
CDK17	1.5792878	0.00123983	0.0240808	PDE7A	-1.6687323	0.0006106	0.015989757
ERCC1	1.5788184	0.0002015	0.0085651	SLC9A3R1	-1.6681258	2.64E-05	0.003114349
HIVEP2	1.5781145	0.00173167	0.0294807	KLHL41	-1.6673685	0.0026708	0.038448664
MUC13	1.5779825	0.0024172	0.0362679	ZMAT3	-1.6673263	4.35E-05	0.003973143
MALT1	1.577677	0.00075463	0.0179076	SSH3	-1.6672964	0.0001839	0.008175558
ZNF585B	1.5775507	0.00065782	0.0166065	OPA1	-1.6671011	0.0001073	0.006215054
GTPBP10 /// LO	1.5773019	8.91E-05	0.0056041	PARVB	-1.666597	0.000627	0.016209008
SULF1	1.5770042	7.25E-05	0.0050906	TACC1	-1.6665362	0.0030262	0.041271441
CCDC88C	1.5759627	0.00128917	0.0245937	SCD	-1.666123	0.0019955	0.032171034
METTL12 /// SN	1.5757533	7.88E-05	0.0052779	OGDH	-1.6649925	0.0001499	0.007310186
MYNN	1.5756638	0.00016876	0.0077797	RNASEH2C	-1.664705	7.83E-05	0.005273797
SYNE1	1.5755672	0.00128513	0.0245508	B4GALNT1	-1.6639748	0.0002549	0.009772891
H2AFJ	1.5754553	0.00085998	0.0192917	TM9SF3	-1.6636069	2.84E-05	0.003261239
USP36	1.5746594	0.00036068	0.0116941	TBC1D16	-1.6629215	2.84E-05	0.003261239
FAM168B	1.5742974	0.00412093	0.0499805	TP53INP2	-1.6620385	0.0002976	0.010539318
RBMS1	1.5742321	0.00012915	0.0067678	LDLRAD4	-1.6619693	0.0001101	0.006302674
C1orf86	1.5742113	0.00157214	0.0278087	ZFYVE21	-1.6619316	0.0002734	0.010108197
SH3BGRL3	1.5738807	8.08E-05	0.0053813	LOC1005075	-1.6607251	9.69E-05	0.005863235
ZBTB47	1.5732565	0.00098219	0.0208873	BLVRA	-1.660672	0.0002354	0.009325151
RNF212	1.5728419	9.15E-05	0.0056647	DPYSL2	-1.6598972	7.62E-05	0.00521106
RUNX3	1.5720983	0.00047028	0.0135733	TNS3	-1.6593657	0.0006591	0.016613394
ETV5	1.5720176	0.00372131	0.0469457	C1orf85	-1.6592646	0.0001919	0.00843615
CLEC2B	1.5715299	0.00039284	0.0122174	EGLN3	-1.6591411	0.0001224	0.006608733
AP4S1	1.5714368	0.00029763	0.0105393	FAM111B	-1.6581642	0.0005808	0.015565822
FZD8 /// MIR4	1.5708903	0.00120871	0.0237635	PPIG	-1.6568021	6.67E-05	0.004922551
JUP	1.5703278	0.00051682	0.0144833	RP11-182L21	-1.6565937	0.0018669	0.030868648
TMEM39B	1.570055	0.00028196	0.0102041	TMCO3	-1.6553995	0.0004069	0.012438224

HCAR3	1.5691116	0.00113536	0.0228123	EID2B	-1.6547176	5.17E-05	0.004393137
YBEY	1.5687758	0.00010717	0.0062151	HIST2H2BE	-1.6542175	0.0003528	0.011574583
SPANXB1	1.568673	0.00270072	0.0386218	CAAP1	-1.6540316	0.0007071	0.017289212
PSTK	1.5685647	0.00130447	0.0247732	PPIC	-1.6528582	9.42E-05	0.005806079
AADACP1	1.5683491	0.00188753	0.0310683	IRAK1BP1	-1.6526076	0.0001741	0.007905684
POT1	1.5682702	4.41E-05	0.0039989	RP11-127B20	-1.6516818	0.0001275	0.006716938
SPOCK1	1.5678744	0.00019365	0.0084719	TAF7L	-1.6512156	0.0025577	0.037456511
YAE1D1	1.5677677	6.81E-05	0.0049531	AKT1	-1.6503723	5.92E-05	0.004582711
PTPN12	1.5672603	0.00074471	0.0177514	MRGPRF	-1.649996	0.0001959	0.008481046
CAPN15	1.5670394	0.00012436	0.0066622	PAPD4	-1.6499857	0.002201	0.034240689
GOSR2	1.5665964	0.00015001	0.0073102	HNRNPLL	-1.6496903	0.0008312	0.019004831
DR1	1.5665332	0.00010379	0.0061151	C6orf62	-1.6492713	2.61E-05	0.003092269
MEX3C	1.5650933	0.00150696	0.0271209	WDR61	-1.6491758	0.0020518	0.032734332
LANCL2	1.5644404	0.00033065	0.0112216	SKAP2	-1.6484797	7.40E-05	0.005126027
SOCS6	1.5643144	0.00296916	0.0409258	GATSL2	-1.6472901	0.0008902	0.019640947
SVIL	1.564187	0.00086235	0.0192917	BRI3BP /// TH	-1.6472186	2.97E-05	0.003339129
TRAF3IP2-AS1	1.5640616	0.00275129	0.0391097	RP11-196G18	-1.6462948	0.0026079	0.037958404
DDRGK1	1.5639457	0.00012721	0.0067169	TNFAIP8	-1.6459727	0.001048	0.021810731
RUNDC1	1.5638166	0.00015325	0.0073918	PEX1	-1.6456028	0.0001738	0.007905232
YAP1	1.5637999	0.0006713	0.0167517	SNORD89	-1.6449915	0.0002206	0.009029277
ABL2	1.5633683	0.00045939	0.0133786	RMDN2	-1.6449745	0.000613	0.016013361
MAGI1	1.5629304	0.00034861	0.0115237	PDGFD	-1.6449014	0.0009746	0.020815649
COL12A1	1.5628085	7.29E-05	0.0050978	HIGD1A	-1.6417215	0.0029326	0.040653469
ZNF174	1.5627689	5.26E-05	0.0044443	ADAM10	-1.6414756	0.0012047	0.02370205
ASPHD1	1.5623913	6.97E-05	0.0049876	AGPS	-1.6405815	0.0001395	0.007044168
ZNF346	1.5614719	0.00175994	0.029754	TPCN2	-1.6402382	0.0001304	0.006808175
DPF2	1.5614537	5.79E-05	0.0045603	HPSE	-1.6400352	0.000118	0.00649501
ZNF165	1.56117	0.00300025	0.0411434	ULK1	-1.6398761	0.0017226	0.029385867
ETV6	1.5609283	0.00024241	0.0094803	OSBPL7	-1.6395732	0.0010892	0.022312312
KLF4	1.5603368	0.00025217	0.0097195	FRAT2	-1.6395223	4.89E-05	0.004238963
MOSPD1	1.5601335	0.00049601	0.0140586	KIF26A	-1.6385738	5.48E-05	0.004508253
CDH2	1.557959	0.00086778	0.0193735	GM2A	-1.6382928	0.0008453	0.01912995
COPZ1	1.5577598	6.17E-05	0.0046954	PTGR2	-1.63728	0.003777	0.047397782
OSGEPL1	1.5576489	0.00012012	0.0065804	MARCKS	-1.6367191	3.78E-05	0.00368328
LYAR	1.5576122	0.00059788	0.0158146	ATP8B2	-1.6366329	0.0003382	0.011358378
FSD1L	1.5575071	0.00361137	0.0460475	PCSK9	-1.6361162	0.0008453	0.01912995
WDR73	1.5574218	0.00012061	0.006583	GAS1	-1.6353008	0.0028623	0.040055898
RHEBL1	1.5565404	0.00022275	0.0090605	ILF3	-1.6352602	7.00E-05	0.004994232
NCF2	1.5561417	0.00066212	0.0166321	SEL1L3	-1.6346035	0.0003311	0.011222563
FANCF	1.5558073	0.00022981	0.0091783	PTPN14	-1.6326769	0.00354	0.045434347
CGRRF1	1.5551827	0.00034731	0.0114945	FAM114A1	-1.6311476	0.0002379	0.009349125
OGT	1.5543788	0.00145245	0.0265682	FLRT3	-1.6300663	0.0004709	0.013580154
CSRNP1	1.5526274	7.59E-05	0.0051967	MBOAT1	-1.6295418	0.0001332	0.006848257
PPM1A	1.5523083	0.00280966	0.0396121	TMEM41B	-1.6292421	3.95E-05	0.003790056
MRPS10	1.5522874	8.10E-05	0.0053813	MAP4K4	-1.6289803	0.000434	0.0129101
KLF6	1.5521026	0.00081373	0.0188041	SUGP2	-1.6287905	0.0012178	0.023838794
RNF6	1.5515973	0.00262358	0.0380792	LOC1027242(	-1.6283748	0.0007772	0.018246002
TBC1D30	1.551514	0.00398327	0.0489404	LMO4	-1.6264068	0.001817	0.030324436
TMEM182	1.5512252	8.47E-05	0.0054912	WDR11	-1.6263758	0.0019001	0.031178152
MXD1	1.5510696	0.00397299	0.0488582	DKFZP434I07	-1.6256493	7.55E-05	0.005184495

SERPINE1	1.5510604	0.00050379	0.0141837	CERS5	-1.6245242	0.0001357	0.006941753
TSC22D1	1.5509901	0.00022399	0.0090883	MOB1A	-1.6243121	0.0035695	0.045727057
ARHGEF28	1.5509366	0.00397032	0.0488473	KDELR2	-1.6239844	3.73E-05	0.00368328
PPP2R3C	1.5507992	9.01E-05	0.0056149	SERPINB6	-1.6236963	0.0001649	0.007684682
NECAP2	1.5500822	0.00012295	0.0066294	CTD-3092A11	-1.6215599	0.0004056	0.012438224
CCDC113	1.5499034	0.00043326	0.012903	TMEM47	-1.6213532	0.0026916	0.038589217
IRAK1	1.5492706	6.25E-05	0.0047345	PIGG	-1.6206758	6.86E-05	0.00495918
PDE6D	1.5489666	0.0002695	0.0100345	DICER1	-1.620142	0.0017644	0.029766324
RPF2	1.5481452	0.00022648	0.0091519	TSPAN3	-1.6192842	0.0001263	0.00669642
HCG18	1.5473814	0.00148745	0.0268936	EPB41	-1.6191435	3.56E-05	0.003616699
IL1R1	1.5458849	0.00225318	0.0347315	SLC47A1	-1.6190411	2.96E-05	0.003330777
TXNL4B	1.5452967	0.00016706	0.0077342	UBE2I	-1.6181053	5.67E-05	0.004543098
NFXL1	1.5447933	0.00173299	0.0294807	CALM1 /// CA	-1.6179801	3.10E-05	0.0034191
EYA3	1.54449	8.28E-05	0.0054148	MTG2	-1.6172789	0.0006143	0.016032582
CHRM3	1.5440308	0.00024928	0.0096661	TRIM34 /// T	-1.6164308	0.000514	0.014410462
CYB5D1	1.5439967	0.00022705	0.0091569	FAR2	-1.6164066	0.001001	0.021188113
PDLIM5	1.543125	0.00395695	0.0487157	GALM	-1.6163848	0.0001113	0.006327189
RAD23A	1.543049	8.72E-05	0.0055813	GNAS	-1.615633	0.0006601	0.016623647
FAM174A	1.5428467	0.0005671	0.0153251	ASF1B	-1.6148308	4.00E-05	0.003802895
LOC101927746	1.5426771	0.00199587	0.032171	SFT2D2	-1.6140563	0.000107	0.006215054
PTGR2 /// ZNF4	1.5424369	6.34E-05	0.0047895	TRIM36	-1.6140405	0.0028125	0.039612119
KLF7	1.5422364	0.00176232	0.0297566	AGO1	-1.6137448	0.0004567	0.013337351
NFATC2	1.5420875	0.00041108	0.0124945	SLC38A10	-1.6128193	0.0004177	0.012624781
THOC6	1.5414172	0.00076688	0.0181057	SLC44A1	-1.6125307	0.0008446	0.01912995
SERTAD1	1.5412132	0.00084703	0.0191606	SMIM19	-1.6124712	4.19E-05	0.003902876
FIP1L1	1.540891	0.00136876	0.0255182	DHFRL1	-1.6122858	0.0019628	0.031815798
SMIM11	1.5408048	0.00065507	0.0166045	ACTR3C	-1.6120277	0.0002987	0.010549097
ITGB5	1.540533	0.00253785	0.0372955	SHISA5	-1.6115895	0.000407	0.012438224
LOC339803	1.540367	7.79E-05	0.0052738	LGALS3BP	-1.6112474	2.91E-05	0.003328475
MARK3	1.5403549	0.00031038	0.0108434	SLC25A51	-1.6105149	0.0001973	0.008510469
PAPPA	1.5402353	0.00413387	0.0500518	GPALPP1	-1.6096394	0.0001533	0.007391792
DPH5	1.5400244	0.00017951	0.0080446	PCNA	-1.6095948	2.26E-05	0.002884643
EGFLAM	1.5399402	0.00015439	0.0074371	IL10RB	-1.6078802	7.32E-05	0.005097785
GABPB1	1.5399126	0.00039266	0.0122174	LOC1027244	-1.6048712	0.0005413	0.01489089
WDR53	1.5395425	0.00076791	0.0181205	AMACR /// C	-1.6047106	0.0004506	0.01321027
TCF12	1.5390775	0.00031458	0.0109342	FAIM	-1.6023072	6.43E-05	0.004838351
TMEM167B	1.5383561	0.00013175	0.0068254	CYP51A1 ///	-1.6020781	3.69E-05	0.003662068
ZNF277	1.537965	0.00176281	0.0297566	PTPN13	-1.6019964	0.00011	0.006302674
CPEB2	1.5370427	0.00157836	0.0278764	PGD	-1.6018619	0.000117	0.006468963
EIF1AD	1.5363285	0.00090304	0.0198176	SENP2	-1.6017958	0.0014845	0.026857594
DUS4L	1.5362652	0.00200864	0.0323232	SMC6	-1.6011738	0.0020453	0.032659469
EPS8L1	1.5361198	0.00012745	0.0067169	SLC30A4	-1.6000675	0.0001672	0.007734689
TRAPPC13	1.5360672	0.00302364	0.0412571	RRM2	-1.598825	0.0001263	0.00669642
PBX2	1.5356531	0.00012469	0.0066635	DIP2A	-1.598668	0.0016572	0.028756638
FBN1	1.535509	0.0003006	0.0106006	TMF1	-1.5982086	0.0006514	0.016550154
MURC	1.5350709	0.00047918	0.0137095	TSPAN6	-1.5979555	0.0002751	0.010127117
SLC2A14 /// SL	1.5346237	9.59E-05	0.0058277	ADAMTS3	-1.5974668	0.000477	0.013679665
ARHGAP29	1.5338447	0.00047975	0.0137121	RAP1GDS1	-1.5974606	0.0003416	0.011395702
LINC00958	1.5334654	0.00103128	0.0215541	CROT	-1.5970383	0.0006186	0.016074069
GADD45B	1.5327197	0.00077662	0.018246	SMC1A	-1.5956005	0.0006607	0.016632093

CDCP1	1.532522	0.00169978	0.0291965	AL832909 ///	-1.5948441	5.42E-05	0.004491723
GTPBP6	1.5323277	0.00012035	0.0065804	RARS2	-1.5946505	0.000142	0.007077297
NEDD4	1.532285	0.00350305	0.0450975	CAT	-1.5945745	0.0001324	0.006825365
MTERF4	1.5318189	0.0003719	0.011905	MLLT11	-1.5943341	0.0001599	0.007555892
FBLIM1	1.5314084	0.00113878	0.0228379	ZMYM6	-1.59383	0.0007163	0.017434169
ASCC3	1.5305544	0.00132792	0.0250617	MBOAT2	-1.593457	0.0016333	0.028474334
JADE1	1.5304496	0.00016068	0.0075559	ACTR3	-1.5931767	0.0002007	0.008560016
LUC7L2	1.5301912	6.45E-05	0.0048384	KDSR	-1.5928222	0.0023824	0.035903297
PLAU	1.5301718	0.00098281	0.0208876	SYNM	-1.5922816	0.0003995	0.012295927
RAB5A	1.5297418	9.76E-05	0.00589	PDZK1	-1.5917431	0.0008575	0.019291743
FBXL12	1.5294565	0.00023344	0.0092756	SULT1A2	-1.5905099	0.0012169	0.023838202
ETV4	1.5286548	0.00088869	0.019616	HSP90B1	-1.5895968	0.0002239	0.009088333
LOC100996732	1.5284067	0.00035332	0.0115746	RTN3	-1.5890135	4.50E-05	0.004027564
MPRIP	1.5280497	0.00014557	0.0071789	MCM6	-1.5869922	0.0008576	0.019291743
PRR16	1.527615	0.00251791	0.037147	LRRFIP1	-1.5867312	0.0005184	0.014519724
ZNF621	1.5274103	0.00118807	0.0235314	USP9X	-1.5864456	0.0022142	0.03434392
RANGRF	1.5273068	6.88E-05	0.0049613	CHD2	-1.5862292	0.0001755	0.00793088
ZXDB	1.5271845	0.00021077	0.0087834	HSPA2	-1.5862271	0.0031674	0.042410105
ANXA2	1.5266135	0.00061716	0.0160682	AIG1	-1.5853123	0.0001418	0.007077297
ALKBH8	1.5264956	0.00202909	0.0324576	ANXA6	-1.5848628	8.46E-05	0.00549118
LOC101927841	1.5262278	6.93E-05	0.0049769	FAM230C	-1.5848603	0.0004071	0.012438224
ZNF426	1.5259817	0.00227127	0.0349217	ALDOC	-1.5847309	8.27E-05	0.005414826
ISL1	1.5257597	0.00036969	0.011855	GSN	-1.5846262	0.0005224	0.014609519
RGS16	1.5257167	0.00042936	0.0128578	TCEAL8	-1.5841329	8.21E-05	0.005388028
CENPL	1.5250997	0.00017502	0.007928	TAF9B	-1.5838707	0.00174	0.029544891
SAP30BP	1.5249636	0.00015744	0.0074787	PTPRS	-1.5832384	0.0007498	0.017854585
LINC00341 /// :	1.5245541	0.00038593	0.012107	LAMA3	-1.5830137	4.92E-05	0.004257409
LOC101928717	1.5244182	0.00013707	0.0069648	MUT	-1.5824926	0.0001494	0.007301149
TMEM161B	1.5241807	0.00062173	0.016118	VAPA	-1.5818283	0.0011654	0.023162372
CRY1	1.5236502	0.00299931	0.0411434	RASA2	-1.5804077	0.0003687	0.011842535
EPHB2	1.5234527	0.00039424	0.0122472	MLLT3	-1.5797101	0.001122	0.022678987
IFI16	1.5229156	0.00013208	0.0068254	GNAI1	-1.5795049	6.83E-05	0.004953055
PHF21A	1.5220338	0.00083778	0.0190647	AP5M1	-1.5793597	0.0035142	0.04518763
USP6NL /// USI	1.5219698	0.00270529	0.0386596	TOMM34	-1.5791435	0.001933	0.0315205
MRPS18B	1.5215647	0.000195	0.0084719	BDH1	-1.5790719	0.0002294	0.009178271
PTPN2	1.5215209	0.00021978	0.0090158	PRKAB2	-1.5783069	0.0002949	0.010476973
LOC101060510	1.5213703	0.00022289	0.0090605	ENOPH1	-1.5781731	0.0023068	0.035286607
RGS2	1.5212098	0.00146426	0.02666	ZBED5-AS1	-1.5772723	0.0004764	0.013672243
ATF4	1.521199	9.10E-05	0.0056489	PHKB	-1.5772376	0.0001996	0.008531215
C9orf156	1.52097	0.00052177	0.0145997	CPS1-IT1	-1.576068	0.0004716	0.013591134
BGLAP /// PMF	1.5203544	0.00201814	0.0323773	LOC10099650	-1.5759381	0.0003557	0.011623981
POC5	1.5197666	0.00019513	0.0084719	CCDC167	-1.5757492	0.0001264	0.00669642
PPP5C	1.519456	0.00045953	0.0133786	EID1	-1.5756191	7.57E-05	0.005193524
WDR13	1.5188872	0.0002101	0.0087621	PIEZO1	-1.5748631	0.0026643	0.038394307
RAD23B	1.5181101	0.00011507	0.0064196	C1orf174	-1.5745487	0.0006086	0.015961256
PCGF1	1.5179983	8.16E-05	0.0053813	NSDHL	-1.5743492	0.0002275	0.00916528
TTC17	1.517927	0.0013962	0.0258567	CALU	-1.5731781	7.20E-05	0.005073395
C15orf39	1.5174275	7.31E-05	0.0050978	B4GALT5	-1.5730611	5.14E-05	0.004381543
ZFPM2	1.5173393	0.00020857	0.0087413	SNAP23	-1.5725694	8.65E-05	0.005567382
NXT1	1.5172885	0.00028584	0.0102683	GXYLT2	-1.572262	5.93E-05	0.004582711

KIF1C	1.5170525	0.00098441	0.0209101	NPY1R	-1.5716569	0.0021673	0.033875503
TTC26	1.5169194	0.00160291	0.0281254	ST3GAL5	-1.5715998	0.0005864	0.015641161
DDAH2	1.516731	0.00045384	0.0132729	DDR1 /// MIR	-1.5709927	0.0007418	0.017695461
SOCS2	1.5165646	0.00024906	0.0096644	PTPN4	-1.570732	0.0001692	0.007783009
PI3	1.5163854	0.0003844	0.0120787	CREBZF	-1.570431	0.0010981	0.022452595
LOC100506870	1.5158899	0.00082249	0.0189224	COQ2	-1.5699014	0.0003909	0.012184796
ZNRD1	1.5154392	0.00011425	0.0063999	ADAM22	-1.5694602	0.0010251	0.021481971
PRH1-PRR4 ///	1.5153601	0.00135436	0.0253817	ADIPOR2	-1.5691948	4.37E-05	0.003988215
SCO2	1.5147938	0.00025668	0.0098071	LINC00472	-1.5689035	0.0010596	0.021897674
ZNF75A	1.5142844	0.00154264	0.0275454	PSAT1	-1.5684855	0.0001983	0.008529724
TMEM87A	1.5142368	0.00043425	0.0129107	MTHFD2	-1.5677039	0.0009643	0.020643036
GPANK1	1.511769	0.00046996	0.0135733	MUM1	-1.5674545	0.0003099	0.010833296
LPHN3	1.5113864	0.00020809	0.0087413	FAM115A	-1.5671882	0.0024376	0.03647399
LOC100506403	1.5110381	0.00072641	0.0175116	PWAR6	-1.5669651	0.0016041	0.028126499
CAMK2D	1.5108713	0.00154956	0.0276147	HMGCR	-1.5669135	8.70E-05	0.005580165
PREPL	1.5103845	0.00341964	0.044474	SPTLC3	-1.5666281	0.002766	0.039238724
ZBTB38	1.510067	0.00076356	0.0180492	ST6GAL1	-1.566617	8.75E-05	0.005587874
IL11	1.5097934	0.0004247	0.0127865	RPL22	-1.5662304	0.0001451	0.007178896
CTTN	1.5097408	0.00021998	0.0090158	ME2	-1.5657307	0.0010391	0.021667083
SLPI	1.5092986	0.00071854	0.0174387	ZBTB41	-1.5648962	0.0029712	0.040929421
CARD6	1.509152	0.0022603	0.0348215	STAU2	-1.5644238	0.0001292	0.006767789
CTSS	1.5089783	0.00054786	0.0149793	FABP3	-1.5644167	0.0012793	0.02450022
AKAP12	1.5088999	0.0023171	0.0353414	LRP8	-1.5643302	0.0001974	0.008510469
AKAP8L	1.5088998	0.00105974	0.0218977	SKP2	-1.5631424	0.0001111	0.006327189
MRPS18A	1.508874	8.88E-05	0.0056019	LOC10192702	-1.5619735	0.0031087	0.041889708
SRGAP1	1.5087943	0.00255377	0.0374196	EPM2AIP1	-1.561945	0.0010574	0.021895983
PHLDB2	1.5085138	0.00083791	0.0190647	FDX1	-1.5618353	0.0001113	0.006327189
NFKBIB	1.5078321	0.000322	0.0110744	TOM1L1	-1.5613607	0.0001004	0.005995306
STMN3	1.5076999	0.00149514	0.0270059	TRIM5	-1.5612142	0.0001277	0.006716938
ETS1	1.5074612	0.00252931	0.0372313	IDE	-1.5603462	0.0001102	0.006302674
ABLIM3	1.5068033	0.000254	0.0097662	COX16 /// SY	-1.5601439	4.56E-05	0.004047384
MYH9	1.5065904	0.00016501	0.0076847	CTH	-1.5600461	0.0023762	0.035867056
STAMBP	1.5061454	0.00030586	0.0107337	SCARB2	-1.5595396	0.0002287	0.009173251
FOXA1	1.5058084	0.00158312	0.0279296	LPIN2	-1.5593982	0.0005969	0.015805096
CXCL2	1.5057018	0.00423443	0.0507491	TRAK2	-1.5590838	0.0007211	0.017460363
CCDC127	1.505561	0.00092377	0.0200892	DUSP9	-1.5587914	0.0011355	0.022812343
CREB3	1.505402	0.00041481	0.0125727	HUWE1	-1.5587542	0.0001808	0.008067732
CDK14	1.5052712	0.00054461	0.0149406	PPP2R1B	-1.5583959	0.000288	0.010316833
SNW1	1.5052475	0.00016956	0.007783	CDH19	-1.5583723	0.000706	0.017286279
PXDC1	1.5048642	0.00014198	0.0070773	MLF1	-1.557735	0.0019854	0.032077418
POLR1E	1.5044045	0.00061694	0.0160682	PDE3B	-1.5575517	6.88E-05	0.004961258
JRK	1.5042727	0.00363294	0.0461909	TMEM30A	-1.5572828	0.0012531	0.024235353
RIOK2	1.5041192	0.00035791	0.0116619	CSTF2T	-1.5570092	0.0002427	0.009484415
N4BP2L2	1.5039666	0.00137929	0.0256575	HTRA1	-1.5568681	0.000222	0.009047844
GLRX	1.5039611	0.0015651	0.0277452	AGMAT	-1.5565917	0.000191	0.008409679
AZI2	1.5039038	0.00231908	0.0353414	SNAPC3	-1.5561499	0.0019171	0.031319877
PDLIM7	1.5039016	0.00126858	0.0243777	DESI2	-1.5547509	0.0004911	0.013947767
ZNF473	1.5034125	0.000427	0.0128264	TCF19	-1.5545824	0.0002091	0.008741303
CCDC86	1.5032282	0.00125528	0.0242517	SELENBP1	-1.5544327	0.0017407	0.029547468
MRPS18C	1.503181	0.00011681	0.006469	UHRF1BP1	-1.5533777	0.0003185	0.010993751

SPRY4	1.5031071	0.00053221	0.0147784	APC	-1.5532075	0.0003935	0.01223058
PTPRK	1.502732	0.00010529	0.00619	SEMA4F	-1.5526249	0.0014801	0.026823429
ORC4	1.5026865	0.00017161	0.0078362	COX4I1	-1.5522006	0.0002147	0.008898723
MTO1	1.5022788	9.51E-05	0.0058128	PASK	-1.5519429	7.85E-05	0.005273797
LSM12	1.5021364	0.00144313	0.0264598	SLC38A6	-1.5518352	0.0009276	0.020126439
RBMS3	1.5020217	0.00020969	0.0087585	STK17B	-1.5511605	0.0002772	0.010140519
SPANXA1 /// SI	1.5016748	0.00052993	0.0147226	UBAP2L	-1.5507101	0.0008518	0.019236608
COX10-AS1	1.501592	0.0017155	0.0293018	NAAA	-1.5505252	0.0003858	0.012106977
RARA	1.5015861	0.00012213	0.0066084	TRAPPC3L	-1.5504288	0.0025212	0.037170101
TMC5	1.5015554	0.00136008	0.0254279	RALBP1	-1.5496421	0.002369	0.03579966
IQCG	1.50124	0.00091482	0.0199591	ZNF224	-1.5488794	0.000605	0.015917926
NSRP1	1.5008775	0.00071914	0.0174441	SULT1A1	-1.5487038	0.0007339	0.017599871
PIBF1	1.5004135	0.0024805	0.0368242	AKAP7	-1.5484018	9.60E-05	0.005827684
KCNC4	1.5001818	0.00022876	0.0091733	SRPX	-1.5483296	0.0001308	0.006824753
ZRANB2	1.4995624	0.00101267	0.0213198	VAMP3	-1.5479764	0.0004152	0.012578015
BANP	1.4992205	0.00031976	0.0110301	ZNF326	-1.5478129	0.0025316	0.037248749
				C20orf194	-1.5475197	0.0005637	0.015257322
				SFXN2	-1.5462663	0.0002224	0.009053373
				SMYD2	-1.5457451	0.0014616	0.026655721
				ASRGL1	-1.5457309	0.0008395	0.019076344
				AP001347.6 /	-1.5456132	0.0025542	0.037419645
				NASP	-1.5455129	0.0001767	0.007968972
				ATR	-1.5452537	0.0001944	0.008471931
				ACBD5	-1.5450453	0.0003873	0.012122002
				EPB41L1	-1.5448435	0.0001962	0.008486798
				MAP4	-1.5445722	0.0032748	0.043307718
				CRYL1	-1.544268	0.0007165	0.017434169
				STAU1	-1.5441594	0.0012764	0.024478514
				CEP162	-1.5439485	0.0002546	0.009772891
				SH3BGRL	-1.5435501	0.0006568	0.016606486
				RAB12	-1.5434373	0.0002784	0.010140697
				SCAF11	-1.5430832	0.0027237	0.038865148
				FAM8A1	-1.5430703	0.0001512	0.007341271
				NCOA2	-1.5428101	0.0007325	0.017592099
				PPP1CB	-1.5424937	0.0001448	0.007168893
				N4BP2	-1.5420711	0.0029902	0.041077704
				SFXN1	-1.5417412	7.74E-05	0.005250669
				NES	-1.5409505	0.0009913	0.021007236
				B4GALT1	-1.5408265	0.0013559	0.02539675
				PRSS23	-1.5407642	0.0002319	0.009240254
				DEPDC1	-1.5399323	0.0002981	0.010543002
				HERC4	-1.5395728	0.0007326	0.017592099
				MIR4458 ///	-1.5394213	0.0011908	0.023531362
				PPM1F	-1.5391553	0.0028245	0.03970534
				BRMS1L	-1.5391247	0.0001943	0.008471931
				FAM114A2	-1.5387175	0.0001466	0.0072024
				CRELD2	-1.5384819	0.0002474	0.009612861
				JAK1	-1.5371714	0.0001149	0.006416215
				AKR7A2	-1.5370495	7.04E-05	0.005010977
				RPL31	-1.5370003	0.0012924	0.024629848

				PSMB2	-1.5369031	0.0011072	0.022555047
				LINC00294	-1.5363026	0.0003394	0.011384894
				PHF20L1	-1.5352567	0.0002155	0.008915418
				MBD6	-1.534433	0.0001038	0.006115113
				CD36	-1.5338796	0.0004467	0.013151767
				CBX6	-1.5338764	0.0001567	0.007457369
				GMPS	-1.5338683	0.000587	0.015647871
				CRTC3	-1.5338137	0.0040722	0.049609735
				RPL13 /// SN(	-1.5332778	0.000823	0.018922407
				BCKDHB	-1.5330192	0.0001163	0.006456077
				CABLES2	-1.5327381	8.57E-05	0.005529468
				RBM8A	-1.5326477	0.0001073	0.006215054
				REEP1	-1.532372	0.002647	0.03827736
				NUCB2	-1.531273	0.0002556	0.009795092
				ABCB6	-1.5309762	0.0016072	0.02816455
				FASTKD2	-1.5306979	0.0001698	0.007783009
				MAPK9	-1.5299831	0.0002894	0.010347964
				ATG2A	-1.5289704	0.0003517	0.011562135
				NCOA3	-1.5288646	0.0004379	0.012988674
				SLC25A36	-1.5279721	0.0005559	0.015120588
				MINPP1	-1.5278228	0.0001139	0.00639562
				TCP11L1	-1.5276878	0.0003225	0.011074411
				SCAI	-1.527467	0.0024742	0.036800452
				GLYR1 /// SEF	-1.5269826	0.0006886	0.016981309
				MREG	-1.5263889	0.0001424	0.007077318
				PALLD	-1.5263625	0.001339	0.025193569
				CCDC25	-1.5262591	0.0005474	0.014978788
				TRIM45	-1.525914	0.0002777	0.010140697
				SMCO4	-1.5257969	0.0002783	0.010140697
				WDSUB1	-1.5251202	0.0012249	0.023910456
				ADA	-1.5248754	0.0002388	0.009360302
				TMEM9	-1.5247549	0.0004294	0.012857831
				MMAB	-1.5245256	0.0014188	0.026135537
				JAKMIP2	-1.5240747	0.0020814	0.033055396
				DBI	-1.5237037	0.0002631	0.0099219
				ATP13A3	-1.5235906	0.0039082	0.048420185
				LMBRD2	-1.5222666	0.0011338	0.022812343
				SGTB	-1.5221255	0.0002801	0.010174314
				RSRC1	-1.5218556	0.0004504	0.01321027
				KDM4B	-1.521375	0.0011006	0.022473939
				PKD2	-1.5212289	0.0023589	0.035696125
				GALNS	-1.5209022	0.0001078	0.006231104
				TTL	-1.5207491	0.0003109	0.010849207
				TTC39C	-1.5204409	0.000439	0.012988674
				ZNF204P	-1.5203836	0.0026273	0.038102912
				STX17	-1.5198695	0.0001666	0.007726875
				CRIPAK	-1.519476	0.0003718	0.01190503
				RAB5B	-1.5192794	0.0001993	0.008531215
				SGK1	-1.5192083	0.0041739	0.050288824
				IQGAP3	-1.5189202	0.0001891	0.008356203

				TMEM106C	-1.5181927	0.0006618	0.016632093
				ZNF782	-1.5181051	0.0029356	0.040660415
				SERINC5	-1.5180399	0.001701	0.029200545
				MFI2	-1.5179866	0.0009796	0.020858441
				BMPR2	-1.5179842	0.0001419	0.007077297
				NUDT3	-1.5179633	0.0003204	0.011030185
				HNRNPC	-1.5179326	0.0002949	0.010476973
				NFIA	-1.5178136	0.0001004	0.005995306
				CREG1	-1.5173939	0.0005298	0.014722552
				EXOC5	-1.517389	0.0005363	0.014847033
				EFR3A	-1.5172043	0.0005508	0.015034886
				LOC1027245:	-1.5170899	0.0017715	0.029802028
				POPDC3	-1.5169008	0.0006235	0.016140521
				HECA	-1.5168499	0.000417	0.012615019
				NNT	-1.5164428	0.0024979	0.036976584
				TM7SF3	-1.5159565	0.0026662	0.03841088
				USP48	-1.5153734	0.0001241	0.006659107
				ITM2B	-1.5152523	0.0001398	0.007044168
				CDYL2	-1.5151605	0.0001547	0.007438976
				SDHC	-1.5151568	0.0016125	0.028227744
				SLC25A3	-1.5151404	0.0011531	0.022980594
				NR2F1 /// NR	-1.514909	0.0002715	0.010084886
				ELOVL5	-1.5147838	7.23E-05	0.005084147
				AMFR	-1.5147132	0.0001159	0.00645377
				ERMP1	-1.5146295	0.0010054	0.02123229
				HOGA1	-1.5145103	0.0005818	0.015565822
				SUB1	-1.5144351	9.49E-05	0.00581278
				WDR44	-1.5141999	0.0002616	0.009900719
				TSPYL1	-1.5133882	0.0003735	0.011918191
				MARK1	-1.513345	0.001873	0.030901273
				CMBL	-1.5129819	0.0002881	0.010316833
				LOC1001323!	-1.5129657	0.0002037	0.008619104
				MB	-1.5120912	0.0038924	0.048336836
				IL17RB	-1.5116291	0.0001991	0.008531215
				SLC16A10	-1.5110276	0.0007178	0.017438701
				ARHGEF40	-1.5108312	0.0006142	0.016032582
				SAP30L	-1.5105942	0.0019196	0.031329754
				CLN5	-1.5103629	0.0006501	0.016534452
				BICC1	-1.5102377	0.0001616	0.007576507
				TFF1	-1.5102349	0.0011264	0.022742416
				HIST3H2A	-1.509692	0.0005397	0.014874168
				ROBO1	-1.5096259	0.00013	0.006795332
				ENTPD5	-1.5090865	0.0002602	0.009886341
				WDR92	-1.5090742	7.39E-05	0.005126027
				C1orf216	-1.5090733	0.0001417	0.007077297
				PIGS	-1.5089857	9.60E-05	0.005827684
				RELL1	-1.5084408	0.0006257	0.016181948
				EIF4E3	-1.5082063	0.0030456	0.041397836
				NUP210	-1.5072682	9.44E-05	0.005806079
				B3GNT2	-1.5072149	0.0025149	0.037132823

				DHTKD1	-1.5069011	0.0003013	0.010612377
				RCN2	-1.5067941	0.0013082	0.024826458
				MRPS14	-1.5067777	0.0001552	0.007438976
				CHCHD7	-1.5062596	0.0017614	0.029756589
				IPW /// LOC1	-1.5059389	0.0006811	0.016880195
				KANSL1-AS1	-1.5059223	0.003287	0.04340951
				FRG1 /// FRG	-1.5057276	0.0028039	0.039602781
				KBTBD4 /// P	-1.5054117	0.0002326	0.009254114
				FREM2	-1.5048821	0.0015598	0.027688158
				LPCAT3	-1.5045179	0.0004604	0.013397135
				DEPDC1B	-1.5042598	0.0009507	0.020489301
				RAP2A	-1.5042294	0.0001063	0.006215054
				TOP2A	-1.504186	0.0003873	0.012122002
				SAE1	-1.5041508	0.0001366	0.0069636
				KLHL8	-1.5040845	0.0001478	0.007234909
				PARP9	-1.50369	0.0031127	0.041927997
				MRPL4	-1.5035121	0.0001669	0.007732104
				A2M-AS1	-1.5034336	0.0018702	0.030883314
				EIF2AK1	-1.5022478	0.0017658	0.029766324
				Mar-06	-1.5021593	0.0007579	0.017938906
				HIST1H2AM	-1.5010597	0.0003358	0.011318831
				NDFIP1	-1.5007436	0.0002091	0.008741303
				GAB1	-1.5006662	0.0036894	0.046640446
				SNX13	-1.5003242	0.0003745	0.011918191
				RRAGA	-1.4999904	0.0001552	0.007438976
				RWDD3	-1.4998406	0.0006273	0.016209646
				ZDHHC23	-1.4996999	0.0006152	0.016041275

Supplementary Table 2.2:

Enriched pathways and GO BP terms for down regulated genes

Enriched Pathway/GOBP	NOM p-val	FDR q-val	Genes (Down Regulated)
RESPONSE TO DRUG (GO:0042493)	0.001	0.01819389	SPINK4,VAV3,HMGCS1,FECH,WFDC1,ASS1,CCNB1,ACTC1,ADSSL1,PDE3A,HADH,MAP2K6,SNCA
STEROL BIOSYNTHETIC PROCESS (GO:0016126)	0.001	0.03725657	SQLE,IDI1,G6PD,HMGCS1,INSIG1,SC5D,MSMO1,DHCR24,DHCR7,HSD17B7,FDFT1
ALCOHOL BIOSYNTHETIC PROCESS (GO:0046165)	0.001	0.061849456	CDS1,IDI1,G6PD,HMGCS1,INSIG1,MSMO1,DHCR24,SGMS2,HSD17B7,SQLE,SPTLC2,SC5D,DHCR7,FDFT1
CHOLESTEROL METABOLIC PROCESS (GO:0008203)	0.001	0.06952534	SQLE,IDI1,G6PD,HMGCS1,FECH,INSIG1,SC5D,MSMO1,DHCR24,DHCR7,HSD17B7,FDFT1
CHOLESTEROL BIOSYNTHESIS_HOMO SAPIENS_R-HSA-191273	0.001	0.07502247	SQLE,IDI1,HMGCS1,SC5D,MSMO1,DHCR24,DHCR7,HSD17B7,FDFT1
REGULATION OF NEURON DEATH (GO:1901214)	0.001	0.08331036	NQO1,CASP7,G6PD,EPHA7,BAG1,CTNNB1,DHCR24,CLU,FGFR3,SNCA
STEROID METABOLIC PROCESS (GO:0008202)	0.00113572	0.07811574	IDI1,G6PD,HMGCS1,FECH,INSIG1,SRD5A3,MSMO1,DHCR24,HSD17B7,SQLE,AKR1B10,SC5D,DHCR7,FDFT1
STEROL METABOLIC PROCESS (GO:0016125)	0.00119403	0.08130006	SQLE,IDI1,G6PD,HMGCS1,FECH,INSIG1,SC5D,MSMO1,DHCR24,DHCR7,HSD17B7,FDFT1
RESPONSE TO TOPOLOGICALLY INCORRECT PROTEIN (GO:0035966)	0.0013089	0.08125589	ATXN3,ASNS,HSPB3,KDELR3,CLU
BIOSYNTHESIS OF AMINO ACIDS_HOMO SAPIENS_HSA01230	0.00135227	0.117092654	PRPS2,IDH1,IDH2,PHGDH,GLUL,ASS1
RESPONSE TO TRANSITION METAL NANOPARTICLE (GO:1990267)	0.00137268	0.15510537	CCNB1,FECH,CYBRD1,ASS1,SNCA
POSITIVE REGULATION OF NEURON DEATH (GO:1901216)	0.00137931	0.08067055	NQO1,CASP7,BAG1,CLU,FGFR3
ISOPRENOID BIOSYNTHETIC PROCESS (GO:0008299)	0.00142857	0.17281742	ALDH1A3,IDI1,HMGCS1,FDFT1
CELLULAR RESPONSE TO INORGANIC SUBSTANCE (GO:0071241)	0.00144928	0.21482651	CCNB1,ASPH,ID2,RYR3,SNCA
REGULATION OF CHOLESTEROL BIOSYNTHESIS BY SREBP (SREBF)_HOMO SAPIENS_R-HSA-1655829	0.00253807	0.08318011	SQLE,IDI1,HMGCS1,INSIG1,SC5D,ELOVL6,DHCR7,SEC24D,FDFT1
CHOLESTEROL BIOSYNTHESIS II (VIA 24 25-DIHYDROLANOSTEROL)_HOMO SAPIENS_PWY66-3	0.00273038	0.153645	SQLE,SC5D,MSMO1,DHCR24,DHCR7,HSD17B7,FDFT1
ORGANIC HYDROXY COMPOUND BIOSYNTHETIC PROCESS (GO:1901617)	0.00280899	0.1549759	CDS1,IDI1,G6PD,HMGCS1,INSIG1,MSMO1,DHCR24,HSD17B7,SQLE,SPTLC2,SC5D,DHCR7,FDFT1,SNCA
POSITIVE REGULATION OF NEURON APOPTOTIC PROCESS (GO:0043525)	0.00291758	0.2194076	NQO1,CASP7,EPHA7,BAG1,FGFR3
SUPERPATHWAY OF CHOLESTEROL BIOSYNTHESIS_HOMO SAPIENS_PWY66-5	0.00315457	0.06735528	SQLE,IDI1,HMGCS1,SC5D,MSMO1,DHCR24,DHCR7,HSD17B7,FDFT1
SMALL MOLECULE BIOSYNTHETIC PROCESS (GO:0044283)	0.00319149	0.17480774	CDS1,IDI1,G6PD,HMGCS1,INSIG1,UROS,ASNS,MSMO1,DHCR24,ELOVL6,PLOD1,SGMS2,HSD17B7,ASS1,SQLE,ALDH1A3,FA2H,SPTLC2,SC5D,PHGDH,PTPLAD2,DHCR7,GLUL,FDFT1
MYELOID CELL DIFFERENTIATION (GO:0030099)	0.00320924	0.15093131	DAB2,FECH,PARP1,ID2,PIR,CTNNB1,SNX10,DNASE2
CHOLESTEROL BIOSYNTHESIS_HOMO SAPIENS_WP197	0.00326584	0.16296035	SQLE,IDI1,HMGCS1,SC5D,MSMO1,DHCR7,FDFT1
GLUTAMINE FAMILY AMINO ACID METABOLIC PROCESS (GO:0009064)	0.00328947	0.17866288	ALDH5A1,ADSSL1,ASNS,PHGDH,GLUL,ASS1
CELLULAR RESPONSE TO METAL ION (GO:0071248)	0.00355114	0.22263457	CCNB1,ASPH,ID2,RYR3,SNCA
RESPONSE TO METAL ION (GO:0010038)	0.00422961	0.1684212	CCNB1,FECH,ASPH,ID2,CYBRD1,CTNNB1,RYR3,ASS1,SNCA
CHOLESTEROL BIOSYNTHESIS I_HOMO SAPIENS_PWY66-341	0.00464191	0.16876855	SQLE,SC5D,MSMO1,DHCR24,DHCR7,HSD17B7,FDFT1
CHOLESTEROL BIOSYNTHESIS III (VIA DESMOSTEROL)_HOMO SAPIENS_PWY66-4	0.00472973	0.14859524	SQLE,SC5D,MSMO1,DHCR24,DHCR7,HSD17B7,FDFT1
NEGATIVE REGULATION OF PROTEIN COMPLEX ASSEMBLY (GO:0031333)	0.00550585	0.2092183	SCIN,INSIG1,RDX,CLU,SNCA
STEROID BIOSYNTHESIS_HOMO SAPIENS_HSA00100	0.00649351	0.205981	SQLE,SC5D,MSMO1,DHCR24,DHCR7,HSD17B7,FDFT1
REGULATION OF NEURON APOPTOTIC PROCESS (GO:0043523)	0.00779727	0.20230176	NQO1,CASP7,G6PD,EPHA7,BAG1,FGFR3,SNCA
DICARBOXYLIC ACID METABOLIC PROCESS (GO:0043648)	0.00829082	0.23782068	ALDH5A1,IDH1,IDH2,ADSSL1,ALDH1L2,GLUL,ASS1
STEROID BIOSYNTHETIC PROCESS (GO:0006694)	5.93E-04	0.014826146	SQLE,IDI1,G6PD,HMGCS1,INSIG1,SRD5A3,SC5D,MSMO1,DHCR24,DHCR7,HSD17B7,FDFT1
CHOLESTEROL BIOSYNTHETIC PROCESS (GO:0006695)	6.20E-04	0.018628284	SQLE,IDI1,G6PD,HMGCS1,INSIG1,SC5D,MSMO1,DHCR24,DHCR7,HSD17B7,FDFT1
ISOPRENOID METABOLIC PROCESS (GO:0006720)	6.44E-04	0.0966241	ALDH1A3,IDI1,AKR1B10,HMGCS1,SRD5A3,PDE3A,FDFT1
GLUTAMINE METABOLIC PROCESS (GO:0006541)	7.02E-04	0.0934765	ALDH5A1,ADSSL1,ASNS,PHGDH,GLUL
ALANINE ASPARTATE AND GLUTAMATE METABOLISM_HOMO SAPIENS_HSA00250	7.46E-04	0.16602619	ALDH5A1,ADSSL1,ASNS,GLUL,ASS1

Supplementary Table 2.3:
Enriched pathways and GO BP terms for Up regulated genes

Enriched Pathway/GOBP	NOM p-val	FDR q-val	Genes (Up Regulated)
NEGATIVE REGULATION OF PHOSPHORUS METABOLIC PROCESS (GO:0010563)	0	0.002367434	DUSP4,ERRFI1,PTPRR,EDN1,PID1,SPINK1,GADD45A,TNFAIP3,IRS2,ADARB1,DKK1,DUSP6,SPOCD1,SPRED2,SH3RF2,IL6,UBASH3B,SPRY2,SH3BP4,BIRC3,DACT1
NEGATIVE REGULATION OF PHOSPHATE METABOLIC PROCESS (GO:0045936)	0	0.003546336	DUSP4,ERRFI1,PTPRR,EDN1,PID1,SPINK1,GADD45A,TNFAIP3,IRS2,ADARB1,DKK1,DUSP6,SPOCD1,SPRED2,SH3RF2,IL6,UBASH3B,SPRY2,SH3BP4,BIRC3,DACT1
NEGATIVE REGULATION OF PROTEIN PHOSPHORYLATION (GO:0001933)	0	0.005562093	DUSP4,ERRFI1,PID1,SPINK1,GADD45A,TNFAIP3,ADARB1,DKK1,DUSP6,SPRED2,IL6,UBASH3B,SPRY2
INFLAMMATORY RESPONSE (GO:0006954)	0	0.005735289	CXCL8,TNFAIP6,SPHK1,TNFAIP3,LIAS,THBS1,HDAC9,IL6,CXCL11,BCL6,IRAK2,CCL5,SAA1,PTX3,SCG2,MGLL
NEGATIVE REGULATION OF PROTEIN KINASE ACTIVITY (GO:0006469)	0	0.006173807	DUSP4,SPRED2,ERRFI1,IL6,UBASH3B,GADD45A,SPRY2,TNFAIP3,ADARB1,DUSP6
NEGATIVE REGULATION OF PROTEIN MODIFICATION PROCESS (GO:0031400)	0	0.006272291	DUSP4,ERRFI1,PID1,SPINK1,GADD45A,TNFAIP3,ISG15,ADARB1,DKK1,DUSP6,SPRED2,IL6,UBASH3B,SPRY2
NEGATIVE REGULATION OF PHOSPHORYLATION (GO:0042326)	0	0.006519801	DUSP4,ERRFI1,PTPRR,PID1,SPINK1,GADD45A,TNFAIP3,IRS2,ADARB1,DKK1,DUSP6,SPRED2,IL6,UBASH3B,SPRY2,BIRC3,DACT1
NEGATIVE REGULATION OF KINASE ACTIVITY (GO:0033673)	5.94E-04	0.007425739	DUSP4,SPRED2,ERRFI1,IL6,UBASH3B,GADD45A,SPRY2,TNFAIP3,IRS2,ADARB1,DUSP6
REGULATION OF STRIATED MUSCLE CELL DIFFERENTIATION (GO:0051153)	0	0.019208333	EFNB2,EDN1,BMP2,DDIT3,BHLHE41,BCL2,SOX9,DKK1,HDAC9
NEGATIVE REGULATION OF TRANSFERASE ACTIVITY (GO:0051348)	0	0.019321296	DUSP4,SPRED2,ERRFI1,IL6,UBASH3B,GADD45A,SPRY2,TNFAIP3,IRS2,ADARB1,DUSP6
POSITIVE REGULATION OF LEUKOCYTE CHEMOTAXIS (GO:0002690)	0	0.019422619	IL6,EDN1,CXCL8,CCL5,VEGFC,THBS1
CELL CHEMOTAXIS (GO:0060326)	0	0.026154587	IL6,CXCL11,DOCK4,EDN1,CXCL8,CCL5,SCG2,EPHA2
SENSORY PERCEPTION OF SOUND (GO:0007605)	0	0.02692999	THRB,GJB3,TIMM9,LRIG1,SPRY2,SNAI2
SENSORY PERCEPTION OF MECHANICAL STIMULUS (GO:0050954)	7.19E-04	0.02738574	THRB,GJB3,TIMM9,LRIG1,SPRY2,SNAI2
RESPONSE TO OTHER ORGANISM (GO:0051707)	5.44E-04	0.02757615	DDX58,IFI44,ISG15,BCL10,ADARB1,DCLK1,OASL,FOSL1,ISG20,IFIH1,IL6,CCL5,PTX3,IER3
REGULATION OF LEUKOCYTE CHEMOTAXIS (GO:0002688)	0.00196464	0.027696999	IL6,EDN1,CXCL8,CCL5,VEGFC,THBS1
POSITIVE REGULATION OF LEUKOCYTE MIGRATION (GO:0002687)	0	0.027698588	IL6,EDN1,CXCL8,CCL5,VEGFC,THBS1
SIGNALING BY FGFR3_HOMO SAPIENS_R-HSA-5654741	0	0.027749043	DUSP4,SPRED2,IRS1,AGO2,SPRY2,IRS2,DUSP6,IER3
TAXIS (GO:0042330)	0.00116482	0.027916707	FOSL1,IL6,CXCL11,DOCK4,EDN1,CXCL8,CCL5,VEGFC,SCG2,EPHA2
RESPONSE TO MECHANICAL STIMULUS (GO:0009612)	0	0.028261505	FOSL1,IL6,GADD45A,TNC,TXNIP,ETV1,SOX9,BCL10,THBS1
CHEMOTAXIS (GO:0006935)	0.00114745	0.02828646	FOSL1,IL6,CXCL11,DOCK4,EDN1,CXCL8,CCL5,VEGFC,SCG2,EPHA2
POSITIVE REGULATION OF CHEMOTAXIS (GO:0050921)	0	0.028466364	IL6,EDN1,CXCL8,CCL5,VEGFC,SCG2,THBS1,F3
POSITIVE REGULATION OF LOCOMOTION (GO:0040017)	0.0010929	0.028520552	EDN1,TGFB2,CXCL8,IRS1,ANXA3,SPHK1,PRKCE,VEGFC,IRS2,PTGS2,THBS1,F3,HDAC9,EGFR,CYR61,IL6,BMP2,CCL5,BCL2,SNAI2,CDH13,SOX9,SCG2
NEGATIVE REGULATION OF MAPK CASCADE (GO:0043409)	0.00123839	0.02854295	DUSP4,SPRED2,PTPRR,SPRY2,DUSP6,DACT1
REGULATION OF ESTABLISHMENT OF PROTEIN LOCALIZATION (GO:0070201)	5.73E-04	0.02866387	CD274,SNAP25,TGFB2,PID1,DDX58,SPHK1,CARD8,VEGFC,PTGS2,EGFR,IL6,SAA1,BCL2,SNAI2,PMAIP1,TNFRSF21,EPHA2,DACT1,BCL2L1
INTERLEUKIN SIGNALING PATHWAY_HOMO SAPIENS_P00036	7.56E-04	0.028743032	IL6,CXCL8,IRS1,IRS2
MAPK1_MAPK3 SIGNALING_HOMO SAPIENS_R-HSA-5684996	0.00185071	0.028902713	DUSP4,SPRED2,IL6,IRS1,IRS2,DUSP6
REGULATION OF TOLL-LIKE RECEPTOR SIGNALING PATHWAY_HOMO SAPIENS_WP1449	0.00203666	0.028926255	IL6,CXCL11,JUN,CXCL8,IRAK2,CCL5,TNFAIP3
POSITIVE REGULATION OF BEHAVIOR (GO:0048520)	0.00122324	0.028980754	IL6,EDN1,CXCL8,CCL5,VEGFC,SCG2,THBS1,F3
POSITIVE REGULATION OF CELL MIGRATION (GO:0030335)	5.57E-04	0.029618705	EDN1,TGFB2,CXCL8,IRS1,ANXA3,SPHK1,PRKCE,VEGFC,IRS2,PTGS2,THBS1,F3,HDAC9,EGFR,CYR61,IL6,BMP2,CCL5,BCL2,SNAI2,CDH13,SOX9
SIGNALING BY FGFR4_HOMO SAPIENS_R-HSA-5654743	0.00117371	0.029731862	DUSP4,SPRED2,IRS1,AGO2,SPRY2,IRS2,DUSP6,IER3
RESPONSE TO VIRUS (GO:0009615)	5.73E-04	0.029734995	SERPINB3,DDX58,IFI44,ISG15,ADARB1,DCLK1,OASL,FOSL1,ISG20,IFIH1,IL6,CCL5,CYP1A1,BCL2,PMAIP1,GBP1,BCL2L1
SIGNALING BY FGFR1_HOMO SAPIENS_R-HSA-5654736	0	0.029983453	DUSP4,SPRED2,IRS1,AGO2,SPRY2,IRS2,DUSP6,IER3
REGULATION OF MUSCLE CELL DIFFERENTIATION (GO:0051147)	6.51E-04	0.03018443	EFNB2,EDN1,BMP2,DDIT3,BHLHE41,BCL2,SOX9,DKK1,HDAC9
SIGNALING BY FGFR2_HOMO SAPIENS_R-HSA-5654738	0.00233645	0.030239318	DUSP4,SPRED2,IRS1,AGO2,SPRY2,IRS2,DUSP6,IER3
POSITIVE REGULATION OF INNATE IMMUNE RESPONSE (GO:0045089)	0.00121507	0.030241227	DUSP4,IFIH1,JUN,DDX58,IRAK2,CCL5,TNFAIP3,BCL10,DUSP6,BIRC3
CYTOKINE SIGNALING IN IMMUNE SYSTEM_HOMO SAPIENS_R-HSA-1280215	5.33E-04	0.031034347	DUSP4,ISG20,SPRED2,IL6,DDX58,IRS1,ISG15,IRS2,IL7R,DUSP6,BIRC3,OASL
NEGATIVE REGULATION OF MAP KINASE ACTIVITY (GO:0043407)	0	0.031773083	DUSP4,SPRED2,SPRY2,DUSP6
POSITIVE REGULATION OF CELL MOTILITY (GO:2000147)	0.00112045	0.033348013	EDN1,TGFB2,CXCL8,IRS1,ANXA3,SPHK1,PRKCE,VEGFC,IRS2,PTGS2,THBS1,F3,HDAC9,EGFR,CYR61,IL6,BMP2,CCL5,BCL2,SNAI2,CDH13,SOX9
POSITIVE REGULATION OF CELLULAR COMPONENT MOVEMENT (GO:0051272)	0.00110988	0.033505257	EDN1,TGFB2,CXCL8,IRS1,ANXA3,SPHK1,PRKCE,VEGFC,IRS2,PTGS2,THBS1,F3,HDAC9,EGFR,CYR61,IL6,BMP2,BCL6,CCL5,BCL2,SNAI2,CDH13,SOX9
REGULATION OF LEUKOCYTE MIGRATION (GO:0002685)	0.00129786	0.033555984	IL6,EDN1,CXCL8,CCL5,VEGFC,THBS1
SIGNALING BY FGFR_HOMO SAPIENS_R-HSA-190236	0	0.03366679	DUSP4,SPRED2,IRS1,AGO2,SPRY2,IRS2,DUSP6,IER3
PATTERN RECOGNITION RECEPTOR SIGNALING PATHWAY (GO:0002221)	0.00123533	0.036397062	DUSP4,IFIH1,JUN,DDX58,IRAK2,TNFAIP3,BCL10,DUSP6,BIRC3
INNATE IMMUNE RESPONSE-ACTIVATING SIGNAL TRANSDUCTION (GO:0002758)	6.25E-04	0.038572934	DUSP4,IFIH1,JUN,DDX58,IRAK2,TNFAIP3,BCL10,DUSP6,BIRC3

REGULATION OF INNATE IMMUNE RESPONSE (GO:0045088)	0.00431832	0.046441685	DUSP4,IFIH1,JUN,DDX58,IRAK2,CCL5,TNFAIP3,BCL10,DUSP6,BIRC3
REGULATION OF BEHAVIOR (GO:0050795)	0.00243754	0.047637463	IL6,EDN1,CXCL8,CCL5,VEGFC,SCG2,THBS1,F3
RESPONSE TO FIBROBLAST GROWTH FACTOR (GO:0071774)	0.00372209	0.04765472	CXCL8,IRS1,CCL5,AGO2,TNC,SPRY2,IRS2
CELLULAR RESPONSE TO BIOTIC STIMULUS (GO:0071216)	0.00190718	0.04867306	ABCA1,IL6,CXCL8,IRAK2,CCL5,DDIT3,TXNIP,TNFAIP3
SIDS SUSCEPTIBILITY PATHWAYS_HOMO SAPIENS_WP706	0.00264375	0.049318876	RYR2,SNAP25,IL6,JUN,CXCL8,THRB,NFYA,HDAC9
LEUKOCYTE CHEMOTAXIS (GO:0030595)	6.53E-04	0.052524004	IL6,CXCL11,EDN1,CXCL8,CCL5,SCG2
REGULATION OF CHEMOTAXIS (GO:0050920)	0.00299222	0.05419648	IL6,EDN1,CXCL8,CCL5,VEGFC,SCG2,THBS1,F3
SECRETION BY CELL (GO:0032940)	0.00304878	0.05518527	ABCA1,IL6,EDN1,CCL5,TMEM79,VEGFC,SCG2,THBS1
POSITIVE REGULATION OF STEM CELL PROLIFERATION (GO:2000648)	0.00274348	0.05532315	NFYA,IRS1,VEGFC,IRS2,SOX9
NEGATIVE REGULATION OF PROTEIN SERINE_THREONINE KINASE ACTIVITY (GO:0071901)	0.00200133	0.055681203	DUSP4,SPRED2,SPRY2,DUSP6
MAPK FAMILY SIGNALING CASCADES_HOMO SAPIENS_R-HSA-5683057	0.00119761	0.056000385	DUSP4,SPRED2,IL6,IRS1,AGO2,IRS2,DUSP6
NON-ALCOHOLIC FATTY LIVER DISEASE (NAFLD)_HOMO SAPIENS_HSA04932	0.00204499	0.056360893	IL6,JUN,NDUFA7,CXCL8,IRS1,DDIT3,IRS2
POSITIVE REGULATION OF PROTEIN TRANSPORT (GO:0051222)	0.00504732	0.05664086	CD274,IL6,TGFB2,DDX58,SPHK1,CARD8,SAA1,VEGFC,PTGS2,EGFR,DACT1
REGULATION OF CELL CYCLE PROCESS (GO:0010564)	0.00116279	0.05700247	EDN1,PID1,PRKCE,GATA6,LIF,GIGYF2,RAD51B,CUL4A,FLCN,CCND1,CYP1A1,BCL2,SOX9,HORMAD1,ZNF655,MAD1L1,DACT1,IER3,BCL2L1
ACTIVATION OF INNATE IMMUNE RESPONSE (GO:0002218)	0.00309215	0.058400616	DUSP4,IFIH1,JUN,DDX58,IRAK2,TNFAIP3,BCL10,DUSP6,BIRC3
SIGNALING BY PDGF_HOMO SAPIENS_R-HSA-186797	0.00638051	0.05900691	DUSP4,SPRED2,IRS1,AGO2,IRS2,THBS1,DUSP6,IER3
RESPONSE TO TUMOR NECROSIS FACTOR (GO:0034612)	0.0026936	0.059184715	EDN1,PID1,CXCL8,CCL5,PTGS2,THBS1,TNFRSF21,KLF2
MONOCARBOXYLIC ACID METABOLIC PROCESS (GO:0032787)	0.00431035	0.06335281	EDN1,SPHK1,ELOVL2,LIAS,PTGS2,PRKAB1,PDP1,SRR,ACOX2,SLCO1B3,UST,CYP1A1,RPP14,ALDH8A1,MGLL
CELLULAR RESPONSE TO TUMOR NECROSIS FACTOR (GO:0071356)	0.00143575	0.06395416	EDN1,PID1,CXCL8,CCL5,THBS1,TNFRSF21,KLF2
BEHAVIOR (GO:0007610)	0.00547113	0.064056285	JUN,THRB,PRKCE,ETV1,PTGS2,THBS1,OXR1,GIGYF2,EGFR,NPAS2,FOSL1,IL6,BCL2
SIGNALING BY EGFR_HOMO SAPIENS_R-HSA-177929	0.00576701	0.06536959	DUSP4,SPRED2,IRS1,AGO2,SPRY2,IRS2,DUSP6,IER3
REGULATION OF CELL ADHESION (GO:0030155)	0.00497238	0.06542526	PTPRR,CXCL8,PRKCE,TPM1,TNC,VEGFC,THBS1,CYR61,PTPRG,BMP2,BCL6,UBASH3B,CCL5,SAA1,BCL2,SNAI2,CDH13,SOX9,CYTH1,EPHA2,TGM2
MAPK CASCADE (GO:0000165)	0.00324044	0.06554355	DUSP4,JUN,IRAK2,CCL5,SOX9,SCG2,DUSP6,EGFR
REGULATION OF PROTEIN TRANSPORT (GO:0051223)	0.00247985	0.06591955	CD274,TGFB2,DDX58,SPHK1,CARD8,VEGFC,PTGS2,EGFR,IL6,SAA1,SNAI2,TNFRSF21,DACT1
POSITIVE REGULATION OF EPITHELIAL CELL PROLIFERATION (GO:0050679)	0.00364964	0.06624051	IL6,JUN,BMP2,CCND1,VEGFC,TNFAIP3,CDH13,SOX9,SCG2,F3,EGFR
MYD88-INDEPENDENT TLR3_TLR4 CASCADE_HOMO SAPIENS_R-HSA-166166	0.00205761	0.066564776	DUSP4,JUN,IRAK2,SAA1,DUSP6,BIRC3
REGULATION OF PROTEIN KINASE B SIGNALING (GO:0051896)	0.00313283	0.06741587	INPP4B,IL6,SPRY2,THBS1,F3,EPHA2
POSITIVE REGULATION OF PROTEIN KINASE B SIGNALING (GO:0051897)	0.00207756	0.068253644	IL6,SPRY2,THBS1,F3
INFLUENZA A_HOMO SAPIENS_HSA05164	0.00465116	0.06847375	IFIH1,IL6,CXCL8,DDX58,CCL5
TOLL LIKE RECEPTOR 3 (TLR3) CASCADE_HOMO SAPIENS_R-HSA-168164	0.00210674	0.06901073	DUSP4,JUN,IRAK2,SAA1,DUSP6,BIRC3
CELL-TYPE SPECIFIC APOPTOTIC PROCESS (GO:0097285)	0.00283688	0.069695525	GNGT1,RYR2,IL6,BCL2,BCL10,TNFRSF21,BCL2L1
RIG-I-LIKE RECEPTOR SIGNALING PATHWAY_HOMO SAPIENS_HSA04622	0.00435098	0.070998356	IFIH1,CXCL8,DDX58,ISG15
ACTIVATED TLR4 SIGNALLING_HOMO SAPIENS_R-HSA-166054	0.00266667	0.07113266	DUSP4,JUN,IRAK2,SAA1,DUSP6,BIRC3
NEGATIVE REGULATION OF PURINE NUCLEOTIDE METABOLIC PROCESS (GO:1900543)	0.00289226	0.07118217	EDN1,PID1,SPRY2,SH3BP4
SIGNALING BY SCF-KIT_HOMO SAPIENS_R-HSA-1433557	0.0066305	0.071652986	DUSP4,SPRED2,IRS1,AGO2,IRS2,DUSP6,IER3
LIPID CATABOLIC PROCESS (GO:0016042)	0.00422833	0.07166646	PLCB4,ACOX2,IRS1,SPHK1,MGLL
TRIF-MEDIATED TLR3_TLR4 SIGNALING_HOMO SAPIENS_R-HSA-937061	0.00208044	0.071672745	DUSP4,JUN,IRAK2,SAA1,DUSP6,BIRC3
REGULATION OF MULTI-ORGANISM PROCESS (GO:0043900)	0.00916905	0.07169645	ISG20,CXCL8,SPINK1,DDX58,CCL5,TNFAIP3,PTX3,ISG15,ADARB1,BIRC3,OASL
POSITIVE REGULATION OF CELL ACTIVATION (GO:0050867)	0.00837321	0.07444609	CD274,IL6,BCL6,CCL5,IRS2,BCL10,IL7R,THBS1
SINGLE-ORGANISM BEHAVIOR (GO:0044708)	0.00434783	0.07547154	FOSL1,JUN,PRKCE,BCL2,ETV1,PTGS2,THBS1,OXR1,GIGYF2,EGFR,NPAS2
RESPONSE TO INORGANIC SUBSTANCE (GO:0010035)	0.00908575	0.075513065	RYR2,JUN,TXNRD2,SPHK1,TNFAIP3,THBS1,EGFR,KLF2,FOSL1,IL6,CCND1,CYP1A1,BCL2,TXNIP,GART
CELLULAR RESPONSE TO LIPOPOLYSACCHARIDE (GO:0071222)	0.0091683	0.07563052	ABCA1,IL6,CXCL8,IRAK2,CCL5,TNFAIP3
REGULATION OF MITOTIC CELL CYCLE PHASE TRANSITION (GO:1901990)	0.00567108	0.07577528	RAD51B,CUL4A,PID1,CCND1,CYP1A1,BCL2,ZNF655,GIGYF2,MAD1L1,DACT1,IER3,BCL2L1
NEGATIVE REGULATION OF NUCLEOTIDE METABOLIC PROCESS (GO:0045980)	0.0043573	0.0760714	EDN1,PID1,SPRY2,SH3BP4

CELLULAR RESPONSE TO LIPID (GO:0071396)	0.00852273	0.07618898	ABCA1,IL6,PID1,CXCL8,THRB,IRAK2,CCL5,TNC,TNFAIP3,SOX9
CELLULAR RESPONSE TO MOLECULE OF BACTERIAL ORIGIN (GO:0071219)	0.00529801	0.076322004	ABCA1,IL6,CXCL8,IRAK2,CCL5,TNFAIP3
DOWNSTREAM SIGNAL TRANSDUCTION_HOMO SAPIENS_R-HSA-186763	0.00603136	0.07714068	DUSP4,SPRED2,IRS1,AGO2,IRS2,DUSP6,IER3
VEGFR2 MEDIATED CELL PROLIFERATION_HOMO SAPIENS_R-HSA-5218921	0.00925355	0.07750045	DUSP4,SPRED2,IRS1,SPHK1,IRS2,DUSP6
TNF SIGNALING PATHWAY_HOMO SAPIENS_HSA04668	0.006875	0.07976957	IL6,EDN1,JUN,JAG1,CCL5,LIF,VEGFC,TNFAIP3,PTGS2,BIRC3
DOWNSTREAM SIGNALING OF ACTIVATED FGFR3_HOMO SAPIENS_R-HSA-5654708	0.00591716	0.08037108	DUSP4,SPRED2,IRS1,AGO2,IRS2,DUSP6,IER3
CELLULAR RESPONSE TO CYTOKINE STIMULUS (GO:0071345)	0.00437159	0.081886984	EDN1,PID1,CXCL8,ISG15,THBS1,OASL,ISG20,IL6,CXCL11,IRAK2,CCL5,SOX9,IL7R
REGULATION OF GRANULOCYTE CHEMOTAXIS (GO:0071622)	0.00592154	0.08215986	EDN1,CXCL8,CCL5,THBS1
SIGNALING BY ERBB4_HOMO SAPIENS_R-HSA-1236394	0.00596659	0.08233849	DUSP4,SPRED2,IRS1,AGO2,IRS2,DUSP6,IER3
DAP12 SIGNALING_HOMO SAPIENS_R-HSA-2424491	0.00654372	0.08236539	DUSP4,SPRED2,IRS1,AGO2,IRS2,DUSP6,IER3
RESPONSE TO DRUG (GO:0042493)	0.00802292	0.082616284	ABCA1,EDN1,JUN,VEGFC,THBS1,DUSP6,EGFR,FOSL1,IL6,SRR,CCND1,LOX,CYP1A1,BCL2,TXNIP
DOWNSTREAM SIGNALING OF ACTIVATED FGFR2_HOMO SAPIENS_R-HSA-5654696	0.00522648	0.08274677	DUSP4,SPRED2,IRS1,AGO2,IRS2,DUSP6,IER3
TOLL-LIKE RECEPTOR SIGNALING PATHWAY_HOMO SAPIENS_HSA04620	0.00498221	0.08287968	IL6,CXCL11,CXCL8,CCL5
DAP12 INTERACTIONS_HOMO SAPIENS_R-HSA-2172127	0.01016746	0.08292123	DUSP4,SPRED2,IRS1,AGO2,IRS2,DUSP6,IER3
NEGATIVE REGULATION OF NUCLEOSIDE METABOLIC PROCESS (GO:0045978)	0.00597907	0.08297979	PID1,SPRY2,SH3BP4
DOWNSTREAM SIGNALING OF ACTIVATED FGFR4_HOMO SAPIENS_R-HSA-5654716	0.00955224	0.08358416	DUSP4,SPRED2,IRS1,AGO2,IRS2,DUSP6,IER3
POSITIVE REGULATION OF DEFENSE RESPONSE (GO:0031349)	0.00399088	0.08361114	DUSP4,JUN,DDX58,TNFAIP3,BCL10,DUSP6,EGFR,IFIH1,IL6,IRAK2,CCL5,BIRC3,TGM2
REGULATION OF REACTIVE OXYGEN SPECIES METABOLIC PROCESS (GO:2000377)	0.00842697	0.08380972	PID1,GADD45A,THBS1,EGFR,BIRC3,IER3
SMALL MOLECULE BIOSYNTHETIC PROCESS (GO:0044283)	0.00973236	0.083816536	EDN1,ELOVL2,LIAS,PTGS2,FGF2,PRKAB1,SRR,ACOX2,MTHFD2L,UST,CYP1A1,ALDH8A1,GART,MGLL
TRANSCRIPTIONAL MISREGULATION IN CANCER_HOMO SAPIENS_HSA05202	0.00715991	0.08396103	IL6,CXCL8,BCL6,ETV1,DUSP6,BIRC3
REGULATION OF CELL CYCLE PHASE TRANSITION (GO:1901987)	0.00641437	0.08407585	RAD51B,CUL4A,PID1,CCND1,CYP1A1,BCL2,ZNF655,GIGYF2,MAD1L1,DACT1,IER3,BCL2L1
AGE-RAGE SIGNALING PATHWAY IN DIABETIC COMPLICATIONS_HOMO SAPIENS_HSA04933	0.01386263	0.08420209	IL6,EDN1,JUN,TGFB2,CXCL8,PLCB4,CCND1,PRKCE,BCL2,VEGFC,F3
DOWNSTREAM SIGNALING OF ACTIVATED FGFR1_HOMO SAPIENS_R-HSA-5654687	0.01294118	0.084466435	DUSP4,SPRED2,IRS1,AGO2,IRS2,DUSP6,IER3
NEGATIVE REGULATION OF VIRAL PROCESS (GO:0048525)	0.00967118	0.08448238	ISG20,CCL5,PTX3,ISG15,OASL
POSITIVE REGULATION OF LEUKOCYTE ACTIVATION (GO:0002696)	0.00961539	0.08475803	CD274,IL6,BCL6,CCL5,IRS2,BCL10,IL7R,THBS1
SHC1 EVENTS IN EGFR SIGNALING_HOMO SAPIENS_R-HSA-180336	0.00991326	0.08482032	DUSP4,SPRED2,IRS1,IRS2,DUSP6
LIPID BIOSYNTHETIC PROCESS (GO:0008610)	0.01053284	0.08494102	EDN1,SPHK1,ELOVL2,LIAS,PTGS2,FGF2,PRKAB1,INPP4B,ACOX2,CYP1A1,PIGL,ALDH8A1,AJUBA,MGLL
SIGNALING PATHWAYS IN GLIOBLASTOMA_HOMO SAPIENS_WP2261	0.00220751	0.085006595	ERRFI1,CCND1,IRS1,SPRY2,EGFR
PEPTIDYL-TYROSINE DEPHOSPHORYLATION (GO:0035335)	0.00856955	0.085065186	DUSP4,PTPRR,UBASH3B,DUSP6,PTPRG
IMMUNE RESPONSE-ACTIVATING SIGNAL TRANSDUCTION (GO:0002757)	0.01499423	0.08520345	DUSP4,JUN,DDX58,PRKCE,TNFAIP3,BCL10,DUSP6,IFIH1,ENAH,IRAK2,BCL2,TNFRSF21,BIRC3
NEGATIVE REGULATION OF INTRACELLULAR SIGNAL TRANSDUCTION (GO:1902532)	0.00762943	0.08538064	DUSP4,SPRED2,PTPRR,BCL6,SPRY2,SH3BP4,THBS1,DUSP6,EPHA2,DACT1
NEGATIVE REGULATION OF TRANSPORT (GO:0051051)	0.00824014	0.08551345	EDN1,PID1,SPINK1,IRS1,PRKCE,LIF,IRS2,PTGS2,THBS1,YRDC,IL6,BCL2,SNAI2,TNFRSF21,IER3
RESPONSE TO CALCIUM ION (GO:0051592)	0.00653168	0.08558642	RYR2,IL6,JUN,CCND1,TXNIP,THBS1,EGFR
NEGATIVE REGULATION OF CYTOKINE PRODUCTION (GO:0001818)	0.00866873	0.08569638	IFIH1,IL6,BCL6,DDX58,GATA6,TNFAIP3,ISG15,THBS1,TNFRSF21,KLF2
TOLL-LIKE RECEPTOR SIGNALING PATHWAY_HOMO SAPIENS_WP75	0.00724113	0.08571813	IL6,CXCL11,CXCL8,CCL5
SIGNALING BY INTERLEUKINS_HOMO SAPIENS_R-HSA-449147	0.00845547	0.085936375	DUSP4,SPRED2,IL6,IRS1,IRS2,IL7R,DUSP6
RESPONSE TO EXTRACELLULAR STIMULUS (GO:0009991)	0.00848416	0.08595282	ABCA1,JUN,SPHK1,TNC,BCL10,PTGS2,EGFR,FOSL1,IL6,CCND1,DDIT3,STC2,CYP1A1,BCL2,PMAIP1
ARMS-MEDIATED ACTIVATION_HOMO SAPIENS_R-HSA-170984	0.01111797	0.08595473	DUSP4,SPRED2,IRS1,IRS2,DUSP6
REGULATION OF LEUKOCYTE APOPTOTIC PROCESS (GO:2000106)	0.00601052	0.088058785	BCL6,CCL5,IRS2,BCL10

PROLONGED ERK ACTIVATION EVENTS_HOMO SAPIENS_R-HSA-169893	0.01288344	0.08807814	DUSP4,SPRED2,IRS1,IRS2,DUSP6
REGULATION OF CYTOKINE PRODUCTION (GO:0001817)	0.00786075	0.08819868	CD274,TGFB2,DDX58,SPHK1,CARD8,GATA6,TNFAIP3,ISG15,BCL10,DTX4,PTGS2,THBS1,KLF2,IFIH1,IL6,BCL6,DDIT3,SAA1,SNAI2,TNFRSF21
POSITIVE REGULATION OF PROTEIN IMPORT INTO NUCLEUS (GO:0042307)	0.00929235	0.08823063	IL6,DDX58,SPHK1,DACT1
RESPONSE TO REACTIVE OXYGEN SPECIES (GO:0000302)	0.01044868	0.08834711	FOSL1,IL6,JUN,TXNRD2,SPHK1,TPM1,BCL2,TXNIP,TNFAIP3,KLF2
SIGNALING BY LEPTIN_HOMO SAPIENS_R-HSA-2586552	0.01719902	0.08839871	DUSP4,SPRED2,IRS1,IRS2,DUSP6
RESPONSE TO PROGESTERONE (GO:0032570)	0.00782361	0.08843364	FOSL1,SPHK1,TXNIP,THBS1
SIGNALING BY VEGF_HOMO SAPIENS_R-HSA-194138	0.01353738	0.088488415	DUSP4,SPRED2,IRS1,SPHK1,VEGFC,IRS2,DUSP6
SIGNALING BY WNT_HOMO SAPIENS_R-HSA-195721	0.00861009	0.08857965	GNGT1,AGO2,RSPO3,ROR1,SOX9,DKK1,DACT1
HEPATITIS C_HOMO SAPIENS_HSA05160	0.00769769	0.08886272	CXCL8,DDX58,PPP2R2D,CLDN1
GRB2 EVENTS IN EGFR SIGNALING_HOMO SAPIENS_R-HSA-179812	0.00809969	0.08908706	DUSP4,SPRED2,IRS1,IRS2,DUSP6
FRS-MEDIATED FGFR1 SIGNALING_HOMO SAPIENS_R-HSA-5654693	0.0124844	0.089140415	DUSP4,SPRED2,IRS1,IRS2,DUSP6
REGULATION OF VIRAL PROCESS (GO:0050792)	0.01153613	0.08916335	ISG20,CXCL8,CCL5,PTX3,ISG15,ADARB1,OASL
MORPHOGENESIS OF AN EPITHELIUM (GO:0002009)	0.00676437	0.08916446	RYR2,EDN1,FOXD1,JAG1,TNC,VEGFC,EGFR,IL6,BMP2,BCL2,RSPO3,SOX9,EPHA2,TGM2
REGULATION OF NUCLEOCYTOPLASMIC TRANSPORT (GO:0046822)	0.0136646	0.089166015	INPP4B,IL6,DDX58,SPHK1,SNAI2,EGFR,DACT1,IER3
SHC1 EVENTS IN ERBB4 SIGNALING_HOMO SAPIENS_R-HSA-1250347	0.01489758	0.08917179	DUSP4,SPRED2,IRS1,IRS2,DUSP6
REGULATION OF STEM CELL PROLIFERATION (GO:0072091)	0.00962199	0.08918695	NFYA,IRS1,VEGFC,SNAI2,IRS2,SOX9
LEUKOCYTE MIGRATION (GO:0050900)	0.01244555	0.089277714	IL6,CXCL11,EDN1,CXCL8,CCL5,SCG2
FRS-MEDIATED FGFR4 SIGNALING_HOMO SAPIENS_R-HSA-5654712	0.01220256	0.08934107	DUSP4,SPRED2,IRS1,IRS2,DUSP6
POSITIVE REGULATION OF LEUKOCYTE PROLIFERATION (GO:0070665)	0.0096022	0.08948557	CD274,IL6,BCL6,CCL5,IRS2
NGF SIGNALLING VIA TRKA FROM THE PLASMA MEMBRANE_HOMO SAPIENS_R-HSA-187037	0.01957296	0.0895527	DUSP4,SPRED2,IRS1,AGO2,IRS2,DUSP6,IER3
RESPONSE TO METAL ION (GO:0010038)	0.01208459	0.08955971	RYR2,IL6,JUN,CCND1,SPHK1,CYP1A1,BCL2,TXNIP,THBS1,EGFR
RESPONSE TO PURINE-CONTAINING COMPOUND (GO:0014074)	0.0064982	0.08962952	FOSL1,RYR2,IL6,JUN,SPHK1
NCAM SIGNALING FOR NEURITE OUT-GROWTH_HOMO SAPIENS_R-HSA-375165	0.00991941	0.0896903	DUSP4,SPRED2,IRS1,IRS2,DUSP6
POSITIVE REGULATION OF LYMPHOCYTE PROLIFERATION (GO:0050671)	0.01081081	0.089813426	CD274,IL6,BCL6,CCL5,IRS2
MONOCARBOXYLIC ACID BIOSYNTHETIC PROCESS (GO:0072330)	0.00965873	0.08991038	EDN1,SRR,ACOX2,ELOVL2,UST,CYP1A1,LIAS,PTGS2,ALDH8A1,PRKAB1,MGLL
SIGNALLING TO ERKS_HOMO SAPIENS_R-HSA-187687	0.0093985	0.08997404	DUSP4,SPRED2,IRS1,IRS2,DUSP6
SIGNALLING TO P38 VIA RIT AND RIN_HOMO SAPIENS_R-HSA-187706	0.01201011	0.08999913	DUSP4,SPRED2,IRS1,IRS2,DUSP6
REGULATION OF LYMPHOCYTE APOPTOTIC PROCESS (GO:0070228)	0.01259259	0.090015225	BCL6,CCL5,IRS2,BCL10
SIGNALLING TO RAS_HOMO SAPIENS_R-HSA-167044	0.01177929	0.09003018	DUSP4,SPRED2,IRS1,IRS2,DUSP6
POSITIVE REGULATION OF INTRACELLULAR PROTEIN TRANSPORT (GO:0090316)	0.00906555	0.090066575	IL6,DDX58,SPHK1,DACT1
FRS-MEDIATED FGFR3 SIGNALING_HOMO SAPIENS_R-HSA-5654706	0.01125	0.090205744	DUSP4,SPRED2,IRS1,IRS2,DUSP6
REGULATION OF HORMONE LEVELS (GO:0010817)	0.00836013	0.09021323	CPA3,IL6,EDN1,SCG5,DHRS2
SOS-MEDIATED SIGNALLING_HOMO SAPIENS_R-HSA-112412	0.0067734	0.090219	DUSP4,SPRED2,IRS1,IRS2,DUSP6
INTERLEUKIN-3 5 AND GM-CSF SIGNALING_HOMO SAPIENS_R-HSA-512988	0.0142063	0.0902249	DUSP4,SPRED2,IRS1,IRS2,DUSP6
FRS2-MEDIATED ACTIVATION_HOMO SAPIENS_R-HSA-170968	0.01433022	0.090239815	DUSP4,SPRED2,IRS1,IRS2,DUSP6
TOLL LIKE RECEPTOR 4 (TLR4) CASCADE_HOMO SAPIENS_R-HSA-166016	0.01132578	0.09026391	DUSP4,JUN,IRAK2,SAA1,DUSP6,BIRC3
CHAGAS DISEASE (AMERICAN TRYPANOSOMIASIS)_HOMO SAPIENS_HSA05142	0.01082544	0.09039036	IL6,JUN,CXCL8,PLCB4,CCL5,PPP2R2D
CARBOXYLIC ACID BIOSYNTHETIC PROCESS (GO:0046394)	0.0105068	0.09040088	EDN1,ELOVL2,LIAS,PTGS2,PRKAB1,SRR,ACOX2,MTHFD2L,UST,CYP1A1,ALDH8A1,GART,MGLL
REGULATION OF EPITHELIAL CELL PROLIFERATION (GO:0050678)	0.01357466	0.09046081	JUN,VEGFC,TNFAIP3,THBS1,F3,EGFR,EFNB2,IL6,BMP2,CCND1,SNAI2,CDH13,SOX9,SCG2
RESPONSE TO NUTRIENT LEVELS (GO:0031667)	0.01175088	0.090470925	ABCA1,JUN,SPHK1,TNC,BCL10,PTGS2,EGFR,IL6,CCND1,DDIT3,STC2,CYP1A1,BCL2,PMAIP1

INTERLEUKIN RECEPTOR SHC SIGNALING_HOMO SAPIENS_R-HSA-912526	0.01997503	0.09050358	DUSP4,SPRED2,IRS1,IRS2,DUSP6
NOD-LIKE RECEPTOR SIGNALING PATHWAY_HOMO SAPIENS_HSA04621	0.00930233	0.09062472	IL6,CXCL8,CCL5,TNFAIP3,BIRC3
REGULATION OF DNA METABOLIC PROCESS (GO:0051052)	0.0122399	0.090688206	IL6,JUN,PID1,CXCL8,BCL6,IL7R,RAD18,EGFR,NPAS2,IER3
INTERLEUKIN-2 SIGNALING_HOMO SAPIENS_R-HSA-451927	0.01065163	0.09075626	DUSP4,SPRED2,IRS1,IRS2,DUSP6
NEGATIVE REGULATION OF MUSCLE CELL DIFFERENTIATION (GO:0051148)	0.00654546	0.090767026	BMP2,DDIT3,BHLHE41,SOX9,DKK1
ORGANIC ACID BIOSYNTHETIC PROCESS (GO:0016053)	0.01604938	0.090780325	EDN1,ELOVL2,LIAS,PTGS2,PRKAB1,SRR,ACOX2,MTHFD2L,UST,CYP1A1,ALDH8A1,GART,MGLL
FRS-MEDIATED FGFR2 SIGNALING_HOMO SAPIENS_R-HSA-5654700	0.01180858	0.09081796	DUSP4,SPRED2,IRS1,IRS2,DUSP6
RAF_MAP KINASE CASCADE_HOMO SAPIENS_R-HSA-5673001	0.01270648	0.09082938	DUSP4,SPRED2,IRS1,IRS2,DUSP6
REGULATION OF SYMBIOSIS ENCOMPASSING MUTUALISM THROUGH PARASITISM (GO:0043903)	0.0087925	0.090856366	ISG20,CXCL8,CCL5,PTX3,ISG15,ADARB1,OASL
MICRORNAS IN CANCER_HOMO SAPIENS_HSA05206	0.01674641	0.09098774	TGFB2,IRS1,PRKCE,TPM1,TNC,IRS2,PTGS2,THBS1,EGFR,CCND1,BCL2,SPRY2,MCL1
HERPES SIMPLEX INFECTION_HOMO SAPIENS_HSA05168	0.01342756	0.09100722	IFIH1,IL6,DDX58,CCL5
TRIF-DEPENDENT TOLL-LIKE RECEPTOR SIGNALING PATHWAY (GO:0035666)	0.01686578	0.09114178	DUSP4,JUN,IRAK2,DUSP6,BIRC3
NEGATIVE REGULATION OF CELL ADHESION (GO:0007162)	0.01737968	0.09120485	BMP2,BCL6,UBASH3B,TNC,SNAI2,CDH13,THBS1
POSITIVE REGULATION OF MONONUCLEAR CELL PROLIFERATION (GO:0032946)	0.01680108	0.09121822	CD274,IL6,BCL6,CCL5,IRS2
INNATE IMMUNE SYSTEM_HOMO SAPIENS_R-HSA-168249	0.00952885	0.09122859	DUSP4,JUN,DDX58,IRS1,TNFAIP3,ISG15,IRS2,BCL10,DTX4,DUSP6,EGFR,IFIH1,SPRED2,IRAK2,AGO2,SAA1,TXNIP,BIRC3,IER3
NEGATIVE REGULATION OF TYPE I INTERFERON PRODUCTION (GO:0032480)	0.00676692	0.09124986	IFIH1,DDX58,TNFAIP3,ISG15
POSITIVE REGULATION OF NUCLEOCYTOPLASMIC TRANSPORT (GO:0046824)	0.00606878	0.09126266	IL6,DDX58,SPHK1,DACT1
EPITHELIAL CELL DIFFERENTIATION (GO:0030855)	0.01259748	0.09131282	TAGLN,JUN,BMP2,THRB,JAG1,CYP1A1,BCL2,GATA6,TXNIP,SOX9,KLF2,EPHA2
NEGATIVE REGULATORS OF RIG-I_MDA5 SIGNALING_HOMO SAPIENS_R-HSA-936440	0.00970149	0.09139549	IFIH1,DDX58,TNFAIP3,ISG15
NEGATIVE REGULATION OF HYDROLASE ACTIVITY (GO:0051346)	0.00748848	0.09139797	SPOCD1,SERPINB3,SH3RF2,IL6,SPINK1,SPRY2,SH3BP4,PTX3,THBS1
POSITIVE REGULATION OF LYMPHOCYTE ACTIVATION (GO:0051251)	0.01214329	0.09148757	CD274,IL6,BCL6,CCL5,IRS2,BCL10,IL7R
IL4-MEDIATED SIGNALING EVENTS_HOMO SAPIENS_CFF33F50-6193-11E5-8AC5-06603EB7F303	0.01155235	0.09158369	BCL6,IRS1,IRS2
DEPHOSPHORYLATION (GO:0016311)	0.01276596	0.091692574	DUSP4,PDP1,INPP4B,PTPRR,UBASH3B,DUSP6,PTPRG
EPITHELIAL CELL PROLIFERATION (GO:0050673)	0.01267512	0.09171365	IL6,CCND1,FST,CDH13,SOX9,EPHA2
TOLL-LIKE RECEPTOR 3 SIGNALING PATHWAY (GO:0034138)	0.01259622	0.09192158	DUSP4,JUN,IRAK2,DUSP6,BIRC3
CYTOPLASMIC PATTERN RECOGNITION RECEPTOR SIGNALING PATHWAY (GO:0002753)	0.00883653	0.09363945	IFIH1,DDX58,IRAK2,TNFAIP3
MYELOID LEUKOCYTE MIGRATION (GO:0097529)	0.01688049	0.093690656	IL6,EDN1,CXCL8,CCL5,SCG2
REGULATION OF LEUKOCYTE PROLIFERATION (GO:0070663)	0.01408451	0.09378819	CD274,IL6,BCL6,CCL5,BCL2,TNFAIP3,IRS2,TNFRSF21
REGULATION OF MYOTUBE DIFFERENTIATION (GO:0010830)	0.0095941	0.094104476	DDIT3,BHLHE41,BCL2,SOX9,HDAC9
RESPONSE TO ALKALOID (GO:0043279)	0.02086137	0.09414926	RYR2,IL6,EDN1,SRR,PRKCE,BCL2,HNMT,BCL2L1
RESPONSE TO LIPOPOLYSACCHARIDE (GO:0032496)	0.01995305	0.09438113	ABCA1,IL6,EDN1,JUN,SRR,CXCL8,IRAK2,CCL5,CYP1A1,TNFAIP3,LIAS
PI3K-AKT SIGNALING PATHWAY_HOMO SAPIENS_HSA04151	0.01686341	0.09443853	LAMB3,IRS1,TNC,VEGFC,OSMR,THBS1,EGFR,GNGT1,IL6,CCND1,GNG4,PPP2R2D,PDGFC,BCL2,IL7R,EPHA2,BCL2L1,MCL1
GROWTH (GO:0040007)	0.01192504	0.09446284	IL6,EDN1,TNC,SPRY2,SOX9,IL7R,GIGYF2,DCLK1
CALCIUM-MEDIATED SIGNALING (GO:0019722)	0.01017442	0.09459127	RYR2,EDN1,CXCL8,SPHK1,EGFR
PROTEIN DEPHOSPHORYLATION (GO:0006470)	0.01619938	0.094946116	DUSP4,PDP1,PTPRR,UBASH3B,DUSP6,PTPRG
REGULATION OF VIRAL GENOME REPLICATION (GO:0045069)	0.01527689	0.097258426	ISG20,CXCL8,CCL5,ISG15,ADARB1,OASL
REGULATION OF PROTEIN SECRETION (GO:0050708)	0.00710227	0.09793691	CD274,IL6,TGFB2,CARD8,SAA1,VEGFC,TNFRSF21
RIG-I_MDA5 MEDIATED INDUCTION OF IFN-ALPHA_BETA PATHWAYS_HOMO SAPIENS_R-HSA-168928	0.01197183	0.09807048	IFIH1,DDX58,SAA1,TNFAIP3,ISG15
CELL FATE COMMITMENT (GO:0045165)	0.02181323	0.0983794	ACVR1,IL6,BMP2,TGFB2,BCL2,SPRY2,SOX9,RUNX2
CELLULAR RESPONSE TO FIBROBLAST GROWTH FACTOR STIMULUS (GO:0044344)	0.01849568	0.098609306	CXCL8,IRS1,CCL5,AGO2,SPRY2,IRS2
REGULATION OF IMMUNE EFFECTOR PROCESS (GO:0002697)	0.02060606	0.09873231	IL6,BCL6,DDX58,IL7R,BIRC3

FC EPSILON RECEPTOR (FCERI) SIGNALING_HOMO SAPIENS_R-HSA-2454202	0.01849711	0.098839134	DUSP4,SPRED2,IRS1,AGO2,IRS2,BCL10,DUSP6,IER3
TOLL LIKE RECEPTOR TLR6:TLR2 CASCADE_HOMO SAPIENS_R-HSA-168188	0.01977401	0.098968625	DUSP4,JUN,IRAK2,SAA1,DUSP6
REGULATION OF ERK1 AND ERK2 CASCADE (GO:0070372)	0.02291422	0.09901012	IL6,PTPRR,SPRY2,DUSP6,EPHA2
TRAF6 MEDIATED INDUCTION OF NFKB AND MAP KINASES UPON TLR7_8 OR 9 ACTIVATION_HOMO SAPIENS_R-HSA-975138	0.01732502	0.09906246	DUSP4,JUN,IRAK2,SAA1,DUSP6
REGULATION OF INTRACELLULAR TRANSPORT (GO:0032386)	0.02122777	0.09916547	RYR2,DDX58,SPHK1,PTGS2,EGFR,INPP4B,IL6,CXCL11,UBASH3B,BCL2,SNAI2,PMAIP1,DACT1,IER3
RESPONSE TO OXIDATIVE STRESS (GO:0006979)	0.02757456	0.0991728	JUN,TXNRD2,SPHK1,TPM1,TNFAIP3,LIAS,DHRS2,OXR1,EGFR,KLF2,FOSL1,IL6,STC2,BCL2,TXNIP
REGULATION OF HORMONE SECRETION (GO:0046883)	0.01668727	0.09918941	IL6,EDN1,IRS1,CCL5,SCG5,IRS2
MYD88 CASCADE INITIATED ON PLASMA MEMBRANE_HOMO SAPIENS_R-HSA-975871	0.01336146	0.09923533	DUSP4,JUN,IRAK2,SAA1,DUSP6
EMBRYONIC MORPHOGENESIS (GO:0048598)	0.0167727	0.099238	RYR2,EDN1,MAFB,LRIG1,SPRY2,RSPO3,VEGFC,SOX9,DKK1,DACT1
RESPONSE TO HYDROGEN PEROXIDE (GO:0042542)	0.01083493	0.09927581	FOSL1,IL6,JUN,SPHK1,BCL2,TXNIP,TNFAIP3,KLF2
FATTY ACID METABOLIC PROCESS (GO:0006631)	0.02465931	0.09929374	EDN1,ACOX2,SPHK1,ELOVL2,CYP1A1,LIAS,RPP14,PTGS2,MGLL
POSITIVE REGULATION OF PROTEIN LOCALIZATION TO NUCLEUS (GO:1900182)	0.0147552	0.09935633	IL6,DDX58,SPHK1,LIF,PTGS2,EGFR,DACT1
POSITIVE REGULATION OF SYNAPTIC TRANSMISSION (GO:0050806)	0.01440922	0.0994376	SNAP25,SPHK1,PRKCE,NAT8L,PTGS2,EGFR
TOLL LIKE RECEPTOR TLR1:TLR2 CASCADE_HOMO SAPIENS_R-HSA-168179	0.01597222	0.099590294	DUSP4,JUN,IRAK2,SAA1,DUSP6
TOLL LIKE RECEPTOR 5 (TLR5) CASCADE_HOMO SAPIENS_R-HSA-168176	0.01257862	0.09961472	DUSP4,JUN,IRAK2,SAA1,DUSP6
REGULATION OF CELLULAR KETONE METABOLIC PROCESS (GO:0010565)	0.01620527	0.09968879	PDP1,IL6,IRS1,IRS2
REGULATION OF CARBOHYDRATE BIOSYNTHETIC PROCESS (GO:0043255)	0.01658604	0.099718615	IL6,IRS1,IRS2
TOLL LIKE RECEPTOR 10 (TLR10) CASCADE_HOMO SAPIENS_R-HSA-168142	0.01694915	0.099731155	DUSP4,JUN,IRAK2,SAA1,DUSP6
REGULATION OF MUSCLE ORGAN DEVELOPMENT (GO:0048634)	0.01464036	0.09979082	EFNB2,IL6,BMP2,DDIT3,BCL2,GATA6,SOX9,DKK1,HDAC9
DIRECT P53 EFFECTORS_HOMO SAPIENS_67C3B75D-6191-11E5-8AC5-06603EB7F303	0.01964395	0.09982809	JUN,BCL6,NFYA,GADD45A,BCL2,LIF,SNAI2,PMAIP1,DKK1,EGFR,EPHA2,MCL1
TOLL LIKE RECEPTOR 9 (TLR9) CASCADE_HOMO SAPIENS_R-HSA-168138	0.01988636	0.099881515	DUSP4,JUN,IRAK2,SAA1,DUSP6
FOXO SIGNALING PATHWAY_HOMO SAPIENS_HSA04068	0.0203202	0.09989662	IL6,BCL6,IRS1,IRS2,IL7R
FCERI MEDIATED MAPK ACTIVATION_HOMO SAPIENS_R-HSA-2871796	0.01717791	0.09990984	DUSP4,SPRED2,IRS1,IRS2,DUSP6
REGULATION OF T CELL DIFFERENTIATION (GO:0045580)	0.01397624	0.09994338	IL6,BCL6,IL7R
NEGATIVE REGULATION OF MITOTIC CELL CYCLE (GO:0045930)	0.01641497	0.09995994	BTG3,CCND1,BCL2,ZNF655,GIGYF2,EGFR,MAD1L1,DACT1,IER3,BCL2L1
MYD88 DEPENDENT CASCADE INITIATED ON ENDOSOME_HOMO SAPIENS_R-HSA-975155	0.01552576	0.10008633	DUSP4,JUN,IRAK2,SAA1,DUSP6
RESPONSE TO MOLECULE OF BACTERIAL ORIGIN (GO:0002237)	0.02258251	0.10009409	ABCA1,IL6,EDN1,JUN,SRR,CXCL8,IRAK2,CCL5,CYP1A1,TNFAIP3,BCL10,LIAS
TRAF6 MEDIATED INDUCTION OF PROINFLAMMATORY CYTOKINES_HOMO SAPIENS_R-HSA-168180	0.01460362	0.10014907	DUSP4,JUN,IRAK2,SAA1,DUSP6
TOLL LIKE RECEPTOR 2 (TLR2) CASCADE_HOMO SAPIENS_R-HSA-181438	0.01198872	0.10027214	DUSP4,JUN,IRAK2,SAA1,DUSP6
TOLL LIKE RECEPTOR 7_8 (TLR7_8) CASCADE_HOMO SAPIENS_R-HSA-168181	0.01414427	0.100328825	DUSP4,JUN,IRAK2,SAA1,DUSP6
DIVALENT INORGANIC CATION HOMEOSTASIS (GO:0072507)	0.0216987	0.10356614	INPP4B,RYR2,EDN1,CCL5,DDIT3,STC2,PRKCE,SAA1,BCL2,EGFR,TGM2
NEGATIVE REGULATION OF VIRAL GENOME REPLICATION (GO:0045071)	0.01798064	0.10400655	ISG20,CCL5,ISG15,OASL
POSITIVE REGULATION OF SEQUENCE-SPECIFIC DNA BINDING TRANSCRIPTION FACTOR ACTIVITY (GO:0051091)	0.01933874	0.10429333	FOSL1,IL6,DDX58,IRAK2,SPHK1,BCL10
SIGNALING BY TYPE 1 INSULIN-LIKE GROWTH FACTOR 1 RECEPTOR (IGF1R)_HOMO SAPIENS_R-HSA-2404192	0.01778305	0.10456859	DUSP4,SPRED2,IRS1,IRS2,DUSP6
CELLULAR RESPONSE TO EXTERNAL STIMULUS (GO:0071496)	0.01716738	0.10596281	FOSL1,JUN,GADD45A,SPHK1,TNC,BCL2,PMAIP1,SOX9,BCL10,PTGS2,EGFR

REGULATION OF ANGIOGENESIS (GO:0045765)	0.02996475	0.10596724	IL6,TNFRSF12A,ANXA3,SPHK1,GATA6,VEGFC,TNFAIP3,PTGS2,THBS1,F3,HDAC9,EPHA2
SIGNALING BY INSULIN RECEPTOR_HOMO SAPIENS_R-HSA-74752	0.0266584	0.106099345	DUSP4,SPRED2,IRS1,IRS2,DUSP6
POSITIVE REGULATION OF B CELL ACTIVATION (GO:0050871)	0.02616279	0.106262095	IL6,BCL6,IRS2
IRS-RELATED EVENTS TRIGGERED BY IGF1R_HOMO SAPIENS_R-HSA-2428928	0.02467613	0.10626693	DUSP4,SPRED2,IRS1,IRS2,DUSP6
TOLL-LIKE RECEPTORS CASCADES_HOMO SAPIENS_R-HSA-168898	0.02076574	0.106325716	DUSP4,JUN,IRAK2,SAA1,DUSP6,BIRC3
MYD88:MAL CASCADE INITIATED ON PLASMA MEMBRANE_HOMO SAPIENS_R-HSA-166058	0.02177068	0.10635562	DUSP4,JUN,IRAK2,SAA1,DUSP6
POSITIVE REGULATION OF POSITIVE CHEMOTAXIS (GO:0050927)	0.01890756	0.10636473	CXCL8,VEGFC,CDH13,SCG2,F3
REGULATION OF LYMPHOCYTE DIFFERENTIATION (GO:0045619)	0.0212766	0.10640381	IL6,BCL6,IL7R
ERBB SIGNALING PATHWAY (GO:0038127)	0.01770452	0.106469885	PTPRR,IRS1,AGO2,SPRY2,IRS2,SOX9
REGULATION OF POSITIVE CHEMOTAXIS (GO:0050926)	0.01572112	0.10663065	CXCL8,VEGFC,CDH13,SCG2,F3
IGF1R SIGNALING CASCADE_HOMO SAPIENS_R-HSA-2428924	0.02565669	0.106685124	DUSP4,SPRED2,IRS1,IRS2,DUSP6
INSULIN RECEPTOR SIGNALLING CASCADE_HOMO SAPIENS_R-HSA-74751	0.02182045	0.10703934	DUSP4,SPRED2,IRS1,IRS2,DUSP6
VEGFA-VEGFR2 PATHWAY_HOMO SAPIENS_R-HSA-4420097	0.02611276	0.10723629	DUSP4,SPRED2,IRS1,SPHK1,IRS2,DUSP6
VALIDATED TARGETS OF C-MYC TRANSCRIPTIONAL REPRESSION_HOMO SAPIENS_6BBDAFA6-6196-11E5-8AC5-06603EB7F303	0.02323992	0.10755258	NFYA,CCND1,GADD45A,CCL5,DDIT3,BCL2,DKK1
NEGATIVE REGULATION OF CELL CYCLE (GO:0045786)	0.02111111	0.10764173	PPP1R15A,BTG3,TGFB2,CXCL8,GADD45A,LIF,TNFAIP3,PTGS2,THBS1,GIGYF2,EGFR,CUL4A,BMP2,CCND1,DDIT3,BCL2,HORMAD1,MAD1L1,DACT1,IER3,BCL2L1
NEUROTROPHIN SIGNALING PATHWAY (GO:0038179)	0.02429668	0.10877707	DUSP4,IRS1,AGO2,IRS2,DUSP6
REGULATION OF GLUCOSE METABOLIC PROCESS (GO:0010906)	0.02380952	0.10900087	IL6,IRS1,IRS2
DNA DAMAGE RESPONSE (ONLY ATM DEPENDENT)_HOMO SAPIENS_WP710	0.02506596	0.109123334	FOSL1,JUN,BCL6,CCND1,IRS1,BCL2,PMAIP1
REGULATION OF PROTEIN MODIFICATION BY SMALL PROTEIN CONJUGATION OR REMOVAL (GO:1903320)	0.024	0.10945668	SPHK1,TNFAIP3,ISG15,BCL10,BIRC3
POSITIVE REGULATION OF SECRETION BY CELL (GO:1903532)	0.03285803	0.10958146	CD274,IL6,EDN1,TGFB2,SPHK1,PRKCE,CARD8,SAA1,VEGFC,IRS2,RAB27B
RESPONSE TO GLUCOSE (GO:0009749)	0.01702443	0.10972666	TXNIP,IRS2,THBS1
TISSUE REMODELING (GO:0048771)	0.02250352	0.10984	IL6,TGFB2,JAG1,LIF,HOXA3,EPHA2,TGM2
NEGATIVE REGULATION OF MULTI-ORGANISM PROCESS (GO:0043901)	0.02581864	0.10994586	ISG20,JUN,CCL5,TNFAIP3,PTX3,ISG15,OASL
CHEMOKINE SIGNALING PATHWAY_HOMO SAPIENS_HSA04062	0.02188783	0.11006861	GNGT1,CXCL11,CXCL8,PLCB4,CCL5
CELLULAR RESPONSE TO INTERLEUKIN-1 (GO:0071347)	0.02896552	0.110106535	EDN1,CXCL8,IRAK2,CCL5,SOX9
NEUROTROPHIN TRK RECEPTOR SIGNALING PATHWAY (GO:0048011)	0.02652684	0.11034576	DUSP4,IRS1,AGO2,IRS2,DUSP6
PEPTIDE LIGAND-BINDING RECEPTORS_HOMO SAPIENS_R-HSA-375276	0.02635328	0.11054753	CXCL11,EDN1,CXCL8,CCL5,SAA1
IRS-MEDIATED SIGNALLING_HOMO SAPIENS_R-HSA-112399	0.02843315	0.11166824	DUSP4,SPRED2,IRS1,IRS2,DUSP6
REGULATION OF SEQUENCE-SPECIFIC DNA BINDING TRANSCRIPTION FACTOR ACTIVITY (GO:0051090)	0.02228739	0.1127494	FOSL1,IL6,JUN,DDX58,IRAK2,SPHK1,DDIT3,TNFAIP3,BCL10
RESPONSE TO INTERLEUKIN-1 (GO:0070555)	0.02977346	0.113167204	EDN1,CXCL8,IRAK2,SPHK1,CCL5,SOX9
REGULATION OF LEUKOCYTE ACTIVATION (GO:0002694)	0.03103448	0.115206905	CD274,IL6,BCL6,CCL5,TNFAIP3,IRS2,BCL10,IL7R,THBS1
TISSUE MORPHOGENESIS (GO:0048729)	0.02480706	0.11559695	RYR2,EDN1,FOXD1,JAG1,TPM1,TNC,VEGFC,EGFR,IL6,BMP2,BCL2,RSPO3,SNAI2,SOX9,EPHA2,TGM2
REGULATION OF CELL ACTIVATION (GO:0050865)	0.02664399	0.11608129	CD274,IL6,BCL6,UBASH3B,CCL5,TNFAIP3,IRS2,BCL10,IL7R,THBS1
POSITIVE REGULATION OF INTRACELLULAR TRANSPORT (GO:0032388)	0.02694236	0.1164248	IL6,CXCL11,DDX58,SPHK1,DACT1
GRANULOCYTE CHEMOTAXIS (GO:0071621)	0.02031144	0.11662957	EDN1,CXCL8,CCL5,SAA1,SCG2
SIGNAL TRANSDUCTION BY PHOSPHORYLATION (GO:0023014)	0.03289058	0.116828	DUSP4,JUN,IRAK2,CCL5,SOX9,SCG2,DUSP6,EGFR
REGULATION OF PROTEIN UBIQUITINATION (GO:0031396)	0.0260521	0.11686695	SPHK1,TNFAIP3,ISG15,BCL10,BIRC3
SIGNALLING BY NGF_HOMO SAPIENS_R-HSA-166520	0.02672865	0.1168984	DUSP4,SPRED2,IRS1,AGO2,IRS2,DUSP6,IER3
NEGATIVE REGULATION OF ERK1 AND ERK2 CASCADE (GO:0070373)	0.02762431	0.11698072	PTPRR,SPRY2,DUSP6
G ALPHA (I) SIGNALLING EVENTS_HOMO SAPIENS_R-HSA-418594	0.02833226	0.11734296	GNGT1,CXCL11,CXCL8,CCL5,SAA1,RGS20,RGS7

REGULATION OF NUCLEAR BETA CATENIN SIGNALING AND TARGET GENE TRANSCRIPTION_HOMO SAPIENS_1590A3B3-6195-11E5-8AC5-06603EB7F303	0.02331779	0.11833839	JUN,CXCL8,TLE1,CCND1,SNAI2,DKK1
MULTICELLULAR ORGANISMAL REPRODUCTIVE PROCESS (GO:0048609)	0.02471265	0.11881515	EDN1,THRB,RNF114,BCL6,FST,CYP1A1,HSF2BP,SOX9,HORMAD1,RAD18,EGFR,BIRC3
REGULATION OF TRANSLATION (GO:0006417)	0.02554745	0.11978746	IL6,CCL5,AGO2,ENC1,THBS1,GIGYF2
CELLULAR CHEMICAL HOMEOSTASIS (GO:0055082)	0.02925373	0.12122305	INPP4B,RYR2,EDN1,CCL5,DDIT3,STC2,PRKCE,SAA1,BCL2,IRS2,RAB38,TGM2
SENSORY PERCEPTION (GO:0007600)	0.03542062	0.12229912	EDN1,THRB,GJB3,TIMM9,LRIG1,SPRY2,SNAI2
FORMATION OF PRIMARY GERM LAYER (GO:0001704)	0.02346041	0.122436546	DUSP4,DKK1,EPHA2
POSITIVE REGULATION OF SECRETION (GO:0051047)	0.02960725	0.12268689	CD274,IL6,EDN1,TGFB2,SPHK1,PRKCE,CARD8,SAA1,VEGFC,IRS2,RAB27B
CELLULAR CALCIUM ION HOMEOSTASIS (GO:0006874)	0.03298071	0.12409176	INPP4B,RYR2,EDN1,CCL5,DDIT3,STC2,PRKCE,SAA1,BCL2,TGM2
ACTIVATION OF IMMUNE RESPONSE (GO:0002253)	0.02357677	0.124229535	DUSP4,JUN,DDX58,PRKCE,TNFAIP3,BCL10,DUSP6,IFIH1,ENAH,IRAK2,BCL2,TNFRSF21,BIRC3
AP-1 TRANSCRIPTION FACTOR NETWORK_HOMO SAPIENS_3CE2F9C5-6189-11E5-8AC5-06603EB7F303	0.02941177	0.1243189	FOSL1,IL6,EDN1,JUN,CXCL8,CCND1
CELLULAR DIVALENT INORGANIC CATION HOMEOSTASIS (GO:0072503)	0.02883436	0.1245423	INPP4B,RYR2,EDN1,CCL5,DDIT3,STC2,PRKCE,SAA1,BCL2,TGM2
NOTCH SIGNALING PATHWAY (GO:0007219)	0.03371501	0.124974586	BMP2,TLE1,JAG1,CCND1,AGO2,SNAI2,MAML3,DTX4,HDAC9
LPA RECEPTOR MEDIATED EVENTS_HOMO SAPIENS_4B994CDE-6194-11E5-8AC5-06603EB7F303	0.03267974	0.12561157	IL6,JUN,CXCL8,PRKCE,EGFR
MAP KINASE ACTIVATION IN TLR CASCADE_HOMO SAPIENS_R-HSA-450294	0.02351212	0.12583952	DUSP4,JUN,IRAK2,DUSP6
TOLL-LIKE RECEPTOR 4 SIGNALING PATHWAY (GO:0034142)	0.02376103	0.12703057	DUSP4,JUN,IRAK2,DUSP6,BIRC3
FIBROBLAST GROWTH FACTOR RECEPTOR SIGNALING PATHWAY (GO:0008543)	0.02960103	0.12918353	IRS1,AGO2,SPRY2,IRS2
MITOTIC CELL CYCLE CHECKPOINT (GO:0007093)	0.02925731	0.12930368	CCND1,GIGYF2,MAD1L1,IER3,BCL2L1
REGULATION OF EPITHELIAL CELL MIGRATION (GO:0010632)	0.03294118	0.12950738	NRP1,PTPRR,EDN1,TGFB2,ANXA3,PRKCE,ITGA2,PDGFB,VEGFC,PTGS2,THBS1,HDAC9,FGF2,PTPRG,SOX9,SLIT2,EPHA2
MYD88-INDEPENDENT TOLL-LIKE RECEPTOR SIGNALING PATHWAY (GO:0002756)	0.03183391	0.13011351	DUSP4,JUN,IRAK2,DUSP6,BIRC3
DEVELOPMENTAL GROWTH INVOLVED IN MORPHOGENESIS (GO:0060560)	0.02232143	0.13011974	TNC,SPRY2,DCLK1
CATION HOMEOSTASIS (GO:0055080)	0.03315571	0.13023448	INPP4B,RYR2,EDN1,CCL5,DDIT3,STC2,PRKCE,SAA1,BCL2,RAB38,EGFR,TGM2
REGULATION OF MITOTIC CELL CYCLE (GO:0007346)	0.03641618	0.13038805	BTG3,EDN1,PID1,SPHK1,PDGFB,GIGYF2,EGFR,THAP1,RAD51B,CUL4A,CCND1,CYP1A1,BCL2,ZNF655,MAD1L1,DACT1,IER3,BCL2L1
REGULATION OF MONONUCLEAR CELL PROLIFERATION (GO:0032944)	0.03550678	0.13272074	CD274,IL6,BCL6,CCL5,BCL2,IRS2,TNFRSF21
SYNDECAN-4-MEDIATED SIGNALING EVENTS_HOMO SAPIENS_076BC549-6196-11E5-8AC5-06603EB7F303	0.0197119	0.1327364	CCL5,TNC,THBS1
TOLL-LIKE RECEPTOR SIGNALING PATHWAY (GO:0002224)	0.03903709	0.13282043	DUSP4,JUN,IRAK2,BCL10,DUSP6,BIRC3
CELLULAR ION HOMEOSTASIS (GO:0006873)	0.0365408	0.13302873	INPP4B,RYR2,EDN1,CCL5,DDIT3,STC2,PRKCE,SAA1,BCL2,RAB38,TGM2
CELLULAR CATION HOMEOSTASIS (GO:0030003)	0.0419916	0.13310824	INPP4B,RYR2,EDN1,CCL5,DDIT3,STC2,PRKCE,SAA1,BCL2,RAB38,TGM2
CALCIUM ION HOMEOSTASIS (GO:0055074)	0.03738318	0.13323577	INPP4B,RYR2,EDN1,CCL5,DDIT3,STC2,PRKCE,SAA1,BCL2,TGM2
MORPHOGENESIS OF A BRANCHING STRUCTURE (GO:0001763)	0.03429603	0.1344726	ACVR1,NRP1,EDN1,COL13A1,PDGFB,FGF2,IL6,BMP2,BCL2,RSPO3,SOX9,SLIT2,EPHA2,TGM2
REGULATION OF MAP KINASE ACTIVITY (GO:0043405)	0.03388823	0.1347459	DUSP4,SPRED2,EDN1,IRAK2,SPRY2,THBS1,DUSP6
VITAMIN B12 METABOLISM_HOMO SAPIENS_WP1533	0.02721563	0.13485013	ABCA1,IL6,CCL5,SAA1
RESPONSE TO ACID CHEMICAL (GO:0001101)	0.03617863	0.1352796	ABCA1,IL6,EDN1,PID1,TNC,SH3BP4,SOX9,DKK1
ATF-2 TRANSCRIPTION FACTOR NETWORK_HOMO SAPIENS_C76C900A-618A-11E5-8AC5-06603EB7F303	0.03555268	0.13532294	IL6,JUN,CXCL8,CCND1,GADD45A,DDIT3,BCL2
NEGATIVE REGULATION OF CELL CYCLE PROCESS (GO:0010948)	0.03882877	0.13744661	CCND1,BCL2,LIF,HORMAD1,GIGYF2,MAD1L1,DACT1,IER3,BCL2L1
LONG-CHAIN FATTY ACID METABOLIC PROCESS (GO:0001676)	0.03549383	0.1374492	SPHK1,ELOVL2,CYP1A1,PTGS2,MGLL
APOPTOSIS-RELATED NETWORK DUE TO ALTERED NOTCH3 IN OVARIAN CANCER_HOMO SAPIENS_WP2864	0.0238806	0.13769756	IL7R,THBS1,TNFRSF21,IER3
POSITIVE REGULATION OF PROTEIN SECRETION (GO:0050714)	0.02575423	0.13776438	CD274,IL6,TGFB2,CARD8,SAA1,VEGFC
REGULATION OF LYMPHOCYTE PROLIFERATION (GO:0050670)	0.03470858	0.13788338	CD274,IL6,BCL6,CCL5,BCL2,IRS2,TNFRSF21
G ALPHA (Z) SIGNALLING EVENTS_HOMO SAPIENS_R-HSA-418597	0.02551021	0.13988152	GNGT1,RGS17,GNG4,PRKCE,RGS20

ALPHA9 BETA1 INTEGRIN SIGNALING EVENTS_HOMO SAPIENS_0F5519CF-6188-11E5-8AC5-06603EB7F303	0.0329912	0.13999735	TNC,VEGFC,SAT1
GRANULOCYTE MIGRATION (GO:0097530)	0.03099174	0.14023876	EDN1,CXCL8,CCL5,SAA1,SCG2
POSITIVE REGULATION OF RESPONSE TO EXTERNAL STIMULUS (GO:0032103)	0.03658536	0.14038624	IL6,EDN1,CXCL8,CCL5,VEGFC,SCG2,THBS1,F3
REGULATION OF STRIATED MUSCLE TISSUE DEVELOPMENT (GO:0016202)	0.03519799	0.14045337	EFNB2,ACVR1,BMP2,DDIT3,BCL2,GATA6,SOX9,DKK1,HDAC9,FGF2
REGULATION OF PROTEIN TARGETING (GO:1903533)	0.03213115	0.14056045	IL6,DDX58,SPHK1,SNAI2,EGFR,DACT1
REGULATION OF SKELETAL MUSCLE TISSUE DEVELOPMENT (GO:0048641)	0.04063861	0.14060557	ACVR1,DDIT3,BCL2,SOX9,DKK1,HDAC9
INSULIN SIGNALING_HOMO SAPIENS_WP481	0.03305204	0.1408384	SNAP25,JUN,IRS1,IRS2,STX4
POSITIVE REGULATION OF T CELL ACTIVATION (GO:0050870)	0.03194888	0.14097166	CD274,IL6,CCL5,BCL10,IL7R
REGULATION OF INTRACELLULAR PROTEIN TRANSPORT (GO:0033157)	0.0309822	0.14361973	IL6,DDX58,SPHK1,SNAI2,EGFR,DACT1
POSITIVE REGULATION OF PEPTIDYL-SERINE PHOSPHORYLATION (GO:0033138)	0.04027778	0.14425153	IL6,SPRY2
POSITIVE REGULATION OF OSSIFICATION (GO:0045778)	0.03840878	0.14496726	ACVR1,IL6,BMP2,TGFB2,JAG1,RUNX2,CYR61
REGULATION OF MUSCLE TISSUE DEVELOPMENT (GO:1901861)	0.04077792	0.14532499	EFNB2,ACVR1,BMP2,DDIT3,BCL2,GATA6,SOX9,DKK1,HDAC9,FGF2
EGF_EGFR SIGNALING PATHWAY_HOMO SAPIENS_WP437	0.04623179	0.14534949	ERRFI1,PTPRR,SPRY2
CYTOKINE-CYTOKINE RECEPTOR INTERACTION_HOMO SAPIENS_HSA04060	0.04022989	0.1466679	ACVR1,TGFB2,CXCL8,TNFRSF12A,LIF,VEGFC,OSMR,EGFR,IL6,CXCL11,BMP2,CCL5,PDGFC,IL7R,TNFRSF21
REGULATION OF CELLULAR CARBOHYDRATE METABOLIC PROCESS (GO:0010675)	0.04809619	0.14774422	IL6,IRS1,IRS2,IER3
REGULATION OF G-PROTEIN COUPLED RECEPTOR PROTEIN SIGNALING PATHWAY (GO:0008277)	0.04299754	0.14803502	GNGT1,RGS17,EDN1,CXCL8,GNG4,CCL5,RGS20,MGLL,GPR158,RGS7
GPCR DOWNSTREAM SIGNALING_HOMO SAPIENS_R-HSA-388396	0.03657143	0.14807259	GNGT1,RGS17,CXCL11,EDN1,CXCL8,PLCB4,CCL5,SAA1,RGS20,MGLL,RGS7
TGF-BETA SIGNALING PATHWAY_HOMO SAPIENS_P00052	0.02930665	0.14809336	FOSL1,ACVR1,JUN,BMP2,TGFB2,MAP3K7CL
RETROGRADE ENDOCANNABINOID SIGNALING_HOMO SAPIENS_HSA04723	0.02969562	0.14809698	GNGT1,PLCB4,GNG4,PTGS2,MGLL
REGULATION OF PROTEIN IMPORT INTO NUCLEUS (GO:0042306)	0.03590426	0.1481836	IL6,DDX58,SPHK1,SNAI2,EGFR,DACT1
SECOND-MESSENGER-MEDIATED SIGNALING (GO:0019932)	0.0343461	0.14818491	RYR2,EDN1,CXCL8,SPHK1,SOX9,EGFR
UNSATURATED FATTY ACID METABOLIC PROCESS (GO:0033559)	0.03465347	0.14822233	EDN1,SPHK1,ELOVL2,CYP1A1,PTGS2,MGLL
CELL CYCLE CHECKPOINT (GO:0000075)	0.03963839	0.14829318	CCND1,HORMAD1,GIGYF2,MAD1L1,IER3,BCL2L1
POSITIVE REGULATION OF EPITHELIAL CELL MIGRATION (GO:0010634)	0.040625	0.1484203	NRP1,EDN1,TGFB2,ANXA3,PRKCE,ITGA2,PDGFB,VEGFC,PTGS2,THBS1,HDAC9,FGF2,SOX9
REGULATION OF NEUROLOGICAL SYSTEM PROCESS (GO:0031644)	0.03610906	0.14861088	IL6,EDN1,MGLL,TNFRSF21
TRANSMEMBRANE TRANSPORT OF SMALL MOLECULES_HOMO SAPIENS_R-HSA-382551	0.04886212	0.15063761	GNGT1,RYR2,GNG4,MYO5B,SLCO1B3,SLC2A3,NALCN,ANO2
INTEGRATED BREAST CANCER PATHWAY_HOMO SAPIENS_WP1984	0.0432295	0.15080522	FOSL1,JUN,CCND1,IRS1,GADD45A,MAP3K7CL,BCL2,EGFR
MEASLES_HOMO SAPIENS_HSA05162	0.03708791	0.1509264	IFIH1,IL6,CCND1,DDX58,TNFAIP3
INSULIN RESISTANCE_HOMO SAPIENS_HSA04931	0.04385965	0.15115532	IL6,IRS1,IRS2
INTERFERON ALPHA_BETA SIGNALING_HOMO SAPIENS_R-HSA-909733	0.03597651	0.1518284	ISG20,ISG15,OASL
REGULATION OF LYMPHOCYTE ACTIVATION (GO:0051249)	0.04117647	0.15425865	CD274,IL6,BCL6,CCL5,TNFAIP3,IRS2,BCL10,IL7R
RESPONSE TO KETONE (GO:1901654)	0.04688504	0.15432705	FOSL1,EDN1,CCND1,SPHK1,TXNIP,THBS1,EGFR
MYELOID LEUKOCYTE ACTIVATION (GO:0002274)	0.04752066	0.15659487	JUN,CXCL8,ANXA3,CCL5,PRKCE,DHRS2,RELB
REGULATION OF PEPTIDYL-SERINE PHOSPHORYLATION (GO:0033135)	0.0541272	0.15777053	IL6,SPRY2,DKK1
RESPONSE TO ENDOPLASMIC RETICULUM STRESS (GO:0034976)	0.05039267	0.15797396	PPP1R15A,JUN,CXCL8,CCND1,DDIT3,STC2,BCL2,THBS1
INTRACELLULAR RECEPTOR SIGNALING PATHWAY (GO:0030522)	0.04618227	0.1588073	IFIH1,RBM14,THRB,DDX58,IRAK2,BCL2,TXNIP,TNFAIP3,BCL2L1
REGULATION OF CYCLIC NUCLEOTIDE METABOLIC PROCESS (GO:0030799)	0.03445635	0.1588684	ABCA1,CXCL11,EDN1
RESPONSE TO TYPE I INTERFERON (GO:0034340)	0.03736264	0.15977755	ISG20,ISG15,OASL
NUCLEOTIDE-BINDING DOMAIN LEUCINE RICH REPEAT CONTAINING RECEPTOR (NLR) SIGNALING PATHWAYS_HOMO SAPIENS_R-HSA-168643	0.04041096	0.16011046	IRAK2,BCL2,TXNIP,TNFAIP3,BIRC3,BCL2L1
REGULATION OF CELL CYCLE G1_S PHASE TRANSITION (GO:1902806)	0.04689564	0.16017501	CUL4A,PID1,CCND1,CYP1A1,BCL2,ZNF655,GIGYF2,DACT1
ANGIOGENESIS (GO:0001525)	0.05192532	0.16034986	NRP1,JUN,TGFB2,CXCL8,JAG1,TNFRSF12A,FN1,VEGFC,PTGS2,SAT1,THBS1,CYR61,HOXA3,CDH13,SCG2,EPHA2

TWEAK SIGNALING PATHWAY_HOMO SAPIENS_WP2036	0.04034582	0.16279334	IL6,JUN,TNFRSF12A,CCL5,BIRC3,RELB
REGULATION OF RAS PROTEIN SIGNAL TRANSDUCTION (GO:0046578)	0.05053908	0.1631174	ABCA1,BCL6,SPRY2
POSITIVE REGULATION OF SMOOTH MUSCLE CELL PROLIFERATION (GO:0048661)	0.05	0.16577965	IL6,EDN1,JUN,CCL5,CDH13,EGFR,TGM2
NEGATIVE REGULATION OF CATABOLIC PROCESS (GO:0009895)	0.04497355	0.16620047	BCL2,SPRY2,SH3BP4,EGFR,MAD1L1,IER3,BCL2L1
TOLL-LIKE RECEPTOR 2 SIGNALING PATHWAY (GO:0034134)	0.03862661	0.16630861	DUSP4,JUN,IRAK2,DUSP6
NEGATIVE REGULATION OF HORMONE SECRETION (GO:0046888)	0.04492053	0.1664696	IL6,EDN1,IRS1
CELL-CELL ADHESION (GO:0098609)	0.04778637	0.1667154	PCDH9,AMIGO2,CDH13,CLDN1
MAPK SIGNALING PATHWAY_HOMO SAPIENS_WP382	0.04323308	0.16674723	DUSP4,PTPRR,JUN,GADD45A,DDIT3,DUSP6,EGFR
REGULATION OF CAMP METABOLIC PROCESS (GO:0030814)	0.04118497	0.16722266	ABCA1,CXCL11,EDN1
CELL-CELL ADHESION VIA PLASMA-MEMBRANE ADHESION MOLECULES (GO:0098742)	0.03971119	0.16738053	PCDH9,AMIGO2,CDH13,CLDN1
INTEGRIN CELL SURFACE INTERACTIONS_HOMO SAPIENS_R-HSA-216083	0.04659014	0.16827577	COL13A1,TNC,THBS1
FOCAL ADHESION_HOMO SAPIENS_WP306	0.04379124	0.16968043	JUN,LAMB3,CCND1,TNC,BCL2,VEGFC,THBS1,EGFR,BIRC3
DEVELOPMENTAL GROWTH (GO:0048589)	0.05256242	0.16986242	TNC,SPRY2,SOX9,DCLK1
CELLULAR RESPONSE TO TYPE I INTERFERON (GO:0071357)	0.04745042	0.17008469	ISG20,ISG15,OASL
POSITIVE REGULATION OF T CELL PROLIFERATION (GO:0042102)	0.04839911	0.17011103	CD274,IL6,CCL5
STEM CELL DEVELOPMENT (GO:0048864)	0.0405694	0.17029867	EDN1,JAG1,BCL2,SNAI2,SOX9
TOLL-LIKE RECEPTOR 10 SIGNALING PATHWAY (GO:0034166)	0.04978355	0.1704176	DUSP4,JUN,IRAK2,DUSP6
REGULATION OF G1_S TRANSITION OF MITOTIC CELL CYCLE (GO:2000045)	0.04878049	0.17054307	CUL4A,PID1,CCND1,CYP1A1,BCL2,ZNF655,GIGYF2,DACT1
TYPE I INTERFERON SIGNALING PATHWAY (GO:0060337)	0.03971631	0.17056389	ISG20,ISG15,OASL
NUCLEOTIDE-BINDING DOMAIN LEUCINE RICH REPEAT CONTAINING RECEPTOR SIGNALING PATHWAY (GO:0035872)	0.05479452	0.17057422	IRAK2,BCL2,TXNIP,TNFAIP3,BCL2L1
NEGATIVE REGULATION OF CELL CYCLE PHASE TRANSITION (GO:1901988)	0.05917553	0.1706547	CCND1,BCL2,ZNF655,GIGYF2,MAD1L1,DACT1,IER3,BCL2L1
STEROID METABOLIC PROCESS (GO:0008202)	0.04562453	0.17071578	ABCA1,ACOX2,SLCO1B3,CYP1A1,DHRS2
REGULATION OF CYTOKINE SECRETION (GO:0050707)	0.04459562	0.17083833	CD274,IL6,SAA1,TNFRSF21
POSITIVE REGULATION OF NITRIC OXIDE BIOSYNTHETIC PROCESS (GO:0045429)	0.05532213	0.1710209	IL6,EDN1,PTX3
SIGNALING BY NOTCH_HOMO SAPIENS_R-HSA-157118	0.0608365	0.1711638	JUN,TLE1,JAG1,CCND1,AGO2,MAML3,DTX4,HDAC9
NEGATIVE REGULATION OF MITOTIC CELL CYCLE PHASE TRANSITION (GO:1901991)	0.06466667	0.17157277	CCND1,BCL2,ZNF655,GIGYF2,MAD1L1,DACT1,IER3,BCL2L1
OSTEOBLAST DIFFERENTIATION (GO:0001649)	0.05344494	0.17170724	RSL1D1,SYNCRIP,BMP2,TNC,FHL2,SNAI2,RUNX2,CYR61,EPHA2
RESPONSE TO HEXOSE (GO:0009746)	0.04232425	0.17197302	TXNIP,IRS2,THBS1
TOLL-LIKE RECEPTOR TLR6:TLR2 SIGNALING PATHWAY (GO:0038124)	0.04646176	0.17216	DUSP4,JUN,IRAK2,DUSP6
TOLL-LIKE RECEPTOR 9 SIGNALING PATHWAY (GO:0034162)	0.04454023	0.17243175	DUSP4,JUN,IRAK2,DUSP6
REGULATION OF NITRIC OXIDE BIOSYNTHETIC PROCESS (GO:0045428)	0.05156038	0.1724514	IL6,EDN1,PTX3
CYTOKINE-MEDIATED SIGNALING PATHWAY (GO:0019221)	0.05670971	0.1727706	ISG20,IL6,CXCL11,CCL5,ISG15,IL7R,OASL
RHEUMATOID ARTHRITIS_HOMO SAPIENS_HSA05323	0.05184659	0.17412148	IL6,JUN,CXCL8,CCL5
REGULATION OF CYSTEINE-TYPE ENDOPEPTIDASE ACTIVITY (GO:2000116)	0.05393401	0.17414275	IL6,BCL10,THBS1,F3,BIRC3
TOLL-LIKE RECEPTOR 5 SIGNALING PATHWAY (GO:0034146)	0.04625088	0.17430152	DUSP4,JUN,IRAK2,DUSP6
REGULATION OF ANOIKIS (GO:2000209)	0.04464286	0.17443053	TLE1,BCL2,SNAI2,BCL2L1,MCL1
NEGATIVE REGULATION OF ANOIKIS (GO:2000811)	0.05510052	0.17443891	TLE1,BCL2,SNAI2,BCL2L1,MCL1
CELLULAR METAL ION HOMEOSTASIS (GO:0006875)	0.05142503	0.17471069	INPP4B,RYR2,EDN1,CCL5,DDIT3,STC2,PRKCE,SAA1,BCL2,TGM2
WNT SIGNALING PATHWAY_HOMO SAPIENS_P00057	0.06050956	0.17494428	EDN1,PLCB4,TLE1,PCDH9,CCND1,GNG4,PRKCE,CDH13,DACT1
MORPHOGENESIS OF A BRANCHING EPITHELIUM (GO:0061138)	0.0627666	0.17631757	ACVR1,NRP1,EDN1,PDGFB,FGF2,IL6,BMP2,BCL2,RSPO3,SOX9,SLIT2,EPHA2,TGM2
REGULATION OF CELLULAR RESPONSE TO STRESS (GO:0080135)	0.05736783	0.17649291	EDN1,TGFB2,GPR37,GADD45A,EGFR,NPAS2,SH3RF3,CUL4A,SPRED2,RSL1D1,BMP2,SNAI2,PMAIP1,DACT1,IER3,BCL2L1,MCL1
REGULATION OF CELL-MATRIX ADHESION (GO:0001952)	0.05274412	0.17652763	BCL6,BCL2,VEGFC,CDH13,THBS1
REGULATION OF RESPONSE TO DNA DAMAGE STIMULUS (GO:2001020)	0.06482064	0.17654446	CUL4A,SPRED2,SNAI2,PMAIP1,EGFR,NPAS2,IER3,BCL2L1,MCL1
POSTTRANSCRIPTIONAL REGULATION OF GENE EXPRESSION (GO:0010608)	0.06655093	0.17670701	IL6,CCL5,AGO2,ENC1,THBS1,GIGYF2,DACT1

DEFENSE RESPONSE TO VIRUS (GO:0051607)	0.05882353	0.17671134	ISG20,IL6,BCL2,PMAIP1,ISG15,ADARB1,GBP1,OASL,IFIT2
REGULATION OF PRODUCTION OF MOLECULAR MEDIATOR OF IMMUNE RESPONSE (GO:0002700)	0.04806992	0.17695221	IL6,BCL6,BCL10
TOLL-LIKE RECEPTOR TLR1:TLR2 SIGNALING PATHWAY (GO:0038123)	0.05961675	0.17731333	DUSP4,JUN,IRAK2,DUSP6
TCF DEPENDENT SIGNALING IN RESPONSE TO WNT_HOMO SAPIENS_R-HSA-201681	0.05160858	0.17743568	TLE1,RSPO3,SOX9,DKK1,DACT1
REGULATION OF FIBROBLAST GROWTH FACTOR RECEPTOR SIGNALING PATHWAY (GO:0040036)	0.05155367	0.17752923	SPRY2,THBS1,DUSP6
POSITIVE REGULATION OF PROTEIN CATABOLIC PROCESS (GO:0045732)	0.05737158	0.17844623	ZFAND2A,TNFAIP3,SOX9,DACT1,IER3
WNT SIGNALING PATHWAY (GO:0016055)	0.06003639	0.17898339	RYR2,TLE1,CCND1,DDIT3,RSPO3,SNAI2,DKK1,DACT1
RESPONSE TO BACTERIUM (GO:0009617)	0.05506608	0.18000452	IL6,ISG15
REGULATION OF CARBOHYDRATE METABOLIC PROCESS (GO:0006109)	0.05666452	0.18009026	IL6,IRS1,IRS2,IER3
POSITIVE REGULATION OF T CELL DIFFERENTIATION (GO:0045582)	0.04988995	0.1801531	IL6,IL7R
PI3K EVENTS IN ERBB4 SIGNALING_HOMO SAPIENS_R-HSA-1250342	0.05401845	0.1801881	IRS1,AGO2,IRS2,IER3
GASTRIN-CREB SIGNALLING PATHWAY VIA PKC AND MAPK_HOMO SAPIENS_R-HSA-881907	0.05818597	0.1802498	DUSP4,GNGT1,SPRED2,EDN1,PLCB4,IRS1,SAA1,IRS2,DUSP6,MGLL
REGULATION OF EXTRINSIC APOPTOTIC SIGNALING PATHWAY (GO:2001236)	0.05518896	0.18028095	ACVR1,TGFB2,TNFRSF12A,BCL2,TNFAIP3,SNAI2,PMAIP1,BCL10,SCG2,THBS1,BCL2L1,MCL1
POSITIVE REGULATION OF CELL CYCLE PROCESS (GO:0090068)	0.05163043	0.1804912	RAD51B,CUL4A,EDN1,CCND1,PRKCE,CYP1A1,GATA6
FOLATE METABOLISM_HOMO SAPIENS_WP176	0.04640719	0.18053494	ABCA1,IL6,SAA1,GART
HEPATITIS B_HOMO SAPIENS_HSA05161	0.06314433	0.18059407	IFIH1,IL6,JUN,CXCL8,CCND1,DDX58,BCL2
BRAIN DEVELOPMENT (GO:0007420)	0.0505618	0.18064277	SRR,SPHK1,IRS2,HNMT,PTPRG
PI-3K CASCADE:FGFR2_HOMO SAPIENS_R-HSA-5654695	0.06580733	0.18080635	IRS1,AGO2,IRS2,IER3
EMBRYO DEVELOPMENT (GO:0009790)	0.06332757	0.18080808	ACVR1,AKIRIN2,PTPRR,EDN1,TGFB2,TPM1,GATA6,KLF2,FOSL1,FLCN,BMP2,GJB3,MAFF,CYP1A1,SNAI2,SLIT2,BCL2L1
EPITHELIUM DEVELOPMENT (GO:0060429)	0.0620438	0.18088315	ERRFI1,BMP2,JAG1,LAMB3,GJB5,SNAI2,SOX9,DKK1,EGFR,EPHA2
POSITIVE REGULATION OF TRANSLATION (GO:0045727)	0.05431755	0.18098208	IL6,CCL5,SAMD4A,THBS1
MESENCHYMAL CELL DEVELOPMENT (GO:0014031)	0.05498534	0.18116781	EDN1,JAG1,BCL2,SNAI2,SOX9
REGULATION OF CALCIUM ION TRANSPORT (GO:0051924)	0.0551543	0.18120417	RYR2,CXCL11,SPINK1,UBASH3B,CCL5,STC2,BCL2,PTGS2
REGULATION OF T CELL ACTIVATION (GO:0050863)	0.05803299	0.18154158	CD274,IL6,BCL6,CCL5,BCL10,IL7R
SENESCENCE AND AUTOPHAGY IN CANCER_HOMO SAPIENS_WP615	0.05705329	0.18163383	RSL1D1,IL6,JUN,BMP2,CXCL8,BCL2,THBS1
POSITIVE REGULATION OF ENDOTHELIAL CELL PROLIFERATION (GO:0001938)	0.05710401	0.18188782	NRP1,JUN,BMP2,PDGFB,CDH13,SCG2,F3,FGF2
REGULATION OF EXTRINSIC APOPTOTIC SIGNALING PATHWAY IN ABSENCE OF LIGAND (GO:2001239)	0.04023845	0.1819397	TGFB2,BCL2,SNAI2,BCL2L1,MCL1
REGULATION OF PROTEIN LOCALIZATION TO NUCLEUS (GO:1900180)	0.06688103	0.18196799	IL6,DDX58,SPHK1,LIF,SNAI2,PTGS2,EGFR,DACT1
HOMEOSTASIS OF NUMBER OF CELLS (GO:0048872)	0.05060585	0.18204924	BCL2,TNFAIP3,PMAIP1,IL7R,GIGYF2
REGULATION OF B CELL ACTIVATION (GO:0050864)	0.0538262	0.18205807	IL6,BCL6,BCL2,TNFAIP3,IRS2,TNFRSF21
PI-3K CASCADE:FGFR3_HOMO SAPIENS_R-HSA-5654710	0.06191117	0.18232019	IRS1,AGO2,IRS2,IER3
REGULATION OF TYPE I INTERFERON PRODUCTION (GO:0032479)	0.05751015	0.1823886	IFIH1,DDX58,TNFAIP3,ISG15,DTX4
DEFENSE RESPONSE TO OTHER ORGANISM (GO:0098542)	0.0591133	0.18723206	ISG20,IL6,ANXA3,BCL2,PMAIP1,ISG15,ADARB1,GBP1,OASL,IFIT2
POSITIVE REGULATION OF REACTIVE OXYGEN SPECIES METABOLIC PROCESS (GO:2000379)	0.05551443	0.18757626	PID1,GADD45A,THBS1,EGFR
RESPONSE TO STARVATION (GO:0042594)	0.05904059	0.18760653	JUN,SPHK1,DDIT3,BCL2,PMAIP1
REGULATION OF CELL DIVISION (GO:0051302)	0.05469235	0.18760678	FLCN,EDN1,TGFB2,PRKCE,PDGFC,LIF,PDGFB,TXNIP,VEGFC,HORMAD1,FGF2,MAD1L1
GAB1 SIGNALOSOME_HOMO SAPIENS_R-HSA-180292	0.06233244	0.18761477	IRS1,AGO2,IRS2,IER3
METAL ION HOMEOSTASIS (GO:0055065)	0.0667071	0.1876567	INPP4B,RYR2,EDN1,CCL5,DDIT3,STC2,PRKCE,SAA1,BCL2,EGFR,TGM2
NEGATIVE REGULATION OF RAS PROTEIN SIGNAL TRANSDUCTION (GO:0046580)	0.05833957	0.18776582	BCL6,SPRY2
POSITIVE REGULATION OF OSTEOBLAST DIFFERENTIATION (GO:0045669)	0.07868383	0.19036266	ACVR1,IL6,BMP2,JAG1,RUNX2,CYR61
MYOMETRIAL RELAXATION AND CONTRACTION PATHWAYS_HOMO SAPIENS_WP289	0.06662665	0.19136508	GNGT1,RGS17,RYR2,IL6,JUN,GNG4,PRKCE,MAFF,RGS20,RGS7
OSSIFICATION (GO:0001503)	0.0573711	0.19305442	COL13A1,BCL2,SOX9,EGFR,RUNX2
PIP3 ACTIVATES AKT SIGNALING_HOMO SAPIENS_R-HSA-1257604	0.0634501	0.19413638	IRS1,AGO2,IRS2,IER3

PI-3K CASCADE:FGFR4_HOMO SAPIENS_R-HSA-5654720	0.05737158	0.19432934	IRS1,AGO2,IRS2,IER3
REGULATION OF VASCULATURE DEVELOPMENT (GO:1901342)	0.06760729	0.19448404	IL6,TNFRSF12A,ANXA3,SPHK1,GATA6,VEGFC,TNFAIP3,PTGS2,THBS1,F3,HDAC9,EPHA2
ROLE OF LAT2_NTAL_LAB ON CALCIUM MOBILIZATION_HOMO SAPIENS_R-HSA-2730905	0.06233244	0.1945235	IRS1,AGO2,IRS2,IER3
KERATINOCYTE DIFFERENTIATION_HOMO SAPIENS_H_KERATINOCYTEPATHWAY	0.05635838	0.19481778	JUN,PRKCE,BCL2,EGFR,BIRC3
IL2-MEDIATED SIGNALING EVENTS_HOMO SAPIENS_A2A1883C-6193-11E5-8AC5-06603EB7F303	0.05229227	0.19481862	JUN,IRS1,PRKCE,BCL2,IRS2
REGULATION OF SMOOTH MUSCLE CELL PROLIFERATION (GO:0048660)	0.06953846	0.19521652	IL6,EDN1,JUN,CCL5,TNFAIP3,CDH13,EGFR,TGM2
EMBRYO DEVELOPMENT ENDING IN BIRTH OR EGG HATCHING (GO:0009792)	0.06978119	0.19595394	FOSL1,PTPRR,EDN1,BMP2,GJB3,MAFF,TPM1,CYP1A1,GATA6,KLF2,BCL2L1
TNF ALPHA SIGNALING PATHWAY_HOMO SAPIENS_WP231	0.07345808	0.19605312	IL6,JUN,TNFAIP3,BIRC3,BCL2L1
POSITIVE REGULATION OF APOPTOTIC SIGNALING PATHWAY (GO:2001235)	0.07367716	0.19843322	TGFB2,TNFRSF12A,BCL2,PMAIP1,BCL10,THBS1,BCL2L1,MCL1
PI-3K CASCADE:FGFR1_HOMO SAPIENS_R-HSA-5654689	0.07588986	0.19954018	IRS1,AGO2,IRS2,IER3
PI3K_AKT ACTIVATION_HOMO SAPIENS_R-HSA-198203	0.07124183	0.20127298	IRS1,AGO2,IRS2,IER3
SELENIUM MICRONUTRIENT NETWORK_HOMO SAPIENS_WP15	0.07271418	0.20182845	ABCA1,IL6,TXNRD2,SAA1,PTGS2
NITROGEN COMPOUND TRANSPORT (GO:0071705)	0.07816538	0.2028195	ABCA1,SNAP25,IL6,EDN1,SLC35F3,SH3BP4
PROTEIN FOLDING_HOMO SAPIENS_R-HSA-391251	0.07251909	0.2029478	GNGT1,GNG4,SPHK1,KIFC3,RGS7
APOPTOSIS_HOMO SAPIENS_WP254	0.06176853	0.20421074	JUN,CASP4,BCL2,PMAIP1,TNFRSF21,BIRC3,BCL2L1,MCL1
POSITIVE REGULATION OF CELL CYCLE (GO:0045787)	0.07620818	0.20438285	FOSL1,RAD51B,CUL4A,EDN1,TGFB2,CCND1,SPHK1,PRKCE,CYP1A1,GATA6,EGFR
AXON GUIDANCE_HOMO SAPIENS_R-HSA-422475	0.07790055	0.20444283	DUSP4,SPRED2,IRS1,NTN4,IRS2,DUSP6,EPHA2
CELLULAR RESPONSE TO EXTRACELLULAR STIMULUS (GO:0031668)	0.07016394	0.2052456	FOSL1,JUN,SPHK1,TNC,BCL2,PMAIP1
STABILIZATION AND EXPANSION OF THE E-CADHERIN ADHERENS JUNCTION_HOMO SAPIENS_F1A6CD94-6195-11E5-8AC5-06603EB7F303	0.06545454	0.20532356	ENAH,STX4,KIFC3,EGFR,EPHA2
CHAPERONIN-MEDIATED PROTEIN FOLDING_HOMO SAPIENS_R-HSA-390466	0.07071483	0.2059546	GNGT1,GNG4,SPHK1,KIFC3,RGS7
CANONICAL WNT SIGNALING PATHWAY (GO:0060070)	0.06271777	0.20635484	RYR2,CCND1,RSPO3,SNAI2
POSITIVE REGULATION OF NF-KAPPAB TRANSCRIPTION FACTOR ACTIVITY (GO:0051092)	0.0602649	0.20712556	IL6,IRAK2,SPHK1,BCL10
BLOOD VESSEL REMODELING (GO:0001974)	0.06306306	0.20738791	TGFB2,JAG1,LIF,HOXA3,TGM2
CELL GROWTH (GO:0016049)	0.07799619	0.20769049	IL6,EDN1,SOX9,IL7R,DCLK1
MUSCLE STRUCTURE DEVELOPMENT (GO:0061061)	0.06681351	0.21043625	IFRD1,ETV1
SMALL CELL LUNG CANCER_HOMO SAPIENS_HSA05222	0.09250507	0.2112614	LAMB3,CCND1,ITGA2,BCL2,FN1,PTGS2,BIRC3,BCL2L1
REGULATION OF BLOOD PRESSURE (GO:0008217)	0.07212622	0.211262	EDN1,VEGFC,IER3
NEGATIVE REGULATION OF TRANSLATION (GO:0017148)	0.07198306	0.21129848	SYNCRIP,AGO2,ENC1,SAMD4A,GIGYF2
CALCIUM REGULATION IN THE CARDIAC CELL_HOMO SAPIENS_WP536	0.08219178	0.2125747	GNGT1,RGS17,RYR2,GJB3,GNG4,PRKCE,GJB5,RGS20,RGS7
EPIDERMIS DEVELOPMENT (GO:0008544)	0.07112676	0.21557052	TGFB2,LAMB3,GJB5,SOX9,DKK1,EGFR
NEGATIVE REGULATION OF EPITHELIAL CELL MIGRATION (GO:0010633)	0.07648725	0.21622181	PTPRR,THBS1,PTPRG
PATHWAYS IN CANCER_HOMO SAPIENS_HSA05200	0.08264004	0.21927792	JUN,CXCL8,LAMB3,VEGFC,EGFR,GNGT1,IL6,BMP2,PLCB4,CCND1,GNG4,BCL2,BIRC3,BCL2L1
POSITIVE REGULATION OF LYMPHOCYTE DIFFERENTIATION (GO:0045621)	0.08117565	0.22109841	IL6,IL7R
RESPONSE TO RETINOIC ACID (GO:0032526)	0.08067665	0.22163706	ABCA1,TNC,SOX9,DKK1
POSITIVE REGULATION OF ENDOTHELIAL CELL MIGRATION (GO:0010595)	0.08500669	0.22265644	NRP1,EDN1,ANXA3,PDGFB,VEGFC,PTGS2,THBS1,HDAC9,FGF2
EPIDERMAL GROWTH FACTOR RECEPTOR SIGNALING PATHWAY (GO:0007173)	0.08404908	0.22356027	IRS1,AGO2,SPRY2,IRS2,SOX9
SPHINGOLIPID SIGNALING PATHWAY_HOMO SAPIENS_HSA04071	0.0754717	0.22443059	PLCB4,SPHK1,PRKCE,PPP2R2D,BCL2
ORGANIC HYDROXY COMPOUND METABOLIC PROCESS (GO:1901615)	0.07582349	0.22469665	ABCA1,INPP4B,TGFB2,PLCB4,GPR37,ACOX2,SPHK1,SLCO1B3,CYP1A1,BCL2
AMOEBIASIS_HOMO SAPIENS_HSA05146	0.0804829	0.22475287	SERPINB3,IL6,CXCL8,PLCB4,LAMB3
CELLULAR RESPONSE TO NUTRIENT LEVELS (GO:0031669)	0.08811189	0.22557797	JUN,SPHK1,TNC,BCL2,PMAIP1
POSITIVE REGULATION OF LIPID METABOLIC PROCESS (GO:0045834)	0.08161866	0.22623818	IRS1,PRKCE,PDGFB,IRS2,PTGS2,FGF2,CYR61
NEGATIVE REGULATION OF IMMUNE SYSTEM PROCESS (GO:0002683)	0.10034803	0.2273377	INPP4B,CD274,MAFB,BCL6,TNFAIP3,SOX9,BCL10,IL7R,THBS1,TNFRSF21
FATTY ACID DERIVATIVE METABOLIC PROCESS (GO:1901568)	0.09252406	0.22921039	EDN1,SPHK1,CYP1A1,PTGS2,MGLL

APOPTOSIS SIGNALING PATHWAY_HOMO SAPIENS_P00006	0.08695652	0.230252	JUN,PRKCE,BCL2,BIRC3,BCL2L1,RELB,MCL1
RESPONSE TO AMINO ACID (GO:0043200)	0.08298755	0.23028962	IL6,EDN1,PDGFC,SH3BP4,EGFR,BCL2L1
POSITIVE REGULATION OF CYTOKINE PRODUCTION (GO:0001819)	0.09462617	0.23032522	IFIH1,CD274,IL6,DDX58,DDIT3,SAA1,BCL10,DTX4,THBS1
REGULATION OF CALCIUM ION TRANSPORT INTO CYTOSOL (GO:0010522)	0.08563135	0.23041616	RYR2,CXCL11,UBASH3B,BCL2
REGULATION OF MEMBRANE POTENTIAL (GO:0042391)	0.09108041	0.23044696	RYR2,EDN1,JUN,PID1,BCL2,PMAIP1,NALCN,BCL2L1
HAIR CYCLE PROCESS (GO:0022405)	0.08701564	0.23047684	FST,TMEM79,BCL2,SOX9,DKK1,EGFR
REGULATION OF MITOCHONDRION ORGANIZATION (GO:0010821)	0.08465245	0.23070076	PID1,INF2,BCL2,PMAIP1,IER3,BCL2L1
RESPONSE TO MONOSACCHARIDE (GO:0034284)	0.08281998	0.23071659	TXNIP,IRS2,THBS1
MOLTING CYCLE PROCESS (GO:0022404)	0.08835341	0.23162687	FST,TMEM79,BCL2,SOX9,DKK1,EGFR
SIGNALING BY THE B CELL RECEPTOR (BCR)_HOMO SAPIENS_R-HSA-983705	0.08868895	0.23339921	IRS1,AGO2,IRS2,BCL10,IER3
NEGATIVE REGULATION OF SECRETION BY CELL (GO:1903531)	0.09134287	0.23459958	IL6,EDN1,IRS1
POSITIVE REGULATION OF ERK1 AND ERK2 CASCADE (GO:0070374)	0.08635465	0.23473974	IL6,SPRY2
REGULATION OF RAS GTPASE ACTIVITY (GO:0032318)	0.10071514	0.23494364	ERRFI1,DOCK4,BCL6,SPRY2,EPHA2
FOCAL ADHESION_HOMO SAPIENS_HSA04510	0.09090909	0.2349988	JUN,LAMB3,ITGA2,TNC,FN1,PDGFB,VEGFC,THBS1,EGFR,CCND1,PDGFC,BCL2,MYL9,BIRC3
PLATELET ACTIVATION (GO:0030168)	0.1016731	0.23513596	IL6,SAA1,VEGFC,STX4,THBS1,MGLL
REGULATION OF ENDOTHELIAL CELL PROLIFERATION (GO:0001936)	0.09042905	0.23761246	NRP1,JUN,BMP2,PDGFB,CDH13,SCG2,THBS1,F3,FGF2
PANCREATIC SECRETION_HOMO SAPIENS_HSA04972	0.08422665	0.23932806	CPA3,RYR2,PLCB4,RAB27B
REGULATION OF PEPTIDE TRANSPORT (GO:0090087)	0.09389067	0.24230587	SNAP25,PCSK1,IRS1,SPHK1,CCL5,PRKCE,IRS2,PTGS2
FATTY ACID BIOSYNTHETIC PROCESS (GO:0006633)	0.09723208	0.24232142	EDN1,ELOVL2,LIAS,PTGS2,PRKAB1,MGLL
NEGATIVE REGULATION OF SMALL GTPASE MEDIATED SIGNAL TRANSDUCTION (GO:0051058)	0.09455588	0.2424259	BCL6,SPRY2
MULTICELLULAR ORGANISMAL DEVELOPMENT (GO:0007275)	0.09295612	0.24271227	SPRED2,HHAT,RNF114,IFRD1,ENC1,EPHA2
APOPTOTIC MITOCHONDRIAL CHANGES (GO:0008637)	0.10320285	0.24358886	JUN,BCL2,PMAIP1,BCL2L1,IFIT2,MCL1
SIGNALING EVENTS MEDIATED BY PTP1B_HOMO SAPIENS_BE498A9B-6195-11E5-8AC5-06603EB7F303	0.08382353	0.24534336	IRS1,SPRY2
LEUKOCYTE ACTIVATION (GO:0045321)	0.09648127	0.24929076	EDN1,JUN,CXCL8,ANXA3,PRKCE,DHRS2,ITFG2,HDAC9,RUNX2,RELB,MAFB,BCL6,CCL5,BCL2,IL7R

Supplementary Table 3:

Raw data of miRNA expression.

		Group1	Group1	Group1	Group1	Group2	Group2	Group2	Group2	Group3	Group3	Group3	Group3
		Input1	Input2	Input3	Input4	NclKD1	NclKD2	NclKD3	NclKD4	NclKD1	NclKD2	NclKD3	NclKD4
897	U83CDbox_001	67.44868746	68.77536607	62.76830045	47.81700113	153.6774529	146.5732831	142.903985	154.5223785	116.9900125	110.8808665	108.2782146	102.8762522
896	U82_aj011568	40.89041809	37.79429426	22.81604858	10.74948736	44.78285728	5.378592967	18.01797825	9.164173956	22.70547518	19.64471836	19.88498746	9.416331498
895	U8_001	39.24267485	37.13218104	32.88927544	16.2130234	28.326549	14.24731818	15.50281563	10.66794771	33.50839817	18.55319493	19.03913452	11.62687697
894	U6_af452638	31.70166541	33.98272458	41.95341805	0.012344103	21.37422494	0.047265655	0.052740788	4.021322289	6.863982597	10.59720714	0.016877215	4.39236238
893	U50_ab017710	462.6656508	508.1176141	532.2471537	480.9329029	729.7654124	777.179559	789.0404181	808.0141292	280.5302902	312.2453173	316.5141809	306.2849016
892	U30_001	1734.074481	1978.197844	2059.092083	1660.269689	2251.230933	2146.568363	2122.627553	2258.406855	1547.480853	1603.292268	1541.452053	1435.295119
891	U3_001	33.90944961	32.61367101	23.28490696	0.013104504	33.87618043	0.047785004	0.052513007	8.706495158	9.60735259	15.35623126	1.384967369	0.016150911
890	U23_aj007015	36.63798641	35.84827636	28.75852463	12.50398964	107.6572678	81.7512292	90.62575979	87.13381167	41.15187256	44.8372359	37.46967308	24.30284011
889	U22_001	60.84735362	54.44057917	43.77660585	27.52995803	68.03018793	45.95446069	34.32094726	31.43042535	46.12004213	50.98263474	40.99903485	33.54356262
888	U20_86604696	33.78484267	31.03524902	24.02366368	0.014790833	26.6481993	0.048006962	0.048265884	0.040755085	17.65467269	11.70445534	5.953796554	0.014532577
887	U18_ab061820	340.4752064	408.7397035	416.736612	336.848543	803.4266077	931.780624	866.0960386	930.4688024	491.0959518	546.6703951	517.2830278	434.3626019
886	U14_001	185.6430706	291.4367121	264.067018	201.6487451	321.9690589	423.4282911	374.313511	369.8261322	109.7193821	139.0457031	123.976817	102.073198
885	U13_001	213.4968931	261.0981565	245.1361252	192.8007769	504.633697	523.4859223	501.693902	513.5423638	258.0825862	268.9971268	253.9400069	227.9339729
884	U1_002	13908.5085	15052.77699	15001.93752	13346.05126	22533.88289	19649.87804	18747.51971	22633.98732	14467.93963	13784.97379	13528.05241	13915.6092
883	U1_001	4166.898011	4554.742227	4515.620852	3788.383849	5729.031092	5132.318279	4801.684543	5794.813868	2392.348208	2224.687531	2163.651464	2188.015547
882	snRNA-U1-related	35.72535506	30.19861771	24.40894188	8.801480834	67.21063911	48.47124647	49.23302103	56.1359924	54.39938223	55.81669909	49.44889548	34.98842067
881	Rrna-320-300	39.24985058	30.3818648	25.66877286	11.03274373	63.57748337	31.95479985	45.82196953	41.20639442	36.53104157	39.55380732	34.48178819	30.70533102
880	Rrna-21-1	41.54147053	37.88115113	38.20112513	20.98225694	49.88885662	27.47454969	23.32881823	32.75376301	27.40965591	26.56633761	21.7247067	13.31826711
879	Rrna-123-103	48.17917045	46.67770123	35.75524113	17.38778009	37.91264522	48.20073006	38.42036925	36.6871173	51.79987222	35.48841056	41.84120046	34.45599449
878	hsa-miR-99b*	6.551983262	0.011562238	20.19991317	35.0591933	18.86614422	18.32106602	28.55661335	25.88304276	27.28547445	28.96691143	33.46083341	20.11974568
877	hsa-miR-99b	178.788894	211.602309	221.5306672	176.7766824	728.574133	728.680065	722.0518488	756.2724934	778.85314	791.0343003	760.8939891	757.3019659
875	hsa-miR-99a	151.8854014	168.4611027	199.2576647	127.4614304	270.4247373	274.976437	329.1559089	202.4106256	246.8828832	291.8886025	275.1036816	240.4176894
874	hsa-miR-98	412.8585932	427.3364367	376.8729345	354.030645	624.1102178	1293.088265	640.294531	452.7842093	542.7629501	1137.224043	590.9053082	456.5914795
872	hsa-miR-96	244.4506888	458.2594029	376.3985906	347.4792714	24.69178684	2.493961387	21.37372511	0.043430505	22.13953766	23.20513394	16.53397378	44.76029616
871	hsa-miR-95	9.683329146	26.32993836	44.72375078	17.75642741	21.39274074	3.272935348	20.91512452	4.044696367	20.6905686	28.94825673	16.94547009	24.50526131
870	hsa-miR-944	3.289080492	13.1230805	43.45016901	6.551294975	0.042586546	8.582852466	4.229939464	0.445542439	17.88755093	10.29260568	15.67302233	14.43792186
869	hsa-miR-943	0.212497513	21.23364552	33.93399666	14.97017598	8.617329609	16.26035365	27.99350435	17.58162685	42.23072391	18.44952332	32.45695978	34.26193168
868	hsa-miR-942	0.011878864	15.44542622	34.93686709	11.81139913	6.634691177	14.35969447	19.47499448	0.03922043	21.72672625	8.830187739	18.53856919	15.06358489
867	hsa-miR-941	2.504759929	19.60164109	35.91910874	9.204219993	5.4383149	20.71155983	30.4955712	8.222080919	39.71224486	23.31717377	27.06733418	43.7300851
866	hsa-miR-940	70.19517117	95.66255008	116.7326775	104.888993	148.9552114	153.8635859	152.3593719	156.3849176	356.9367509	332.11338	317.152653	346.7281728
865	hsa-miR-939	69.25852833	93.75880077	107.157365	86.81542895	92.64333551	85.91686718	86.49790359	62.77531348	146.2397394	118.1042339	125.371239	125.05908
863	hsa-miR-937	0.011455707	17.47674511	41.57107685	20.21625434	24.07379003	30.09093245	27.56770512	23.21080691	25.77451288	20.96405333	35.36317639	46.63312133
862	hsa-miR-936	230.7049885	209.8173624	203.0961184	223.6155524	37.42037481	32.23978012	43.92309188	30.45281519	72.0347233	61.3058881	67.25191543	73.18934467
861	hsa-miR-935	0.011348264	14.46380776	32.81536918	9.32727446	27.79639243	29.43532655	30.64290443	19.68517978	28.18072245	31.01260006	29.80823832	58.75404022
860	hsa-miR-934	0.01170183	19.42604051	39.05821565	16.51609997	20.7669025	23.422883	33.05740186	20.64373065	14.99052674	27.31181801	29.44587894	66.19140533
859	hsa-miR-933	32.56211336	37.97842325	69.42352733	49.24957672	50.39253675	71.24311793	58.62070191	50.06314844	85.3748592	86.81992656	101.3040383	129.2869216
858	hsa-miR-93*	6.267614474	28.11188087	42.45959585	21.16843494	26.46835398	31.07165615	36.89813388	30.24208454	0.360829853	34.57269196	40.55224125	56.05565137
857	hsa-miR-93	291.8094358	382.253673	406.8245702	359.3033661	1031.645522	1115.603728	1114.784128	979.0647723	1004.665968	1126.715612	1086.425529	1057.381996
856	hsa-miR-92b*	1.046198679	10.62719668	34.8933928	17.68551912	41.37099879	29.93176509	28.76913529	16.86724814	63.99962448	18.74087335	39.23258431	61.95702296
855	hsa-miR-92b	1139.769608	1368.166811	1379.137777	1494.512595	1850.657678	1986.429096	1970.795799	2072.452491	1596.172271	1666.019151	1691.889703	1745.265521
854	hsa-miR-92a-2*	0.015751071	7.777871331	39.01514093	20.62803487	27.34023243	22.29398217	14.27135202	5.151307256	41.81714138	26.8406664	28.37449682	32.85680877
853	hsa-miR-92a-1*	9.179041229	27.15951354	43.05322536	28.71172601	162.3396718	123.4512538	151.5804343	112.453412	105.9819228	103.4033766	125.2592853	99.90187304
852	hsa-miR-92a	3287.987071	4526.780338	4806.709438	4756.826627	6358.411202	7205.859615	7089.173874	7305.748984	5138.775293	6202.892126	6199.126342	6166.05447

851	hsa-miR-924	0.012520606	4.067282968	33.29717641	9.335563917	16.52344137	18.02969844	22.91451098	1.026576852	18.6829616	271.8107142	29.59666146	38.45654373
850	hsa-miR-922	0.016503569	24.11432306	39.56528926	12.34380905	3.856190402	14.11150093	0.060579102	10.21874543	41.04110224	22.58863978	17.40360141	30.13188239
849	hsa-miR-921	0.01556946	11.74317399	41.6211971	15.12844511	38.29718878	23.02904944	19.43449319	6.109019442	56.29980409	29.47604318	27.63571129	27.931255
848	hsa-miR-920	0.015662952	7.800870735	32.80667238	16.21484898	26.67022085	23.50899704	21.5203559	21.77614858	69.71776368	33.83897881	23.98480032	39.09686498
847	hsa-miR-9*	7.822919732	29.11500162	6.69379502	33.27645816	8.734802805	19.87570774	10.77816266	0.0427737	14.66877003	21.48737513	12.69814033	82.59249347
846	hsa-miR-9	14.34686761	31.56641428	11.45095588	32.54766966	7.734966025	21.58965902	9.458234433	0.044293473	10.09314792	26.85482405	0.018392275	11.35028331
844	hsa-miR-892a	13.3424009	32.4100327	5.435896086	31.50943544	21.5825	17.58301127	24.57486372	0.04420528	21.98827269	20.94038484	14.68889044	1.477021866
843	hsa-miR-891b	4.032459013	28.23070204	0.116285406	33.11589905	6.967683569	12.42341897	12.78951068	0.049045198	18.05937069	1.268369019	10.272033	0.015902048
842	hsa-miR-891a	11.07939871	41.51820931	20.7394402	46.49562888	26.23322591	31.48041108	29.37319433	4.546463807	45.83832718	17.52262446	27.9960267	10.91514764
841	hsa-miR-890	11.34133306	33.03088137	8.938456603	42.17800847	14.63262733	19.06375347	15.165535	0.043264032	15.30095345	9.878768494	10.6316494	4.880557796
840	hsa-miR-889	1.31744232	27.3079882	7.763476469	40.07537602	21.81353975	26.45329217	5.368516092	0.050038976	0.37942344	12.29365762	1.528467657	0.016147978
838	hsa-miR-888	0.011974703	27.08721366	12.56124599	37.88579199	15.6143329	25.47100645	0.047198053	0.043743563	22.45608302	13.74483052	15.44985693	2.424163979
837	hsa-miR-887	8.476022476	26.15539231	13.68775563	42.01395781	41.72562503	40.95689139	28.22646224	13.86030017	45.41990642	33.55644836	32.31724599	35.45314917
836	hsa-miR-886-5p	71.65904614	95.29648992	76.07889539	106.9108366	271.9328967	244.3926972	228.0538691	209.0630572	234.0415818	207.6337093	209.3783631	196.2659027
835	hsa-miR-886-3p	7.13304534	33.33190076	16.82854031	44.01574424	139.6722206	125.9100939	107.220831	107.1999994	122.8808514	87.55957161	91.01332908	85.75679084
834	hsa-miR-885-5p	19.54347768	25.2268194	11.47290381	49.8518257	42.48178066	39.57703922	34.30422608	22.11276946	31.49445448	36.02248663	23.53440475	29.80268509
833	hsa-miR-885-3p	10.38770668	27.86556985	17.87511523	43.64593922	30.44076936	35.40450226	9.745259729	13.8141005	41.58151031	19.82375061	21.0839707	15.3472024
832	hsa-miR-877*	12.8849003	27.31209493	9.04859523	43.54376029	52.2067131	30.38446792	33.85227675	30.15360186	12.37138927	28.82288379	15.32896103	28.17695796
831	hsa-miR-877	64.75174539	69.86834383	53.85716844	82.7588275	146.2831282	120.8524582	128.8390647	110.9777274	156.3097897	133.9522921	116.0460424	147.5485635
830	hsa-miR-876-5p	0.031486594	25.91015836	0.522671714	40.49189337	18.96272255	31.02266747	1.966289237	0.044942696	16.31755018	11.87206471	9.804918892	11.63681594
828	hsa-miR-875-5p	0.011575155	26.65826707	9.787052383	36.78169774	32.15901359	53.42382067	18.82521502	7.012691761	22.15785908	25.5380739	24.54065785	22.46790131
826	hsa-miR-874	114.6578369	108.413765	107.8879474	123.2321477	54.82708202	51.21500338	42.43436873	35.13719763	58.44313846	44.938471	47.55629116	47.49270041
825	hsa-miR-873	0.011996566	29.05295458	7.582594045	32.0525338	31.11290182	31.07567059	7.818293664	0.042828865	17.06915283	17.70289765	16.31609514	17.74270873
824	hsa-miR-802	5.370042311	29.8820581	1.757112969	30.25091281	74.47272827	38.66426178	0.04932604	8.965431775	61.29769149	21.1818865	18.87268719	22.0526662
823	hsa-miR-770-5p	0.015389078	28.12328004	0.012193215	38.6523247	59.94090227	38.89439184	17.85106308	4.334015773	37.99291386	40.21432784	27.09125989	31.4084953
822	hsa-miR-769-5p	10.08351544	24.21218105	8.148916861	47.00315242	59.82223589	49.78143774	24.6133809	25.48699337	55.53404028	36.12262313	17.50542614	18.3965061
821	hsa-miR-769-3p	311.5669568	285.2586284	278.3323543	358.7014154	45.69404824	34.153993	25.27320999	0.053005155	90.80745186	57.95252887	32.91558234	27.82713678
820	hsa-miR-767-5p	0.01504981	30.59942833	12.86017564	36.72791211	125.385578	79.42091349	39.49175061	32.59048985	84.06642241	44.09294243	23.95640199	37.74434408
819	hsa-miR-767-3p	20.73795739	37.63698172	5.247750982	39.7009209	109.6590877	62.17053582	29.92563671	14.33195717	118.2142697	92.48741745	91.40795912	79.59936004
818	hsa-miR-766	19.69133872	40.13043793	24.47685864	51.49091222	160.535089	113.8988076	112.4431537	87.86992746	50.00043313	44.12577063	32.5154585	0.016797915
817	hsa-miR-765	245.1352723	206.2021554	312.1087682	362.9797893	44.34631871	36.81904547	27.38646369	1.344948157	62.64934845	51.32475807	42.10331714	16.98668612
816	hsa-miR-760	23.33210159	9.448717905	42.37716698	55.18571032	45.51700837	41.73842055	24.40299443	11.37387609	33.16900937	33.19731716	8.318968961	5.772258024
815	hsa-miR-758	21.92617687	0.013446921	34.33230427	58.42654183	40.9004884	27.4724341	8.418634112	0.042028115	62.45485013	53.12404392	37.82717443	20.54571259
814	hsa-miR-744*	18.15833267	6.237708081	38.05372071	51.95790182	63.11750716	42.16977934	37.11717997	29.71052522	61.81129806	55.75296892	35.72975202	30.68882359
813	hsa-miR-744	27.37582229	17.4318116	43.77027852	55.37069945	77.87265114	65.83778504	53.63526378	48.2016278	1305.360524	1364.552582	1278.219433	1267.626956
812	hsa-miR-720	581.5618213	587.3813659	691.1782231	693.6262554	1689.743017	1701.348937	1532.619144	1514.017857	23.18265763	0.909649154	7.028128342	1.218682373
811	hsa-miR-7-2*	21.80833333	0.013093738	30.76442204	50.97319625	38.47997944	23.22434918	7.000555521	3.831404995	116.606289	72.81808414	95.36386723	68.01361365
810	hsa-miR-7-1*	40.81946312	20.78551548	50.8939037	53.05688558	117.5332464	119.600961	110.5502624	92.63942299	29.06634319	4.070387713	0.018930901	1.953099851
809	hsa-miR-708*	18.19706417	0.012318341	39.662228	49.27061295	30.43216186	15.08326498	1.435551261	3.553267262	33.43614883	16.06354989	7.344746865	4.424872597
808	hsa-miR-708	15.42753242	0.012018659	39.85755608	55.92792191	33.47968024	12.09734048	0.138121192	0.042073089	795.9577989	1662.063115	1628.995033	700.4695783
807	hsa-miR-7	13.79156262	3.806838233	45.82404474	48.07716185	836.3932575	2424.374349	2226.871959	752.0395467	69.03784637	56.77399283	46.08978057	48.21302806
806	hsa-miR-675*	26.08577064	0.433051818	29.68990719	47.5924566	77.7105311	45.41023034	58.04334581	50.21410317	35.65781745	5.660495882	17.36168397	19.07576173
805	hsa-miR-675	27.5286901	0.532092128	40.11119059	49.60257784	37.76240321	17.83646613	21.75229789	11.69377689	180.7787104	107.9244134	125.8522276	121.9367606
804	hsa-miR-671-5p	342.7838453	300.8243898	344.2156412	379.3847022	116.3105965	102.5372092	94.8807658	72.30868403	53.8793016	9.873976055	29.87654303	39.19603689
803	hsa-miR-671-3p	36.12025516	1.053292541	37.71196947	50.09789613	56.70678164	35.04421516	30.76485509	25.51390041	91.2777475	53.86947655	68.68959686	68.30640022
802	hsa-miR-668	25.15323831	3.339881255	37.76665542	48.928378	89.3400834	83.73183009	63.18026972	74.90358845	107.683068	95.25803825	105.1839305	107.3985684
801	hsa-miR-665	34.68340689	9.842473796	43.64984695	51.53910063	47.62268137	31.49816581	33.92747326	27.3746039	13.21307408	1.931251662	12.37494044	18.76241101

800	hsa-miR-664*	23.01846502	0.012520325	35.31353957	51.45533446	46.42564817	13.2301744	16.18569659	30.00623918	27.48653925	22.46910113	21.55599471	31.88878557
799	hsa-miR-664	24.01988704	3.856282906	40.63814035	52.56791824	48.95594681	24.80963306	19.23543753	36.62968467	53.04798646	18.48634029	40.56376382	41.0891367
798	hsa-miR-663b	8.863540375	0.012107831	25.37740607	39.45838552	54.50247149	2.897163647	24.95402862	25.27686576	73.81556198	31.97261702	38.69585617	70.72633492
797	hsa-miR-663	1195.718178	1278.071307	1585.384819	1366.911192	68.51270907	56.39942526	62.83413567	57.45232446	42.42799267	26.60171461	50.59162859	43.14754319
796	hsa-miR-662	16.65040365	0.011358634	41.65886973	42.94022987	68.27639767	32.06280252	56.31986124	39.56343569	14.27226507	2.12317366	29.75921409	20.56093685
795	hsa-miR-661	14.72298904	2.420914221	41.14590266	37.21952891	34.25681847	19.90935042	19.35561983	25.30561384	66.57518091	7.747955348	58.34885814	36.47169391
794	hsa-miR-660	16.52923108	3.112991818	44.49567127	52.96770707	99.87692476	30.72399644	68.57363889	42.85120312	52.84287793	6.478546028	19.86405729	39.15115984
793	hsa-miR-659	36.99826537	13.72188801	63.0662754	66.93388674	37.64629215	10.34353343	0.050206513	30.76278523	26.62577613	0.02003929	40.0285438	24.01894669
792	hsa-miR-658	41.1707605	20.41289269	72.07663112	66.11434597	56.58855922	14.19001952	20.09999927	17.58746134	71.88469883	44.29602356	55.00214446	64.98861586
791	hsa-miR-657	10.66460593	2.236425506	30.90270124	46.2690213	79.22438121	49.49604787	53.68614755	59.80926847	79.69061129	6.505417593	34.6307666	37.10110799
790	hsa-miR-656	4.778613695	4.445693679	34.42856797	46.06474378	112.1215752	22.21666442	24.59240466	24.14935537	51.26386717	0.088401773	18.36173739	23.57586534
789	hsa-miR-655	0.015587103	0.012695994	29.26768982	39.35597818	38.3032691	16.28503936	7.067876665	12.44505576	65.13370725	7.69091604	35.72081985	55.16440202
788	hsa-miR-654-5p	329.4941604	189.1979744	224.7746408	191.1941053	63.955834	19.57640804	0.051988821	21.23173204	19.198736	2.51263242	14.39935428	27.33595755
787	hsa-miR-654-3p	2.98627042	2.539601863	26.16260038	37.14301836	20.03895866	30.68936264	36.5714882	19.39930744	5.466039288	0.017178642	0.020283452	3.728472684
786	hsa-miR-653	0.013011984	2.574715797	40.47875433	41.02235697	9.817297482	11.18591297	0.065493337	9.668395329	52.66689939	35.55595664	58.77665078	36.97851676
785	hsa-miR-652	4.825998678	9.52270226	35.65649671	31.54726779	38.72508437	44.92603734	45.38685086	38.84751319	17.396506	9.947668754	8.199708702	27.40491186
783	hsa-miR-650	6.506453267	7.805385156	28.31051749	70.89705909	21.1022288	8.198889762	17.52182196	10.84355281	4.445361049	0.018234135	8.509783313	1.363637703
782	hsa-miR-649	0.338699658	3.334233618	29.88811033	32.7090054	7.094064414	16.5988287	19.78721964	2.85954729	19.87165747	14.10096071	39.09365995	21.68561511
780	hsa-miR-647	0.011022189	2.832881521	27.59848947	31.26530607	39.50126164	41.62440249	45.81086794	46.25564538	47.67878907	48.93424463	65.53235931	63.10740469
779	hsa-miR-646	0.011720589	27.36761271	34.69447044	19.0983651	22.88797464	20.8917673	23.93136122	3.623011439	14.37246445	28.97871148	23.18625347	791.0957845
778	hsa-miR-645	2.239647299	28.60915812	28.96349781	32.70088514	11.35579142	15.880625	4.375529984	0.03825043	5.344236018	1.264364605	9.96745236	44.2385996
775	hsa-miR-642	1.664061753	24.92799194	35.50592982	37.6806751	26.39919665	27.09687425	27.63941619	8.804723711	6.44822891	21.20802097	22.00399263	35.34608471
774	hsa-miR-641	13.13793173	31.53810804	41.82969922	43.68969549	46.86904669	74.81530653	47.86530392	47.47048472	15.01898496	12.65759173	0.018230569	11.19253338
773	hsa-miR-640	0.011653985	32.91832646	35.15575129	30.45543288	23.90126979	18.30815842	23.35493807	8.047778228	40.02356015	42.61202064	34.99360286	42.6531753
772	hsa-miR-639	1.310889475	28.92447059	24.66557219	40.40788138	8.210455513	30.0397756	15.63947098	11.3221949	7.518149621	24.33040329	11.21083995	4.367443195
771	hsa-miR-638	6907.690623	8940.755606	9397.015433	9124.047692	874.0369182	915.4639442	846.1210106	946.0351341	15.42769756	14.91615502	8.032266231	0.016671649
770	hsa-miR-637	13.64907617	30.12952027	36.24579099	52.29896538	15.30074997	24.47937984	27.82288816	12.92585647	738.9310873	804.3193154	785.0826703	772.6977109
769	hsa-miR-636	17.85328808	35.18658968	29.16473945	41.6333964	49.05261388	67.99378629	44.77452385	34.95389658	9.725304734	35.43635985	1.553300071	11.13841268
768	hsa-miR-635	0.011986472	41.64244176	32.93787735	32.94322336	16.05581572	43.61790544	10.13817426	1126.362233	49.2377854	50.60288766	40.29238834	40.7872027
767	hsa-miR-634	9.487355214	32.45198589	38.9743473	36.58919892	47.91126314	60.2206024	49.13762741	47.40285712	4.849034591	32.68519148	9.68320254	8.806241051
766	hsa-miR-633	0.018068122	26.35002476	38.43877663	31.62442588	3.239036432	26.58170378	17.27902176	4.03441691	40.14962798	40.42217784	33.91258523	48.49274593
764	hsa-miR-631	6.171159122	26.33159667	34.82595168	34.85726576	40.88351338	47.98709655	34.51527194	31.45456417	0.032932111	0.022266348	4.495189937	15.62887997
763	hsa-miR-630	20.02561497	35.52637485	52.08250755	50.065015	1.738911559	44.25367706	12.49235029	20.64180657	27.91087099	25.16976242	37.22590041	43.75414935
762	hsa-miR-629*	10.17828988	38.49292549	49.1945327	51.08868125	54.13935909	81.2045151	67.25214021	65.82409192	15.30337321	16.78433271	29.73639526	31.98571783
761	hsa-miR-629	63.13510377	96.95441789	115.6153881	106.9629716	144.6674896	179.6287041	166.7405074	142.9026981	37.50116072	35.33483033	60.07520216	67.66559358
760	hsa-miR-628-5p	0.011967462	19.48381621	25.52272881	34.47862675	27.26085181	70.03269751	28.60576658	21.55058518	128.0540305	117.640717	122.044097	119.9667757
759	hsa-miR-628-3p	8.00509212	39.26637462	43.14251856	53.12563968	37.01919179	70.90049011	47.18376511	56.81817842	10.46162943	14.43802424	25.94194709	34.1400581
758	hsa-miR-627	6.273172684	31.54375981	38.61882669	46.35987297	11.86093091	26.87016869	25.86209056	8.234408207	31.04905585	29.8044077	46.80695907	44.04548125
756	hsa-miR-625*	17.72432911	25.47646113	33.83709582	40.71929097	30.55792484	58.93600466	47.22901124	36.92543823	25.98311821	3.308572894	27.65377972	31.83636196
755	hsa-miR-625	10.41410344	33.68600681	30.50597621	56.39175109	24.04703367	36.35193087	36.50902963	21.99682101	62.16842332	56.78129808	47.87056248	59.93391043
753	hsa-miR-624	0.0195008	11.4270772	22.19786627	25.81943034	0.099292453	36.93444963	9.957485602	5.54496767	22.21408691	20.89611656	30.63942998	26.91943458
752	hsa-miR-623	6.134231749	32.02080746	32.0200832	42.81568918	36.62611233	31.08226595	31.87057421	19.03999474	36.46950224	6.04996737	16.53564242	14.04248075
751	hsa-miR-622	0.013231689	20.82863039	22.36353707	27.10272938	19.02839402	41.66852947	29.42648402	24.10508599	40.63659919	31.38143024	38.83201686	42.48586341
750	hsa-miR-621	0.016989506	16.81028669	27.64751044	22.77288753	42.50981303	26.96012133	22.55953036	10.90678847	21.34474219	38.55215366	26.45177396	31.51773352
749	hsa-miR-620	10.07511606	13.79841152	32.55645574	39.87267916	17.26233388	15.03622446	0.403086733	0.042251162	19.31810721	25.0882675	7.330375555	86.08136841
748	hsa-miR-619	11.28421896	17.1598215	28.85444176	51.34531129	43.56141812	27.18353622	22.66100819	0.047794999	11.12305091	34.5345953	4.820172079	0.015498811
747	hsa-miR-618	15.09820779	16.75981297	26.54507116	55.04627752	23.78005843	9.598678319	5.662792869	0.049314617	1.36189695	18.36105339	4.784019323	0.015905829

746	hsa-miR-617	18.36887736	23.2875817	29.01991612	49.47656631	62.56203464	61.04511199	57.24632566	29.18124395	46.12510308	62.42743984	40.55147546	82.92738973
745	hsa-miR-616*	8.03953516	20.08703115	21.84877575	44.18929993	47.37591499	23.42996706	18.37633349	11.14925038	17.4577894	18.23381507	7.53403726	104.0345073
744	hsa-miR-616	5.800100882	19.03134011	22.34546198	53.17125449	31.81602614	31.24288231	20.4231933	0.040311836	29.60769285	25.9272244	22.82475714	82.17326163
743	hsa-miR-615-5p	14.50612449	24.53799515	28.11415351	49.09763987	33.83591882	37.76768285	23.52788805	3.78543337	32.572869	34.57200797	20.99205022	56.53742976
742	hsa-miR-615-3p	20.69282785	32.60994751	39.62695937	50.69597866	59.94294281	41.74347741	37.69010683	30.39157287	35.00322825	44.5553228	15.56015925	17.51101098
741	hsa-miR-614	8.803455078	19.91635019	25.88179764	50.34209018	46.88669895	26.39560362	13.79822715	0.044598066	25.88978887	30.97701034	17.05891848	4.400020542
740	hsa-miR-613	7.804369517	26.0121304	25.5300248	40.92634722	46.26598444	13.25163237	19.42823528	4.099656919	32.27683798	27.63057642	31.30592567	10.49758814
739	hsa-miR-612	16.40101578	27.5336843	33.46358205	53.862248	28.423441	13.8894653	26.46103377	0.042899089	21.64974693	23.62331464	0.019585208	25.99655282
738	hsa-miR-611	24.50535696	23.82366501	32.24989533	57.09595996	56.60266641	23.0442202	27.76570514	9.512271887	36.82683683	27.44287117	13.26253376	14.49109461
737	hsa-miR-610	2.971501607	16.74879342	30.45997483	47.98611625	33.71935603	16.29413849	14.94772686	0.044468711	39.25001959	17.05196675	3.054297406	2.523301545
736	hsa-miR-609	0.011609878	15.20538155	27.16213478	47.74240591	33.16622504	17.12814152	15.94195237	0.048380762	24.83223784	24.30563536	4.071073886	0.015669516
735	hsa-miR-608	7.877563584	21.80659474	28.16078219	42.77474673	43.22555772	29.09300844	12.52299802	12.00054152	23.33018389	13.90987646	0.01818111	14.33185243
734	hsa-miR-607	10.12338406	18.01072464	22.477085	44.26235124	27.35298796	21.76293085	13.04636509	0.052701817	24.49250113	0.245310355	0.01881087	0.016858814
733	hsa-miR-606	0.012137287	22.22275925	26.38042444	47.91189619	25.95653566	24.01991223	4.902859066	0.051101036	17.08836588	0.018136549	0.239744822	1.348474816
732	hsa-miR-605	36.24847253	55.85786708	60.59958234	69.25854764	84.97316283	73.65430603	77.75198871	65.58442149	312.3659416	320.9981384	285.8642355	345.1772516
731	hsa-miR-604	8.972682059	18.38170409	19.69923086	46.86415535	37.34445942	18.52572317	15.24802273	19.35610439	29.91369288	19.09877307	10.86970086	14.46936499
730	hsa-miR-603	1.935654748	18.82156097	18.04949352	46.66534314	40.89099838	30.12996706	28.18103138	2.757153197	22.11920047	0.364806581	0.021937785	0.023020377
729	hsa-miR-602	42.94262537	43.23482303	48.51049844	78.29265403	64.23258018	46.20388707	32.46644527	27.30569735	51.93008718	38.43152239	37.53065846	39.16904076
728	hsa-miR-601	39.955921	45.6166914	57.82152766	80.4179395	104.7280445	91.2414231	99.14613379	79.26329054	91.32124668	63.91777428	70.32920301	75.36453701
727	hsa-miR-600	3.903967176	23.11329658	15.28337753	46.70302119	36.62454376	19.93570306	27.0980563	0.04744877	20.74322186	10.73298387	13.89768478	9.009316071
726	hsa-miR-599	0.015361841	14.64053687	13.46733364	34.59071075	65.07560785	11.13825533	17.68961105	0.308025103	60.40805988	3.001873331	0.019980495	3.677789555
725	hsa-miR-598	13.53488429	18.09776744	17.76107992	54.81373657	66.73265427	19.35022908	25.61866551	2.602055323	49.37519541	0.017622726	2.367910075	0.01796761
724	hsa-miR-597	2.122146012	13.8716638	17.03938711	47.23575364	38.01501687	23.62439225	22.88895887	0.945635312	23.56938203	2.159919359	2.472163046	9.505001969
723	hsa-miR-596	15.13414367	23.52662564	28.98523081	50.14935891	148.0802984	113.9032493	124.3819875	108.0012822	105.9506477	90.23025827	94.19594584	99.78472321
722	hsa-miR-595	3.272558068	17.86337015	20.071879	48.33551006	94.1548471	25.69605138	18.67502488	0.049509456	46.3126519	23.7630011	3.520142882	16.83387757
721	hsa-miR-593*	0.015554591	25.73236905	19.84671375	47.53981316	85.73707787	29.16941556	30.13011771	7.759529775	12.60058396	13.1009293	0.020423979	5.210109054
720	hsa-miR-593	164.932539	186.5735713	148.6181564	240.0077171	92.47587474	30.32872919	30.51594554	12.6656499	24.69328622	16.63652653	11.20951587	17.51924429
719	hsa-miR-592	14.37406446	14.07573932	57.81896133	46.46746947	23.11639696	24.24865114	0.060042676	0.048201595	4.454043941	0.020346334	0.018785295	0.015623989
718	hsa-miR-591	13.36562118	13.90986092	39.2603918	38.24525759	15.49301944	3.92990936	8.703831077	0.045360464	15.66871265	14.69242533	10.38488631	0.014865017
717	hsa-miR-590-5p	17.50789336	8.241702205	53.78339513	31.36532719	29.10766937	24.5204908	0.053913162	13.33585389	9.777016231	16.21725222	1.701092399	0.016504947
716	hsa-miR-590-3p	10.32828907	0.013637182	49.84231497	45.27294698	21.11228072	8.222172631	0.054525987	0.047466699	1.538155433	0.017730068	0.571223995	0.015425357
715	hsa-miR-589*	24.12644674	19.93426032	59.98667914	49.249419	34.74573091	44.41554681	11.06025781	6.096809801	14.19221117	17.08532617	0.018983693	7.757638581
714	hsa-miR-589	9.641529315	1.923034407	46.29288881	35.30625803	39.95350036	9.486961237	12.47098248	9.558203574	20.37783335	15.39490611	3.847753343	7.670147018
713	hsa-miR-588	14.46741555	5.099062071	43.4702041	46.75699425	24.47907676	22.76785927	1.548490005	0.04639425	13.29264206	0.019588232	1.143191782	0.015125267
712	hsa-miR-587	11.53567988	8.786195767	49.13892488	37.41191396	18.52464707	12.96967063	0.055838352	1.981635207	9.157730237	12.58605243	0.017689329	3.662911328
711	hsa-miR-586	13.72829078	7.731448299	47.17784266	37.78002999	13.9059091	15.50611851	0.058272009	1.538710752	11.700239	5.219532751	0.018347056	0.016403764
710	hsa-miR-585	7.212881794	0.133914198	41.67219393	30.75366856	13.6533879	11.35971347	0.049267794	0.396461481	17.00111682	6.895661766	1.654093296	0.393107981
709	hsa-miR-584	26.04926381	13.57693121	49.1446326	44.74890555	57.59478261	50.16170658	41.91906075	32.07560774	44.70632164	29.55683295	40.05759594	26.1544732
708	hsa-miR-583	9.251756557	0.012044364	46.41594756	45.57573824	25.55391044	13.52462731	2.359020363	0.042733351	8.148596201	10.24884114	4.585406139	1.753132125
707	hsa-miR-582-5p	8.938391994	0.043831858	57.81672418	45.04169902	24.73560309	12.54674272	2.425883979	1.717949652	22.92496538	1.690343202	0.036479256	4.162920858
706	hsa-miR-582-3p	9.697967383	2.481295571	62.78908219	34.92196611	20.67378153	19.70045235	5.083512914	12.13547325	16.08668443	0.017641863	0.019255819	4.282821787
705	hsa-miR-581	0.011861042	2.45874115	32.70659774	45.4324745	10.73746871	12.8203023	2.280451644	0.048486642	8.355962546	0.018579166	5.129677442	0.015705324
704	hsa-miR-580	0.01191637	0.013092963	54.58848834	48.69981683	9.088172711	4.291576425	0.059682173	0.045987824	8.356368881	1.676870903	0.165347445	3.822747889
703	hsa-miR-579	2.307952604	0.013036983	38.35706309	43.41128771	24.60348262	19.38492667	6.98652479	0.851815008	17.30363762	0.016933851	0.019136937	12.26184612
702	hsa-miR-578	6.67015854	3.91105201	48.09774574	37.21057614	18.62540188	10.68610968	2.031585008	10.5583806	14.69062007	7.056134558	9.592902669	0.017168708
701	hsa-miR-577	1.108476525	0.033290469	50.49352828	40.07900641	18.04084673	0.045170389	0.060893681	12.77646662	10.83298941	0.01574517	0.019033033	1.291625089
700	hsa-miR-576-5p	0.011614668	0.740529425	51.55061614	34.43784051	27.4689373	21.64115806	5.943888605	9.921140268	13.16993642	2.851138279	0.036973359	11.86833088

699	hsa-miR-576-3p	3.892675149	1.987334758	49.0768447	36.46903042	13.96712543	2.618141049	17.68235971	3.920306254	9.061076835	11.23133707	1.753836848	24.07461338
698	hsa-miR-575	758.718025	701.190857	925.105591	878.7086735	73.588131	58.89690499	55.01291985	55.65764974	71.60954303	40.31107553	53.19781017	66.09047219
697	hsa-miR-574-5p	259.7240863	218.0460213	268.6478636	266.631567	178.6567278	123.1694222	153.8971754	142.6806442	175.8582991	102.9298004	136.029049	138.3873495
696	hsa-miR-574-3p	165.9530703	150.6714954	176.6346548	194.542744	189.0239588	177.6312355	175.2219675	146.7562216	159.3291394	109.4167325	132.1723883	128.7072166
695	hsa-miR-573	0.015675837	0.81261346	53.10305621	50.07478508	38.47270704	12.29722859	0.123625904	0.049902969	31.82477138	2.483210339	0.034669814	2.161080836
694	hsa-miR-572	62.53261749	49.67262105	90.30060475	83.23220507	48.18971837	35.52944281	35.06485876	38.50314194	45.19227081	26.92578645	24.42438685	33.49997261
693	hsa-miR-571	12.79759669	1.34470395	39.25493763	40.77684867	58.43254712	21.11701601	3.601583206	5.870399806	57.36769197	14.79652644	22.26086144	38.05884792
692	hsa-miR-570	2.072385979	0.013574896	36.43849897	38.06748741	47.57210194	14.01277309	5.981752978	4.445397522	16.06345153	0.018247128	6.999831444	21.08782578
691	hsa-miR-569	0.015550301	1.442428122	39.61013822	39.88640065	81.62188365	16.50806397	12.36824089	10.45213932	14.19174295	0.01699828	5.186189712	9.81653291
690	hsa-miR-568	5.45230983	13.63569248	32.87519263	41.96575779	129.141917	70.01727566	61.38620738	46.68641996	74.35660562	27.07618311	30.08550726	50.93953007
689	hsa-miR-567	1.55926711	3.219803498	24.54401202	39.91848931	24.43362347	12.28897978	53.51344081	15.35508375	4.129349348	0.01916221	3.623450299	5.648321303
688	hsa-miR-566	0.011639247	13.51928461	23.4416684	41.2343579	23.56041927	7.237715746	19.27009992	24.06415945	8.773983612	2.557315136	22.18722458	31.29416796
687	hsa-miR-564	6.862911569	22.46844937	39.50507324	62.36619574	34.73186481	18.69692639	35.83232823	31.37798072	41.85444878	30.94305102	29.70883527	35.00633756
686	hsa-miR-563	8.557900626	15.54377259	42.77435521	50.2778623	32.77894373	23.36804182	35.58078781	24.34164608	35.2913641	26.1528295	37.51376	24.56267571
685	hsa-miR-562	0.012094737	11.26149952	28.49345636	33.83817792	21.91964057	11.54163688	7.960361841	28.43555883	16.29236683	7.772404346	17.17903126	21.75596742
684	hsa-miR-561	0.01239949	2.758210695	18.14651684	36.79513749	23.27697111	25.60183704	41.1786125	12.73712133	7.808820583	0.021796017	23.43476327	12.44450368
683	hsa-miR-559	0.013173991	8.512855975	21.9395921	42.44906093	21.00739048	21.56700057	10.70770354	8.276617932	2.415079948	0.030524064	13.4675049	13.76070639
682	hsa-miR-558	7.272597258	16.69692574	10.48379718	34.75772893	20.85802746	14.02707442	13.60274801	13.00339374	22.91292997	0.021492773	22.74102194	12.64296093
681	hsa-miR-557	7.790219447	33.70497476	43.95000771	25.55615484	18.08478181	11.10916959	21.04953136	0.039341171	7.183074055	8.412132463	21.85424331	189.3802818
680	hsa-miR-556-5p	0.018592669	27.00933605	42.09043662	15.20094429	0.092229752	0.049676922	16.04691198	0.039974741	3.270459857	7.099225836	20.63701395	31.78597792
679	hsa-miR-556-3p	0.013159612	15.31905346	43.95120027	16.99582435	7.536661607	23.94769888	34.35957896	6.008473601	10.69694903	1.407211393	5.068168492	12.70060846
678	hsa-miR-555	0.428981781	13.28049963	38.42448786	15.77251691	11.64810804	21.41353178	24.60187963	2.401185304	15.11768069	13.95996064	12.11693706	0.641877171
677	hsa-miR-554	11.82832679	26.07072507	33.70237198	26.7917936	30.54177538	22.37388346	21.43705625	1.517719254	11.12351528	11.98779892	9.684790219	0.016225596
676	hsa-miR-553	242.8799172	339.7158096	387.3705207	337.0690253	7.919715922	22.37813125	26.01108926	0.052329508	14.70586549	7.741832909	1.202962485	0.01646568
674	hsa-miR-551b*	2.763216333	22.92318833	36.75122028	26.07794529	19.28945871	24.33744895	33.46888166	3.884278812	40.46567736	32.31041249	27.14213527	4.370454024
672	hsa-miR-551a	0.011997811	24.18283737	36.76444599	18.72519786	33.48170422	46.16069513	50.72761583	41.40202099	40.88397421	43.79901237	28.8671417	21.35793594
671	hsa-miR-550*	15.19262028	19.16679844	37.21008642	26.15863175	38.5957381	33.7855096	53.07342409	20.09028693	8.969827046	12.62689246	13.29699128	15.29861986
670	hsa-miR-550	27.41829561	33.27934528	47.52508597	45.55971548	24.84229223	39.96926507	35.73375221	14.92410291	41.93893764	48.08770521	33.6494409	15.368532
669	hsa-miR-549	2.651149905	18.97401739	47.81929378	18.18252305	3.339505744	2.802793687	41.91494042	2.210981862	20.43203847	17.1320949	0.038162102	1.28474378
668	hsa-miR-548p	0.013734808	16.32286978	34.16756316	24.20555798	17.64094747	22.71197407	25.33623167	7.059125676	17.4510286	20.16344158	9.021147362	0.016403326
667	hsa-miR-548o	0.012085665	15.85779455	38.97645066	24.2725462	15.75147369	22.47024284	63.11063502	12.22650058	15.4006036	0.120547165	34.83867659	4.391328975
666	hsa-miR-548n	264.5751656	363.2511552	527.3376528	328.3759498	20.97238125	17.03988726	49.06078234	13.18792619	7.914444821	0.020823934	31.65906751	14.99793471
665	hsa-miR-548m	2311.037253	2880.152961	3381.422803	2977.760034	20.65030675	29.09335164	22.19533238	36.89641187	40.15874476	28.88959972	40.78352541	34.93030697
664	hsa-miR-548l	121.5779574	192.1465384	237.2308981	199.2060116	16.71196413	21.70546503	27.86433322	15.71997144	24.55522241	9.960215999	12.17297135	19.41912224
662	hsa-miR-548j	1.282911807	26.40527986	36.14059312	32.56014137	6.658848228	23.78912133	22.45675435	15.56644809	15.49068393	14.89328204	14.64727509	17.36152654
661	hsa-miR-548i	0.945433794	18.62501017	35.53348172	21.2623459	8.404808229	14.7745147	30.52636516	11.46564106	26.22419397	14.32856177	23.32980536	20.12295393
660	hsa-miR-548h	4146.92718	5486.010463	3272.305915	2505.146833	51.53868877	29.87656062	31.54399958	24.41348888	104.4414376	29.94734672	46.65570924	28.50741706
658	hsa-miR-548f	984.4001177	864.2356721	1066.621029	966.0572153	25.79512394	35.07123814	37.38570736	23.03606306	50.70145882	14.39264053	41.1567015	41.08282745
657	hsa-miR-548e	0.015850907	21.38693536	29.92899436	14.99429627	34.10087284	31.61915853	25.17585177	14.82150296	3.45891112	17.98703586	27.14756664	22.60350099
654	hsa-miR-548c-5p	57.65139441	90.9799954	94.60654902	119.9127529	15.60001362	19.47252982	40.4916169	12.75072091	15.31462365	23.21697048	19.74793659	7.945420038
651	hsa-miR-548b-3p	24.29620369	31.8246819	18.83974539	46.76202269	23.48143947	30.26956652	10.59812026	0.043595921	21.34288693	13.59300513	10.13315363	0.015159523
650	hsa-miR-548a-5p	17.7909395	30.51243563	25.44525294	41.3022225	16.48746478	26.27466035	0.046105282	0.04645932	19.55563752	15.75964465	9.413462753	0.015333084
649	hsa-miR-548a-3p	10.41816727	21.2366422	18.14114824	38.72970926	14.77973717	18.57926306	9.412139281	0.047056644	16.76240582	10.63437294	0.018230297	0.015958401
648	hsa-miR-545*	1.283176913	33.68194788	17.20500192	42.54873644	11.24642961	1.735377051	6.885481403	0.049376771	17.18889296	14.54656973	2.159607182	9.123758698
647	hsa-miR-545	11.77879721	26.34941845	19.58206818	41.11300458	19.01307519	20.32233325	10.67119321	0.042180404	13.10877273	13.12467669	0.036319373	0.016988205
646	hsa-miR-544	13.79917261	23.11698072	32.38670208	38.23889054	23.59126077	28.45743672	2.955441077	9.551290387	28.80836107	28.74991094	20.33407983	4.267815422
645	hsa-miR-543	12.70280035	19.85637772	19.27775184	39.04436339	25.79174067	27.1571018	19.40301934	1.986553951	60.11438249	51.19064276	45.82210485	31.12020561

644	hsa-miR-542-5p	28.43572028	40.17399143	35.26621086	52.48932277	56.73139534	60.51770585	40.26721929	56.00572259	20.37523181	15.64062469	8.300378505	0.015119005
643	hsa-miR-542-3p	2.546763028	23.09209173	22.25386511	45.05914557	30.56465847	14.78872363	0.074980932	0.046354057	43.36969759	44.25700973	35.29458643	24.32623004
642	hsa-miR-541*	12.56893808	20.63959876	20.01402972	36.74346368	39.38257681	45.40058639	34.75617411	26.37607804	34.22887108	12.75011399	16.79082507	9.974943316
641	hsa-miR-541	0.011357657	20.02380572	17.53866443	38.19315897	18.97038228	28.37665882	26.52481271	1.827193555	19.4168189	16.30190805	0.024923384	4.928037438
640	hsa-miR-539	9.790530178	28.16372209	28.46096333	42.18758695	13.16505118	20.65231239	13.35799471	0.045498173	76.6248825	53.55430447	32.99157809	33.26158883
639	hsa-miR-532-5p	30.05757697	32.21875138	40.81958326	58.14916301	89.03529486	91.65888183	93.06868607	65.63280746	43.8514524	41.01028826	36.42732975	28.15674946
638	hsa-miR-532-3p	13.36032853	20.30828965	16.90484716	35.30325856	53.90141849	63.2652981	37.86351247	38.88499068	35.55787011	3.845225465	0.019097803	9.483116732
637	hsa-miR-526b*	4.834646381	20.72474581	21.55303964	41.15618037	37.91140827	21.14793514	9.096463607	0.331833927	23.42499422	2.959706228	0.710379878	0.015963503
636	hsa-miR-526b	11.37069469	20.67757675	13.04054613	43.05374085	31.02256218	22.5579987	10.35906398	0.049401358	34.50602319	5.615442776	1.153017732	3.498723583
635	hsa-miR-525-5p	3.129485794	22.28528382	7.222911698	38.29182622	21.83009477	19.09189487	2.935094627	7.452696602	22.96472753	9.090191064	0.018999585	10.25411114
634	hsa-miR-525-3p	8.56688106	16.46174311	12.97024189	33.54785665	31.77335718	32.6287116	33.76776933	0.642845774	22.68038404	7.967356311	0.01897427	0.90159577
633	hsa-miR-524-5p	11.86523447	15.75618402	20.19801626	45.59805854	39.88102935	32.47523061	0.060105944	0.045810627	19.09656612	16.88705877	1.613273157	11.44790747
632	hsa-miR-524-3p	0.011488842	18.36833924	20.48076174	40.95608472	21.70613889	40.04791537	18.08448229	17.65274069	23.49032327	18.6053945	13.53244529	28.97976682
631	hsa-miR-523	8.3664641	15.25918713	15.63305787	38.12547533	23.94905485	21.18390306	13.15128616	7.173322629	29.6793353	8.916776385	8.643809576	8.943353326
630	hsa-miR-522	5.198481083	21.56318976	15.86968837	38.82980556	32.3767689	43.52416687	30.39275811	0.042988698	19.86476341	9.658253574	15.44409838	16.01973382
629	hsa-miR-521	0.011656367	16.90789268	13.20495939	40.58921574	26.60388882	45.89163802	45.4932848	1.926862994	12.61860179	11.85031095	0.01906303	13.69602163
628	hsa-miR-520h	5.023831739	15.50389026	9.672010878	33.16430155	33.61057439	50.48759796	24.21308617	0.855773059	27.14522277	8.237614285	5.639636468	16.98550808
627	hsa-miR-520g	8.012993054	17.82364273	15.39197153	43.12845469	64.71932227	43.64520805	25.83623536	6.994323917	74.81483071	25.10699537	0.322643143	11.14919563
626	hsa-miR-520f	243.3080168	239.5043759	216.5025756	279.0294471	96.5259464	40.82249373	25.89577818	9.097365009	52.25586408	31.26293106	4.82850924	10.25784167
625	hsa-miR-520e	3.803597753	16.56285896	21.42527952	41.37487562	92.97331813	37.53554524	8.399051793	1.202290022	27.60212734	21.15369133	5.512230611	15.42769117
624	hsa-miR-520d-5p	6.01261735	15.12207966	7.103773753	67.72986591	42.49621824	31.09344486	24.02750902	6.6350196	43.71844315	31.00589157	12.34531954	31.43136279
623	hsa-miR-520d-3p	4.254344164	12.21934362	13.19458254	39.88119804	89.18456347	38.81206573	23.30038636	0.045357321	48.30019213	18.92984786	16.54455455	14.57145448
622	hsa-miR-520c-3p	0.015364226	12.65451997	1.599338313	63.67055784	72.48699305	30.30923859	20.97771402	7.915237311	31.56366013	15.1108262	6.503791854	0.016475813
621	hsa-miR-520b	9.940985876	12.26630722	30.37162141	38.56152106	50.91005834	16.96719139	7.42353147	6.597082499	18.41119481	2.316636273	0.018507075	0.015066371
620	hsa-miR-520a-5p	10.03746571	3.369711828	36.31002008	42.79723148	24.02112264	5.931497459	12.5816262	0.046139393	22.65040332	14.47791538	6.686647317	4.094721783
619	hsa-miR-520a-3p	11.8993122	11.49496249	31.52503453	47.19048944	28.92312224	20.00363618	14.06672321	0.042494446	21.33709985	15.85152523	7.480566429	0.017298192
618	hsa-miR-519e*	7.104873271	10.68301452	39.56407913	48.60490442	16.31282335	35.84332804	5.376903558	14.21233711	20.18638718	15.14362098	13.68177836	13.1523276
617	hsa-miR-519e	0.011461276	1.81075522	23.83063557	33.99684443	42.5177211	41.6545581	31.34135505	28.6852584	37.13993847	18.93917628	24.24491818	20.87007301
616	hsa-miR-519d	12.99455922	2.890480202	34.99011346	35.37640899	52.73556523	46.03764496	33.57033264	30.20133067	12.46264671	5.113664237	10.54709071	0.330844161
615	hsa-miR-519c-3p	2.91610547	4.36206391	40.6043821	38.2929988	37.84735514	1.912331736	0.053048079	6.836455998	19.57993456	8.855784495	15.96885151	4.006253814
614	hsa-miR-519b-3p	104.4798981	106.2515648	137.1262939	133.7979093	32.06159576	19.2291132	1.585329885	12.31052447	24.04252837	2.654720418	7.899965694	0.015372155
613	hsa-miR-519a	4.549693088	5.258230268	32.89077307	47.98079197	28.49951978	16.52665745	14.06272266	0.047301663	12.76343678	0.017807161	6.498843517	4.186262701
612	hsa-miR-518f*	4.416448793	8.240977095	26.6414069	46.97393593	23.69773657	7.993304703	11.21079569	5.238818538	21.8321244	13.21248773	1.917938007	0.015578529
611	hsa-miR-518f	9.552993639	2.722323956	32.9634211	47.77503194	26.54185779	11.14077432	0.054061265	0.048049786	31.90804519	9.394051094	0.018281982	6.69690121
610	hsa-miR-518e*	5.246250652	2.061299938	34.95033516	42.0493047	34.27710463	13.27435706	4.675344389	5.494157478	28.93311555	2.02749552	12.79042663	5.447093609
609	hsa-miR-518e	4.076644554	3.434981457	23.8235371	35.94027481	36.94741868	21.04694029	15.57580459	15.59357505	28.67822168	7.647550019	0.019008588	3.16106862
608	hsa-miR-518d-5p	15.80431469	6.287752025	31.19110974	46.22977847	37.62085332	15.42254597	27.25800803	12.51185619	26.82380724	0.660071246	1.683393126	15.13963438
607	hsa-miR-518d-3p	9.141268925	1.204010445	26.69033842	41.6509843	35.22419692	19.70379297	12.32873535	17.45672318	32.71180415	3.507740695	0.019017157	16.68569957
606	hsa-miR-518c*	12.60304448	8.657227147	33.95413091	55.13577335	32.95950585	30.22618552	21.23490333	9.021667526	25.01311081	0.017802768	3.678286722	7.587833341
605	hsa-miR-518c	5.539045101	0.013632929	23.32828922	39.66786898	36.06214813	8.772533902	19.22889006	19.48928023	25.05830699	13.67949028	1.311018262	35.15180838
604	hsa-miR-518b	11.19026792	0.527433624	30.08798393	48.2154647	42.23306757	24.26045326	42.09652324	27.8540361	32.70698779	19.62504127	0.018682596	26.19489329
603	hsa-miR-518a-5p	7.990586063	9.155574133	32.40815237	44.49239541	49.56394904	21.06028782	24.46789062	15.85163799	21.75931147	13.00409479	3.96582782	22.93979747
602	hsa-miR-518a-3p	7.521818671	0.011617989	23.08300877	37.20814999	43.79582018	16.58949265	12.56517741	18.26761571	12.95259177	11.87860803	0.038135203	23.41545849
601	hsa-miR-517c	1.429045752	3.369083484	37.19938229	31.68320325	31.32946351	6.036796985	49.0536207	13.57898608	18.4988398	15.41165242	11.32474972	22.10643604
600	hsa-miR-517b	71.47281261	49.73601495	93.57055146	102.5428725	38.33632135	15.44880907	12.12489562	12.71402545	17.52328874	3.12189834	8.503331355	19.25081655
599	hsa-miR-517a	0.011742145	0.012077616	22.03864297	38.96510953	31.28059226	23.93815726	21.19289025	18.8122547	34.11097006	1.564868414	8.573232639	19.60685688
598	hsa-miR-517*	0.012689831	6.156522983	24.61868226	38.68387938	28.25223302	13.77780696	11.11562377	10.85059407	19.3529661	0.275256968	0.01875255	17.47143375

597	hsa-miR-516b	10.97621875	6.177672479	36.06591883	37.03627284	33.81777936	13.81942348	0.059630635	9.083250504	34.44312356	0.020131607	0.020506134	12.33761827
596	hsa-miR-516a-5p	14.60811342	5.769850838	32.07915438	43.52313554	102.6708593	21.4013074	13.46384006	9.517903335	65.43827393	28.04151955	9.873275467	39.7325212
595	hsa-miR-516a-3p	8.458761775	4.144751515	29.21752623	35.92389734	94.42444363	28.58057779	32.38730593	20.91543961	0.034352501	0.020972876	8.121450742	16.7001855
594	hsa-miR-515-5p	0.016989009	0.014221269	29.08082499	44.92432873	42.56184386	19.64133406	25.03224327	15.32507318	49.54861008	1.989852506	0.194803394	13.50142313
593	hsa-miR-515-3p	103.2173234	60.91975259	126.5021595	164.1860942	89.93232561	18.57765309	9.262375175	0.050500394	9.156320956	3.599021867	15.81362322	16.92159002
592	hsa-miR-514	0.015909017	4.35766287	33.4448677	34.51267154	39.03133985	17.44024636	3.798020102	12.35470306	18.89287144	0.020743176	20.37314864	15.42415884
591	hsa-miR-513c	3.572144079	7.237764335	28.56468025	35.2487094	22.72867344	31.04080512	25.68124763	5.570257135	13.73871095	0.01883633	20.47356922	14.01328283
590	hsa-miR-513b	3.766248991	20.97674621	23.95187752	39.76439648	16.95166348	11.72986773	8.87851514	2.115570576	48.73721697	8.068654788	19.7490564	29.38670565
589	hsa-miR-513a-5p	244.7802715	223.4939456	271.7029814	245.4835184	26.36264802	12.56091931	25.40036774	14.93339832	13.82426259	15.08097761	35.64930448	19.82361109
588	hsa-miR-513a-3p	0.012351108	5.205773851	28.27229847	38.86672437	11.20411233	24.18624735	23.47557351	8.901136011	19.13839301	0.017999785	24.9248853	20.4784644
587	hsa-miR-512-5p	2.373203099	12.90192395	18.91311686	34.32729096	22.21802883	15.80455749	12.67133074	11.85796083	40.31752431	7.322427207	29.8269313	22.42838432
586	hsa-miR-512-3p	0.011869559	7.297309525	22.22607174	36.60800773	19.82554266	9.492991749	16.74078003	7.70057278	27.0548988	15.34624174	20.03347771	22.90767247
584	hsa-miR-510	11.25833513	6.605625281	30.33546297	37.5182041	34.87478875	8.788359906	16.02446171	15.64786439	10.89176492	27.81518337	24.64974706	295.4005612
582	hsa-miR-509-3p	2.197667492	30.89333589	35.74404175	17.85124689	17.70901952	0.054075941	6.406001389	1.416224629	0.017262993	22.44989827	19.12465248	164.0447007
581	hsa-miR-509-3-5p	1.465513481	19.40077664	32.01178835	18.57863206	15.1128094	17.70270155	22.1676688	2.933207229	4.18786927	30.97194155	25.73250458	19.33678067
580	hsa-miR-508-5p	6.206503966	21.81155683	39.8656638	21.53386026	26.26457744	24.15065551	23.30008964	8.546158429	0.017892356	40.83201499	18.27070069	0.016239644
578	hsa-miR-507	2.778473234	23.58963159	34.29383712	28.86708476	3.56335403	2.436850084	11.01337974	0.046264986	12.45949273	15.40844339	14.32480219	0.015152744
577	hsa-miR-506	1.640292929	22.81748509	25.68754968	17.58261572	40.58882703	37.19000663	44.22871304	30.51993143	33.75656932	42.53118036	22.94147107	23.80563194
576	hsa-miR-505*	19.47668237	63.97969952	51.22933775	40.43282855	44.0605552	95.75464484	56.03155909	35.77759606	43.98450592	93.78677709	40.0685562	10.65973337
575	hsa-miR-505	0.011618857	21.97014355	39.33333287	22.40007327	25.15390726	29.91027972	16.81457727	12.41282922	26.96333133	30.46230299	27.58936532	8.98609318
573	hsa-miR-503	15.29774968	43.96552671	41.94183696	38.77621854	46.50322609	49.06745112	58.79115222	35.54057872	24.85785715	42.93647634	44.15837984	13.05911459
571	hsa-miR-502-3p	0.011916253	22.42836931	62.29623482	27.65937588	22.68122413	41.47119374	26.23761862	19.76081465	2.269348362	19.73281711	16.28607314	0.016439583
570	hsa-miR-501-5p	0.0115832	23.32217993	34.93383117	23.72491511	21.23396415	28.52826939	25.83073843	16.4123347	41.15231398	36.10740974	36.56964312	9.453340763
568	hsa-miR-500*	0.011585263	28.98402874	30.77584713	28.83671364	29.5757061	34.80588208	31.3525314	29.88363629	30.78984899	12.36040274	34.04287787	19.75101788
564	hsa-miR-498	2649.401338	3531.82736	3783.785359	5253.764081	39.36734204	76.59738927	44.36872337	36.91101173	95.36427659	99.61735581	92.46334252	72.646334
562	hsa-miR-497	31.15680811	45.05651704	69.20811249	54.22137221	49.15061226	40.79376323	12.26496297	21.42902483	37.03518555	14.77235431	22.92596023	20.97267451
560	hsa-miR-495	1.580918378	18.99155257	27.16900181	18.19458758	64.96752394	12.77853562	17.08383643	24.26605346	54.86974492	39.82687894	44.57702846	19.34964805
559	hsa-miR-494	31.47134178	38.50767938	43.10534809	49.5054956	42.39751278	37.86787923	38.15361505	23.82043048	27.37892938	36.31451612	41.94684273	29.59252939
558	hsa-miR-493*	0.015765948	20.23383466	30.11764334	29.01513502	37.05519083	7.476397214	4.210767928	0.045274857	17.74775912	30.58715409	32.71403144	18.74067872
556	hsa-miR-492	0.017524974	16.81998161	26.55092243	19.22995692	11.60996957	33.07909466	14.57696242	12.89278598	18.75640083	25.52427947	23.82278377	0.102887657
555	hsa-miR-491-5p	3.458124951	23.30108513	26.09056336	23.69261332	52.40742219	38.27475184	30.6463122	25.9238537	14.12118263	18.86435738	10.99654879	0.015467315
553	hsa-miR-490-5p	15.45359332	29.26821489	17.03077676	42.65280467	30.40784862	30.94331749	20.50304141	0.044865512	26.72304237	37.19315927	12.25686156	0.014665695
552	hsa-miR-490-3p	13.31557852	25.34964167	17.35305615	42.76155653	23.33497998	18.56814565	16.87137081	0.047591292	11.28851004	34.12429352	8.806882963	0.01563725
551	hsa-miR-489	30.14299014	40.05066243	46.80951384	66.66368692	22.32886638	26.69986757	10.52249614	0.044897887	10.0787419	21.01519842	1.611742935	0.583646905
550	hsa-miR-488*	8.101992487	23.02464149	16.39027326	35.13411715	41.21074808	14.2138317	23.65301757	0.048112089	23.91519209	29.36293511	0.017693698	2.834189422
549	hsa-miR-488	20.5589041	21.15589073	19.11908997	39.62152232	24.73586853	13.29007491	17.14740634	0.044171318	16.17111853	20.30800251	0.759811484	0.016226496
548	hsa-miR-487b	12.4477856	24.31209603	24.07299406	33.96042443	43.49453408	11.05267843	21.53271106	3.941625239	81.34255514	85.64694423	51.78391221	41.27201123
547	hsa-miR-487a	8.578205955	21.09657318	22.00187303	49.31364778	37.4002101	26.51534571	16.19441764	1.42637688	71.58632792	68.41419954	41.42436335	17.15943102
546	hsa-miR-486-5p	19.18803252	26.02390494	25.82573609	42.06895349	81.02959248	50.78291679	47.84820197	47.07299983	31.52653743	30.01171827	0.017650099	5.964302637
545	hsa-miR-486-3p	12.71703962	20.91600068	15.63351042	42.5356409	42.81822228	49.4476201	20.98633944	14.54246237	62.42577391	66.82910729	46.72076121	38.94120268
544	hsa-miR-485-5p	10.41623033	23.27396707	18.66576216	38.45299377	36.64267748	13.34669372	11.31008874	0.042411584	94.22476454	108.1039859	64.58564373	65.38491167
543	hsa-miR-485-3p	16.21190596	20.32184887	31.42990723	42.71062191	95.38228858	76.97825424	77.32414984	61.88504453	189.0184814	173.8695707	119.0625841	143.3059548
542	hsa-miR-484	36.9588673	38.46190387	32.18992987	57.84186973	149.4127884	130.7184704	156.8131773	116.711891	37.6231846	27.58728292	11.09871716	25.18587131
541	hsa-miR-483-5p	1916.103053	1803.783077	1924.210772	2381.500105	172.6576959	150.7933235	133.9124308	138.7952967	34.11469488	24.17125869	5.978306531	1.732579852
540	hsa-miR-483-3p	8.035496444	15.59529195	16.64341081	37.95436065	50.15003096	31.07909373	35.38254349	17.89345909	209.4258321	195.4994623	172.1292421	181.2885649
539	hsa-miR-455-5p	8.394425541	17.66134169	24.8009195	38.5756511	28.91957177	28.48655995	14.57478634	2.386568362	22.87912079	19.05232648	7.468925077	11.12234976
538	hsa-miR-455-3p	94.64184661	89.50450419	107.0362876	117.2499729	251.0704097	229.7856131	240.7809791	206.6203296	319.0591825	290.2789803	303.1457386	314.1339198

537	hsa-miR-454*	11.65634628	12.80019228	15.47981451	45.46244565	31.34628907	28.28789873	34.01285464	0.043561999	24.25318667	27.3844437	5.901853756	6.39779141
536	hsa-miR-454	101.171341	75.51169464	82.91610797	128.3359949	364.5268172	312.7159432	362.0519756	330.8322862	14.93462356	19.06358217	0.019068314	3.028369621
535	hsa-miR-453	3.068558668	15.61464501	11.18772438	42.70022493	30.42939884	26.89249241	19.18932208	4.590182433	152.3891944	134.2839792	125.3884777	125.8933279
534	hsa-miR-452*	0.011784923	8.081118819	17.2095753	41.3037824	28.12855893	11.82510797	8.143833835	2.071456179	108.9633543	80.51668473	80.55158768	82.92222846
533	hsa-miR-452	70.08985435	61.37429245	73.53197155	91.83785206	185.9177507	132.3990533	209.5633502	114.7038155	27.32457149	16.80355577	2.218596217	2.46711595
532	hsa-miR-451	3.732347963	16.03334416	19.4394095	33.058326	94.60379019	63.95831129	46.68328368	42.07705876	22.31150093	24.23731466	14.73931902	7.093577504
531	hsa-miR-450b-5p	15.42670447	12.31394558	15.84287049	34.54402056	35.01078431	25.62354615	12.35928698	3.775073322	43.46538863	17.1924466	5.119413187	0.018422362
530	hsa-miR-450b-3p	0.012798038	10.77560365	9.836354242	41.77542998	24.5591932	20.91986054	16.44151948	0.048506609	57.81449393	48.25904758	29.72444861	37.04176318
529	hsa-miR-450a	0.015465699	12.70510255	23.10046093	47.52752707	57.95781814	22.53153559	10.68723873	0.058558759	0.020255694	16.11727798	1.334401649	16.99819543
528	hsa-miR-449b*	13.26093889	22.43270944	29.48557714	40.66753157	75.45882123	46.66807005	41.72915701	22.83821474	21.7283402	18.53742027	0.8680279	10.84363261
527	hsa-miR-449b	1.695040569	8.653593673	11.3765612	35.26653742	48.52198001	21.20790296	7.262779001	2.619619533	56.13589784	26.69379853	13.83906707	17.61729845
526	hsa-miR-449a	2.171194813	14.17200875	12.51159906	31.10490917	46.54443763	17.50605275	23.15738833	20.62642477	38.61090021	9.195571625	9.621263804	0.015271756
525	hsa-miR-448	8.595759974	8.180619166	16.91738551	41.73637102	58.84693011	16.83823018	17.43917026	5.728728089	35.63522308	19.99031439	13.43550861	2.039549793
524	hsa-miR-433	0.015350904	10.02287843	1.458526317	32.40119833	69.62333029	30.77907298	14.22461975	14.72400551	35.38564958	30.62136215	15.96787697	0.800285357
523	hsa-miR-432*	11.90565893	14.12021251	33.92232573	31.82887609	36.48926265	19.98422829	15.54849429	2.826912877	14.98698472	7.125925846	9.659150662	5.860203554
522	hsa-miR-432	16.01144192	10.76779197	36.51760428	46.27463719	28.13972862	6.073539541	19.17068774	0.04148327	16.43087604	0.022134849	5.780545989	2.804018141
521	hsa-miR-431*	0.011176443	12.47824784	30.61591006	38.81714298	30.83384447	30.55185471	10.48651199	6.307113814	48.14866253	22.57063655	26.03130561	25.82220521
520	hsa-miR-431	17.77618116	2.373381411	34.91485756	37.99439168	40.57583873	36.96419584	12.37473067	10.40252053	120.9501404	133.3245328	122.6850359	91.49453287
519	hsa-miR-429	4.461618773	7.11768893	33.33708424	46.79028041	21.39297313	18.28689874	0.047335333	0.041387625	76.26030192	54.89193289	58.51293395	54.17304176
518	hsa-miR-425*	32.9339469	25.89862161	43.73990839	58.65265976	32.38385685	23.12312887	18.32461315	29.10685757	46.57221093	15.74956598	30.67898798	11.9597053
517	hsa-miR-425	110.9271806	173.6009653	195.7534667	135.8294378	136.8874042	156.2518217	161.4062194	113.4638367	749.2658061	684.1346156	703.0606324	716.3104415
516	hsa-miR-424*	26.30476701	26.49688545	43.44215803	49.76439822	96.90046827	71.44804609	83.84696878	66.97078741	72.83327602	43.75293698	45.48315544	31.07636936
515	hsa-miR-424	21.94929889	21.97335406	52.73806636	52.66051357	38.00631976	35.24152064	37.74172067	15.62949588	55.92102797	13.15565316	15.29051359	16.58944535
514	hsa-miR-423-5p	450.9303333	457.535079	528.2344168	551.3619266	705.8084649	698.3571818	637.3842106	651.1080911	121.9585277	68.70731379	67.69598837	70.64908742
513	hsa-miR-423-3p	15.02174415	19.2791542	36.2345649	45.4779366	40.40704376	41.01327029	24.42134641	25.75943148	42.73856424	16.15250682	5.510812227	16.41650649
512	hsa-miR-422a	16.064238	11.71840271	32.29988812	41.92707471	30.74078363	29.87211943	20.19853883	0.039429053	40.35290402	11.39236297	0.018055891	17.72543846
511	hsa-miR-421	36.57823013	31.93496079	54.44003903	66.54767681	130.3098864	104.8421815	111.8876612	79.83680294	34.99376752	0.020123702	0.018793934	12.56824418
510	hsa-miR-412	5.272942586	5.231203054	34.80722987	39.47023577	42.26764929	23.87992308	12.32185296	15.59121632	27.3232994	9.89475158	1.346194912	11.11022206
509	hsa-miR-411*	0.011400574	10.83762283	36.48828759	44.05745989	23.26328132	34.83874949	3.547179922	11.14304174	41.08515928	1.276665077	12.35253986	19.64223101
508	hsa-miR-411	9.997596316	8.922148717	29.92734646	38.1059393	29.68274565	29.49594413	0.483716903	5.959975258	105.0684093	91.67880679	79.29845266	85.91296769
507	hsa-miR-410	1.962251692	11.62791307	21.47279842	47.00188312	27.16609607	19.34518635	4.770397704	12.84377051	33.74457437	0.019108119	3.432751804	9.85603126
506	hsa-miR-409-5p	1.775797576	8.190540836	32.76945911	40.04159808	28.01096417	26.72643401	12.75865308	22.42716542	45.96075189	3.000304229	11.20030758	24.05586517
505	hsa-miR-409-3p	25.65992284	14.70911731	42.94915783	48.59749684	44.08647339	33.72717331	32.05000838	26.37435227	38.19846519	6.751196124	18.58047606	21.06040458
504	hsa-miR-384	4.891711776	2.564560213	29.4410775	39.92270547	19.00869194	20.79071062	4.569963005	2.557025379	34.80077329	0.016355075	27.83620692	27.00707962
503	hsa-miR-383	0.011586731	0.012104614	27.33642152	40.67432753	15.54540666	17.59322323	9.634199216	13.97136623	46.30166746	0.015989139	36.96462561	29.54976767
502	hsa-miR-382	4.825609038	10.69460435	21.61500609	38.95577699	38.9837607	17.68703853	5.937421626	5.05287341	26.49105685	0.017410593	9.381762912	27.25302109
501	hsa-miR-381	0.094990851	0.01297726	39.89098693	43.8440883	24.92551745	14.16125871	31.12054465	16.43503278	52.74637755	0.018641759	9.733111593	23.40414571
500	hsa-miR-380*	0.48352405	0.012782825	23.66271571	39.75151373	28.33754665	17.1475685	5.964569381	7.882680315	88.99014946	2.140988691	21.5163379	26.23923542
499	hsa-miR-380	0.013036721	1.438521614	28.26666007	41.13919995	39.02153115	17.31024575	5.956927327	15.06047486	48.31006544	8.298059315	33.32196303	36.09000847
498	hsa-miR-379*	4.180712143	8.797224283	35.131843	44.47707271	50.82098319	25.08868363	4.731650773	18.72737522	258.5331627	167.5234441	201.5494741	185.0372072
497	hsa-miR-379	0.162204353	16.48449759	29.90018793	42.65699217	70.22503441	13.70808952	30.94672304	14.57803532	69.82911407	0.017963732	20.94271632	29.14137322
496	hsa-miR-378*	3.607435625	7.853154756	28.36350723	38.96036798	64.74275987	33.96778291	33.63883939	22.34995359	15.79429989	9.504020432	8.545725773	28.35322916
495	hsa-miR-378	88.69226185	85.35737772	121.0237669	96.76355608	342.0740539	254.1222143	268.7414005	189.5915344	37.77382538	18.33632146	15.08221123	26.80239723
494	hsa-miR-377*	0.015370639	1.376612791	23.97001651	27.39617811	58.98468155	19.67884916	3.436524761	8.879470142	22.38370227	6.784143544	17.25430445	23.16328995
493	hsa-miR-377	0.012105266	12.84789819	33.68265361	28.25711693	24.79524641	21.47908019	18.35191862	32.64072595	23.32205075	14.9186996	34.54317867	33.81461373
492	hsa-miR-376c	2.843018282	11.84051896	29.64410617	32.68844486	22.89935459	18.19514605	13.97634948	23.02724444	9.503780835	1.718831746	20.74693022	33.1040691
490	hsa-miR-376a*	0.011707446	6.289369207	33.7135575	34.87063562	20.74121022	22.97179019	11.41722737	26.03145042	113.6371273	53.94685911	162.2490814	98.5539747

489	hsa-miR-376a	0.01173377	11.33921592	41.78342918	31.33485974	21.10551214	11.99434417	0.082606246	34.13695165	48.09308523	23.28266405	46.25769603	1.317106251
487	hsa-miR-374b*	6.594299303	2.963820345	45.57263822	28.11525472	10.68873673	5.024297913	0.10988622	20.50181187	24.95291378	25.18108828	49.0756196	17.50456987
486	hsa-miR-374b	95.71980476	88.6264128	122.6918769	111.6977841	119.9008916	94.72107946	139.4157972	93.85586377	21.10726997	34.5157659	27.51870218	15.21592692
484	hsa-miR-374a	0.015508647	35.15746958	30.53133324	32.57846802	28.34748689	8.771231898	32.33861416	0.044888573	12.31818853	14.72123132	18.68041184	14.54220892
483	hsa-miR-373*	23.79012001	45.18593905	51.41170902	44.2256655	25.90690385	22.45751262	29.48235339	16.14145511	114.3901394	119.5705136	118.124335	66.83933317
482	hsa-miR-373	2.153544186	28.63271806	32.19721701	27.78032834	18.16024128	17.81409403	35.31695349	10.82436471	34.73655741	28.06989926	42.45778418	11.1781321
481	hsa-miR-372	0.011963137	25.9107858	37.26982321	28.5023617	9.285589007	2.730836788	21.22306974	2.408086579	57.06314915	48.1168696	42.94801691	11.2467414
480	hsa-miR-371-5p	43.05157389	63.04022483	53.97947146	61.16477811	71.75531122	54.13127322	77.20228414	38.34623685	28.26054218	29.1700306	31.10857878	5.009616109
479	hsa-miR-371-3p	0.011532427	20.65859202	32.26054031	34.08633375	17.06050006	21.18292742	59.19061518	13.24301827	30.00028485	46.52579283	30.18319069	0.01556845
478	hsa-miR-370	6.596066168	27.72417997	34.50264891	31.77077544	22.30138254	34.75570665	39.44233667	19.31481245	27.68823389	20.37116827	26.09252759	0.016467077
477	hsa-miR-369-5p	0.01170889	29.6524257	32.02485711	35.98753621	11.33962126	15.10808014	23.47097891	14.07114984	30.98668635	22.64673865	22.88415728	0.016644568
476	hsa-miR-369-3p	0.011892945	26.58965642	28.18142183	45.93846942	12.33884262	24.2435141	22.51574938	0.047993858	43.28708041	34.06020638	46.40234523	2.260091659
475	hsa-miR-367*	17.21057249	21.83887914	30.52692752	37.32700064	12.52280313	23.29607014	41.12195118	9.677666639	86.44547067	94.11053951	89.2112977	44.45161713
474	hsa-miR-367	0.78620447	29.70425445	32.66681991	34.56417106	12.98346827	4.678674764	31.38731168	2.935995941	24.8857723	12.86868561	22.86010178	6.432173935
473	hsa-miR-365*	11.1089741	33.13828716	44.20765497	42.31714105	0.041531782	19.16517729	32.24436562	2.521609288	48.14039448	50.99120578	54.9909608	30.78905074
472	hsa-miR-365	59.17735611	44.94396947	55.72690074	58.13052245	53.52641486	70.17751526	102.5379151	48.41270407	52.22425832	32.07009795	37.088677	25.34302417
470	hsa-miR-363	4.129568975	14.2417723	25.96672898	37.97203729	12.75478191	19.80953703	22.99724417	0.04366651	785.7215212	864.141863	766.0838993	702.9663696
469	hsa-miR-362-5p	6.220354067	21.51034636	41.01809756	41.57236084	35.8747694	43.81272982	73.66556227	34.80086409	50.1626151	35.87752711	47.38504049	21.02076374
468	hsa-miR-362-3p	10.45457126	23.00846976	32.02424362	31.21878155	33.38494241	44.51810083	51.97673552	26.93551552	39.32636716	12.5204159	31.22983097	17.08988279
467	hsa-miR-361-5p	392.3417738	490.8935901	442.0860351	461.4708403	885.1479995	964.2616362	874.8472422	829.3189454	67.07169683	44.38465693	40.70750315	42.41530723
466	hsa-miR-361-3p	122.0076227	204.3388824	183.4493445	177.1727838	29.64595361	20.29640828	46.50572045	3.758094073	63.40017707	18.51731729	16.24218232	22.45917471
465	hsa-miR-34c-5p	0.012035613	16.35034035	27.24361278	29.1427612	13.68750378	29.40192149	34.18924961	10.54656291	70.70874015	17.33320847	33.96276563	29.94698073
464	hsa-miR-34c-3p	17.65863963	27.7631216	30.52094842	38.1187342	39.58625625	40.90749878	45.12974209	26.2705072	75.14499086	31.50724116	50.44477466	35.68801975
463	hsa-miR-34b*	0.01580586	16.24297058	23.6655861	27.07874019	19.08812958	29.3611644	43.02219956	6.525915246	181.2959091	71.78921965	102.4941942	81.95238466
462	hsa-miR-34b	6.527758472	23.90378844	32.42424071	33.53805429	47.13531377	29.6413418	43.8182702	29.51586201	89.81087516	34.51349482	47.82893978	35.71821823
461	hsa-miR-34a*	7.410648453	27.84416067	33.73018767	39.09486917	20.4726123	22.0697748	30.12803268	20.36868562	58.18242181	47.06903994	51.79852747	40.28644567
460	hsa-miR-34a	28.87408458	49.1773268	69.81631571	76.83766084	230.0797519	103.5699474	169.4021877	105.2834438	84.55299181	31.28230203	36.93681754	36.31825256
459	hsa-miR-346	0.015338125	26.00331073	32.04856401	40.36533968	56.7082319	47.11258403	43.5258521	23.84719993	166.5432364	103.3239302	123.1832315	132.5630916
458	hsa-miR-345	5.970735901	23.00273629	40.71418127	41.93674148	50.10287363	61.42377527	58.66426503	63.03416111	21.12682683	35.8441562	18.17070762	0.01508504
457	hsa-miR-342-5p	0.015325659	31.09675384	35.4521453	34.78088155	60.52355885	36.10098162	37.2573569	29.52690293	17.99424206	25.29839482	12.68247629	3.803760453
456	hsa-miR-342-3p	82.07572618	59.10522809	72.78041504	83.19932795	204.695925	137.4599422	141.3947149	144.5247313	54.27689941	66.13180891	57.80575211	29.37600653
455	hsa-miR-340*	9.572937954	24.06543076	35.24568092	44.22021871	26.99440312	37.90346291	15.41826627	0.046222321	21.09388755	36.24227187	19.91235876	1.594747536
454	hsa-miR-340	13.82193879	20.84828594	24.6118196	41.92954183	23.37827313	33.93970085	12.13730467	0.04233399	23.5187588	5.404007537	16.35897442	3.756613072
453	hsa-miR-33b*	40.13736789	43.36402516	47.31711738	53.3115178	57.18889079	54.28003348	36.64092517	17.743877	37.6330429	21.6311563	12.47803531	7.827410794
452	hsa-miR-33b	10.94338334	21.20127779	25.23491403	45.6207181	8.408198311	31.26926074	20.41926046	2.390318362	37.46276017	52.40334497	31.18744326	8.436390067
451	hsa-miR-33a*	24.3232328	23.01287145	30.79189158	41.30126768	6.596722996	30.53519723	2.395769492	2.44217767	54.38852303	57.8848076	32.04141405	15.28572119
450	hsa-miR-33a	14.41358835	16.30955544	33.75878537	40.09931373	13.43701364	33.37864747	20.4476122	0.041541822	30.40808773	42.93574961	11.26297958	0.016278046
449	hsa-miR-339-5p	16.97209767	22.00797088	28.47888742	43.01922264	24.87858208	40.85226685	27.34125854	6.480490532	15.20720447	38.45322229	6.107024584	0.016099557
448	hsa-miR-339-3p	14.01334817	23.5496874	34.64155873	46.45552994	41.73322614	56.29093137	37.6541152	17.23590873	35.01978237	36.22982768	8.685782188	0.017021095
447	hsa-miR-338-5p	10.86749389	19.22439213	34.88288979	50.02757694	21.85104451	34.16291442	13.47422868	1.342620234	29.42792469	31.40951772	1.818365513	1.977691761
446	hsa-miR-338-3p	14.46175366	21.86845146	22.52416542	57.21419192	11.15416237	27.78316653	0.051006693	0.050043889	34.4522647	45.16469234	17.65046449	14.00552509
445	hsa-miR-337-5p	20.29347029	22.9925298	36.08219802	56.26826712	32.94066864	33.88549507	20.74568754	6.778404634	88.2543582	69.0859061	29.14678284	38.58733932
444	hsa-miR-337-3p	7.423199515	19.2637246	21.1572242	45.65391988	36.03860887	25.00027206	26.26490132	0.043985929	39.83858179	43.32985492	18.02288905	1.271455347
443	hsa-miR-335*	14.26310581	22.35381131	36.58501436	51.13899872	55.02331952	43.88083686	33.48890016	4.367631076	52.00569952	53.19213697	26.77952502	27.51333476
442	hsa-miR-335	46.61758723	36.52048436	56.37779518	80.41627154	88.14919333	81.03775465	56.12091315	53.03783597	43.85386076	31.70372654	17.11435696	21.41020771
441	hsa-miR-331-5p	1.503182472	20.90978117	30.51957823	56.56530444	43.12873619	45.8015658	16.96247888	0.043286221	42.46574132	35.25077759	34.23338353	18.00636491
440	hsa-miR-331-3p	10.54295031	33.0354425	41.65269897	62.22150969	76.9864939	87.02774699	50.67371908	30.56285643	38.7082383	32.02024537	20.39317529	26.05673072

439	hsa-miR-330-5p	0.011240299	11.73488954	28.45563543	41.28548579	37.4549289	36.60473687	26.07039506	14.96994797	38.62181662	34.82875688	21.74391017	21.81237095
438	hsa-miR-330-3p	10.09848572	19.22690083	36.45972122	47.92944847	45.16806141	60.29575372	63.31979161	15.96140107	49.10990789	31.61957979	28.39696196	30.37272375
437	hsa-miR-329	386.0167872	401.6200152	505.0535552	482.6464843	36.04993509	56.88937692	34.04394849	12.2724284	28.18127997	29.86123717	19.18558101	15.03468107
436	hsa-miR-328	0.01135845	23.78703946	30.91054304	53.60023387	42.44301768	46.89396733	42.02370482	10.29807335	160.4761494	111.7281421	118.4049841	117.9531216
435	hsa-miR-326	4.777814679	17.54393564	36.91172845	47.14216627	48.23818596	54.14819753	68.7766643	12.98580994	75.65499802	55.79382168	30.90265319	41.16183895
434	hsa-miR-325	0.011948448	6.937658954	18.60440441	41.04557305	35.48409017	41.52516358	38.36459234	4.511526459	78.96743101	39.36846245	25.33557268	40.75000786
433	hsa-miR-324-5p	26.18396851	41.48415503	50.03965738	69.33640169	190.0600927	176.4536563	209.7279936	145.0736628	79.03480875	31.77720791	19.4814164	22.04864741
432	hsa-miR-324-3p	10.66730819	28.15970814	39.92534842	52.21083001	65.77321191	42.55593766	34.81452277	19.6609525	1729.868444	1683.19578	1563.208747	1592.705783
431	hsa-miR-323-5p	11.279276	23.17393992	26.43282317	41.0446108	59.31341283	47.97486431	28.61928359	13.16920319	3610.602483	3404.789571	3208.274726	3168.646681
430	hsa-miR-323-3p	0.019189689	15.46726862	22.24828714	47.23613125	111.0292471	45.05999247	19.25844257	7.381090074	2942.640387	2795.250194	2469.815576	2564.468466
429	hsa-miR-320d	774.301643	807.0876928	875.0110868	968.3850106	1435.408536	1363.107886	1223.935571	1136.118941	2961.352433	2982.694573	3065.939347	2948.721774
428	hsa-miR-320c	2717.05159	2868.284997	2978.733691	3386.283532	2693.998455	2591.072012	2327.562088	2088.979356	40.35039218	32.05673568	0.034755009	0.015658773
427	hsa-miR-320b	2169.405329	2405.378524	2465.911475	2808.442844	2138.836009	2005.516807	1748.39455	1721.771559	40.65357363	19.86136208	5.074021946	0.016800889
426	hsa-miR-320a	2547.703216	2838.942733	2961.385753	3273.829564	2300.27823	2499.98252	2253.406876	2051.586693	50.40743553	52.1799091	36.90647498	30.30220966
425	hsa-miR-32*	7.734701246	34.90988123	63.50955589	40.46540606	27.11080163	22.28329734	0.124211597	0.048259924	1985.07537	2103.19063	1949.26042	1989.980344
424	hsa-miR-32	12.72440503	20.53956724	52.62986653	32.70630426	31.45134377	16.41649407	0.054779027	4.648760121	182.5340939	183.7014068	173.6232787	158.674961
423	hsa-miR-31*	4.115223796	21.3010536	45.04517977	27.51660804	63.2935408	81.54516992	38.16502199	41.10702072	163.8937643	166.2645266	143.8588913	99.49592685
422	hsa-miR-31	647.1704831	737.4491165	781.0442954	676.721051	1927.99396	1927.138963	1688.645178	1854.93894	33.82143059	26.70293313	8.418193208	9.164499096
421	hsa-miR-30e*	35.98438462	47.22687346	78.40143792	72.32969353	189.1677632	159.3825506	166.100627	159.0225041	295.813072	276.8991413	292.6703299	211.4676836
420	hsa-miR-30e	56.01135564	72.439924	90.71543801	74.48698612	170.9566675	152.7642745	143.1304712	121.2269433	60.35110409	52.99343137	34.2514628	33.67938294
419	hsa-miR-30d*	4.734847139	18.01938801	43.48403754	37.20438394	35.55507341	19.24498295	6.992503483	18.30468861	49.44196225	25.58639208	9.820608951	10.00247131
418	hsa-miR-30d	270.6973989	335.7077736	390.8730428	254.1730451	396.6785311	397.6376598	374.2127223	338.1779498	1506.337647	1486.434486	1554.861273	1376.219185
417	hsa-miR-30c-2*	16.85816449	27.36304772	41.69745442	43.13883042	76.52946065	41.73607055	39.14787155	35.31689744	47.64252121	23.67050037	11.08604866	18.93552684
416	hsa-miR-30c-1*	18.4625115	24.71527186	53.55176864	43.69528187	48.44301924	19.44186739	0.046733715	22.362952	1141.361273	984.0180918	964.1022414	873.0367271
415	hsa-miR-30c	678.9153255	737.2435624	788.7400588	779.6123915	1428.624515	1304.196406	1358.246034	1300.531162	358.2560321	369.4790665	328.9118329	307.1635217
414	hsa-miR-30b*	12.26935126	16.92911791	46.94591188	35.22002291	45.73051019	26.47768737	15.71381719	25.99622742	594.4116112	670.7129299	570.967297	465.1053689
413	hsa-miR-30b	495.0287421	470.9609339	514.2394581	491.1973896	993.232596	818.1616842	780.1105123	724.9038565	48.98858272	31.10297192	5.646622989	30.49844188
412	hsa-miR-30a*	131.9657298	156.5623644	172.6081153	141.3666773	365.3831239	362.0820288	339.1993828	306.0078632	41.0540138	36.6965664	7.928849268	23.4472505
411	hsa-miR-30a	354.4364259	475.4657454	439.6336925	329.6960661	688.749923	785.2056285	630.0738963	545.9071538	42.86680532	35.88065391	16.50430041	24.86183665
410	hsa-miR-302f	590.5846012	672.4963186	825.6265856	699.1922301	33.41659508	8.72092762	8.757570488	20.98000186	39.52261366	24.73537739	6.20157071	28.03362598
409	hsa-miR-302e	2.670210529	15.60280798	32.26176226	36.22224763	38.3050025	22.53615411	8.224432574	27.23174025	51.94163609	32.20380364	19.66356138	27.08599327
408	hsa-miR-302d*	1.317122427	11.70799533	44.06596455	42.47828861	32.34718815	28.5419851	5.749222607	26.68739865	41.85258926	33.11420963	2.229605959	25.24358706
407	hsa-miR-302d	0.011831443	0.012475115	49.37106317	33.65895606	28.58489631	7.153773087	18.61673668	27.18234772	46.77408915	7.766373913	0.019455649	21.38993396
406	hsa-miR-302c*	7.98054349	16.16265444	45.89550592	39.95722758	45.07856849	16.1018413	9.831782863	35.56785156	57.71859203	0.018387615	3.289959936	26.33785908
405	hsa-miR-302c	0.011990026	17.52448525	47.04570386	37.81688667	38.81597673	18.36869476	7.913329617	26.01043933	41.63897193	0.018356799	9.084798539	26.36229928
404	hsa-miR-302b*	1.870383066	3.79156978	36.93399038	28.57288946	46.74598227	2.176448588	16.1773722	21.8253447	83.22315082	13.13845457	6.166293499	33.42355602
403	hsa-miR-302b	2.034191735	23.44805161	45.01344102	35.34605036	39.50667431	10.16697832	0.113507903	21.26290598	65.2229222	2.27835384	21.20762994	31.41436039
402	hsa-miR-302a*	0.582585977	12.77385316	47.86266548	35.05073339	39.61380238	22.11451802	12.72937555	26.29419041	165.3388228	51.92905082	57.96984322	95.28877142
401	hsa-miR-302a	0.015338977	15.74960485	39.77174744	28.93751247	58.82359136	19.05590128	4.590640674	30.31734162	37.86756732	12.61940334	19.5417508	34.51691463
400	hsa-miR-301b	1.94339433	8.881867921	50.10689471	33.39328043	85.93894084	11.65574933	1.786871896	27.87692156	45.76066499	6.908591017	3.588838087	32.70364376
399	hsa-miR-301a	20.07902215	17.8729812	52.1241368	49.25801879	194.8458052	85.40135597	63.91476666	85.96082351	107.9875887	24.52059897	39.0011261	46.97501239
398	hsa-miR-300	2.87113824	12.75275271	31.50924939	29.37326515	63.28305672	26.3954775	16.71055836	37.13349774	36.82888756	24.44043207	22.22973434	27.1605533
397	hsa-miR-29c*	0.015346253	11.28368002	38.55360468	29.20137021	78.2880595	20.70323574	9.269990755	33.68215279	88.33955732	66.9512305	123.4307583	90.96416319
396	hsa-miR-29c	31.45142303	33.13643463	64.07108103	41.32171497	100.0366633	25.99589008	45.28539667	33.03310812	49.50374555	18.43408897	49.00835759	32.09134506
395	hsa-miR-29b-2*	15.26396363	8.243985553	44.61008895	40.78516037	24.56434748	40.34031965	3.406723464	31.74593955	24.51212423	6.612134965	28.92381355	27.15235849
394	hsa-miR-29b-1*	38.73346738	39.5299506	76.61349122	72.53940463	128.6019096	97.67399369	200.3170095	106.1022138	1402.02942	1173.498227	1411.068973	1175.557567
393	hsa-miR-29b	12.4083423	23.05157219	59.44948033	49.2355393	24.29447432	32.79104283	36.5035002	28.69323701	69.06186386	46.11194807	63.64350819	66.46888595

392	hsa-miR-29a*	1.599392393	14.02055244	43.91818563	45.92867407	31.75685677	22.40284552	4.067909515	24.69970903	44.57678554	5.261591186	25.87819685	24.06994703
391	hsa-miR-29a	971.9109884	878.4101597	1233.800426	890.1460822	1252.614994	1102.672213	1236.280887	1080.30424	41.72334478	12.09169444	26.91166382	38.73759352
390	hsa-miR-299-5p	11.91509662	20.31752641	40.20027012	48.10042928	83.51913925	77.5750288	72.08364551	70.24866096	2.526305215	24.58920567	28.60520312	0.014777131
389	hsa-miR-299-3p	9.966331686	12.28750759	37.79799904	39.28997983	35.59381451	40.22766633	8.556159201	27.78906645	105.1094681	96.99146473	100.5755186	55.93768713
388	hsa-miR-298	1.433516292	17.14276084	39.60158208	47.95211979	34.37913437	22.03689554	15.17468581	24.08292339	26.23579579	30.73841862	46.26305658	13.46035711
387	hsa-miR-297	8.751391701	25.70840825	44.41374304	15.9746929	41.76535757	1.780305262	17.4946414	0.04464379	86.51428426	83.63950646	81.97599794	59.89965024
386	hsa-miR-296-5p	58.05527125	56.85060874	62.7416374	49.88829021	80.38328631	72.23316211	83.62793466	54.61636542	89.17349328	81.63720901	77.75575665	66.72190588
385	hsa-miR-296-3p	16.75777673	35.02228646	52.46390244	29.41727726	22.36079654	19.24779445	26.32623809	12.24335007	139.5648719	162.0540081	152.9476652	131.2507625
384	hsa-miR-28-5p	39.14208285	50.80738566	69.23949047	37.94624235	105.6178998	99.31447627	102.0631126	75.26257825	1534.833774	1608.8715	1511.48575	1436.675111
383	hsa-miR-28-3p	37.8065148	45.31060501	60.60861158	38.50473232	122.4128545	97.40366567	121.1259534	72.47535484	39.37855324	52.36063144	45.59933862	17.32775733
382	hsa-miR-27b*	25.22391356	43.27192861	55.26696525	31.10134779	208.257663	183.3694845	205.1527464	153.3914914	2290.765453	2465.353662	2315.885423	2254.674654
381	hsa-miR-27b	936.1126	1024.534603	981.853872	1058.52023	1440.492351	1495.845007	1438.483828	1360.413894	11.56733941	26.68141432	15.33737756	1.435971673
380	hsa-miR-27a*	4.759375861	33.16514307	41.33336104	14.00697412	51.46134957	27.3529579	35.9331457	18.15871664	1090.928502	801.9265047	950.9424017	847.3696854
379	hsa-miR-27a	1593.957491	2008.887795	1884.838135	1852.817285	2247.120034	2337.640314	2308.865951	2208.684513	30.50676936	24.08028632	14.91166678	3.680222539
378	hsa-miR-26b*	1.592468031	25.43118943	44.74789654	22.60764124	14.98088036	17.11410636	28.74464137	11.9640264	12.96570813	8.939076712	14.65855096	19.47279222
377	hsa-miR-26b	781.764197	575.7131066	712.3123898	746.9318036	1067.987529	715.8121258	871.2729375	841.1162454	2145.483797	1895.530064	2010.099461	1759.535959
376	hsa-miR-26a-2*	0.015620413	26.1826888	40.64468185	25.14936906	45.64814699	17.89816511	21.73684588	6.204194339	87.83382417	87.62861965	71.54981275	76.81624346
375	hsa-miR-26a-1*	0.012076505	24.53839258	48.21167856	19.67425618	19.02931834	10.29324964	15.76667469	3.4223444	1856.426529	1832.022418	1858.212358	1809.625873
374	hsa-miR-26a	1891.480388	1829.902034	1999.816042	1798.213334	2006.457033	1700.183422	1782.331542	1736.487516	160.9015502	153.0703949	154.7400833	158.9773505
373	hsa-miR-25*	3.281921444	19.26623919	54.07791814	28.91299682	124.6600203	100.0445086	127.7194216	99.19147322	21.522202	19.18677918	28.44226082	18.74350814
372	hsa-miR-25	906.9215145	937.8665614	983.5774368	1074.520141	1714.521108	1600.620172	1664.282781	1590.495908	3581.352445	3838.048332	3925.35279	3811.090165
371	hsa-miR-24-2*	64.63453201	87.0491412	113.0271843	87.55352195	171.3550891	173.3977396	198.3796404	171.8853323	38.27218887	37.55309544	36.46193985	22.07931937
370	hsa-miR-24-1*	0.328956596	32.16085205	43.85708092	26.84414072	30.54363415	18.6909488	21.08812714	12.65804613	9080.224322	9659.393132	9666.849296	9713.425391
369	hsa-miR-24	2027.235687	2473.400965	2719.644627	2596.617473	3567.215789	3592.958355	3666.568668	3423.868334	333.1819532	231.0658029	238.3080565	244.9914815
368	hsa-miR-23b*	107.1539965	166.6634607	195.4860903	160.2471638	40.03761784	31.85719827	42.35956873	16.59889705	11537.05122	12545.47222	12553.6925	12698.4413
367	hsa-miR-23b	7155.143809	8569.237348	8872.942666	9022.330806	8480.467263	8643.6739	8708.645964	8341.446611	77.02368473	45.47652499	43.04722171	43.44488267
366	hsa-miR-23a*	272.1110168	326.2363785	365.1095471	278.9251646	276.3601892	227.2794441	247.4114074	209.4929373	4680.510569	1582.865501	1826.376841	1729.840123
365	hsa-miR-23a	8809.774928	10626.22958	11222.03923	11483.30197	11361.89518	11805.3735	11701.57512	11394.84853	57.82736938	22.4005277	32.07458075	30.76743365
364	hsa-miR-224*	21.13623016	49.17776746	64.53497814	46.13199214	67.99816023	42.45632757	55.10978252	39.7950935	48.65862559	32.09832364	37.32766251	37.42247179
363	hsa-miR-224	1600.452518	622.5040663	751.576481	729.2989745	5107.459796	1418.482459	1673.501832	1496.737666	34.92672721	26.53290974	36.28601704	21.63892402
362	hsa-miR-223*	0.015508707	20.83353814	35.68060726	20.34597213	44.54843069	18.08202336	22.92778459	22.98893652	1177.694417	1193.633757	1233.409963	1254.318555
361	hsa-miR-223	9.880809457	24.19177421	49.74842163	34.52263754	62.83932123	46.83552367	50.06525945	32.47912339	40.26309854	20.6086838	26.18360083	24.90457677
360	hsa-miR-222*	12.60240286	31.87996564	40.86284346	32.33015545	49.51243437	23.87096945	37.8875826	16.64164536	783.0478039	844.7935429	865.2087965	690.6499823
359	hsa-miR-222	485.0616115	581.9792732	595.119579	627.3956457	1095.630912	1028.259926	1022.645744	1025.376213	20.23047945	42.32238507	23.57180575	1.357738018
358	hsa-miR-221*	0.015581262	25.34035684	42.27297616	22.2317292	44.03053853	23.47369731	40.60633552	28.7396083	43.68005151	41.3756175	30.17985805	6.549880374
357	hsa-miR-221	427.8367843	473.2764622	497.3305533	456.2220574	882.4638456	884.9060811	901.0393784	733.1033085	37.0350618	46.0308179	29.24871891	13.56330019
356	hsa-miR-220c	18.8886623	26.93625578	17.66556367	45.90519228	22.99492132	25.26850433	18.10118122	0.042705401	53.64857671	61.68861494	49.70476634	29.72232304
355	hsa-miR-220b	16.01445003	32.96071634	22.41677474	44.21215073	38.76715691	37.43646681	27.44182844	15.71993258	132.8103067	190.8356662	183.4444953	117.7402138
354	hsa-miR-220a	25.91058887	37.41172095	19.77095526	48.48967712	50.25838397	60.13577146	35.98092654	11.90250948	31.96581651	20.24249217	10.12069435	0.08151875
353	hsa-miR-22*	33.10953348	47.48519153	37.83508186	59.76933902	71.21110869	64.43717421	47.63315368	49.62959773	41.17916076	34.55157385	24.48906808	0.015181443
352	hsa-miR-22	98.60993508	144.1624064	119.4640521	125.7021224	147.4713669	220.1516907	195.5492193	140.3000694	39.19982406	53.12286385	27.75933112	3.293154895
351	hsa-miR-219-5p	22.79623958	35.29098508	24.54753745	58.2341838	22.80423689	37.50231103	17.69544638	0.046155413	54.5639446	41.71084738	35.42863539	8.522107917
350	hsa-miR-219-2-3p	11.00807908	35.32635001	18.53605991	57.05383369	23.59258446	38.33973882	12.88863065	0.046692537	40.61512285	42.43987536	29.38018609	4.77884022
349	hsa-miR-219-1-3p	18.81945444	33.39423451	20.26325031	48.23523483	28.60557394	18.79812938	34.22053128	4.870958026	41.48259916	37.06196787	5.53366842	1.960261894
348	hsa-miR-218-2*	27.09271403	36.00422478	28.26058184	62.9707302	32.74225231	41.50903313	28.7342321	2.238158682	39.36999155	25.06716158	3.86636684	3.112104874
347	hsa-miR-218-1*	12.78089273	30.89008463	21.45252393	58.32631949	27.02393475	39.86803465	33.2376581	0.042095747	39.54612015	39.27408571	4.255292972	0.015784002
346	hsa-miR-218	20.13380416	24.14192437	19.64117012	64.25736911	21.09598753	26.76847936	37.38039596	0.045002907	27.6708812	21.11884729	2.544676934	0.016706096

345	hsa-miR-217	16.48694577	29.84980518	19.67015486	42.1646898	30.69750437	25.52732088	29.03890158	4.867126942	51.26828069	25.38715237	10.99967497	16.53403891
344	hsa-miR-216b	18.23363927	29.6075252	21.16329039	58.9384256	23.46536056	36.95426806	40.96522419	0.0489034	44.210737	30.52433064	7.297967096	11.69330037
343	hsa-miR-216a	10.87581897	25.49161125	19.83071469	51.52137974	17.28590907	34.09922469	35.34800316	0.052220008	39.81483276	38.86029281	28.65529012	31.58647538
342	hsa-miR-215	12.76977991	24.08489645	27.30638341	53.00626166	27.44247702	38.09501381	14.3561935	12.95702713	112.0044446	104.3469135	113.1835499	100.3375797
341	hsa-miR-214*	4.877717043	25.17823118	15.91075294	44.57530654	26.20843659	34.03016313	16.43935859	4.858513798	42.03713903	12.99179851	11.83230179	9.637238581
340	hsa-miR-214	26.07457141	36.1302382	33.94434452	66.16317789	49.27192554	55.40421721	38.27594596	34.95416359	59.79975007	46.05571299	46.09583226	39.95883235
339	hsa-miR-212	31.69865653	37.76084635	32.39790688	56.82322323	118.0085617	161.681877	133.8684121	107.0339344	37.02825562	29.6291481	17.07902543	26.68489843
338	hsa-miR-2113	10.5829311	15.76910901	23.71069217	44.24351543	27.49668206	28.11766015	24.65886721	1.053806774	44.98400369	32.49610339	55.4009343	31.28192717
337	hsa-miR-2110	20.66532482	28.02059677	23.88835759	56.57879917	34.29528275	40.87923976	58.46429879	8.718097701	61.1858704	28.99104527	29.45228841	35.87861655
336	hsa-miR-211	3.515775917	26.01852426	25.91194343	46.32094609	23.13525288	44.49537484	27.06538425	11.29559412	12852.17495	12831.27298	25383.8535	12223.20713
335	hsa-miR-210	26.63342354	33.82188142	52.72345778	58.82397895	33.81994509	49.38194195	82.54484232	21.53537762	94.35519015	22.23516647	25.42544318	29.12334104
334	hsa-miR-21*	15.44721759	12.14443947	16.00856796	54.78222269	40.7818291	35.30516415	32.45564658	9.402892941	2394.798324	2113.24393	5037.229204	2073.81926
333	hsa-miR-21	9733.767611	10518.65419	25562.47716	12459.41256	12879.35569	12883.64127	26423.70584	10883.45212	90.69963401	30.09224347	21.34385446	17.76186383
332	hsa-miR-20b*	4.823179329	16.38007764	21.55420452	43.53306052	80.58329704	40.43249929	17.75257755	8.662589356	4938.567108	4370.46624	5200.531348	4305.631243
331	hsa-miR-20b	1094.03612	1116.83449	2987.81924	1424.737726	2019.057826	1736.30579	5160.221799	1674.491582	54.00031642	26.07153045	20.35341648	19.55170397
330	hsa-miR-20a*	11.91109376	20.51964403	27.02717874	51.67701391	106.5718097	48.69909972	26.07410567	5.829695735	32.84029836	2.990096375	18.00756041	0.015649658
329	hsa-miR-20a	2401.126122	2314.522725	2971.493512	2687.198923	4996.418207	4205.086908	4891.323894	3628.39823	109.7434615	87.29677872	101.2381248	80.54649966
328	hsa-miR-208b	0.01525985	23.81708387	27.71859435	53.01355161	91.27593464	34.03729657	43.76520551	0.101586194	0.026049162	18.94892563	9.601769112	3.274421453
327	hsa-miR-208a	21.19678772	20.01180564	49.33979503	65.41235197	40.10625186	17.75082907	0.049272036	0.04846421	12.85851777	18.20177292	9.281588016	17.29662662
326	hsa-miR-206	17.48572258	21.0626638	49.23663876	49.58657895	69.32416656	46.95710835	28.11476203	44.21116651	21.96726581	36.13449693	13.34238561	1.740341728
325	hsa-miR-2054	15.27610079	33.55106687	61.40989498	41.93612938	28.60824243	11.63611788	7.178042068	2.157464915	22.5298591	14.52868321	6.482405718	8.221750967
324	hsa-miR-2053	20.9425079	21.27745509	47.78547367	52.93535625	44.29130326	29.42002436	14.61643354	7.770511115	43.31455685	23.50960538	18.05312845	15.52990109
323	hsa-miR-2052	15.01987387	13.5641188	42.90169173	46.09478623	23.55532086	20.42409268	0.445284814	4.517471978	30.16286123	8.354619234	8.71050408	13.05165217
322	hsa-miR-205*	19.11387269	18.06690338	56.4299836	44.15184701	24.66403734	21.20410249	0.051941454	11.90441085	91.32380231	82.43538086	76.43621494	60.7236063
321	hsa-miR-205	17.72032261	10.04004564	48.1575647	46.53143134	34.47984157	33.88530448	8.978151984	19.51709612	30.28356266	0.020728159	0.034772839	3.909146048
320	hsa-miR-204	17.60670226	17.92645915	47.21488028	54.94037898	36.70584891	21.57693835	26.10546909	7.477080201	53.61759106	31.82180222	20.75619441	19.74023669
319	hsa-miR-203	51.03987885	49.5839718	78.15026544	77.3384424	122.3828518	82.80835688	77.68773163	84.18535112	28.17863061	34.94994411	19.14514553	15.23803622
318	hsa-miR-202*	13.95404553	18.75436178	65.92956703	51.51993041	27.42033409	11.89826672	0.12433434	5.357588438	24.18547869	50.88679551	30.98613493	39.79083925
317	hsa-miR-202	16.55339703	24.23003744	42.04800539	54.31511969	29.70991024	31.89989353	11.51661761	11.05250745	40.45843366	22.06994447	14.51364686	17.50467972
316	hsa-miR-200c*	9.198971634	19.3089581	48.63877824	46.9951582	40.01768101	34.73958821	16.50862855	13.92987476	52.2147143	10.92213135	3.724788557	3.881725491
315	hsa-miR-200c	29.71926518	42.45216651	73.30739746	73.32075086	86.17713878	103.7537059	63.19512956	68.88854779	38.42925375	25.33758213	12.44588243	14.86493526
314	hsa-miR-200b*	19.45160974	24.73665993	59.52488413	62.31771676	31.70726749	27.98158745	7.900103297	17.41598261	30.83203687	24.66456689	4.129401482	15.98153603
313	hsa-miR-200b	0.86480842	19.38738489	49.45016627	54.8424648	47.66476039	32.82688039	0.054422527	15.16131525	23.90637589	17.95924928	6.10123523	18.75193305
312	hsa-miR-200a*	0.011559536	13.48219814	49.91719987	52.85714189	24.49808105	41.4007727	11.56717172	17.40194527	41.25664147	26.5178624	23.45044246	31.79480527
311	hsa-miR-200a	2.530830753	10.59340569	50.40378354	56.44803477	37.77042227	13.88011413	0.048411431	8.970510412	1598.743404	2028.52739	1804.392077	1471.009684
310	hsa-miR-19b-2*	0.01148583	5.304361123	45.84741137	49.00389777	33.54496325	15.41139259	0.050057451	23.58121473	32.47222544	10.68105953	0.017034404	25.38524597
309	hsa-miR-19b-1*	2.223117582	9.509749097	37.19349276	47.34862322	28.7859543	21.66688225	5.66092212	29.46911591	145.352482	68.18991506	110.3089221	84.35070814
308	hsa-miR-19b	362.7101183	446.3749855	477.3564556	339.8486774	1528.113752	1795.847412	1592.2673	1251.150302	36.92156473	6.436395139	3.864406791	28.34750339
307	hsa-miR-19a*	2.258273065	7.78529322	42.42877594	41.51415936	26.50253213	20.71352675	0.053327028	22.38180389	49.17368737	11.51840319	12.75788277	21.41940613
306	hsa-miR-19a	14.7031279	17.23863521	59.37994792	49.60706987	114.8718061	67.31090174	129.2362936	80.04571759	73.10951787	6.187032242	7.267108809	33.76194111
305	hsa-miR-199b-5p	0.01272302	5.246269113	36.68714886	46.34789958	22.55790632	31.26568037	13.87016747	19.85130396	176.7511052	25.73755709	38.12098244	53.86073575
304	hsa-miR-199a-5p	6.444558995	5.670248904	41.67704822	45.83458248	55.52519233	33.3299957	6.130113548	28.38530667	7490.463358	8453.915434	8206.628559	8602.320835
303	hsa-miR-199a-3p	14.78995908	17.3079346	53.40368335	59.1162625	80.1174343	32.5047147	0.705420737	26.12735998	260.3740736	257.3443038	251.6773908	310.0490523
302	hsa-miR-198	87.02335063	64.8308463	89.67671614	99.69448724	126.0781686	45.22528238	25.29278778	36.16929232	1828.041617	1793.721134	1713.306254	1717.640564
301	hsa-miR-1979	2365.791976	2974.351585	3256.506714	3320.790083	10003.46002	10660.72349	9644.604524	10618.20036	65.5883524	15.86274747	23.76774195	41.91458247
300	hsa-miR-1978	533.9903843	712.3139801	771.9226675	785.1431719	633.6029017	650.9351486	589.4444589	655.2713698	9122.818654	8938.570251	8854.21407	9354.384358
299	hsa-miR-1977	978.3719816	1062.055405	1133.366851	1126.760753	2855.372879	2851.256365	2582.115743	2680.162862	5891.769864	5918.564729	5803.902751	6140.519273

298	hsa-miR-1976	0.015230903	13.80318677	44.16357351	45.70115276	89.51035693	39.27760028	11.27944242	36.60427859	47.45445054	25.70394893	34.2889696	33.98156609
297	hsa-miR-1975	2911.414903	2940.226894	3306.473852	3272.764243	10611.3995	10462.4434	9348.5228	10464.78212	50.16583386	31.05544483	36.17811487	40.37513383
296	hsa-miR-1974	2739.13969	2807.800162	3133.918202	3145.872725	5309.99817	5273.938488	4748.263295	5261.924132	174.238847	151.7238965	183.5176473	148.2870599
295	hsa-miR-1973	20.66635348	41.23967355	49.72530648	46.74211638	50.05586341	63.05291041	34.73833498	39.90675462	47.70093331	28.26387604	45.49722314	38.12089712
294	hsa-miR-1972	10.31773583	20.53664846	32.91839587	26.44944762	30.98443602	37.79147812	20.51805678	38.50241159	55.89739952	22.39873979	89.41305162	34.49243129
293	hsa-miR-197	28.61839277	40.13667513	53.20660469	42.25810157	197.1074133	192.48958	198.2642416	171.2417077	40.41535777	30.0724492	26.42806181	21.86898675
292	hsa-miR-196b*	6.599486927	28.18380671	30.60348443	35.16460814	38.09569848	25.77651494	19.02077026	22.45071894	730.0316594	2067.002868	738.2153747	710.327034
291	hsa-miR-196b	24.61531965	26.96934878	38.52451546	41.3227012	44.54722961	39.47944099	53.77949397	40.53639238	29.35345728	29.14230112	42.50663693	5.706382665
290	hsa-miR-196a*	12.77590746	21.21698914	29.02998979	30.96978441	38.14863234	30.67608202	15.02895864	18.9446368	179.4737941	202.279943	189.35162	131.4234795
289	hsa-miR-196a	341.1308197	604.7091523	354.8783233	402.3269655	801.4428046	2016.8027	815.9757467	753.1240165	29.60095709	31.3155133	9.894836699	0.016177538
288	hsa-miR-195*	20.92536358	29.05217955	32.92584324	53.30373802	48.00094025	11.91669639	27.34293968	0.040462316	66.07391286	64.64664003	70.43573054	47.18158908
287	hsa-miR-195	108.2194432	82.85243909	101.5404389	101.9397514	192.8027177	156.0612073	161.0675195	131.6387721	143.6984104	139.5194864	133.4440426	109.7355622
286	hsa-miR-194*	9.621605118	21.94403084	39.38298597	50.77887626	18.02875971	9.434737983	21.00000117	3.779797998	53.46115228	34.8680189	41.89156964	22.51473141
285	hsa-miR-194	30.82086607	38.42828836	40.35903351	62.0198731	67.3478652	54.7773598	82.83324393	51.49516109	111.0385447	134.9793436	94.3334333	63.1912968
284	hsa-miR-193b*	50.65572051	52.4843849	67.50054647	69.67898322	128.7882415	124.5003071	124.7942392	107.1490997	35.82781576	32.33948122	22.17960495	0.016382579
283	hsa-miR-193b	31.39548062	31.75450743	44.52734345	61.37545044	54.2724328	44.13473304	44.90112737	21.33004142	23.64803815	25.29404641	22.9914277	7.528173047
282	hsa-miR-193a-5p	71.26150081	85.08560252	71.85938721	78.5322199	107.1867654	114.677813	112.4186556	93.82334571	129.034461	151.3599538	127.0810436	117.6146348
281	hsa-miR-193a-3p	20.92095188	32.48632116	30.71803984	57.33766507	33.88702169	29.70457068	24.76644609	1.108085009	43.58059983	38.08031243	37.69250651	11.02846203
280	hsa-miR-192*	1.130324795	24.45675332	26.25267242	47.1175354	37.93572583	22.35277312	19.16048443	0.042634675	399.5555324	388.7668091	377.6641408	347.2865843
279	hsa-miR-192	67.21268433	82.1370522	69.63402092	89.16430645	133.4866042	121.1593497	125.6540432	98.04791934	44.13825515	47.84295557	54.97768069	4.410485043
278	hsa-miR-1915*	12.51713753	23.63909285	24.62646107	45.42770891	39.36552688	21.70592414	31.67964344	11.1975253	89.61615186	78.30634279	84.66346044	65.09250567
277	hsa-miR-1915	2040.619143	2458.590642	2750.214712	2652.572327	391.2187547	403.550713	407.8727828	356.8519984	104.2482035	85.93766089	91.34450703	69.28322791
276	hsa-miR-1914*	14.03695679	21.5504494	23.00451673	53.41199627	39.73577907	31.50730427	32.93253404	11.7572381	32.6967086	28.47124098	48.45342638	14.21403052
275	hsa-miR-1914	11.89678941	27.11620481	35.2788505	49.80436023	90.79057095	73.40507002	95.05822267	68.63282194	32.34756084	30.92387244	43.32385213	12.8686051
274	hsa-miR-1913	32.71690338	38.5728809	40.6484163	55.77557855	84.40179143	73.19682554	74.02219338	64.96757796	33.4839556	40.74995867	47.45092455	29.80768116
273	hsa-miR-1912	0.011391971	18.73001943	29.92496666	48.75740492	31.11594696	16.50455121	23.60150649	6.557352423	47.70508617	55.04875155	57.92129152	31.88642802
272	hsa-miR-1911*	2.951486977	31.1856563	29.42597206	48.65674662	34.95548455	28.71733497	22.15288379	13.80580443	92.87440221	74.87573537	64.65519855	65.70770473
271	hsa-miR-1911	0.011347113	20.78678569	28.65867036	41.25845151	37.48496806	34.92362908	42.3427666	33.07972428	798.8617117	815.3566458	794.7441763	802.906448
270	hsa-miR-1910	11.70184547	20.67219185	35.39108375	42.33163147	43.00651088	34.96426011	36.33074949	32.2484696	42.9158514	26.95354388	31.46678962	13.9859233
269	hsa-miR-191*	19.49712292	51.48726812	48.41470051	67.01579022	66.64035479	58.82083993	54.52559727	42.08896142	52.85095344	32.14906722	44.50874532	35.69072587
268	hsa-miR-191	687.0039899	776.03161	729.9950343	803.169732	788.3040575	808.6011358	790.1457501	764.5639028	58.73842184	48.71348201	63.40696936	38.50256315
267	hsa-miR-190b	11.59759035	17.35612149	30.03480606	50.34545116	0.104589056	22.98519708	32.6554655	0.051569127	86.17372807	53.5622815	73.40105081	41.4550486
266	hsa-miR-1909*	1.311488317	19.10527718	25.57337168	44.41176846	37.50758025	22.76679584	29.44047573	15.689326	36.08600577	29.46052074	21.83474615	21.80005858
265	hsa-miR-1909	0.011788909	17.11974577	23.05535741	43.88504495	38.4098221	34.3442307	31.48252564	24.50087975	60.6329786	54.01043573	49.58133823	64.47268685
264	hsa-miR-1908	132.3899546	88.88174311	92.43416504	119.5785888	59.0443347	49.56848733	31.43061425	44.53806321	248.2558253	128.63886	136.2474564	137.2072048
263	hsa-miR-190	3.655406956	19.02394415	20.78489738	42.29108386	58.88658735	0.180999645	12.12972036	0.053934987	85.09147543	42.41566546	62.70125268	29.18258254
262	hsa-miR-18b*	17.8051007	22.79504967	28.52812933	45.75049996	45.93672131	42.62527204	43.90920339	37.89329866	525.5999687	466.4908818	514.3343539	341.1582212
261	hsa-miR-18b	32.76357193	38.58275004	51.74809286	71.28597413	296.6125856	153.4623664	141.6378929	308.9847219	169.451014	159.4017627	127.7825183	87.71083042
260	hsa-miR-18a*	0.015111137	2.468275879	13.13083513	38.37624428	109.211453	52.20849949	46.47986834	31.15485355	40.49183225	49.97062513	18.50142547	0.015062066
259	hsa-miR-18a	92.58988113	83.70107337	128.5512894	99.69744396	566.0789696	489.1461304	521.33712	404.2646586	70.28828543	54.38145806	38.46016174	25.84457322
258	hsa-miR-188-5p	65.66983108	46.60529409	75.4292281	67.83521629	116.7177566	103.921839	98.15971151	68.52217902	30.53974753	23.9020026	18.98552789	1.900261506
257	hsa-miR-188-3p	15.94845649	11.92724659	35.62546063	48.75229786	28.88913994	30.09429954	21.04568041	0.046185297	39.7899733	29.64293992	31.7293831	4.249959609
256	hsa-miR-187*	20.49684716	21.94385342	46.32616136	50.49642173	35.59178842	37.05830824	21.2619613	13.54182755	157.2602189	125.2262535	106.9869268	89.065703
255	hsa-miR-187	22.35743318	28.20174741	47.87353174	51.02315803	16.94598153	28.03687628	13.06951902	4.923560012	42.29405563	35.1271792	19.35294628	8.751667985
254	hsa-miR-186*	653.7534581	372.239465	915.0273936	669.3899629	30.7990472	17.68651589	16.89216958	0.047158703	183.345182	170.5247752	371.7602331	134.9546136
253	hsa-miR-186	21.38457105	32.57017737	88.59992228	61.61790863	127.5824226	141.2334198	103.0882282	93.19872081	46.74054379	23.91479755	17.11267493	1.325248114
252	hsa-miR-185*	11.8851932	17.23836557	30.247445	44.03077189	18.91777362	34.64083265	17.04451904	2.476251175	55.22953455	56.47714885	44.68181256	20.5329274

251	hsa-miR-185	91.4737313	103.5241275	172.1895758	127.0016727	222.0978522	220.5924742	503.2771121	168.5649268	532.3604265	496.3202425	541.9380672	398.7637787
250	hsa-miR-184	12.47331504	14.85572461	33.32378244	51.47328554	33.65899335	26.15211247	21.78291087	0.044929558	47.29601844	26.52526611	14.65975947	2.61343635
249	hsa-miR-183*	14.61893594	24.4856581	45.90278055	50.35920978	58.29876574	53.57421305	39.93087911	22.60377666	10861.77864	11056.5221	11010.08553	10377.34115
248	hsa-miR-183	206.9627722	216.6848758	299.6742645	249.9713776	513.8774443	493.1308614	649.0647196	436.1121728	345.4373217	305.8646814	286.4548586	303.7331981
247	hsa-miR-1827	0.480350111	13.8851714	31.2126799	44.32932578	22.36302662	26.81442735	24.68222818	0.042960674	47.28029951	24.14481482	3.477361633	10.54250508
246	hsa-miR-1826	15162.8437	16562.30135	17512.71805	18102.31353	21488.31232	22634.75012	20528.49901	18278.56966	1224.103597	1185.21703	1331.332164	1277.863831
245	hsa-miR-1825	22.3884346	23.27993898	34.56510885	37.31656925	97.1385311	84.40977868	74.62694267	70.82355761	279.5456573	228.6751603	392.7967449	234.4783228
244	hsa-miR-182*	10.29031713	16.6575093	38.14322835	53.46820609	32.98220407	19.37513313	19.78051697	0.046542567	36.51050044	31.98371844	26.48900266	23.58099583
243	hsa-miR-182	527.7007863	539.5058302	648.3102932	764.2207138	1368.289774	1354.874477	1408.723503	1265.004605	49.29476234	33.35016048	28.51086676	28.66023878
242	hsa-miR-181d	41.87525124	37.8308449	69.00836785	89.88319036	257.3763944	223.7447824	481.4509811	235.2342543	1259.899945	1013.290976	1266.118645	1107.715276
241	hsa-miR-181c*	10.10766306	15.88278108	37.05256338	37.41050294	37.61206626	21.88802104	6.084033249	21.97775593	198.1738266	141.0720446	157.5238354	145.9039857
240	hsa-miR-181c	7.414426243	16.19277405	31.78880874	41.22837443	46.12129004	28.75832897	30.70908228	16.69239099	57.30236195	55.11910784	59.90674035	57.16308799
239	hsa-miR-181b	119.2756494	101.0222332	146.6374895	149.6789571	1209.891802	1022.505895	1238.840667	1018.106256	980.5114439	889.8922338	998.7203502	929.108704
238	hsa-miR-181a-2*	24.52443305	25.13533102	38.30763747	58.25630194	169.4952606	128.2343982	160.4896653	124.1389348	71.25624232	39.84663541	50.52300959	34.38825295
237	hsa-miR-181a*	0.397625763	19.98718422	40.6175833	45.84407101	51.31750444	50.26848342	58.93141831	31.43226112	3633.831114	3567.131385	3663.102042	3487.417487
236	hsa-miR-181a	382.6962815	415.9658602	449.9375162	507.0262157	1110.048568	1086.488099	1049.896916	969.2992743	177.5399837	89.1482178	93.81971705	98.86636058
235	hsa-miR-17*	6.384347324	22.68651968	56.94702099	50.86442905	67.23997404	58.30555998	92.39027956	34.98716441	68.95283718	31.18515693	22.48191981	24.04932685
234	hsa-miR-17	1846.336242	1833.72219	2211.067891	2251.69145	3490.240044	3310.394725	3213.964161	2887.315833	3016.987849	2531.534328	2837.508324	2794.406441
233	hsa-miR-16-2*	52.50109332	36.37683561	64.72630571	70.23708425	251.8273564	106.3063959	160.1837406	92.60157116	145.9959514	50.28094049	41.06686127	39.46015232
232	hsa-miR-16-1*	0.015379857	20.64446654	29.8022146	48.39805593	57.88462139	21.56784298	24.40310266	0.048026103	1589.623269	1610.619978	1564.954915	1559.228399
231	hsa-miR-16	1761.203866	1560.023906	1936.300261	2108.942887	2808.554311	2341.175802	2344.937951	2254.547559	46.66971039	27.15265807	15.13854896	2.65971527
230	hsa-miR-15b*	3.322692904	17.46466255	37.41226786	52.92766015	140.5150494	47.18060044	46.38057777	21.82188663	107.4974824	136.4551673	106.4534428	78.19577585
229	hsa-miR-15b	1165.036633	1309.294396	1418.159447	1388.37438	1733.227603	1658.910503	1550.619047	1751.051349	23.93146108	41.37001734	0.01784166	1.700103205
228	hsa-miR-15a*	10.73398631	26.5626363	44.1717756	31.06681176	17.29912457	18.35556465	1.425204959	0.039472064	536.7747092	647.0240553	550.540509	504.7039614
227	hsa-miR-15a	42.74246219	66.76222425	65.27205094	62.55755142	122.7813267	163.8092637	117.3387719	106.3898991	39.57761852	26.20307567	10.04669026	0.016337586
226	hsa-miR-155*	3.069659003	38.21765282	40.14548568	34.64032573	17.66443503	36.79416645	2.891682898	11.51251055	33.89571638	22.70669249	8.823691434	8.704623475
225	hsa-miR-155	270.868829	363.7297701	332.941298	290.5986886	547.1693638	653.4196751	493.0273388	574.7046065	46.47390591	35.78391326	16.27104639	10.49939382
224	hsa-miR-154*	10.33129396	32.64997486	52.80391972	34.8779982	16.91312647	25.70576904	0.059318103	15.95906337	37.36671615	37.44561261	17.24928538	7.583519476
223	hsa-miR-154	7.351495397	34.82092481	38.72524766	25.34015508	23.53948969	30.88119752	2.955760551	10.93410081	30.96670198	24.25749754	1.538493722	8.32581097
222	hsa-miR-1539	7.764812375	34.0345499	36.64963376	30.28149093	18.78127401	31.89006227	10.71039698	12.93932039	26.84601146	19.55876859	2.172202152	10.14180203
221	hsa-miR-1538	11.60854482	33.72671855	49.16401621	27.79655746	28.54815755	26.23294765	5.123888446	21.35101383	50.22297432	37.67596478	15.21324544	19.66188495
220	hsa-miR-1537	5.736796304	35.10208657	63.01275845	36.1983567	21.31120866	19.23424802	0.116089145	15.31538429	971.7512924	1117.981736	1158.143649	881.435252
219	hsa-miR-153	0.011827865	28.71112843	48.71026582	30.81857691	8.384845506	20.97631971	0.68110585	17.05863102	198.8431223	259.8696905	221.8505569	162.2395231
218	hsa-miR-152	48.32339863	72.52661812	90.69756503	73.70395638	29.81372825	44.47652191	17.5541399	15.0610223	150.4193988	116.0850663	108.8518762	104.2957406
217	hsa-miR-151-5p	261.227504	355.5929285	474.2463698	374.024084	978.2763285	1094.690813	1124.180108	896.2760046	64.6159833	41.94227807	34.94352331	57.04827561
216	hsa-miR-151-3p	87.398834	131.2623958	151.6437946	102.4551746	206.6390945	283.6477332	293.7038028	193.2493428	181.3370417	175.8357668	153.7285313	145.3200955
215	hsa-miR-150*	223.5267765	287.899749	325.5636625	262.2895208	68.70336555	73.32679082	68.84718086	62.29897178	64.85786044	52.03247418	41.54619635	59.05569154
214	hsa-miR-150	96.92484868	130.7722012	129.0294824	129.2944397	90.02412878	104.3782325	70.42108045	89.21759573	25.71940784	24.65971741	16.78932975	33.65626466
213	hsa-miR-149*	1902.243444	2177.337998	2311.764043	2438.01816	110.6491254	107.1840903	92.08870649	93.68120209	206.011696	226.5281115	216.8734559	157.6388871
212	hsa-miR-149	26.44132153	42.94674561	40.2652603	42.30663115	53.35205123	43.45197718	40.13283212	51.4211719	23.48332664	22.87867775	44.30880572	28.86652746
211	hsa-miR-148b*	9.349887362	25.44819161	40.44436171	34.41104795	31.23051331	16.26404086	0.052868827	24.2264843	27.57398889	29.70032346	25.14091483	32.21708838
210	hsa-miR-148b	59.10160165	61.9612463	87.15599355	86.14692322	250.0587339	275.169563	269.795614	215.3064589	25.37157382	11.1089325	16.8901706	34.52097407
209	hsa-miR-148a*	3.018079578	28.57693846	51.7875405	36.89831498	31.32210826	24.81460949	10.90197428	30.02064994	56.31282168	40.92407887	24.56143925	44.11317413
208	hsa-miR-148a	7.38240014	28.59537994	48.81941188	36.0295743	18.51896578	28.5954283	31.0019275	23.45925758	76.12815677	33.54434534	34.84713274	58.04747963
207	hsa-miR-147b	2.734311362	17.47293477	41.13656983	25.03431395	14.43218091	31.77034251	0.057689502	24.97977369	31.47327423	12.07589777	18.19582841	48.64356689
206	hsa-miR-1471	3.291329103	22.26890752	39.97706018	30.33611222	16.25221588	20.97994099	20.87207983	23.0463927	96.96516678	30.95856697	25.14363097	45.98076068
205	hsa-miR-1470	22.24845008	30.06228584	34.51278696	33.74921389	44.91254237	69.97792325	26.59910707	42.39412303	35.5008241	18.36915574	23.49313523	42.95955231

203	hsa-miR-146b-5p	55.39765862	33.4912335	45.25708993	49.09023524	107.9598503	39.21676644	26.7174079	42.67260598	20.02001248	1.71576155	17.44755633	24.97949363
202	hsa-miR-146b-3p	3.534801717	19.89900402	32.27871213	21.50035964	57.502225	33.97862405	12.42052829	31.99261784	60.35219877	30.26125788	43.73425081	49.81818697
201	hsa-miR-146a*	0.01582859	14.63074072	35.08649163	27.69973682	32.39558674	29.68386361	18.19156064	18.82373505	334.5637114	325.5350092	328.1189918	340.0531712
200	hsa-miR-146a	949.8921329	719.7897184	905.4910934	641.1856445	41.01552708	30.95574227	27.40126523	30.1622608	17.73459139	28.49881217	15.72057425	29.39266627
199	hsa-miR-1469	1323.532077	1526.530424	1559.323443	1622.401407	201.0317826	199.0175346	204.4505496	208.8080933	23.12196451	17.585779	56.89790116	34.52997049
198	hsa-miR-1468	4.649892778	24.04294006	28.47974141	34.14094618	23.0313894	28.80786174	23.81806104	34.88442515	128.1937976	151.5350173	174.856389	125.4717371
197	hsa-miR-145*	0.012058162	21.52013571	37.31893285	30.15745802	24.41650341	27.4874706	48.49745694	34.26062994	15.01369919	35.87532766	35.08842441	18.91232543
196	hsa-miR-145	15.77466382	35.41156736	46.39145589	48.53875418	185.352338	184.5171186	215.5769622	157.5515231	19.56896725	31.14759479	39.72546404	37.33937875
195	hsa-miR-144*	0.0157389	25.2162742	19.61003133	28.48129976	36.87163408	3.295262823	13.32592834	27.88403164	190.2189629	208.626633	213.3573246	167.6852799
194	hsa-miR-144	0.012190278	14.0149797	29.21814359	26.24906944	11.92718655	18.31750235	35.79545901	22.17865618	24.50490594	28.18862201	24.04604663	0.015906971
192	hsa-miR-143	12.78078561	24.19812495	26.40295211	34.03574327	228.1433366	250.7960192	235.4024883	175.4037629	12.51295636	32.73909093	17.68341912	0.015924067
191	hsa-miR-142-5p	21.77226627	17.01302373	24.7284621	45.19111661	27.8401864	16.34838948	30.79889122	0.049219381	24.23033018	25.1784252	29.77651175	4.741570421
190	hsa-miR-142-3p	24.13575722	25.44231086	27.38574426	43.43656031	21.86056761	15.32959917	26.24529201	0.04926185	27.35015907	15.75285696	23.91588884	4.788029718
189	hsa-miR-141*	0.012137801	22.2726963	24.42928217	41.1814438	14.77840874	11.54959405	22.91910927	0.860873598	33.36684766	32.09805604	35.77086712	13.58275844
188	hsa-miR-141	16.95490453	25.44341282	27.10019415	41.8573219	15.35349549	15.0578975	27.13632301	0.043381369	160.8801113	163.9531961	136.4752981	119.5236061
187	hsa-miR-140-5p	22.21504718	20.67536355	19.20886633	40.71135753	21.91446703	28.30218096	31.0256694	6.337349371	403.777007	407.8588154	361.363238	352.6077226
186	hsa-miR-140-3p	42.48525041	34.14858813	42.42421353	60.47295761	187.8396126	191.6279247	158.7702492	128.9449615	59.63704626	60.22583205	52.23820363	9.2112083
185	hsa-miR-139-5p	92.95004692	110.212308	91.44643479	110.9956022	432.1074809	415.8932343	387.3199279	351.8944119	44.01749357	40.05994772	31.49203321	1.307344149
184	hsa-miR-139-3p	12.51713881	16.50631546	25.35287099	50.47745679	57.16305467	42.36446483	42.15611949	16.12940237	54.57363356	49.87643664	29.19314224	24.51279779
183	hsa-miR-138-2*	22.33558573	41.8385933	41.97951912	68.54224173	16.41159826	32.61291411	31.71850106	3.836537181	45.15804407	32.72869926	32.39107614	24.79528432
182	hsa-miR-138-1*	19.40688723	21.05830046	24.3540879	44.63129013	42.05194728	36.34295063	45.1628371	16.8462872	37.74559847	29.96813606	33.22822073	8.398146948
181	hsa-miR-138	18.52124848	28.95972407	31.07547786	48.76464334	46.1562495	41.70121317	37.74287818	22.20841578	33.6144185	9.093831647	20.57090366	7.651783021
180	hsa-miR-137	11.49996092	20.99811658	30.34978885	47.74713626	26.67892022	30.52850388	31.30862842	0.045439437	25.74405378	20.98909895	33.7178822	0.016402026
179	hsa-miR-136*	3.820566681	19.40846988	20.91959376	54.11802577	17.95062875	16.82839975	42.18923626	0.044853347	52.14451742	41.89905887	52.13515596	8.812747455
178	hsa-miR-136	0.012320075	17.64282759	23.76514787	48.74480206	14.72525168	24.66968735	28.55318964	0.051071392	14.22806812	21.87991489	23.2128033	1.461056802
177	hsa-miR-135b*	19.06718631	25.63349464	34.07695476	52.18738759	28.44743239	24.51809716	41.31597606	6.406521687	54.95083357	41.05952947	57.48888947	25.54711079
176	hsa-miR-135b	0.011895336	14.67604408	27.99401376	46.47930499	16.48122142	27.99290211	30.63584362	7.53951188	16.64483407	19.12638242	35.78074143	0.635198114
175	hsa-miR-135a*	9.80380444	25.16476795	23.16457694	58.50457009	24.86263452	24.61261732	34.41342893	14.81013903	123.2070673	102.7082982	107.3838074	76.72685332
174	hsa-miR-135a	1.533171688	20.09549059	23.06473154	54.49949464	9.492547025	27.19998717	28.69741296	0.049149434	50.46337627	47.60236541	60.63999453	48.43761088
173	hsa-miR-134	14.14699269	38.64895777	38.15550462	64.32697845	75.54815744	69.19251545	81.82389165	51.88046725	82.74425839	71.188637	63.22942588	59.50538667
172	hsa-miR-133b	19.52259761	14.6787415	22.15563897	54.07531458	41.92884811	64.21429335	58.93873885	34.28101333	33.70168821	11.69131844	17.89697064	21.03401696
171	hsa-miR-133a	8.939877383	22.86573269	30.89639649	46.26502242	48.9424022	87.09373856	59.84481408	33.78031423	59.24392977	24.91345251	44.98070342	14.93892968
170	hsa-miR-1324	7.497226039	17.88770628	23.75463483	50.60148313	34.82191383	33.48911225	21.25402145	0.206838222	40.0102006	41.41964293	39.13352569	38.08941221
169	hsa-miR-1323	240.2249064	269.0283066	216.5978342	299.4355248	29.2311576	32.13941172	29.21547238	29.69615557	52.98119312	32.58543479	49.89147326	18.4420367
168	hsa-miR-1322	1.115071685	8.698671101	16.32866927	43.28753448	24.76840368	20.69411272	30.98794121	14.79821344	61.01127588	27.30546607	34.30254602	20.09409609
167	hsa-miR-1321	0.013488178	12.21890765	12.61520572	46.49661374	12.66886445	44.53840018	30.9726645	19.40414099	695.1885614	701.9996199	700.8977607	784.2796032
166	hsa-miR-132*	0.16570452	4.339314022	23.0203684	37.52013094	63.31203773	35.66355552	31.9806568	15.15388335	38.80807003	41.4681211	34.36369916	16.72999995
165	hsa-miR-132	175.974068	187.0860312	176.6729678	239.9270137	876.3160084	817.9502108	795.2362482	830.6455881	536.1497083	207.9415502	247.4950901	263.1147329
164	hsa-miR-130b*	5.325760613	18.57181442	15.41630546	49.44239153	59.21091788	47.56314487	30.40072805	12.26523586	31.13293461	42.52151346	40.86555496	29.26940791
163	hsa-miR-130b	197.1875181	158.1946132	161.9121753	245.6136641	694.0934891	246.0341888	258.8129227	285.2246882	77.0917728	69.25154646	55.17428778	14.58984293
162	hsa-miR-130a*	0.015571046	12.72429119	19.06078958	42.36114189	51.6285736	38.44593201	30.57232655	13.63122285	973.3009624	989.4440784	912.259569	999.2893219
161	hsa-miR-130a	60.25639162	58.31114091	84.85987305	84.60031159	85.96617946	66.62075597	61.61142588	37.62355261	165.1259434	160.514845	150.3220229	103.8154903
160	hsa-miR-1308	1460.549449	1421.806099	1638.152883	2280.657617	892.7170862	843.8620226	784.5352903	842.2415708	61.53838214	52.78185168	39.90768672	12.98545589
159	hsa-miR-1307	160.1308283	137.4855515	187.2194508	179.1357195	142.5728309	133.3790047	108.7194533	101.6742061	180.6269981	142.4719602	114.0439777	95.20438837
158	hsa-miR-1306	15.60500638	24.99240422	43.54394352	51.30168587	60.45759053	35.30239943	19.69338257	1.952006221	38.27768663	43.23604788	55.48273135	6.214107829
157	hsa-miR-1305	148.5948084	110.842184	179.9763901	187.1446792	106.9408439	87.62804785	84.86046665	57.11412741	58.60333137	52.61380329	26.84230382	9.882477067
156	hsa-miR-1304	6.910763416	18.50544951	55.98166961	44.32200724	42.67319449	29.63043946	5.317880397	2.353456974	37.31528467	26.66130208	11.96889392	0.015342962

155	hsa-miR-1303	9.868326957	21.13888105	47.89562421	49.46137526	55.21589699	40.04679508	40.07915506	19.98858699	73.06375284	55.58285953	32.33259855	27.67351338
154	hsa-miR-1302	7.059869255	14.65688502	35.14177446	41.55774615	28.60714829	26.95637497	19.25069486	0.047126376	73.45305128	62.95180105	43.63203948	29.13495391
153	hsa-miR-1301	13.72782549	17.60050239	51.92603971	41.34033746	53.55083222	43.64773911	22.34259853	6.058217248	69.3209175	48.26986988	36.26619278	14.09832976
152	hsa-miR-1300	53.01514803	52.35778502	78.81121164	84.32378454	56.84247371	40.50583857	44.37842824	25.63192266	36.78888342	31.76587827	14.14700773	11.48825928
151	hsa-miR-1299	9.21832652	17.30116863	39.74714098	43.29558576	39.74274202	48.28293174	14.58627927	11.22049783	43.28503254	46.4923288	9.000276244	8.603873109
150	hsa-miR-1298	3.942626274	9.231824614	38.97189066	34.99958926	36.05605426	32.0335803	5.703778835	1.737705596	57.17394269	57.60464317	21.12544524	19.86036284
149	hsa-miR-1297	0.011243108	7.741956073	40.68085919	38.04861821	36.67902833	24.35338468	8.928353314	0.040972143	57.20752384	32.59713772	12.28386179	14.36917861
148	hsa-miR-1296	14.4590763	8.808838131	44.9420297	36.57096703	35.4708386	46.61975228	17.22395007	4.352817531	66.87605568	49.73285116	6.549167072	24.03872821
147	hsa-miR-129-5p	33.66416657	44.05952126	69.83742634	94.25195123	46.34671025	34.36442312	31.49002365	11.08010344	36.38626535	30.45141848	12.2158929	12.99058637
146	hsa-miR-1295	7.548734277	14.65721425	38.05140419	41.49603658	45.44411285	36.58398112	13.53760032	1.343799088	48.98334942	38.64738634	29.80598344	26.33553906
145	hsa-miR-1294	4.274489773	7.329096239	37.89785408	39.754584	31.48370428	31.86620716	0.046811263	0.04455336	37.05251682	37.87098268	34.90987273	28.41150503
144	hsa-miR-129-3p	11.19173187	14.62396643	43.5267496	50.40811393	57.54342734	42.56883571	31.63742051	14.3241688	57.85392873	37.23133982	21.62748439	29.02862959
143	hsa-miR-1293	7.006084831	11.26400697	53.37737723	41.14926045	33.42597968	35.00506675	53.69129534	11.67789079	52.61241425	38.2692201	66.56827839	31.31050994
142	hsa-miR-1292	17.2277994	6.548762194	39.9470597	39.52142244	50.43989407	49.39950361	31.13202714	26.55243267	89.42980383	55.97505807	39.90256621	48.20473761
141	hsa-miR-1291	0.896530586	5.261780341	46.58515221	39.47364454	39.64625383	37.16256949	59.3279501	7.604534052	44.96939003	49.20480094	26.56502653	39.15404954
140	hsa-miR-1290	578.2699315	388.2418767	507.2110824	503.6981191	44.32749037	35.39199294	24.60034516	20.56344852	64.36543924	35.2623136	28.03009511	39.08397743
139	hsa-miR-129*	6.843054423	4.665138987	39.25944196	39.13044675	46.52145616	52.33060437	29.81955483	27.2070482	51.40995646	31.77970058	19.35590389	27.35212276
138	hsa-miR-1289	1468.280221	1474.746723	3058.329135	1796.485495	50.98629665	44.58569793	14.95402285	14.91894423	43.11539085	40.29918592	21.2189519	34.30046762
137	hsa-miR-1288	6.582830274	7.121950574	33.93656295	35.54242821	65.25969915	43.64467055	14.99933534	11.99508887	66.80320412	48.71383711	26.94917877	36.56322808
136	hsa-miR-1287	2.859235023	20.97147075	37.93886554	30.12496785	49.02608524	32.90152736	12.66953149	14.60368359	117.9792663	52.17206564	44.93053329	29.13355527
135	hsa-miR-1286	3.067609911	14.27258418	37.39079452	40.03296366	57.279367	35.73116921	11.80553776	6.237521053	59.47663068	29.24169541	12.35729458	24.19185669
134	hsa-miR-1285	7.174600355	17.82634927	33.91374578	39.87438398	147.7712365	38.60875171	28.08219525	21.80630283	77.52047194	30.0104765	30.91680569	19.81954862
133	hsa-miR-1284	182.6219908	301.9901477	153.3427811	166.3291423	124.7557694	50.73120561	17.54135821	21.31661463	32.54984191	5.357369582	15.33069145	0.015059996
132	hsa-miR-1283	0.015094046	19.1099561	41.97107485	44.79247817	120.1406111	27.55877413	13.85443201	0.043999167	980.6844986	945.9950773	952.4640186	1025.631018
131	hsa-miR-1282	509.2846823	997.8457823	809.4756985	597.2354904	31.68760148	28.97830074	0.049842423	0.047423961	1807.048233	1669.622945	1733.029219	1876.586487
130	hsa-miR-1281	74.05392297	92.17599917	97.75591104	88.92097535	304.7096896	290.5183722	251.8289165	390.6685517	381.7871841	605.9924192	380.7267825	331.2167919
129	hsa-miR-1280	288.9934998	314.8638199	346.8301446	344.2430411	1542.9974	1492.932113	1391.189366	1587.773986	31.83221737	23.44836152	21.19519499	13.38991343
128	hsa-miR-128	351.3112302	641.9845825	431.0555544	352.9971207	388.4067322	595.6570686	391.4639138	368.7424549	34.91952396	21.31985194	12.81338573	9.755818347
127	hsa-miR-1279	18.91598925	41.62888829	58.37063716	39.01937478	28.26280399	16.08356793	0.044840159	16.61016438	61.46111952	50.97056657	24.16113773	17.35385802
126	hsa-miR-1278	12.33671997	37.45748811	31.1753504	27.41453291	38.01019082	26.51831462	2.275950381	31.24786175	67.16562308	41.45438963	32.89655871	25.45963633
125	hsa-miR-1277	1820.749127	3185.395579	2358.795776	1372.467084	33.27542405	11.67930928	14.2864403	19.14612293	44.89429312	35.82489349	14.47853596	10.48280316
124	hsa-miR-1276	5.62290099	36.91164978	39.37011914	32.67686195	40.05913576	27.24461856	17.6577783	37.75125034	342.6998636	322.5282965	294.2257687	284.8863026
123	hsa-miR-127-5p	5.714247921	28.67019419	38.41729824	37.46846663	38.84405118	27.03784487	1.13529789	31.97549691	89.33342909	76.73497476	60.85463217	50.00913767
122	hsa-miR-1275	3667.91234	3836.978486	4135.533386	5488.919959	257.3899667	238.0575124	213.5744577	224.6126525	72.45996912	43.9658215	34.26276452	32.46903467
121	hsa-miR-1274b	22.79619265	37.13084323	45.42989862	42.56093634	69.31840176	52.06631402	43.52678273	52.70950221	53.15915621	18.7844428	11.61237705	11.16838932
120	hsa-miR-1274a	11.87244761	34.14006845	40.09840298	34.44666323	57.72079317	47.42558624	13.97183128	35.75602134	40.18141575	8.088604078	10.49718316	21.66601641
119	hsa-miR-127-3p	6.482929725	27.51999542	33.22038243	25.50994074	32.95376984	20.62104356	17.77989619	21.91256048	28.10988165	18.19736118	2.831947968	14.65891488
118	hsa-miR-1273	16.11690512	37.36513536	43.68548739	39.96540705	25.02466447	0.063520177	0.111100661	22.76460205	60.59160581	48.43188017	29.71967998	32.83028169
117	hsa-miR-1272	16.3252442	41.82065857	28.63491257	39.50998628	24.30524864	7.084213702	5.086698517	12.40977564	109.3473992	94.44653292	62.19434785	78.44205403
116	hsa-miR-1271	4.97043312	27.28611211	32.41152286	38.33181286	31.52859113	14.94061901	18.70131379	40.20695432	17.9911668	35.44392482	14.43002817	76.90505841
115	hsa-miR-1270	1.121961482	29.89349113	36.19459177	40.54092781	42.22688174	64.91661356	29.16173608	60.86904482	386.9186721	408.3505537	397.344583	385.5889179
114	hsa-miR-1269	11.24943073	22.14878108	34.30230399	28.13746359	26.04621738	28.713922	11.56014652	25.9979959	30.33037587	21.47320344	20.58786192	35.86450558
113	hsa-miR-1268	182.9476448	198.0609142	210.3591929	211.1398478	336.9379082	360.6044505	323.3144181	303.6752975	48.00757209	29.81621843	24.93097756	50.5990574
112	hsa-miR-1267	11.80375471	38.98912411	37.66597076	43.39427762	24.98621649	10.20380399	12.89464026	38.78909157	34.8697084	17.88369591	37.2540797	39.77546119
111	hsa-miR-1266	0.011551049	28.32922017	26.31026965	31.13057528	32.71942599	14.3009022	22.07772238	38.46134872	29.67994837	23.29073892	44.87269297	38.75582029
110	hsa-miR-1265	10.34698152	23.50722441	33.01385845	28.98335992	24.41233682	18.06990648	32.65288463	31.15971656	42.07115279	26.81704268	30.23838733	51.3684214
109	hsa-miR-1264	0.012720847	24.07625964	34.38302012	31.9585733	28.54695009	16.65006958	35.31010384	24.58155723	78.69310178	16.37988581	27.75283114	33.36200782

108	hsa-miR-1263	3.594491111	23.58034833	28.66547727	33.03148013	36.76993241	31.11836955	18.83444112	34.89754767	53.25668166	22.48043428	20.41546492	38.16534365
107	hsa-miR-1262	0.935744449	19.19243235	27.83244792	32.11951049	42.6178805	0.942498846	14.76643175	22.03687093	172.5822785	119.735558	114.8289079	91.69349196
105	hsa-miR-1260	18.4045101	36.9630128	37.57903344	30.70012238	158.4119558	114.2485045	124.535671	81.82233796	43.91039222	11.39689173	15.11192831	41.37017876
104	hsa-miR-126*	0.68500423	35.4534718	36.31507263	25.6345397	68.33797894	22.2723155	0.053444325	29.28745482	184.2904032	74.11346975	75.03401946	90.53104323
103	hsa-miR-126	37.10428228	56.62369413	47.22007343	52.7579476	275.8560704	81.04517971	78.35196567	94.11192645	92.15139775	35.01646062	44.35134284	51.09841154
102	hsa-miR-125b-2*	0.015169054	28.46935961	26.6117034	31.5789153	89.25987064	40.16892793	35.05296981	49.24245274	18.43797911	18.02423307	20.01622158	32.16456169
101	hsa-miR-125b-1*	0.011839546	20.11715287	37.75900692	29.60077856	8.918373276	28.01253882	0.366221431	17.55968716	665.6507043	761.2849396	726.5272481	634.3172129
100	hsa-miR-125b	359.7103744	450.0358264	436.751764	400.5390426	794.600583	892.2893865	794.6699255	718.7319271	1100.059566	1196.449666	1268.599222	1120.989764
99	hsa-miR-125a-5p	224.2541359	240.9123344	278.6617406	247.3321812	1064.181951	1122.917215	1099.70216	1047.098413	132.0495649	118.900525	134.4300589	97.25977912
98	hsa-miR-125a-3p	18.72360081	33.03705772	41.17857791	39.61287491	86.37609972	99.7649638	86.60031598	85.33879106	34.41202392	35.29067775	24.44745609	37.31022431
97	hsa-miR-1259	1212.564409	2659.499637	1616.044219	1343.159524	30.63332963	37.37456994	8.900017254	29.06175843	10.98032307	22.95277581	29.26423264	33.39927137
95	hsa-miR-1257	0.011580363	9.593107875	30.84331141	25.5634117	28.65003822	33.71236868	18.23011513	24.857317	38.15421958	35.36419113	31.68626058	22.99069723
93	hsa-miR-1255b	12.09982099	21.6893456	27.95384008	46.35516656	40.45051174	4.264378782	16.57391384	0.047550851	31.80854569	24.59035711	34.48713261	0.015445367
92	hsa-miR-1255a	9.604128258	24.23669218	28.32366931	40.37445811	63.64255315	8.720994231	35.97845733	2.076068357	25.56396603	12.28713702	29.10422141	1.101253157
91	hsa-miR-1254	13.68788984	23.06138821	25.81878348	46.86230275	44.18508978	20.66082215	43.08885121	7.047482566	40.36029094	42.86683433	46.2643497	12.24659212
90	hsa-miR-1253	16.97326297	30.34601241	20.47995749	50.75535377	37.71034437	24.06531807	38.63236527	0.042492672	12.93562817	28.3475645	21.07027424	4.692594714
89	hsa-miR-1252	0.011911138	14.93214117	17.74393363	57.50143809	24.28636158	13.10229894	21.25186761	0.047203423	21.39936179	14.1075083	22.06721961	0.015321054
88	hsa-miR-1251	6.21015752	21.01482071	17.44500632	46.98589269	20.076194	25.75853112	26.52371443	0.040749062	28.88180948	18.33902072	30.69650756	7.92035567
87	hsa-miR-1250	11.51934582	24.79004512	29.25047072	47.96074945	25.89755745	25.34516212	34.13792628	6.107896477	31.21706224	28.0984914	22.29592108	7.140317765
86	hsa-miR-1249	41.27912452	47.6747076	42.39541377	63.87666909	74.06788309	81.8717095	72.30200848	59.37203609	89.31063306	84.46272634	77.98823655	49.80077341
85	hsa-miR-1248	3.286058672	23.48629811	25.67656023	53.57758196	38.23077448	29.33399323	26.19678957	0.047709803	31.98345744	18.08960589	17.87973962	0.067613601
84	hsa-miR-1247	19.9480017	31.98653907	40.94152418	58.76919716	69.66741256	57.4625744	70.80321704	44.89096047	72.17626081	49.83590157	52.89693358	44.75450103
83	hsa-miR-1246	16089.0454	17137.14797	17446.57764	20728.22141	2647.288725	2021.415417	2331.089567	2541.003641	3345.009419	2799.330868	3123.345603	3426.728886
82	hsa-miR-1245	3.393990634	22.16012027	41.60453965	47.53139655	41.07855089	21.16903313	30.20558397	4.568853112	38.38159482	18.34163031	25.06135102	7.797972206
81	hsa-miR-1244	0.01250992	22.98932297	26.88444762	43.88387524	60.87557263	36.4040202	36.13133971	22.7765332	39.74518182	17.17971093	23.6415098	7.911578261
80	hsa-miR-1243	6.686056684	19.4541875	20.8609505	56.78978615	88.30980269	9.985628208	23.5594957	0.048859445	72.63899857	14.35423248	31.86553126	0.015777056
79	hsa-miR-124*	3.482402682	15.26232685	26.21358284	50.52230377	31.27433142	13.04215197	26.91972815	0.04397106	40.99849369	18.47966516	26.32364752	7.927681618
78	hsa-miR-124	4.351186266	18.23895498	23.29785578	47.55714936	38.81075545	8.748838004	26.76032177	6.888625284	46.62962703	22.77919707	36.89939234	8.659711032
77	hsa-miR-1238	36.22962186	35.1314232	38.19442199	59.38798699	81.44557945	54.59101045	62.76025726	43.89417405	93.15796005	93.78467004	89.68667971	69.47255486
76	hsa-miR-1237	14.32966633	37.61833196	36.13377565	57.37341173	83.98448263	69.94336877	66.54509862	51.7766344	107.2440558	97.48920842	96.79549014	70.63804912
75	hsa-miR-1236	3.023346303	21.88445647	21.80608408	50.07038663	49.6396768	23.61739219	36.84392386	23.90265874	35.91035561	24.45105757	37.50965951	28.23011987
74	hsa-miR-1234	23.06062711	38.46024003	31.6302578	64.55316039	244.8546422	252.3402629	260.4154614	229.7663726	233.6466243	212.9774741	213.4224286	217.8282241
73	hsa-miR-1233	11.90586414	21.48767191	22.99959184	45.2864162	44.90189574	35.69521971	34.9477967	24.20774215	50.26569527	37.89215016	51.97824475	43.32019624
72	hsa-miR-1231	86.19027525	78.34174405	70.29939483	107.8236944	43.42703697	29.45456856	31.75061677	22.98761191	56.80431585	29.56036868	54.05490647	51.57494992
71	hsa-miR-1229	9.123799588	26.6074388	26.49918931	54.49939647	90.75071629	67.42112837	74.48049939	49.27820269	88.10641413	70.02284217	69.97148335	65.05557114
70	hsa-miR-1228*	68.90446818	62.78550932	43.36835348	109.3387949	60.71465333	42.86554139	44.52880844	17.41179063	112.3956229	79.90839117	78.98703668	64.42171927
69	hsa-miR-1228	24.64489304	35.17024792	30.59972923	53.13257124	126.4213008	91.61505828	94.76977801	74.54095238	281.526828	228.5766374	239.3032291	223.3462067
68	hsa-miR-1227	11.73326655	24.35121449	26.07252247	46.5073132	155.9203105	44.9030425	47.31102818	39.88768598	136.4846767	51.85082635	66.88897726	44.21960407
67	hsa-miR-1226*	0.012559242	21.96723204	24.08041632	42.10492582	40.14958827	41.11799981	32.32736363	22.43957614	55.17967153	42.71366195	58.07231605	49.20438866
66	hsa-miR-1226	20.30419733	29.96885733	25.00969052	64.14402375	137.600091	41.80102645	45.57818657	26.57549401	85.28658635	34.93362328	47.7946146	39.09688975
65	hsa-miR-1225-5p	0.012409753	20.26951635	15.5860651	46.96106695	48.76357145	50.65255489	24.79987101	6.658447948	54.69280846	37.44565439	42.51624176	42.21069935
64	hsa-miR-1225-3p	2.160720821	26.07327474	32.61112945	54.17578756	84.01406201	56.19284719	47.39365703	33.42229095	70.88245526	65.39780417	69.3695774	53.42561703
63	hsa-miR-1224-5p	101.4881773	79.25782623	132.8941671	139.5581752	88.39823415	71.92122819	56.52393495	38.57944978	116.7498568	96.56426458	95.9386535	43.66301889
62	hsa-miR-1224-3p	18.38226395	26.38199345	43.39946253	43.72238345	57.11911655	54.53405073	32.71611562	27.97068571	61.68450962	48.9265756	45.47473554	21.92181814
61	hsa-miR-122*	8.364984935	18.34378566	41.65641122	41.7733084	33.32355842	23.66572423	11.85709832	0.048758286	25.92250263	25.70876742	11.6771629	0.015791351
60	hsa-miR-122	16.41196582	21.66986852	50.60552786	48.85652029	22.60094788	17.75552951	0.048984577	0.044662486	45.60498228	42.52331641	37.81206067	6.794225439
59	hsa-miR-1208	35.36570589	36.52349233	56.43088983	64.55356981	51.15896383	61.86059556	29.13698735	19.30355027	73.37409514	69.98256728	52.4478597	32.00319618

58	hsa-miR-1207-5p	50.89210096	41.20270386	86.46114175	81.71188888	50.53157967	58.92324279	33.04570527	23.33360768	78.76908799	67.19485458	73.54016442	31.71739836
57	hsa-miR-1207-3p	19.97945931	18.75003075	43.14974964	41.9330393	34.78353096	32.28312558	12.85366721	7.476818318	45.87138647	35.50272622	22.96704333	1.683440879
56	hsa-miR-1206	14.75029999	8.965661483	49.14810547	41.49688923	29.84604746	27.94136034	0.056793297	0.041608048	30.10990865	23.5175649	30.44671416	6.481077337
55	hsa-miR-1205	0.685509636	13.61693776	51.12737658	44.7121587	30.31589225	31.23389999	14.17457369	0.042660457	41.88831763	29.62150108	50.0454885	7.35015336
54	hsa-miR-1204	18.54543519	19.8660093	69.31009513	50.99189492	18.82162432	30.38217921	2.922042812	6.448099625	28.12145062	19.08477507	8.579595702	0.016679626
53	hsa-miR-1203	17.27703929	19.57082309	43.60522043	50.68305011	42.70069917	26.73349781	4.86122977	0.03949972	42.78368947	38.24142487	34.14627736	12.71836782
52	hsa-miR-1202	17.08396364	15.08931826	47.21995403	47.26411572	48.67881386	52.31426488	32.94509562	10.68644292	94.14916927	72.3829042	52.56511671	30.22992273
51	hsa-miR-1201	9.126979899	15.72665878	52.91609576	42.73882309	35.96160943	36.73114378	21.56008475	13.12234934	26.40154113	28.1377396	29.03185067	3.175051197
50	hsa-miR-1200	12.3514221	10.76075035	40.7069493	41.77088484	35.70199614	32.69574291	19.91813047	0.044006235	27.44628142	19.99509199	19.37422577	11.03978155
49	hsa-miR-1197	8.291257491	12.52744881	44.10401266	41.38590365	30.40931649	28.17804862	12.41501814	4.05248034	30.35031329	23.55340575	0.66665096	5.401405014
48	hsa-miR-1185	5.299320147	13.94152103	39.10146168	44.2958776	33.99850283	27.12706616	16.40208495	2.6915037	30.83865049	25.19748138	17.78744617	13.32324203
47	hsa-miR-1184	19.11620661	17.59055153	57.65762361	38.01177673	15.38568935	23.93557507	0.111092507	0.042865619	23.15406906	22.4397815	15.97597055	17.66287317
46	hsa-miR-1183	4.307165227	18.56502168	37.74053113	48.88440853	29.77466375	19.33982717	22.51276995	10.58223066	65.81545861	52.75557919	42.96445437	39.68268434
45	hsa-miR-1182	16.95731263	29.74075248	54.75901934	61.32686361	47.58019772	42.72779459	28.71133568	28.1410108	64.39141274	59.05670375	53.81882578	45.84002339
44	hsa-miR-1181	24.15850062	28.57109491	46.1951007	46.15192766	63.55912898	59.9355227	46.17511746	50.26000619	64.23361567	56.21438389	41.0114092	55.74589591
43	hsa-miR-1180	31.6415351	28.17849097	91.98456962	65.37973163	179.4510979	179.5909393	385.5051711	164.0335713	152.8826003	158.140537	317.6736179	167.0247086
42	hsa-miR-1179	0.011561272	11.00790336	48.33141939	40.34073007	28.83644154	21.76652033	28.81258146	8.6982404	33.89171601	14.66602222	49.22374472	26.40209175
41	hsa-miR-1178	11.87301089	11.40699924	40.86599488	36.88121978	37.58982937	24.17981692	30.11227277	18.27802194	19.3635874	20.90305758	20.85138981	27.90023804
40	hsa-miR-10b*	1.776807556	18.64837222	33.99686488	45.76760243	27.9751502	22.50238474	3.904994227	4.45941227	41.59891491	34.62881357	24.57315832	34.93957982
39	hsa-miR-10b	6.343281815	20.58969121	53.39055294	50.36606823	43.15654961	40.03700334	74.35671413	16.27926489	24.94921942	22.31324189	45.09564175	34.81524864
38	hsa-miR-10a*	0.813719807	21.82642359	57.08908489	47.92218805	69.97524276	21.87993844	14.53258822	1.827116795	69.92549541	38.80050928	21.95347998	17.44451812
37	hsa-miR-10a	40.38909788	40.35878031	63.12214172	74.28066358	198.0875102	71.19671599	69.12178741	73.02312103	138.4688349	70.34530565	57.0204078	60.52361165
36	hsa-miR-107	841.9493094	415.7926024	688.7737162	586.0750912	2101.883194	605.366095	786.5190089	644.6393559	1389.417904	445.6460423	631.5065756	534.9422522
35	hsa-miR-106b*	1.97958024	20.12315449	40.58189382	40.23844669	129.5748594	91.47866827	88.22748644	72.45072161	96.27130167	73.79995214	66.67132424	82.59732048
34	hsa-miR-106b	514.450962	294.7309061	494.9130859	363.3234319	2959.428461	898.6015689	1259.828348	877.8159296	2448.218729	790.1587832	1140.835793	827.2982334
33	hsa-miR-106a*	7.26904452	35.71120511	41.60828303	42.67593501	12.54543937	15.76887956	0.053595352	0.044526697	13.70530603	23.16905017	0.017926241	0.014619255
32	hsa-miR-106a	1733.966723	2355.074109	2132.345506	1917.309593	3137.789301	3455.174984	2869.902821	3393.517733	3361.423212	4054.854224	3428.810158	3737.674618
31	hsa-miR-105*	7.210444432	37.29914199	39.06775674	26.01950563	21.44267277	0.065517519	22.59534138	5.997288232	27.73435086	20.38557495	0.018038707	5.986891963
30	hsa-miR-105	15.55713651	43.78128302	39.25444247	35.48800968	47.35691053	58.05098149	43.3470336	48.77091545	58.75720583	59.15698154	39.80273105	28.93168267
29	hsa-miR-103-as	13.73937002	52.1015861	37.95075158	36.41489174	72.82872954	129.2421541	42.23059031	55.38741482	48.20410282	100.5543416	37.91314519	33.73220135
28	hsa-miR-103-2*	18.59296274	31.728601	42.61326644	43.52756509	42.79853233	47.23497215	16.80722211	36.81265484	33.1448378	38.59910908	11.73105943	12.22612509
27	hsa-miR-103	454.9241548	898.6689084	670.4430747	495.3755672	811.5093939	1345.981895	991.528641	776.6609065	651.0592349	1114.675578	796.2118657	511.1978021
26	hsa-miR-101*	16.14280286	39.21336481	44.3780009	29.89718248	23.48865965	48.01083955	1.545691937	25.24360273	36.16787015	52.86674982	5.610968308	13.00207353
25	hsa-miR-101	2.862248294	40.96437728	43.83010934	38.10445137	128.7629643	62.8852538	27.31354529	40.81806588	106.801864	105.1489961	25.27961399	10.41891454
24	hsa-miR-100*	8.959234892	37.08628449	45.10701203	31.55150882	23.15861949	59.10494887	0.112634742	16.35489003	26.97093605	58.23805222	5.09432195	9.234567879
23	hsa-miR-100	492.9501845	580.3145386	654.5888319	553.7726059	479.8832079	504.402393	510.625386	456.1786566	524.3068142	562.5342866	528.2797653	506.6560332
22	hsa-miR-1	3.814929277	24.48633585	25.60064277	31.69282248	64.16753824	75.78557309	92.63640521	90.51453168	235.459302	254.3119564	231.514457	199.995115
21	hsa-let-7i*	9.866870332	32.54251504	32.6445073	31.21495138	30.07237309	24.98273272	14.6286707	25.3549461	30.47495751	30.26972393	7.713722263	23.60372472
20	hsa-let-7i	4960.198818	5683.536023	6019.414415	6751.610872	3869.805525	3868.613791	3624.333639	4189.782637	3781.48167	4177.246724	3938.734103	4559.514721
19	hsa-let-7g*	13.26121832	25.59193785	34.70764717	30.08919367	43.63232943	23.97973772	22.9041367	24.10268529	48.31979614	38.12377843	24.07200501	38.34846731
18	hsa-let-7g	2480.822944	6958.426027	3376.660323	3009.624437	2034.329332	6556.061996	2123.736938	1960.46409	2180.442076	6385.15224	2442.674837	2184.820052
17	hsa-let-7f-2*	2.058999039	22.37014902	38.41271805	37.27418649	17.19395009	20.62808689	14.97046643	32.00375753	20.08086033	24.22191108	2.866910281	34.69331601
16	hsa-let-7f-1*	17.60699846	34.57751548	43.90862802	39.08718984	43.71940496	43.88630351	36.45954419	36.55783877	44.19542491	37.1764627	32.40256677	51.51322106
15	hsa-let-7f	4946.655635	6089.653263	6150.094581	6294.904251	4868.079049	4979.409524	4712.063189	5195.25468	5282.243018	5840.36405	5684.490549	6221.546936
14	hsa-let-7e*	8.994846264	20.05347763	23.33800214	35.81319771	37.45475182	41.28825878	20.90630298	34.94111181	39.26900971	47.04007428	21.02452068	39.64108885
13	hsa-let-7e	2834.663445	2965.027289	4151.384355	3132.261849	1537.821714	1443.737434	1957.207573	1396.083144	2037.143598	2007.687177	2591.934837	1921.354015
12	hsa-let-7d*	106.0430892	148.7940628	150.3848202	116.63974	344.0257817	333.3920577	345.1920363	303.1631721	304.7133118	349.5289222	345.036332	285.7582121

11	hsa-let-7d	4762.881758	5258.20365	5454.337114	6328.820351	3371.419252	3194.199215	3044.086943	3536.07714	3801.422186	3896.362553	3830.32881	4229.478977
10	hsa-let-7c*	9.303500883	19.54750852	22.74156259	29.88139204	39.87361358	42.08523886	26.98935768	31.6567584	27.74074922	26.163146	29.40670525	40.27199838
9	hsa-let-7c	5369.570679	5538.501534	5784.356518	7169.248802	3878.793696	3253.186453	3017.762716	4022.51392	5250.510896	4795.768282	4726.253953	5970.985438
8	hsa-let-7b*	54.68394631	55.47607017	57.02345145	55.19227188	169.9387531	127.0978151	121.0507104	101.9724547	109.6063175	104.877665	107.2940643	111.4396166
7	hsa-let-7b	4060.782394	4250.876262	4531.402645	5106.267403	2859.317712	2118.691779	2158.929895	2838.327618	3985.310473	3903.105139	3791.700099	4510.207876
6	hsa-let-7a-2*	0.01527069	22.01736219	28.55717145	30.45961359	89.8562297	49.59187602	26.82163342	40.80685322	52.51647832	34.21267811	30.06001013	46.15758874
5	hsa-let-7a*	0.245417565	17.80372382	44.37877343	30.08457025	43.55906802	31.43432538	0.053657454	13.15198577	46.99759968	10.31482082	6.179772075	17.05123332
4	hsa-let-7a	6221.541803	7439.243177	7795.387536	8727.004311	5303.407735	5428.009382	4823.744708	5928.163453	6173.405309	6913.277358	6563.21928	7816.911532
3	E3_001	35.9733636	26.23821519	23.48536177	0.012503957	33.6726762	9.731724303	8.616099887	14.72374358	24.01236105	9.345312337	2.443982014	0.132980165
2	E2_001	38.45593729	31.04559927	25.73863765	5.178659719	57.19903111	41.40287101	28.14852254	30.74931662	32.26878029	47.44964095	16.09168969	15.4192403
1	E1_17A	45.32256248	40.87297149	32.98457974	3.033925259	85.01745361	70.26676049	64.48769839	71.13169235	72.79907989	80.77317661	61.1923001	52.22572905

Supplementary Table 4:
Up and down regulated miRNAs

Differentially Regulated miRNAs				Enriched target diff. exp. genes in diff. exp. miRNA					
Up Reg miRNA		Down Reg miRNA		Enriched up & down miRNA		Up Reg. mRNA Enriched in		Down Reg. mRNA	
microRNA	FC	microRNA	FC	miRNA(up Reg.)	FC	mRNA(up)	FC	mRNA(Down)	FC
hsa-miR-7-5p	5.81	hsa-miR-548h-5p	-6.81	hsa-miR-16-5p	0.6	TMEM87A	0.60	ZNF362	-1.08
hsa-miR-1281	3.47	hsa-miR-1277-3p	-6.8	hsa-miR-27b-3p	0.61	CPEB2	0.62	SEMA6D	-2.17
hsa-miR-1825	3.4	hsa-miR-548m	-6.73	hsa-miR-24-3p	0.63	GFPT2	0.86	SLC4A4	-1.10
hsa-miR-143-3p	3.19	hsa-miR-498	-6.27	hsa-miR-30a-5p	0.73	CEP41	0.68	ARID5B	-0.99
hsa-miR-181b-5p	3.17	hsa-miR-1289	-5.96	hsa-miR-192-5p	0.77	SOCS6	0.65	ACTC1	-1.12
hsa-miR-1228-3p	2.76	hsa-miR-302f	-5.28	hsa-miR-17-5p	0.82	EPHB2	0.61	CEP120	-1.01
hsa-miR-1234-3p	2.65	hsa-miR-548f-3p	-5	hsa-miR-195-5p	0.83	PTPRK	0.59	PAK1	-0.77
hsa-miR-605-5p	2.51	hsa-miR-663a	-4.66	hsa-miR-106a-5p	0.84	DPF2	0.64	RRM2	-0.68
hsa-miR-145-5p	2.35	hsa-miR-146a-5p	-4.63	hsa-miR-155-5p	0.85	BCL2L1	0.84	SEMA4F	-0.63
hsa-miR-181d-5p	2.33	hsa-miR-186-3p	-4.63	hsa-miR-20a-5p	0.86	DDX19B	0.72	LGR4	-0.74
hsa-miR-18a-5p	2.29	hsa-miR-149-3p	-4.45	hsa-miR-221-3p	0.88	RYBP	0.72	WDR37	-0.93
hsa-miR-27b-5p	2.28	hsa-miR-1275	-4.2	hsa-miR-25-3p	0.91	RAD23B	0.60	COL1A1	-0.80
hsa-miR-125a-5p	2.24	hsa-miR-1290	-3.98	hsa-miR-125b-5p	0.96	BHLHE41	1.48	MAP4K4	-0.70
hsa-miR-18b-5p	2.21	hsa-miR-329-3p	-3.92	hsa-miR-30c-5p	0.99	HDAC9	0.93	SCD	-0.74
hsa-miR-197-3p	2.21	hsa-miR-548n	-3.89	hsa-miR-361-5p	0.99	LPHN3	0.60	TGDS	-0.98
hsa-miR-181a-2-3p	2.14	hsa-miR-575	-3.82	hsa-miR-30e-5p	1	CCND1	0.90	PLA2G3	-0.75
hsa-miR-132-3p	2.09	hsa-miR-483-5p	-3.75	hsa-miR-30b-5p	1.01	PAPPA	0.62	UBE2I	-0.69
hsa-miR-19b-3p	2.09	hsa-miR-96-5p	-3.74	hsa-miR-342-3p	1.08	RND3	1.06	GNPTAB	-0.78
hsa-miR-596	2.07	hsa-miR-513a-5p	-3.64	hsa-miR-222-3p	1.09	IRS2	1.43	EPHA7	-0.99
hsa-miR-1180-3p	2.06	hsa-miR-548l	-3.5	hsa-miR-15a-5p	1.1	MXD1	0.63	Sep-07	-0.81
hsa-miR-1260a	2.01	hsa-miR-638	-3.47	hsa-miR-183-5p	1.1	ACVR1	0.79	SCYL3	-1.16
hsa-miR-139-5p	1.97	hsa-miR-593-3p	-3.4	hsa-miR-182-5p	1.12	PAFAH1B1	0.66	SNTB2	-1.13
hsa-miR-99b-5p	1.97	hsa-miR-593-5p	-3.4	hsa-miR-181a-5p	1.26	FNDC3B	0.71	AGO1	-0.69
hsa-miR-324-5p	1.95	hsa-miR-519b-3p	-3.31	hsa-miR-93-5p	1.57	NFAT5	0.81	RDX	-1.51
hsa-miR-140-3p	1.89	hsa-miR-769-3p	-3.27	hsa-miR-148b-3p	1.78	NRP1	0.75	MAPK9	-0.61
hsa-miR-125a-3p	1.86	hsa-miR-1323	-3.09	hsa-miR-454-3p	1.82	GIGYF2	1.35	SMC1A	-0.67
hsa-miR-454-3p	1.82	hsa-miR-1246	-2.9	hsa-miR-139-5p	1.97	SPRY4	0.59	TAF9B	-0.66
hsa-miR-766-3p	1.81	hsa-miR-1469	-2.89	hsa-miR-19b-3p	2.09	SOX9	1.09	ELOVL5	-0.60
hsa-miR-940	1.8	hsa-miR-1915-3p	-2.71	hsa-miR-18b-5p	2.21	RGS2	0.61	USP48	-0.60
hsa-miR-148b-3p	1.78	hsa-miR-1284	-2.68	hsa-miR-18a-5p	2.29	OSBPL3	0.71	DNAJC13	-1.10
hsa-miR-484	1.74	hsa-miR-936	-2.59	miRNA(Dwn Reg.)	FC	ZFPM2	0.60	GALNT2	-0.91
hsa-miR-212-3p	1.71	hsa-miR-23b-5p	-2.27	hsa-let-7e-5p	-1.05	BCL2	0.86	RPS6KA5	-0.91
hsa-miR-30e-3p	1.58	hsa-miR-517b-3p	-2.24	hsa-let-7b-5p	-0.85	ABCA1	1.43	ICK	-0.83
hsa-miR-93-5p	1.57	hsa-miR-361-3p	-2.15	hsa-let-7c-5p	-0.75	ENAH	0.85	CALU	-0.65
hsa-miR-31-5p	1.5	hsa-miR-548c-5p	-2.04	hsa-let-7d-5p	-0.73	CAMK2D	0.60	USP46	-0.82
hsa-miR-151a-5p	1.49	hsa-miR-150-3p	-2.01	hsa-let-7i-5p	-0.59	NPAS2	0.88	PKD2	-0.61
hsa-miR-378a-3p	1.43	hsa-miR-671-5p	-1.82			ETV1	2.08	IKZF4	-0.78
hsa-miR-378a-5p	1.43	hsa-miR-1231	-1.42			NTN4	1.30	GTF2H1	-0.80
hsa-let-7d-3p	1.35	hsa-miR-874-3p	-1.31			F3	1.25	PTGFRN	-0.83
hsa-miR-181a-5p	1.26	hsa-miR-150-5p	-1.29			EFNB2	0.87	SSX2IP	-0.88
hsa-let-7b-3p	1.23	hsa-miR-1908-5p	-1.23			THBS1	1.27	DPYSL2	-0.73
hsa-miR-30a-3p	1.19	hsa-miR-572	-1.14			MCL1	0.80	RAB5B	-0.60
hsa-miR-28-3p	1.18	hsa-let-7e-5p	-1.05			SLC2A3	0.82	FAM81A	-0.96
hsa-miR-455-3p	1.18	hsa-miR-574-5p	-0.87					RECK	-0.85

hsa-miR-193b-5p	1.13	hsa-let-7b-5p	-0.85
hsa-miR-182-5p	1.12	hsa-let-7c-5p	-0.75
hsa-miR-15a-5p	1.1	hsa-let-7d-5p	-0.73
hsa-miR-183-5p	1.1	hsa-let-7i-5p	-0.59
hsa-miR-188-5p	1.09		
hsa-miR-222-3p	1.09		
hsa-miR-342-3p	1.08		
hsa-miR-1913	1.06		
hsa-miR-151a-3p	1.05		
hsa-miR-1238-3p	1.03		
hsa-miR-877-5p	1.03		
hsa-miR-24-2-5p	1.02		
hsa-miR-30b-5p	1.01		
hsa-miR-30e-5p	1		
hsa-miR-30c-5p	0.99		
hsa-miR-361-5p	0.99		
hsa-miR-1268a	0.98		
hsa-miR-125b-5p	0.96		
hsa-miR-320d	0.94		
hsa-miR-25-3p	0.91		
hsa-miR-221-3p	0.88		
hsa-miR-20a-5p	0.86		
hsa-miR-452-5p	0.86		
hsa-miR-155-5p	0.85		
hsa-miR-106a-5p	0.84		
hsa-miR-195-5p	0.83		
hsa-miR-17-5p	0.82		
hsa-miR-192-5p	0.77		
hsa-miR-30a-5p	0.73		
hsa-miR-99a-5p	0.7		
hsa-miR-24-3p	0.63		
hsa-miR-27b-3p	0.61		
hsa-miR-16-5p	0.6		

MINK1	-0.80
ITGA6	-0.82
EXOC5	-0.60
KAT2B	-0.94
TMEM47	-0.70
INSIG1	-2.18
DICER1	-0.70
DCP2	-1.24

Supplementary table 5 :

The pathways and GO biological processes(GOBP) data were compiled from the various resources as mentioned below. All these pathways and GOBP were combined and made a database in house at BioCOS Life Sciences to be used for the enrichment analysis.

Data sources used in creating Pathway/GOBP DB (Block "pathways/GOBP DB" - genes database for enrichment as shown in figure-1 A)

Data Source	Link	Reference Paper
Kegg Pathways	http://www.genome.jp/kegg-bin/get_htext?query=hsa00010&htext=hsa00001.keg	
Reactome Pathway	http://www.reactome.org/pages/download-data/	https://www.ncbi.nlm.nih.gov/pubmed/27589964
Wiki Pathways	www.wikipathways.org/index.php/Download_Pathways	https://academic.oup.com/nar/article-lookup/doi/10.1093/nar/gkv1024
NCI Nature Pathway	PathwayCommons.8.pid.GSEA.hgnc.gmt.gz (data)	https://academic.oup.com/nar/article/37/suppl_1/D674/1002223/PID-the-Pathway-Interaction-Database
Panther Pathways	ftp://ftp.pantherdb.org//pathway/current_release/SequenceAssociationPathway3.4.1.txt	https://www.ncbi.nlm.nih.gov/pubmed/26578592
Humancyc	https://humancyc.org/ (site) http://www.pathwaycommons.org/archives/PC2/current/ (data: pathway commons) File: PathwayCommons.8.humancyc.GSEA.hgnc.gmt.gz	http://genomebiology.biomedcentral.com/articles/10.1186/gb-2004-6-1-r2
Biological Process	http://www.geneontology.org/page/download-annotations goa_human.gaf.gz(file)	-

Supplementary table 6 :

The miRNA targets data were compiled from the various resources as mentioned below including the experimentally validated targets such as from Tarbase. All these miRNAs and corresponding target genes were combined and made a database in house at BioCOS Life Sciences to be used for the enrichment analysis. To reduce the false positives, only those target genes of a miRNA were taken - (1) Which are experimentally validated (2) which are at least reported in 5 databases/predictions and that is called as target genes with hit score 5.

Data sources used in creating miRNA target genes DB (Block "miRNAs Target Genes DB" - target genes database for enrichment as shown in figure-1 C2)

MiRNA TargetGenes Source	Link	Publication
miRTarbase	http://mirtarbase.mbc.nctu.edu.tw/	https://academic.oup.com/nar/article-lookup/doi/10.1093/nar/gkn809
miRecords	http://c1.accurascience.com/miRecords/	https://academic.oup.com/nar/article-lookup/doi/10.1093/nar/gkn851
**Prediction Algorithm sources**		
DIANA	http://diana.imis.athena-innovation.gr/DianaTools/index.php	https://www.ncbi.nlm.nih.gov/pmc/articles/PMC2703977/
miRanda	http://www.microrna.org/microrna/home.do	http://link.springer.com/protocol/10.1385%2F1-59745-123-1%3A101
targetscan	http://www.targetscan.org/vert_71/	https://elifesciences.org/content/4/e05005<br>http://www.cell.com/cell/pdf/S0092-8674(04)01260-7.pdf
picTar	http://www.pictar.org/	http://www.nature.com/ng/journal/v37/n5/full/ng1536.html
PITA	https://genie.weizmann.ac.il/pubs/mir07/mir07_prediction.html	http://www.nature.com/ng/journal/v39/n10/full/ng2135.html
rna22	https://cm.jefferson.edu/rna22/Interactive/	http://www.cell.com/cell/pdf/S0092-8674(06)01099-3.pdf
miRBridge		https://www.ncbi.nlm.nih.gov/pmc/articles/PMC3110938/

Supplementary Table 7.1:

Literature evidence of the target genes for the miRNA for Supplemetary Figure S2 A and S2 B

Literature evidence of the target genes for the miRNA for Supplementary figure S2 A and S2 B				
Literature evidence of the target genes for the miRNA for Supplementary figure S2 A				
**MiRNA**	**MRNA**	**Pubmed id**	**Refernce**	**Link**
Let-7	RRM2	PMID:23335963	PLoS One. 2013;8(1):e53436. Epub 2013 Jan 15.<br>Differential processing of let-7a precursors influences RRM2 expression and chemosensitivity in pancreatic cancer: role of LIN-28 and SET oncoprotein.<br>Bhutia YD1, Hung SW	https://www.ncbi.nlm.nih.gov/pubmed/23335963
	MAP4K4	PMID:27531260	2016 Jul;38(7):485-91.<br>[Let-7 miRNA silencing promotes Kaposi's sarcoma-associated herpesvirus lytic replication via activating mitogen-activated protein kinase kinase kinase kinase 4 and its downstream factors].<br>[Article in Chinese]<br>[illegible]	https://www.ncbi.nlm.nih.gov/pubmed/27531260
	PAK1,RDX	PMID:23339187	Mol Cancer Res. Epub 2013 Jan 21.<br>The heterochronic microRNA let-7 inhibits cell motility by regulating the genes in the actin cytoskeleton pathway in breast cancer.<br>Hu X, Guo J, Zheng L, Li C	https://www.ncbi.nlm.nih.gov/pubmed/23339187
MiR-106a	MAPK9	PMID:23029228	PLoS One<br>Published online 2012 Sep 24.<br>The miR-17 Family Links p63 Protein to MAPK Signaling to Promote the Onset of Human Keratinocyte Differentiation<br>Ning Wu, Eric Sulpice	https://www.ncbi.nlm.nih.gov/pmc/articles/PMC3454365/
MiR-125	DICER1	PMID:24513630	Oncogenesis. 2014 Feb 10<br>Serum levels of mature microRNAs in DICER1-mutated pleuropulmonary blastoma.<br>Murray MJ, Bailey S	http://www.nature.com/oncsis/journal/v3/n2/full/oncsis20141a.html

	IKZF4	PMID:22723551	Blood. 2012 Jun 21.<br>The down-regulation of miR-125b in chronic lymphocytic leukemias leads to metabolic adaptation of cells to a transformed state.<br>Tili E, Michaille JJ, Luo Z	http://www.bloodjournal.org/content/120/13/2631.long?sso-checked=true
MiR-15a	RECK	PMID:23176145	FEBS J. Epub 2013 Jan 16.<br>MicroRNA-15a promotes neuroblastoma migration by targeting reversion-inducing cysteine-rich protein with Kazal motifs (RECK) and regulating matrix metalloproteinase-9 expression.<br>Xin C, Buhe B, Hongting L	https://www.ncbi.nlm.nih.gov/pubmed/23176145
MiR-16	RECK	PMID:24852767	Cell Physiol Biochem. Epub 2014 May 5.<br>MiR-16 induced the suppression of cell apoptosis while promote proliferation in esophageal squamous cell carcinoma.<br>Zhu Y, Xia Y, Niu H	https://www.karger.com/Article/FullText/358701
	CALU	PMID:19549910	Cancer Res. Epub 2009 Jun 23.<br>miR-15a and miR-16 are implicated in cell cycle regulation in a Rb-dependent manner and are frequently deleted or down-regulated in non-small cell lung cancer.<br>Bandi N, Zbinden S	http://cancerres.aacrjournals.org/content/69/13/5553
	SLC4A4	PMID:25701361	Pathol Res Pract. Epub 2015 Jan 14.<br>Global correlation analysis for microRNA and gene expression profiles in human obesity.<br>Li J, Zhou C	https://www.ncbi.nlm.nih.gov/pubmed/25701361
MiR-17	PKD2	PMID:19821056<br>PMID:23759744<br>PMID:20215348	Mol Biol Rep. Epub 2009 Oct 10.<br>MicroRNA-17 post-transcriptionally regulates polycystic kidney disease-2 gene and promotes cell proliferation.<br>Sun H, Li QW, Lv XY<br><br>Proc Natl Acad Sci U S A. Epub [illegible]	https://www.ncbi.nlm.nih.gov/pubmed/19821056<br><br>http://www.pnas.org/content/110/26/10765.full<br><br>https://www.ncbi.nlm.nih.gov/pubmed/20215348

	IKZF4	PMID:24644282	J Biol Chem. Epub 2014 Mar 18. miR-17-92 cluster targets phosphatase and tensin homology and Ikaros Family Zinc Finger 4 to promote TH17-mediated inflammation. Liu SQ, Jiang S	https://www.ncbi.nlm.nih.gov/pubmed/24644282
	KAT2B	PMID:23095762	BMC Cancer. 2012 Oct 24; miR-17-5p targets the p300/CBP-associated factor and modulates androgen receptor transcriptional activity in cultured prostate cancer cells. Gong AY, Eischeid AN	https://www.ncbi.nlm.nih.gov/pmc/articles/PMC3519561/
	MAPK9	PMID:23029228	PLoS One. Epub 2012 Sep 24. The miR-17 family links p63 protein to MAPK signaling to promote the onset of human keratinocyte differentiation. Wu N, Sulpice E, Obeid P	https://www.ncbi.nlm.nih.gov/pmc/articles/PMC3454365/
MiR-181a	RECK	PMID:25230784	Acta Biochim Biophys Sin (Shanghai). Epub 2014 Sep 17. Clinical significance of up-regulated miR-181a in prognosis and progression of esophageal cancer. Xiang Z, Dong X, Sun Q	http://abbs.oxfordjournals.org/content/46/11/1007.full
MiR-182	RECK	PMID:26609496	Am J Cancer Res. 2015 Aug 15; MicroRNA miR-182 cluster mediated modulation of RECK without changes in cell surface membrane type-1 matrix metalloproteinase (MT1-MMP). Silva M, Hernandez ME	https://www.ncbi.nlm.nih.gov/pmc/articles/PMC4633917/
	AGO1	PMID:21195000	Mol Cell. Epub 2010 Dec 30. miR-182-mediated downregulation of BRCA1 impacts DNA repair and sensitivity to PARP inhibitors. Moskwa P, Buffa FM	https://www.ncbi.nlm.nih.gov/pmc/articles/PMC3249932/
	RDX	PMID:20186779	Am J Med Genet A. 2010 Mar; miRNA mutations are not a common cause of deafness. Hildebrand MS, Witmer PD	https://www.ncbi.nlm.nih.gov/pmc/articles/PMC2830307/

MiR-192	DICER1	PMID:24223844	PLoS One. 2013 Nov 1;<br>MiR-192 directly binds and regulates Dicer1 expression in neuroblastoma.<br>Feinberg-Gorenshtein G, Guedj A	http://journals.plos.org/plosone/article?id=10.1371/journal.pone.0078713
MiR-20a	MAPK9	PMID:23029228	PLoS One. Epub 2012 Sep 24.<br>The miR-17 family links p63 protein to MAPK signaling to promote the onset of human keratinocyte differentiation.<br>Wu N, Sulpice E, Obeid P	https://www.ncbi.nlm.nih.gov/pmc/articles/PMC3454365/
MiR-221	PAK1	PMID:25447917<br>PMID:23333386	Gene. Epub 2014 Nov 18.<br>The investigation of miR-221-3p and PAK1 gene expressions in breast cancer cell lines.<br>Ergun S, Tayeb TS<br><br>Biochem Biophys Res Commun. Epub 2013 Jan 16.<br>Increased expression of microRNA-221 inhibits PAK1 in endothelial progenitor cells and impairs its	http://www.sciencedirect.com/science/article/pii/S0378111914012980<br>https://www.ncbi.nlm.nih.gov/pubmed/23333386
MiR-222	PAK1	PMID:19962668	Cancer Cell. 2009.10.014.<br>miR-221&222 regulate TRAIL resistance and enhance tumorigenicity through PTEN and TIMP3 downregulation.<br>Garofalo M, Di Leva G	http://www.sciencedirect.com/science/article/pii/S1535610809003833
MiR-24	INSIG1	PMID:24677249	Hepatology. Epub 2014 May 19.<br>Inhibition of microRNA-24 expression in liver prevents hepatic lipid accumulation and hyperlipidemia.<br>Ng R, Wu H	https://www.ncbi.nlm.nih.gov/pubmed/24677249
MiR-25	KAT2B	PMID:27507706	Sci Rep. 2016 Aug 10;<br>miRNA profiles in livers with different mass deficits after partial hepatectomy and miR-106b~25 cluster accelerating hepatocyte proliferation in rats.<br>Xu X, Liu Z, Wang J	https://www.ncbi.nlm.nih.gov/pmc/articles/PMC4978973/

	INSIG1	PMID:24643999	Int J Cancer. Epub 2014 Apr 2.<br>Genetic association of gastric cancer with miRNA clusters including the cancer-related genes MIR29, MIR25, MIR93 and MIR106: results from the EPIC-EURGAST study.<br>Espinosa-Parrilla Y, Muñoz X	http://onlinelibrary.wiley.com/doi/10.1002/ijc.28850/full
MiR-27	SEMA6D	PMID:21536891	Proc Natl Acad Sci U S A. Epub 2011 May 2.<br>Regulation of angiogenesis and choroidal neovascularization by members of microRNA-23~27~24 clusters.<br>Zhou Q, Gallagher R<br><br>[illegible]	https://www.ncbi.nlm.nih.gov/pmc/articles/PMC3100947/<br>http://circ.ahajournals.org/content/132/Suppl_3/A15187?cited-by=yes&legid=circulationaha;132/Suppl_3/A15187
	USP46	PMID:25369332	PLoS One. 2014 Nov 4;<br>miR-27 negatively regulates pluripotency-associated genes in human embryonal carcinoma cells.<br>Fuchs H, Theuser M	http://journals.plos.org/plosone/article?id=10.1371/journal.pone.0111637
MiR-30a	SEPT7	PMID:23383034	PLoS One. Epub 2013 Jan 28.<br>MiR-30a-5p antisense oligonucleotide suppresses glioma cell growth by targeting SEPT7.<br>Jia Z, Wang K	https://www.ncbi.nlm.nih.gov/pmc/articles/PMC3557229/
MiR-92 family	PKD2	PMID:23759744	Proc Natl Acad Sci U S A. Epub 2013 Jun 12.<br>miR-17~92 miRNA cluster promotes kidney cyst growth in polycystic kidney disease.<br>Patel V, Williams D	http://www.pnas.org/content/110/26/10765.full
	IKZF4	PMID:24644282	J Biol Chem. Epub 2014 Mar 18.<br>miR-17-92 cluster targets phosphatase and tensin homology and Ikaros Family Zinc Finger 4 to promote TH17-mediated inflammation.<br>Liu SQ, Jiang S, Li C, Zhang B	https://www.ncbi.nlm.nih.gov/pubmed/24644282
**Literature evidence of the target genes for the miRNA in supplementary figure S2 B**				
**MiRNA**	**MRNA**	**Pubmed id**	**Refernce**	**Link**

MiR-106 family	NFAT5	PMID:24287119<br>PMCID:PMC4357785	Original Research<br>An Integrative Meta-analysis of MicroRNAs in Hepatocellular Carcinoma<br>Mahmoud ElHefnawi, et al	http://www.sciencedirect.com/science/article/pii/S1672022913001174
	F3	PMCID:<br>PMC3355544	Published online 2012 May 3<br>miR-93/106b and Their Host Gene, MCM7, Are Differentially Expressed in Leiomyomas and Functionally Target F3 and IL-8<br>Tsai-Der Chuang, et al	https://www.ncbi.nlm.nih.gov/pmc/articles/PMC3355544/
	ABCA1	PMID:22119192<br>PMCID:PMC3328628	Published online 2011 Nov;<br>miR-106b impairs cholesterol efflux and increases Aβ levels by repressing ABCA1 expression<br>Jaekwang Kim, et al	https://www.ncbi.nlm.nih.gov/pmc/articles/PMC3328628/
MiR-125a	RYBP	PMID:26759236	Microenvironment and Immunology<br>Forced Activation of Notch in Macrophages Represses Tumor Growth by Upregulating miR-125a and Disabling Tumor-Associated Macrophages<br>Jun-Long Zhao, et al	http://cancerres.aacrjournals.org/content/76/6/1403.long
MiR-125b	MCL1	PMCID:<br>PMC4609369	Biomed Res Int. 2015;<br>miR-125b Suppresses Proliferation and Invasion by Targeting MCL1 in Gastric Cancer<br>Shihua Wu, et al	https://www.ncbi.nlm.nih.gov/pmc/articles/PMC4609369/
	PAFAH1B1	PMID:26035737	J Neural Eng. 2015 Aug<br>microRNA regulatory mechanism by which PLLA aligned nanofibers influence PC12 cell differentiation.<br>Yu Y, et al	https://www.ncbi.nlm.nih.gov/pubmed/26035737
	PAFAH1B1	PMID:26035737	J Neural Eng. 2015 Aug<br>microRNA regulatory mechanism by which PLLA aligned nanofibers influence PC12 cell differentiation.<br>Yu Y, et al	https://www.ncbi.nlm.nih.gov/pubmed/26035737

MiR-148a	ACVR1	PMID:25271001	Regulatory MiR-148a-ACVR1/BMP circuit defines a cancer stem cell-like aggressive subtype of hepatocellular carcinoma<br>Lian Li, et al	http://onlinelibrary.wiley.com/doi/10.1002/hep.27543/full
	ABCA1	PMID:26437365	Nature Medicine<br>MicroRNA-148a regulates LDL receptor and ABCA1 expression to control circulating lipoprotein levels<br>Leigh Goedeke, et al	http://www.nature.com/nm/journal/v21/n11/abs/nm.3949.html
MiR-15	RAD23B	PMID:24084574	Identification of potential microRNA–target pairs associated with osteopetrosis by deep sequencing, iTRAQ proteomics and bioinformatics<br>Minglin Ou, et al	http://www.nature.com/ejhg/journal/v22/n5/full/ejhg2013221a.html
	CPEB	PMID:26829217<br>PMCID:<br>PMC4734764	Published online 2016 Feb 1<br>CPEB and miR-15/16 Co-Regulate Translation of Cyclin E1 mRNA during Xenopus Oocyte Maturation<br>Ania Wilczynska, et al	https://www.ncbi.nlm.nih.gov/pmc/articles/PMC4734764/
	BCL2	PMID:16166262	Proc Natl Acad Sci U S A. 2005 Sep 27<br>miR-15 and miR-16 induce apoptosis by targeting BCL2.<br>Cimmino A, et al	https://www.ncbi.nlm.nih.gov/pubmed/16166262
	CCND1	PMID:22922827	2012 Aug 24<br>miR-15a and miR-16-1 Downregulate CCND1 and Induce Apoptosis and Cell Cycle Arrest in Osteosarcoma<br>CK Cai et al	https://www.ncbi.nlm.nih.gov/labs/articles/22922827/
MiR-16	CPEB	PMID:26829217<br>PMCID:<br>PMC4734764	Published online 2016 Feb 1<br>CPEB and miR-15/16 Co-Regulate Translation of Cyclin E1 mRNA during Xenopus Oocyte Maturation<br>Ania Wilczynska, et al	https://www.ncbi.nlm.nih.gov/pmc/articles/PMC4734764/

	BCL2	PMID:16166262	Proc Natl Acad Sci U S A. 2005 Sep 27<br>miR-15 and miR-16 induce apoptosis by targeting BCL2.<br>Cimmino A, et al	https://www.ncbi.nlm.nih.gov/pubmed/16166262
	CCND1	PMID:22922827	2012 Aug 24<br>miR-15a and miR-16-1 Downregulate CCND1 and Induce Apoptosis and Cell Cycle Arrest in Osteosarcoma<br>CK Cai et al	https://www.ncbi.nlm.nih.gov/labs/articles/22922827/
	RAD23B	PMID:24084574	Identification of potential microRNA–target pairs associated with osteopetrosis by deep sequencing, iTRAQ proteomics and bioinformatics<br>Minglin Ou, et al	http://www.nature.com/ejhg/journal/v22/n5/full/ejhg2013221a.html
MiR-17	SOCS6	PMID:24801601	miR-17-5p promotes proliferation by targeting SOCS6 in gastric cancer cells<br>Qiong Wu, et al	http://www.sciencedirect.com/science/article/pii/S0014579314003226
	ETV1	PMID:26158900<br>PMID:23969726	Oncotarget. 2015 Aug 7<br>miR-17 regulates melanoma cell motility by inhibiting the translation of ETV1.<br>Cohen R, et al<br>Br J Cancer. 2013 Sep 17;<br>MiR-17-92 and miR-221/222 cluster members target KIT and [illegible]	https://www.ncbi.nlm.nih.gov/pubmed/26158900<br>https://www.ncbi.nlm.nih.gov/pmc/articles/PMC3776993/
	NPAS2	PMID:27440219	Sci Rep. 2016 Jul 21;<br>A novel role of microRNA 17-5p in the modulation of circadian rhythm.<br>Gao Q, et al	https://www.ncbi.nlm.nih.gov/pubmed/27440219
	RND3	PMID: 22132820	Biochem J. 2012 Mar 1;<br>Up-regulated miR-17 promotes cell proliferation, tumour growth and cell cycle progression by targeting the RND3 tumour suppressor gene in colorectal carcinoma.<br>Luo H, et al	https://www.ncbi.nlm.nih.gov/pubmed/22132820

	MCL1	PMID:26513648	Cell Microbiol. 2016 May<br>MicroRNA 17-5p regulates autophagy in Mycobacterium tuberculosis-infected macrophages by targeting Mcl-1 and STAT3.<br>Kumar R, et al	https://www.ncbi.nlm.nih.gov/pubmed/26513648
	EFNB2	PMID:22438230	J Clin Endocrinol Metab. 2012 Jun;<br>Preeclampsia Up-Regulates Angiogenesis-Associated MicroRNA (i.e., miR-17, -20a, and -20b) That Target Ephrin-B2 and EPHB4 in Human Placenta<br>Wen Wang, et al	https://www.ncbi.nlm.nih.gov/pmc/articles/PMC3387422/
	ABCA1	PMID:25785043	Int J Clin Exp Med. 2015 Jan;<br>Up-regulated miR-93 contributes to coronary atherosclerosis pathogenesis through targeting ABCA1.<br>He Y, et al	https://www.ncbi.nlm.nih.gov/pubmed/25785043
	THBS1	PMID:22383169	Genes Chromosomes Cancer. 2012 Jun;<br>The miR-17-92 Cluster and Its Target THBS1 Are Differentially Expressed in Angiosarcomas Dependent on MYC Amplification<br>Antoine Italiano, et al	https://www.ncbi.nlm.nih.gov/pmc/articles/PMC3360479/
	CCND1	PMID:21283765<br>PMID:23246221	PLoS One. 2011<br>MicroRNAs MiR-17, MiR-20a, and MiR-106b Act in Concert to Modulate E2F Activity on Cell Cycle Arrest during Neuronal Lineage Differentiation of USSC<br>Hans-Ingo Trompeter, et al<br>[illegible]	https://www.ncbi.nlm.nih.gov/pmc/articles/PMC3024412/<br>https://www.ncbi.nlm.nih.gov/pubmed/23246221
MiR-18a	THBS1		Nucleic Acid Ther. 2014 Apr;<br>Multiple microRNAs derived from chemically synthesized precursors regulate thrombospondin 1 expression.<br>Dogar AM, et al	https://www.ncbi.nlm.nih.gov/pubmed/24444023

MiR-19b	ABCA1	PMID:25084135	Atherosclerosis. 2014 Sep<br>MicroRNA-19b promotes macrophage cholesterol accumulation and aortic atherosclerosis by targeting ATP-binding cassette transporter A1.<br>Lv YC, et al	https://www.ncbi.nlm.nih.gov/pubmed/25084135
MiR-20a	CCND1	PMID:21283765<br>PMID:23246221	PLoS One. 2011<br>MicroRNAs MiR-17, MiR-20a, and MiR-106b Act in Concert to Modulate E2F Activity on Cell Cycle Arrest during Neuronal Lineage Differentiation of USSC<br>Hans-Ingo Trompeter, et al<br><br>[illegible]	https://www.ncbi.nlm.nih.gov/pmc/articles/PMC3024412/<br>https://www.ncbi.nlm.nih.gov/pubmed/23246221
	MCL1	PMID:23594563	J Exp Clin Cancer Res. 2013;<br>Decrease expression of microRNA-20a promotes cancer cell proliferation and predicts poor survival of hepatocellular carcinoma<br>Ming-Qi Fan, et al	https://www.ncbi.nlm.nih.gov/pmc/articles/PMC3699415
	NTN4 (UP)	PMID:24972566	September 2014<br>miR-20a contributes to endometriosis by regulating NTN4 expression<br>Min Zhao, et al	http://link.springer.com/article/10.1007/s11033-014-3452-7
MiR-25	PTPRK	PMID:27353001	Int J Mol Med. 2016 Aug;<br>PMCID: PMC4935456<br>Altered expression of mir-222 and mir-25 influences diverse gene expression changes in transformed normal and anaplastic thyroid cells, and impacts on MEK and TRAIL protein expression<br>Sinéad T. Aherne, et al	https://www.ncbi.nlm.nih.gov/pmc/articles/PMC4935456/
	PAFAH1B1	PMID:26035737	J Neural Eng. 2015 Aug<br>microRNA regulatory mechanism by which PLLA aligned nanofibers influence PC12 cell differentiation.<br>Yu Y, et al	https://www.ncbi.nlm.nih.gov/pubmed/26035737

MiR-27	RYBP	PMID:20815877	Cooperative and individualistic functions of the microRNAs in the miR-23a~27a~24-2 cluster and its implication in human diseases<br>Ravindresh Chhabra, et al	http://molecular-cancer.biomedcentral.com/articles/10.1186/1476-4598-9-232
MiR-27b	ABCA1	PMID:26520906	Atherosclerosis. 2015 Dec<br>miR-27b inhibits LDLR and ABCA1 expression but does not influence plasma and hepatic lipid levels in mice.<br>Goedeke L, et al	https://www.ncbi.nlm.nih.gov/pubmed/26520906
	PAPPA	PMID:24188450	Gynecol Endocrinol. 2014 Jan; Profiling of differentially expressed microRNAs in premature ovarian failure in an animal model.<br>Kuang H, et al	https://www.ncbi.nlm.nih.gov/pubmed/24188450
	EphB2	PMID:22207734	miR-27b controls venous specification and tip cell fate<br>Dauren Biyashev, et al	http://www.bloodjournal.org/content/119/11/2679?sso-checked=true
MiR-30 family	SOX9	PMID:27261459	J Biol Chem. 2016 Jul 29;<br>PMCID: PMC4965549<br>miR-30 Family Controls Proliferation and Differentiation of Intestinal Epithelial Cell Models by Directing a Broad Gene Expression Program That Includes SOX9 and the Ubiquitin Ligase Pathway*An external file that holds a picture, illustration, etc<br>Bailey C. E. Peck, et al	https://www.ncbi.nlm.nih.gov/pmc/articles/PMC4965549/

	PTPRK	PMID:17434290	Maturational arrest of thymocyte development is caused by a deletion in the receptor-like protein tyrosine phosphatase κ gene in LEC rats<br>Hiroyuki Kose, et al	http://www.sciencedirect.com/science/article/pii/S0888754307000560
	SOX9		Cell Prolif. 2016 Apr; MicroRNA-30a promotes extracellular matrix degradation in articular cartilage via downregulation of Sox9.<br>Chang T, et al	https://www.ncbi.nlm.nih.gov/pubmed/26969024
	BCL2	PMID:24178239<br>PMID:24293274	Mol Cell Biochem. (2014) NF-κB-mediated miR-30b regulation in cardiomyocytes cell death by targeting Bcl-2.<br>Wei C, et al<br><br>J Pathol. 2014 Mar; MicroRNA-30b functions as a tumour suppressor in human colorectal cancer by targeting KRAS, PIK3CD and BCL2.<br>Liao WT, et al	https://www.ncbi.nlm.nih.gov/pubmed/24178239<br>https://www.ncbi.nlm.nih.gov/pubmed/24293274
	CAMK2D	PMID:23360843	J Investig Med. 2013 Mar; MicroRNA-30b-5p is involved in the regulation of cardiac hypertrophy by targeting CaMKIIδ.<br>He J, et al	https://www.ncbi.nlm.nih.gov/pubmed/23360843
	NRP1	PMID:25791621	Brain Res. 2015 Jun 22; MicroRNA-30b promotes axon outgrowth of retinal ganglion cells by inhibiting Semaphorin3A expression.<br>Han F, et al	https://www.ncbi.nlm.nih.gov/pubmed/25791621
	LPHN3	PMID:27802793	Identification of crucial miRNAs and the targets<br>in renal cortex of hypertensive patients by<br>expression profiles<br>Guohua Wang, et al	http://www.tandfonline.com/doi/pdf/10.1080/0886022X.2016.1244083

MiR-92	THBS1	PMID:22383169	Genes Chromosomes Cancer. 2012 Jun;<br>The miR-17-92 Cluster and Its Target THBS1 Are Differentially Expressed in Angiosarcomas Dependent on MYC Amplification<br>Antoine Italiano, et al	https://www.ncbi.nlm.nih.gov/pmc/articles/PMC3360479/
MiR-93	ABCA1	PMID:25785043	Int J Clin Exp Med. 2015 Jan;<br>Up-regulated miR-93 contributes to coronary atherosclerosis pathogenesis through targeting ABCA1.<br>He Y, et al	https://www.ncbi.nlm.nih.gov/pubmed/25785043
MiR-96	HDAC9	PMID:27558292<br>PMID:23360843	The microRNA cluster miR-183/96/182 contributes to long-term memory in a protein phosphatase 1-dependent manner<br>Bisrat T. Woldemichael, et al<br>J Investig Med. 2013 Mar;<br>MicroRNA-30b-5p is involved in the regulation of cardiac hypertrophy by targeting CaMKIIδ.<br>He J, et al	http://www.nature.com/articles/ncomms12594<br><br>https://www.ncbi.nlm.nih.gov/pubmed/23360843/
MiR-181	IRS2		Exp Cell Res. 2016 Oct 15;<br>MicroRNA-181b suppresses TAG via target IRS2 and regulating multiple genes in the Hippo pathway.<br>Chen Z, et al	https://www.ncbi.nlm.nih.gov/pubmed/27616141
	BCL2	PMID:24335172	Cell Physiol Biochem. (2013)<br>The function role of miR-181a in chemosensitivity to adriamycin by targeting Bcl-2 in low-invasive breast cancer cells.<br>Zhu Y, et al	https://www.ncbi.nlm.nih.gov/pubmed/24335172

	SLC2A3	PMID:25733820	Hsa-microRNA-181a is a regulator of a number of cancer genes and a biomarker for endometrial carcinoma in patients: a bioinformatic and clinical study and the therapeutic implication<br>He S, et al	https://www.dovepress.com/hsa-microrna-181a-is-a-regulator-of-a-number-of-cancer-genes-and-a-bio-peer-reviewed-fulltext-article-DDDT
MiR-182	BCL2	PMID:22848417<br>PMID:23936432	Role of MicroRNA-182 in Posterior Uveal Melanoma: Regulation of Tumor Development through MITF, BCL2 and Cyclin D2<br>Dongsheng Yan, et al<br><br>PloS one. 2013; 8(8):e70442<br>Inhibition of proliferation and induction of autophagy by atorvastatin in PC3 prostate cancer cells correlate with downregulation of Bcl2 and upregulation of miR-182 and p21.<br>Peng X, Li W, Yuan L, Mehta RG, Kopelovich L and McCormick DL.	http://journals.plos.org/plosone/article?id=10.1371/journal.pone.0040967<br>https://www.ncbi.nlm.nih.gov/pubmed/23936432
	BHLHE41			

	HDAC9	PMID:27558292<br>PMID:23360843	The microRNA cluster miR-183/96/182 contributes to long-term memory in a protein phosphatase 1-dependent manner<br>Bisrat T. Woldemichael, et al<br>J Investig Med. 2013 Mar;<br>MicroRNA-30b-5p is involved in the regulation of cardiac hypertrophy by targeting CaMKIIδ.<br>He J, et al	http://www.nature.com/articles/ncomms12594<br><br>https://www.ncbi.nlm.nih.gov/pubmed/23360843/
MiR-183	SOCS6	PMID:26400524	Eur Rev Med Pharmacol Sci. 2015<br>MiR-21 and miR-183 can simultaneously target SOCS6 and modulate growth and invasion of hepatocellular carcinoma (HCC) cells.<br>Li ZB, et al	https://www.ncbi.nlm.nih.gov/pubmed/26400524
	ABCA1	PMID:26935154	Oncol Rep. 2016 May<br>MiR-183 functions as an oncogene by targeting ABCA1 in colon cancer.<br>Bi DP, et al	https://www.ncbi.nlm.nih.gov/pubmed/26935154
	HDAC9	PMID:27558292<br>PMID:23360843	The microRNA cluster miR-183/96/182 contributes to long-term memory in a protein phosphatase 1-dependent manner<br>Bisrat T. Woldemichael, et al<br>J Investig Med. 2013 Mar;<br>MicroRNA-30b-5p is involved in the regulation of cardiac hypertrophy by targeting CaMKIIδ.<br>He J, et al	http://www.nature.com/articles/ncomms12594<br><br>https://www.ncbi.nlm.nih.gov/pubmed/23360843/
MiR-194	THBS1		Nucleic Acid Ther. 2014 Apr;<br>Multiple microRNAs derived from chemically synthesized precursors regulate thrombospondin 1 expression.<br>Dogar AM, et al	https://www.ncbi.nlm.nih.gov/pubmed/24444023

MiR-195	CCND1	PMID:25823925	Oncotarget. 2015 Apr 20<br>PMCID: PMC4496189<br>MicroRNA profiling identifies MiR-195 suppresses osteosarcoma cell metastasis by targeting CCND1<br>Kang Han, et al	https://www.ncbi.nlm.nih.gov/pmc/articles/PMC4496189/
MiR let-7a	THBS1		Nucleic Acid Ther. 2014 Apr;<br>Multiple microRNAs derived from chemically synthesized precursors regulate thrombospondin 1 expression.<br>Dogar AM, et al	https://www.ncbi.nlm.nih.gov/pubmed/24444023
MiR-221	THBS1		Nucleic Acid Ther. 2014 Apr;<br>Multiple microRNAs derived from chemically synthesized precursors regulate thrombospondin 1 expression.<br>Dogar AM, et al	https://www.ncbi.nlm.nih.gov/pubmed/24444023
MiR-222-3p	ZFPM2	PMID:25165856	PloS One. 2014;<br>PMCID: PMC4148428<br>Characterization of Circulating MicroRNA Expression in Patients with a Ventricular Septal Defect<br>Dong Li, et al	https://www.ncbi.nlm.nih.gov/pmc/articles/PMC4148428/

Supplementary Table 7.2:

Comparison with Tarbase miRNA/Target genes for supplementary Figure S2A and S2B

Comparision with Tarbase miRNA/Target genes of Fig 6A data S2A

MiRNA Data Portal
(BIOCOS Life Sciences)

home miRNA Info miRNA Target miRNA Pathways

miRNAtoDisease miRNA GeneOntology

miRNA Target gene Information

Show 50 entries Search:

Export to xls Export to csv Export to txt

miRNA ID	Strong evidance	Weak Evidence	Evidence Type
hsa-let-7b-5p	AGO1	RDX, CEP120, GALNT2, PTGFRN, PAK1, SCD, DICER1, UBE2I, RRM2, SMC1A, TAF9B, CALU	[Immunohistochemistry, Immunoprecipitaion, Luciferase reporter assay, Northern blot, qRT-PCR, Western blot:**PUB:23426184**, CLASH:**PUB:23622248**] **AGO1** [Proteomics:**PUB:18668040**, PAR-CLIP:**PUB:21572407**, PAR-CLIP:**PUB:23446348**] **RDX** [PAR-CLIP:**PUB:23592263**] **CEP120** [Proteomics:**PUB:18668040**] **GALNT2** [CLASH:**PUB:23622248**] **PTGFRN** [CLASH:**PUB:23622248**] **PAK1** [Proteomics:**PUB:18668040**, CLASH:**PUB:23622248**] **SCD** [Immunoblot:**PUB:18812516**] **DICER1** [Proteomics:**PUB:18668040**] **UBE2I** [Microarray:**PUB:17699775**, Proteomics:**PUB:18668040**, PAR-CLIP:**PUB:21572407**] **RRM2** [PAR-CLIP:**PUB:20371350**, PAR-CLIP:**PUB:21572407**, PAR-CLIP:**PUB:23446348**, CLASH:**PUB:23622248**] **SMC1A** [pSILAC, Proteomics, Other:**PUB:18668040**] **TAF9B** [PAR-CLIP:**PUB:20371350**] **CALU**
hsa-let-7c-5p	DICER1, AGO1	RDX, CEP120, RRM2, SMC1A, CALU, RAB5B	[Western blot, Northern blot:**PUB:18700235**] **DICER1** [Immunohistochemistry, Immunoprecipitaion, Luciferase reporter assay, Northern blot, qRT-PCR, Western blot:**PUB:23426184**, CLASH:**PUB:23622248**] **AGO1** [PAR-CLIP:**PUB:21572407**, PAR-CLIP:**PUB:23446348**] **RDX** [PAR-CLIP:**PUB:23592263**] **CEP120** [PAR-CLIP:**PUB:21572407**] **RRM2** [PAR-CLIP:**PUB:20371350**, PAR-CLIP:**PUB:21572407**, PAR-CLIP:**PUB:23446348**] **SMC1A** [PAR-CLIP:**PUB:20371350**] **CALU** [CLASH:**PUB:23622248**] **RAB5B**
hsa-let-7d-5p	DICER1	RDX, CEP120, RRM2, SMC1A, CALU	[Luciferase reporter assay, Western blot, Northern blot:**PUB:18700235**] **DICER1** [PAR-CLIP:**PUB:21572407**, PAR-CLIP:**PUB:23446348**] **RDX** [Sequencing:**PUB:20371350**, PAR-CLIP:**PUB:23592263**] **CEP120**

miRNA ID	Strong evidance	Weak Evidence	Evidence Type
			[PAR-CLIP:**PUB:21572407**] **RRM2**<br>[PAR-CLIP:**PUB:20371350**, PAR-CLIP:**PUB:21572407**, PAR-CLIP:**PUB:23446348**] **SMC1A**<br>[PAR-CLIP:**PUB:20371350**] **CALU**
hsa-let-7e-5p	AGO1, SMC1A	RDX, CEP120, SCD, RRM2, CALU	[Immunohistochemistry, Immunoprecipitaion, Luciferase reporter assay, Northern blot, qRT-PCR, Western blot:**PUB:23426184**, CLASH:**PUB:23622248**] **AGO1**<br>[Luciferase reporter assay, Reporter assay, Other:**PUB:15131085**, PAR-CLIP:**PUB:20371350**, PAR-CLIP:**PUB:21572407**, PAR-CLIP:**PUB:23446348**] **SMC1A**<br>[PAR-CLIP:**PUB:21572407**, PAR-CLIP:**PUB:23446348**, CLASH:**PUB:23622248**] **RDX**<br>[PAR-CLIP:**PUB:23592263**] **CEP120**<br>[CLASH:**PUB:23622248**] **SCD**<br>[PAR-CLIP:**PUB:21572407**] **RRM2**<br>[PAR-CLIP:**PUB:20371350**] **CALU**
hsa-let-7i-5p		RDX, CEP120, RRM2, SMC1A, CALU	[PAR-CLIP:**PUB:21572407**, PAR-CLIP:**PUB:23446348**] **RDX**<br>[PAR-CLIP:**PUB:23592263**] **CEP120**<br>[PAR-CLIP:**PUB:21572407**] **RRM2**<br>[PAR-CLIP:**PUB:20371350**, PAR-CLIP:**PUB:21572407**, PAR-CLIP:**PUB:23446348**] **SMC1A**<br>[PAR-CLIP:**PUB:20371350**] **CALU**
hsa-miR-106a-5p	MAPK9	RPS6KA5, PTGFRN, MINK1, GNPTAB, SCD, DPYSL2, AGO1, RRM2, RAB5B, USP48	[Immunoblot, Luciferase reporter assay, qRT-PCR:**PUB:23029228**] **MAPK9**<br>[PAR-CLIP:**PUB:21572407**, PAR-CLIP:**PUB:22012620**] **RPS6KA5**<br>[PAR-CLIP:**PUB:23592263**] **PTGFRN**<br>[HITS-CLIP:**PUB:23313552**] **MINK1**<br>[PAR-CLIP:**PUB:21572407**] **GNPTAB**<br>[PAR-CLIP:**PUB:21572407**] **SCD**<br>[PAR-CLIP:**PUB:22012620**] **DPYSL2**<br>[CLASH:**PUB:23622248**] **AGO1**<br>[PAR-CLIP:**PUB:23592263**] **RRM2**<br>[PAR-CLIP:**PUB:20371350**, PAR-CLIP:**PUB:21572407**, PAR-CLIP:**PUB:23446348**, PAR-CLIP:**PUB:23592263**] **RAB5B**<br>[PAR-CLIP, HITS-CLIP:**PUB:21572407**] **USP48**
hsa-miR-125b-5p	IKZF4	EPHA7	[Luciferase reporter assay, Microarray, qRT-PCR, Western blot:**PUB:22723551**] **IKZF4**<br>[CLASH:**PUB:23622248**] **EPHA7**
hsa-miR-139-5p		PTGFRN	[HITS-CLIP:**PUB:23313552**] **PTGFRN**
hsa-miR-148b-3p		DCP2, FAM81A, TMEM47, DICER1, AGO1, USP48	[Microarray:**PUB:17612493**] **DCP2**<br>[Microarray:**PUB:17612493**] **FAM81A**<br>[Microarray:**PUB:17612493**] **TMEM47**<br>[PAR-CLIP:**PUB:21572407**, PAR-CLIP:**PUB:23592263**] **DICER1**<br>[Microarray:**PUB:17612493**] **AGO1**<br>[Microarray:**PUB:17612493**] **USP48**

miRNA ID	Strong evidance	Weak Evidence	Evidence Type
hsa-miR-155-5p	SSX2IP	SNTB2, SCD, RRM2, CALU	[Reporter assay, Other:**PUB:20584899**] **SSX2IP** [Proteomics:**PUB:18668040**] **SNTB2** [Proteomics:**PUB:18668040**] **SCD** [Proteomics:**PUB:18668040**] **RRM2** [Proteomics:**PUB:18668040**, HITS-CLIP:**PUB:22473208**] **CALU**
hsa-miR-15a-5p	RECK	SNTB2, GTF2H1, MINK1, DICER1, CALU, USP48	[GFP reporter assay, qRT-PCR:**PUB:23176145**, PAR-CLIP:**PUB:21572407**] **RECK** [PAR-CLIP:**PUB:21572407**, PAR-CLIP:**PUB:22012620**] **SNTB2** [Microarray:**PUB:18362358**] **GTF2H1** [HITS-CLIP:**PUB:22473208**] **MINK1** [HITS-CLIP:**PUB:22473208**] **DICER1** [HITS-CLIP:**PUB:22473208**, PAR-CLIP:**PUB:23592263**] **CALU** [PAR-CLIP:**PUB:20371350**, PAR-CLIP:**PUB:21572407**] **USP48**
hsa-miR-16-5p	RECK	SNTB2, GTF2H1, MINK1, IKZF4, DICER1, CALU, USP48, ELOVL5	[Luciferase reporter assay, qRT-PCR, Western blot:**PUB:24852767**, Sequencing:**PUB:20371350**, PAR-CLIP:**PUB:21572407**] **RECK** [PAR-CLIP:**PUB:21572407**, PAR-CLIP:**PUB:22012620**] **SNTB2** [Microarray:**PUB:18362358**] **GTF2H1** [HITS-CLIP:**PUB:22473208**] **MINK1** [CLASH:**PUB:23622248**] **IKZF4** [HITS-CLIP:**PUB:22473208**, CLASH:**PUB:23622248**] **DICER1** [Sequencing:**PUB:20371350**, HITS-CLIP:**PUB:22473208**, PAR-CLIP:**PUB:23592263**] **CALU** [PAR-CLIP:**PUB:20371350**, PAR-CLIP:**PUB:21572407**] **USP48** [Proteomics:**PUB:18668040**] **ELOVL5**
hsa-miR-17-5p	KAT2B, MAPK9, PKD2	SNTB2, WDR37, RPS6KA5, SSX2IP, PTGFRN, MINK1, GNPTAB, SCD, DPYSL2, AGO1, RRM2, TAF9B, RAB5B, USP48	[Immunocytochemistry, Luciferase reporter assay, qRT-PCR, Western blot:**PUB:23095762**] **KAT2B** [qRT-PCR, Luciferase reporter assay, Western blot:**PUB:18700987**, Immunoblot, qRT-PCR:**PUB:23029228**, Luciferase reporter assay:**PUB:21283765**] **MAPK9** [Luciferase reporter assay, qRT-PCR, Western blot:**PUB:19821056**] **PKD2** [HITS-CLIP:**PUB:22473208**] **SNTB2** [HITS-CLIP:**PUB:22473208**] **WDR37** [PAR-CLIP:**PUB:21572407**, PAR-CLIP:**PUB:22012620**] **RPS6KA5** [HITS-CLIP:**PUB:22473208**] **SSX2IP** [PAR-CLIP:**PUB:23592263**] **PTGFRN** [HITS-CLIP:**PUB:22473208**, HITS-CLIP:**PUB:23313552**] **MINK1** [PAR-CLIP:**PUB:21572407**] **GNPTAB** [PAR-CLIP:**PUB:21572407**] **SCD** [PAR-CLIP:**PUB:22012620**, CLASH:**PUB:23622248**] **DPYSL2** [HITS-CLIP:**PUB:22473208**, CLASH:**PUB:23622248**] **AGO1** [HITS-CLIP:**PUB:22473208**, PAR-CLIP:**PUB:23592263**] **RRM2** [CLASH:**PUB:23622248**] **TAF9B** [PAR-CLIP:**PUB:20371350**, PAR-CLIP:**PUB:21572407**, HITS-CLIP:**PUB:22473208**, PAR-CLIP:**PUB:23446348**,

miRNA ID	Strong evidance	Weak Evidence	Evidence Type
			PAR-CLIP:**PUB:23592263**, CLASH:**PUB:23622248**] **RAB5B** [PAR-CLIP, HITS-CLIP:**PUB:21572407**] **USP48**
hsa-miR-181a-5p	KAT2B	SSX2IP, SCD, MAP4K4	[Western blot, Luciferase reporter assay:**PUB:18728182**] **KAT2B** [HITS-CLIP:**PUB:22473208**] **SSX2IP** [Sequencing:**PUB:20371350**, PAR-CLIP:**PUB:21572407**, PAR-CLIP:**PUB:23446348**] **SCD** [Microarray:**PUB:22942087**] **MAP4K4**
hsa-miR-182-5p	RECK		[Luciferase reporter assay:**PUB:23226455**, Luciferase reporter assay, qRT-PCR, Western blot:**PUB:23333633**, Luciferase reporter assay:**PUB:23383207**, PAR-CLIP:**PUB:21572407**] **RECK**
hsa-miR-183-5p		INSIG1, RAB5B	[CLASH:**PUB:23622248**] **INSIG1** [CLASH:**PUB:23622248**] **RAB5B**
hsa-miR-18a-5p	DICER1	RPS6KA5	[Immunohistochemistry, In situ hybridization, Luciferase reporter assay, Microarray, qRT-PCR, Western blot:**PUB:23097559**, Sequencing:**PUB:20371350**] **DICER1** [PAR-CLIP:**PUB:21572407**] **RPS6KA5**
hsa-miR-18b-5p		RPS6KA5	[PAR-CLIP:**PUB:21572407**] **RPS6KA5**
hsa-miR-192-5p	DICER1	INSIG1, KAT2B, ICK, SCD, TAF9B, MAPK9, ELOVL5	[Luciferase reporter assay, qRT-PCR, Western blot:**PUB:24223844**, Microarray:**PUB:19074876**] **DICER1** [Microarray:**PUB:19074876**] **INSIG1** [Microarray:**PUB:19074876**] **KAT2B** [Microarray:**PUB:19074876**, PAR-CLIP:**PUB:22012620**] **ICK** [Microarray:**PUB:19074876**] **SCD** [Microarray:**PUB:19074876**] **TAF9B** [Microarray:**PUB:19074876**] **MAPK9** [Microarray:**PUB:19074876**] **ELOVL5**
hsa-miR-195-5p	DICER1	SNTB2, RECK, MINK1, AGO1, CALU, USP48	[qRT-PCR, Western blot:**PUB:23671264**, HITS-CLIP:**PUB:22473208**] **DICER1** [PAR-CLIP:**PUB:21572407**, PAR-CLIP:**PUB:22012620**] **SNTB2** [PAR-CLIP:**PUB:21572407**] **RECK** [HITS-CLIP:**PUB:22473208**] **MINK1** [CLASH:**PUB:23622248**] **AGO1** [HITS-CLIP:**PUB:22473208**, PAR-CLIP:**PUB:23592263**] **CALU** [PAR-CLIP:**PUB:20371350**, PAR-CLIP:**PUB:21572407**] **USP48**
hsa-miR-19b-3p	KAT2B	DCP2, GNPTAB, SCD, DICER1, AGO1, RAB5B, ELOVL5	[Western blot, Luciferase reporter assay:**PUB:18728182**] **KAT2B** [HITS-CLIP:**PUB:22473208**] **DCP2** [PAR-CLIP:**PUB:21572407**] **GNPTAB** [HITS-CLIP:**PUB:22473208**] **SCD** [PAR-CLIP:**PUB:21572407**, PAR-CLIP:**PUB:23592263**] **DICER1** [Sequencing:**PUB:20371350**, HITS-CLIP:**PUB:22473208**] **AGO1** [Sequencing, PAR-CLIP:**PUB:20371350**, PAR-CLIP:**PUB:21572407**,

miRNA ID	Strong evidance	Weak Evidence	Evidence Type
			HITS-CLIP:**PUB:22473208**, PAR-CLIP:**PUB:23446348**, PAR-CLIP:**PUB:23592263**] **RAB5B** [PAR-CLIP:**PUB:20371350**, PAR-CLIP:**PUB:21572407**, HITS-CLIP:**PUB:22473208**, PAR-CLIP:**PUB:23446348**, CLASH:**PUB:23622248**] **ELOVL5**
hsa-miR-20a-5p	MAPK9	INSIG1, SNTB2, CEP120, WDR37, RPS6KA5, SSX2IP, PTGFRN, MINK1, GNPTAB, SCD, DPYSL2, AGO1, RRM2, RAB5B, USP48	[Luciferase reporter assay:**PUB:21283765**] **MAPK9** [CLASH:**PUB:23622248**] **INSIG1** [HITS-CLIP:**PUB:22473208**] **SNTB2** [CLASH:**PUB:23622248**] **CEP120** [HITS-CLIP:**PUB:22473208**] **WDR37** [PAR-CLIP:**PUB:21572407**, PAR-CLIP:**PUB:22012620**] **RPS6KA5** [HITS-CLIP:**PUB:22473208**] **SSX2IP** [PAR-CLIP:**PUB:23592263**] **PTGFRN** [HITS-CLIP:**PUB:22473208**, HITS-CLIP:**PUB:23313552**] **MINK1** [PAR-CLIP:**PUB:21572407**] **GNPTAB** [PAR-CLIP:**PUB:21572407**] **SCD** [PAR-CLIP:**PUB:22012620**] **DPYSL2** [HITS-CLIP:**PUB:22473208**, CLASH:**PUB:23622248**] **AGO1** [HITS-CLIP:**PUB:22473208**, PAR-CLIP:**PUB:23592263**] **RRM2** [PAR-CLIP:**PUB:20371350**, PAR-CLIP:**PUB:21572407**, HITS-CLIP:**PUB:22473208**, PAR-CLIP:**PUB:23446348**, PAR-CLIP:**PUB:23592263**] **RAB5B** [PAR-CLIP, HITS-CLIP:**PUB:21572407**] **USP48**
hsa-miR-221-3p	SSX2IP, RECK, PAK1, DICER1		[Luciferase reporter assay, qRT-PCR:**PUB:23776679**] **SSX2IP** [Luciferase reporter assay, qRT-PCR, Western blot:**PUB:24269686**] **RECK** [Luciferase reporter assay:**PUB:23333386**] **PAK1** [Luciferase reporter assay:**PUB:21761362**] **DICER1**
hsa-miR-222-3p	SSX2IP, RECK, DICER1	MINK1	[Luciferase reporter assay, qRT-PCR:**PUB:23776679**] **SSX2IP** [Luciferase reporter assay:**PUB:22321642**] **RECK** [Luciferase reporter assay:**PUB:21761362**] **DICER1** [CLASH:**PUB:23622248**] **MINK1**
hsa-miR-24-3p	INSIG1	DCP2, RPS6KA5, PTGFRN, RRM2	[Immunofluorescence, qRT-PCR, Western blot:**PUB:24677249**, PAR-CLIP:**PUB:20371350**, PAR-CLIP:**PUB:23592263**, PAR-CLIP:**PUB:24398324**] **INSIG1** [Microarray:**PUB:19748357**] **DCP2** [PAR-CLIP:**PUB:22100165**] **RPS6KA5** [Sequencing:**PUB:20371350**] **PTGFRN** [qRT-PCR, Microarray:**PUB:19748357**] **RRM2**
hsa-miR-25-3p	KAT2B, RECK	INSIG1, SNTB2, AGO1, EXOC5	[Western blot, Luciferase reporter assay:**PUB:18728182**] **KAT2B** [Luciferase reporter assay, qRT-PCR, Western blot:**PUB:24078004**] **RECK** [PAR-CLIP:**PUB:23592263**] **INSIG1**

miRNA ID	Strong evidance	Weak Evidence	Evidence Type
			[CLASH:**PUB:23622248**] **SNTB2**<br>[CLASH:**PUB:23622248**] **AGO1**<br>[PAR-CLIP:**PUB:21572407**] **EXOC5**
hsa-miR-27b-3p		ICK, USP46, MAPK9	[PAR-CLIP:**PUB:23592263**] **ICK**<br>[PAR-CLIP:**PUB:23592263**] **USP46**<br>[CLASH:**PUB:23622248**] **MAPK9**
hsa-miR-30a-5p	SEPT7	PTGFRN, ITGA6, GTF2H1, SCD, RRM2, ELOVL5	[Luciferase reporter assay:**PUB:23383034**] **SEPT7**<br>[pSILAC, Proteomics, Other:**PUB:18668040**] **PTGFRN**<br>[Proteomics:**PUB:18668040**] **ITGA6**<br>[Proteomics:**PUB:18668040**] **GTF2H1**<br>[Proteomics:**PUB:18668040**] **SCD**<br>[Proteomics:**PUB:18668040**, HITS-CLIP:**PUB:22473208**, PAR-CLIP:**PUB:23592263**] **RRM2**<br>[Proteomics:**PUB:18668040**, PAR-CLIP:**PUB:21572407**] **ELOVL5**
hsa-miR-30b-5p		SNTB2, WDR37, IKZF4, RRM2, ELOVL5	[Sequencing:**PUB:20371350**] **SNTB2**<br>[CLASH:**PUB:23622248**] **WDR37**<br>[CLASH:**PUB:23622248**] **IKZF4**<br>[HITS-CLIP:**PUB:22473208**, PAR-CLIP:**PUB:23592263**] **RRM2**<br>[PAR-CLIP:**PUB:21572407**] **ELOVL5**
hsa-miR-30c-5p	UBE2I	IKZF4, DPYSL2, AGO1, RRM2, ELOVL5	[Luciferase reporter assay, qRT-PCR, Western blot:**PUB:19223510**] **UBE2I**<br>[CLASH:**PUB:23622248**] **IKZF4**<br>[CLASH:**PUB:23622248**] **DPYSL2**<br>[CLASH:**PUB:23622248**] **AGO1**<br>[HITS-CLIP:**PUB:22473208**, PAR-CLIP:**PUB:23592263**, CLASH:**PUB:23622248**] **RRM2**<br>[PAR-CLIP:**PUB:21572407**] **ELOVL5**
hsa-miR-30e-5p	UBE2I	RRM2, ELOVL5	[Luciferase reporter assay, qRT-PCR, Western blot:**PUB:19223510**] **UBE2I**<br>[HITS-CLIP:**PUB:22473208**, PAR-CLIP:**PUB:23592263**] **RRM2**<br>[PAR-CLIP:**PUB:21572407**] **ELOVL5**
hsa-miR-342-3p		INSIG1, RRM2	[PAR-CLIP:**PUB:20371350**, PAR-CLIP:**PUB:21572407**] **INSIG1**<br>[CLASH:**PUB:23622248**] **RRM2**
hsa-miR-361-5p			
hsa-miR-454-3p		SNTB2, RPS6KA5, GNPTAB, DPYSL2, DICER1, RAB5B	[HITS-CLIP:**PUB:23824327**] **SNTB2**<br>[PAR-CLIP:**PUB:21572407**] **RPS6KA5**<br>[PAR-CLIP:**PUB:21572407**] **GNPTAB**<br>[PAR-CLIP:**PUB:22012620**] **DPYSL2**<br>[HITS-CLIP:**PUB:19536157**, PAR-CLIP:**PUB:21572407**, PAR-CLIP:**PUB:23592263**, HITS-CLIP:**PUB:23824327**] **DICER1**<br>[PAR-CLIP:**PUB:20371350**, PAR-CLIP:**PUB:21572407**, PAR-CLIP:**PUB:23446348**, PAR-CLIP:**PUB:23592263**] **RAB5B**
hsa-miR-93-5p	KAT2B, MAPK9	SNTB2, EPHA7, WDR37,	[Western blot, Luciferase reporter assay:**PUB:18728182**] **KAT2B**<br>[Luciferase reporter

miRNA ID	Strong evidance	Weak Evidence	Evidence Type
		RPS6KA5, SSX2IP, PTGFRN, MINK1, IKZF4, GNPTAB, SCD, DPYSL2, AGO1, RRM2, PKD2, RAB5B, USP48	assay:**PUB:20065103**] **MAPK9** [HITS-CLIP:**PUB:22473208**] **SNTB2** [Sequencing:**PUB:20371350**] **EPHA7** [HITS-CLIP:**PUB:22473208**] **WDR37** [PAR-CLIP:**PUB:21572407**, PAR-CLIP:**PUB:22012620**, CLASH:**PUB:23622248**] **RPS6KA5** [HITS-CLIP:**PUB:22473208**] **SSX2IP** [PAR-CLIP:**PUB:23592263**] **PTGFRN** [HITS-CLIP:**PUB:22473208**, HITS-CLIP:**PUB:23313552**] **MINK1** [Sequencing:**PUB:20371350**] **IKZF4** [PAR-CLIP:**PUB:21572407**] **GNPTAB** [PAR-CLIP:**PUB:21572407**] **SCD** [PAR-CLIP:**PUB:22012620**] **DPYSL2** [Sequencing:**PUB:20371350**, HITS-CLIP:**PUB:22473208**, CLASH:**PUB:23622248**] **AGO1** [HITS-CLIP:**PUB:22473208**, PAR-CLIP:**PUB:23592263**] **RRM2** [Sequencing:**PUB:20371350**] **PKD2** [Sequencing, PAR-CLIP:**PUB:20371350**, PAR-CLIP:**PUB:21572407**, HITS-CLIP:**PUB:22473208**, PAR-CLIP:**PUB:23446348**, PAR-CLIP:**PUB:23592263**] **RAB5B** [PAR-CLIP, HITS-CLIP:**PUB:21572407**] **USP48**

Showing 1 to 35 of 35 entries

First Previous 1 Next Last

Comparision with Tarbase miRNA/Target genes of Fig 6B S2B

MiRNA Data Portal (BIOCOS Life Sciences)

home miRNA Info miRNA Target miRNA Pathways

miRNAtoDisease miRNA GeneOntology

miRNA Target gene Information

Show 50 entries Search:

Export to xls Export to csv Export to txt

miRNA ID	Strong evidance	Weak Evidence	Evidence Type
hsa-let-7b-5p	ACVR1, CCND1, IRS2	MXD1, DPF2, SOX9, THBS1	[Luciferase reporter assay, Microarray, qRT-PCR:**PUB:22995917**] **ACVR1** [Immunoblot, Immunofluorescence, Luciferase reporter assay, qRT-PCR, Reporter assay, Other:**PUB:18379589**, immunoblot, Northern blot, Western blot:**PUB:20133835**, Microarray, Western blot, Annexin V-FITC:**PUB:23806108**, PAR-CLIP:**PUB:23592263**] **CCND1** [Flow, Luciferase reporter assay, qRT-PCR, Western blot:**PUB:24810113**, CLASH:**PUB:23622248**] **IRS2** [PAR-CLIP:**PUB:21572407**] **MXD1** [Proteomics:**PUB:18668040**] **DPF2** [Microarray:**PUB:17699775**] **SOX9** [pSILAC, Proteomics, Other:**PUB:18668040**, PAR-CLIP:**PUB:24398324**] **THBS1**
hsa-let-7c-5p	BCL2L1	MXD1, CCND1, THBS1	[Luciferase reporter assay, qRT-PCR, Western blot:**PUB:20347499**, Western blot:**PUB:22917031**] **BCL2L1** [PAR-CLIP:**PUB:21572407**] **MXD1** [PAR-CLIP:**PUB:23592263**] **CCND1** [PAR-CLIP:**PUB:24398324**] **THBS1**
hsa-let-7d-5p		MXD1, CCND1, THBS1	[PAR-CLIP:**PUB:21572407**] **MXD1** [PAR-CLIP:**PUB:23592263**] **CCND1** [Sequencing:**PUB:20371350**, PAR-CLIP:**PUB:24398324**] **THBS1**
hsa-let-7e-5p	CCND1	MXD1, THBS1, IRS2	[Luciferase reporter assay, qRT-PCR, Western blot:**PUB:21969366**, Western blot:**PUB:24100610**, PAR-CLIP:**PUB:23592263**] **CCND1** [PAR-CLIP:**PUB:21572407**] **MXD1** [PAR-CLIP:**PUB:24398324**] **THBS1** [CLASH:**PUB:23622248**] **IRS2**
hsa-let-7i-5p		MXD1, CCND1, THBS1	[PAR-CLIP:**PUB:21572407**] **MXD1** [PAR-CLIP:**PUB:23592263**] **CCND1** [PAR-CLIP:**PUB:24398324**] **THBS1**
hsa-miR-106a-5p	SLC2A3, CCND1	RAD23B, NFAT5, F3	[Luciferase reporter assay, qRT-PCR, Western blot:**PUB:24124917**] **SLC2A3** [qRT-PCR, Western blot:**PUB:23836497**, PAR-CLIP:**PUB:20371350**, PAR-CLIP, HITS-CLIP:**PUB:21572407**, PAR-CLIP:**PUB:22012620**,

miRNA ID	Strong evidance	Weak Evidence	Evidence Type
			PAR-CLIP:**PUB:23446348**, PAR-CLIP:**PUB:23592263**, PAR-CLIP:**PUB:24398324**] **CCND1** [CLASH:**PUB:23622248**] **RAD23B** [PAR-CLIP:**PUB:20371350**, PAR-CLIP:**PUB:21572407**, PAR-CLIP:**PUB:23446348**, PAR-CLIP:**PUB:23592263**] **NFAT5** [PAR-CLIP:**PUB:20371350**, PAR-CLIP:**PUB:21572407**, PAR-CLIP:**PUB:23446348**, PAR-CLIP:**PUB:23592263**] **F3**
hsa-miR-125b-5p	MXD1, MCL1, BCL2	RYBP	[Immunofluorescence, Immunohistochemistry, Luciferase reporter assay, qRT-PCR, Western blot:**PUB:23099851**] **MXD1** [Luciferase reporter assay, Western blot:**PUB:22824797**, qRT-PCR, Western blot:**PUB:22942733**] **MCL1** [Luciferase reporter assay, Western blot:**PUB:22293115**] **BCL2** [CLASH:**PUB:23622248**] **RYBP**
hsa-miR-139-5p	MCL1	SPRY4	[Immunohistochemistry, Luciferase reporter assay, qRT-PCR, Western blot:**PUB:23551751**, Western blot:**PUB:23557151**, PAR-CLIP:**PUB:23592263**] **MCL1** [PAR-CLIP:**PUB:23592263**] **SPRY4**
hsa-miR-148b-3p	ACVR1		[Luciferase reporter assay, qRT-PCR:**PUB:23227223**] **ACVR1**
hsa-miR-155-5p	CEP41, CCND1	RAD23B, FNDC3B, NFAT5, THBS1	[Reporter assay, Other:**PUB:20584899**] **CEP41** [Luciferase reporter assay, Western blot:**PUB:22858023**, Proteomics:**PUB:18668040**] **CCND1** [Proteomics:**PUB:18668040**] **RAD23B** [Proteomics:**PUB:18668040**] **FNDC3B** [HITS-CLIP:**PUB:22473208**] **NFAT5** [Proteomics:**PUB:18668040**] **THBS1**
hsa-miR-15a-5p	BCL2, CCND1	RAD23B, CPEB2, PAFAH1B1, OSBPL3, MCL1, SLC2A3, EFNB2	[Luciferase reporter assay, qRT-PCR, Western blot:**PUB:16166262**, Luciferase reporter assay:**PUB:17707831**, Luciferase reporter assay:**PUB:19478946**, Western blot:**PUB:19903841**, Western blot:**PUB:20876285**, Microarray, qRT-PCR:**PUB:17260024**, proteomics analysis:**PUB:18362358**, HITS-CLIP:**PUB:22473208**] **BCL2** [Luciferase reporter assay:**PUB:18931683**, Luciferase reporter assay:**PUB:19549910**, qRT-PCR, Luciferase reporter assay, Western blot:**PUB:19591824**, CLASH:**PUB:23622248**, PAR-CLIP:**PUB:24398324**] **CCND1** [PAR-CLIP:**PUB:20371350**, HITS-CLIP:**PUB:22473208**] **RAD23B** [HITS-CLIP:**PUB:22473208**] **CPEB2** [HITS-CLIP:**PUB:22473208**] **PAFAH1B1** [PAR-CLIP:**PUB:20371350**] **OSBPL3** [Microarray:**PUB:18362358**] **MCL1** [PAR-CLIP:**PUB:21572407**, PAR-CLIP:**PUB:23446348**] **SLC2A3** [PAR-CLIP:**PUB:20371350**,

miRNA ID	Strong evidance	Weak Evidence	Evidence Type
			PAR-CLIP:**PUB:22012620**] **EFNB2**
hsa-miR-16-5p	BCL2, CCND1	TMEM87A, RAD23B, CPEB2, PAFAH1B1, FNDC3B, OSBPL3, NRP1, MCL1, SLC2A3, EFNB2	[Luciferase reporter assay, qRT-PCR, Western blot:**PUB:16166262**, Luciferase reporter assay:**PUB:17351108**, Luciferase reporter assay:**PUB:17707831**, Luciferase reporter assay:**PUB:17877811**, Western blot, Reporter assay:**PUB:18449891**, Western blot:**PUB:19269153**, Western blot:**PUB:19903841**, Immunohistochemistry, Microarray, Western blot:**PUB:20643754**, Western blot:**PUB:20876285**, Western blot:**PUB:23907579**, Western blot:**PUB:24148817**, proteomics analysis:**PUB:18362358**, Sequencing:**PUB:20371350**, HITS-CLIP:**PUB:22473208**] **BCL2** [Western blot, Luciferase reporter assay:**PUB:18483394**, qRT-PCR, flow, Luciferase reporter assay:**PUB:18701644**, Luciferase reporter assay:**PUB:18931683**, Luciferase reporter assay:**PUB:19549910**, Luciferase reporter assay, Western blot:**PUB:19591824**, Western blot:**PUB:24148817**, PAR-CLIP:**PUB:24398324**] **CCND1** [Proteomics:**PUB:18668040**] **TMEM87A** [PAR-CLIP:**PUB:20371350**, HITS-CLIP:**PUB:22473208**] **RAD23B** [HITS-CLIP:**PUB:22473208**] **CPEB2** [HITS-CLIP:**PUB:22473208**] **PAFAH1B1** [pSILAC, Proteomics, Other:**PUB:18668040**, Sequencing:**PUB:20371350**] **FNDC3B** [Sequencing, PAR-CLIP:**PUB:20371350**, CLASH:**PUB:23622248**] **OSBPL3** [Proteomics:**PUB:18668040**] **NRP1** [Microarray:**PUB:18362358**] **MCL1** [PAR-CLIP:**PUB:21572407**, PAR-CLIP:**PUB:23446348**] **SLC2A3** [Proteomics:**PUB:18668040**, PAR-CLIP:**PUB:20371350**, PAR-CLIP:**PUB:22012620**] **EFNB2**
hsa-miR-17-5p	SOCS6, BCL2, CCND1, RND3, ETV1	PAFAH1B1, MCL1, NFAT5, NPAS2, F3, THBS1, ABCA1	[Luciferase reporter assay, qRT-PCR, Western blot:**PUB:24801601**] **SOCS6** [Luciferase reporter assay:**PUB:19666108**, Luciferase reporter assay, qRT-PCR, Western blot:**PUB:24280866**] **BCL2** [Luciferase reporter assay, qRT-PCR, Western blot:**PUB:18695042**, Luciferase reporter assay:**PUB:21283765**, PAR-CLIP:**PUB:20371350**, PAR-CLIP, HITS-CLIP:**PUB:21572407**, PAR-CLIP:**PUB:22012620**, PAR-CLIP:**PUB:23446348**, PAR-CLIP:**PUB:23592263**, PAR-CLIP:**PUB:24398324**] **CCND1** [Luciferase reporter assay, qRT-PCR, Western blot:**PUB:22132820**] **RND3** [Luciferase reporter assay, qRT-PCR, microarray, Western blotting:**PUB:23969726**] **ETV1** [HITS-CLIP:**PUB:22473208**] **PAFAH1B1** [HITS-CLIP:**PUB:22473208**] **MCL1** [PAR-CLIP:**PUB:20371350**, PAR-CLIP:**PUB:21572407**, HITS-CLIP:**PUB:22473208**, PAR-CLIP:**PUB:23446348**,

miRNA ID	Strong evidance	Weak Evidence	Evidence Type
			PAR-CLIP:**PUB:23592263**, CLASH:**PUB:23622248**] **NFAT5** [CLASH:**PUB:23622248**] **NPAS2** [PAR-CLIP:**PUB:20371350**, PAR-CLIP:**PUB:21572407**, PAR-CLIP:**PUB:23446348**, PAR-CLIP:**PUB:23592263**] **F3** [Microarray, qRT-PCR:**PUB:20940405**] **THBS1** [HITS-CLIP:**PUB:22473208**] **ABCA1**
hsa-miR-181a-5p	MCL1, BCL2	FNDC3B, OSBPL3, NRP1, ENAH	[Luciferase reporter assay:**PUB:22610076**] **MCL1** [Luciferase reporter assay, qRT-PCR, Western blot, Reporter assay, Other:**PUB:20162574**, Immunoblot, Western blot:**PUB:21958558**, Western blot:**PUB:22209977**, Luciferase reporter assay:**PUB:22285729**, Luciferase reporter assay:**PUB:22610076**, Western blot:**PUB:24002437**, Western blot:**PUB:24335172**] **BCL2** [HITS-CLIP:**PUB:22473208**] **FNDC3B** [HITS-CLIP:**PUB:22473208**] **OSBPL3** [Microarray:**PUB:17612493**, Sequencing:**PUB:20371350**] **NRP1** [Microarray:**PUB:17612493**] **ENAH**
hsa-miR-182-5p	BCL2, THBS1		[Flow, Luciferase reporter assay, qRT-PCR, Western blot:**PUB:22848417**, qRT-PCR:**PUB:23936432**] **BCL2** [Luciferase reporter assay:**PUB:24053448**] **THBS1**
hsa-miR-183-5p		SLC2A3, CCND1	[CLASH:**PUB:23622248**] **SLC2A3** [Sequencing:**PUB:20371350**, PAR-CLIP:**PUB:23592263**] **CCND1**
hsa-miR-18a-5p	BCL2	PAFAH1B1, CCND1, F3	[Luciferase reporter assay, qRT-PCR, Western blot:**PUB:24280866**] **BCL2** [Sequencing:**PUB:20371350**] **PAFAH1B1** [PAR-CLIP:**PUB:21572407**] **CCND1** [PAR-CLIP:**PUB:20371350**, PAR-CLIP:**PUB:21572407**, PAR-CLIP:**PUB:23592263**] **F3**
hsa-miR-18b-5p		CCND1, F3	[PAR-CLIP:**PUB:21572407**] **CCND1** [PAR-CLIP:**PUB:20371350**, PAR-CLIP:**PUB:21572407**, PAR-CLIP:**PUB:23592263**] **F3**
hsa-miR-192-5p	BCL2	SPRY4, PAFAH1B1, FNDC3B, EFNB2	[Luciferase reporter assay, qRT-PCR, Reporter assay, Microarray, Other:**PUB:19074876**] **BCL2** [Microarray:**PUB:19074876**] **SPRY4** [Microarray:**PUB:19074876**] **PAFAH1B1** [Microarray:**PUB:19074876**] **FNDC3B** [Microarray:**PUB:19074876**] **EFNB2**
hsa-miR-195-5p	SLC2A3, BCL2, CCND1	RAD23B, CPEB2, PAFAH1B1, OSBPL3, EFNB2	[Luciferase reporter assay, Western blot:**PUB:22265971**, PAR-CLIP:**PUB:21572407**, PAR-CLIP:**PUB:23446348**] **SLC2A3** [Luciferase reporter assay, qRT-PCR, Western blot:**PUB:20727858**, Immunohistochemistry, Western blot:**PUB:23451060**, HITS-CLIP:**PUB:22473208**] **BCL2**

miRNA ID	Strong evidance	Weak Evidence	Evidence Type
			[Luciferase reporter assay, Western blot:**PUB:22217655**, Luciferase reporter assay:**PUB:23383003**, Immunohistochemistry, qRT-PCR, Western blot:**PUB:23451060**, Western blot:**PUB:24402230**, PAR-CLIP:**PUB:24398324**] **CCND1** [PAR-CLIP:**PUB:20371350**, HITS-CLIP:**PUB:22473208**] **RAD23B** [HITS-CLIP:**PUB:22473208**] **CPEB2** [HITS-CLIP:**PUB:22473208**] **PAFAH1B1** [PAR-CLIP:**PUB:20371350**] **OSBPL3** [PAR-CLIP:**PUB:20371350**, PAR-CLIP:**PUB:22012620**] **EFNB2**
hsa-miR-19b-3p	MXD1	ACVR1, THBS1	[Luciferase reporter assay, qRT-PCR, Western blot:**PUB:24675462**] **MXD1** [Sequencing:**PUB:20371350**] **ACVR1** [PAR-CLIP:**PUB:23592263**] **THBS1**
hsa-miR-20a-5p	MCL1, BCL2, CCND1, ETV1	LPHN3, PAFAH1B1, NFAT5, F3, THBS1, ABCA1	[Immunohistochemistry, Luciferase reporter assay, qRT-PCR, Western blot:**PUB:23594563**, HITS-CLIP:**PUB:22473208**] **MCL1** [Luciferase reporter assay:**PUB:19666108**] **BCL2** [Luciferase reporter assay, qRT-PCR, Western blot:**PUB:18695042**, Luciferase reporter assay:**PUB:21283765**, Western blot:**PUB:23836497**, PAR-CLIP:**PUB:20371350**, PAR-CLIP, HITS-CLIP:**PUB:21572407**, PAR-CLIP:**PUB:22012620**, PAR-CLIP:**PUB:23446348**, PAR-CLIP:**PUB:23592263**, PAR-CLIP:**PUB:24398324**] **CCND1** [Luciferase reporter assay, qRT-PCR, microarray, Western blotting:**PUB:23969726**] **ETV1** [CLASH:**PUB:23622248**] **LPHN3** [HITS-CLIP:**PUB:22473208**] **PAFAH1B1** [PAR-CLIP:**PUB:20371350**, PAR-CLIP:**PUB:21572407**, HITS-CLIP:**PUB:22473208**, PAR-CLIP:**PUB:23446348**, PAR-CLIP:**PUB:23592263**] **NFAT5** [PAR-CLIP:**PUB:20371350**, PAR-CLIP:**PUB:21572407**, PAR-CLIP:**PUB:23446348**, PAR-CLIP:**PUB:23592263**] **F3** [Microarray, qRT-PCR:**PUB:20940405**] **THBS1** [HITS-CLIP:**PUB:22473208**] **ABCA1**
hsa-miR-221-3p			
hsa-miR-222-3p			
hsa-miR-24-3p	CCND1	NFAT5, SLC2A3	[Luciferase reporter assay, Microarray, qRT-PCR, Western blot:**PUB:23174883**] **CCND1** [qRT-PCR:**PUB:19296935**, Microarray:**PUB:19748357**] **NFAT5** [Microarray:**PUB:19748357**] **SLC2A3**

miRNA ID	Strong evidance	Weak Evidence	Evidence Type
hsa-miR-25-3p		FNDC3B, GFPT2	[CLASH:**PUB:23622248**] **FNDC3B** [PAR-CLIP:**PUB:21572407**, PAR-CLIP:**PUB:22012620**] **GFPT2**
hsa-miR-27b-3p	THBS1, ABCA1	RYBP	[Luciferase reporter assay:**PUB:24177325**] **THBS1** [Luciferase reporter assay, qRT-PCR, Western blot:**PUB:24608080**] **ABCA1** [HITS-CLIP:**PUB:22473208**] **RYBP**
hsa-miR-30a-5p		PTPRK, TMEM87A, RAD23B, FNDC3B, NFAT5, BCL2L1, RND3, THBS1	[pSILAC, Proteomics, Other:**PUB:18668040**] **PTPRK** [pSILAC, Proteomics, Other:**PUB:18668040**] **TMEM87A** [pSILAC, Proteomics, Other:**PUB:18668040**, HITS-CLIP:**PUB:22473208**] **RAD23B** [Proteomics:**PUB:18668040**] **FNDC3B** [HITS-CLIP:**PUB:22473208**] **NFAT5** [Proteomics:**PUB:18668040**] **BCL2L1** [Proteomics:**PUB:18668040**] **RND3** [Proteomics:**PUB:18668040**] **THBS1**
hsa-miR-30b-5p	BCL2	RAD23B, NFAT5, GFPT2	[Luciferase reporter assay:**PUB:24178239**] **BCL2** [HITS-CLIP:**PUB:22473208**] **RAD23B** [HITS-CLIP:**PUB:22473208**] **NFAT5** [Sequencing:**PUB:20371350**] **GFPT2**
hsa-miR-30c-5p		RAD23B, NFAT5	[HITS-CLIP:**PUB:22473208**] **RAD23B** [HITS-CLIP:**PUB:22473208**] **NFAT5**
hsa-miR-30e-5p		RAD23B, NFAT5	[HITS-CLIP:**PUB:22473208**] **RAD23B** [HITS-CLIP:**PUB:22473208**] **NFAT5**
hsa-miR-342-3p		RAD23B, FNDC3B, HDAC9	[PAR-CLIP:**PUB:22012620**] **RAD23B** [HITS-CLIP:**PUB:23313552**] **FNDC3B** [HITS-CLIP:**PUB:23824327**] **HDAC9**
hsa-miR-361-5p		RAD23B	[PAR-CLIP:**PUB:21572407**] **RAD23B**
hsa-miR-454-3p		ACVR1	[PAR-CLIP:**PUB:21572407**] **ACVR1**
hsa-miR-93-5p		MXD1, PAFAH1B1, RYBP, MCL1, NFAT5, NPAS2, CCND1, RND3, F3, ABCA1	[Microarray, qRT-PCR:**PUB:22815788**] **MXD1** [HITS-CLIP:**PUB:22473208**] **PAFAH1B1** [Sequencing:**PUB:20371350**] **RYBP** [HITS-CLIP:**PUB:22473208**] **MCL1** [PAR-CLIP:**PUB:20371350**, PAR-CLIP:**PUB:21572407**, HITS-CLIP:**PUB:22473208**, PAR-CLIP:**PUB:23446348**, PAR-CLIP:**PUB:23592263**] **NFAT5** [Microarray, qRT-PCR:**PUB:22815788**] **NPAS2** [Sequencing, PAR-CLIP:**PUB:20371350**, PAR-CLIP, HITS-CLIP:**PUB:21572407**, PAR-CLIP:**PUB:22012620**, PAR-CLIP:**PUB:23446348**, PAR-CLIP:**PUB:23592263**, PAR-CLIP:**PUB:24398324**] **CCND1** [Sequencing:**PUB:20371350**] **RND3** [Sequencing, PAR-CLIP:**PUB:20371350**, PAR-CLIP:**PUB:21572407**, PAR-CLIP:**PUB:23446348**,

miRNA ID	Strong evidance	Weak Evidence	Evidence Type
			PAR-CLIP:**PUB:23592263**] **F3**<br>[HITS-CLIP:**PUB:22473208**] **ABCA1**

Showing 1 to 35 of 35 entries

First Previous 1 Next Last
